# Supplementary material for: Cohort Profile: Childhood morbidity and potential non-specific effects of the childhood vaccination programmes in the Nordic countries (NONSEnse): register-based cohort of children born 1990–2017/2018
Source: BMJ Open. 2023 Feb 10;13(2):e065984. doi: 10.1136/bmjopen-2022-065984 (PMC9923270; doi:10.1136/bmjopen-2022-065984)
Supplement: Supplementary data [file bmjopen-2022-065984supp001.pdf]

Lexis diagram for Danish cohort including vaccination programme and data availability

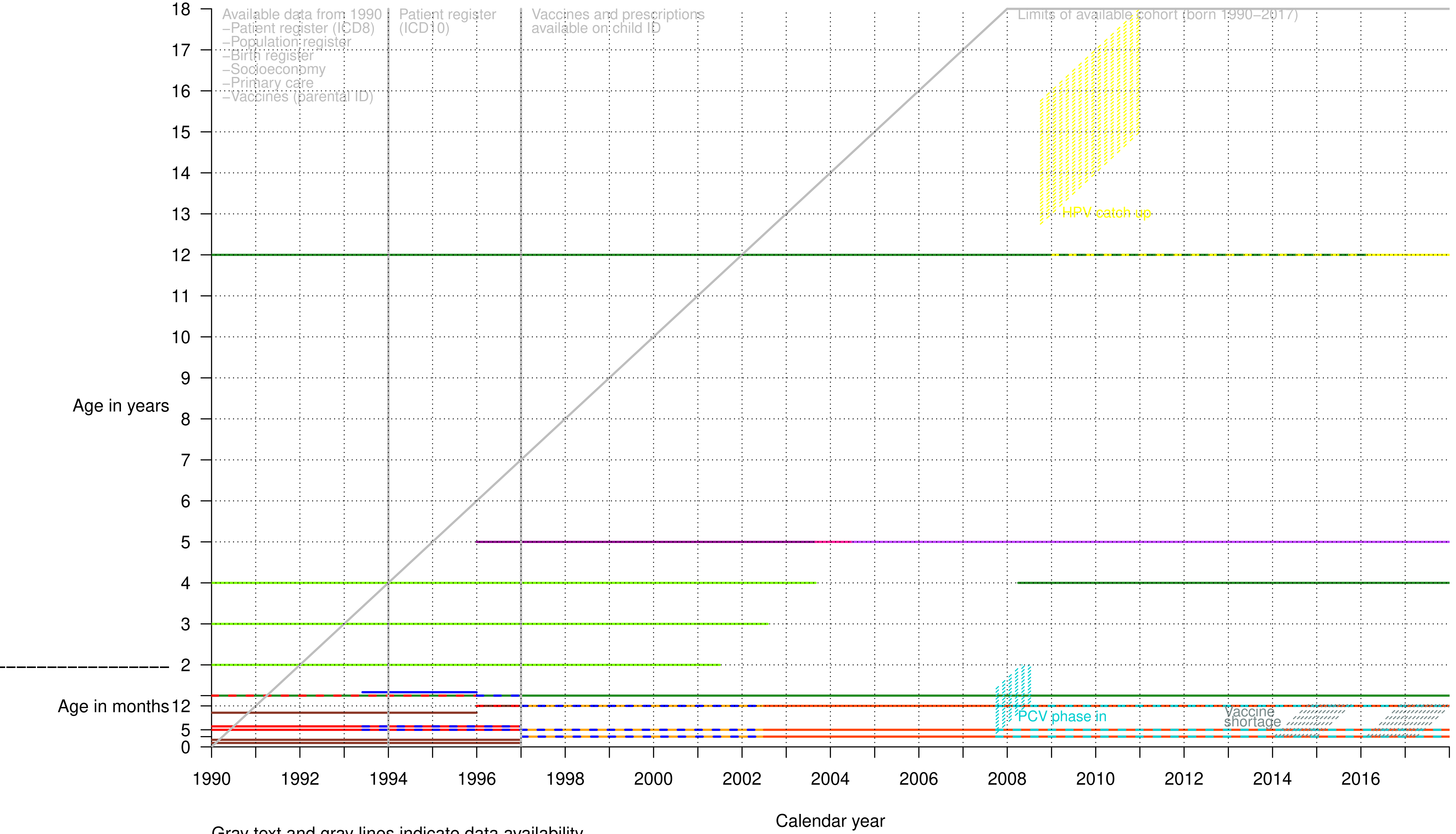

Gray text and gray lines indicate data availability

Color codes for vaccines:

wP ; DT–IPV; DTaP–IPV; DTaP–IPV–Hib; Hib ; PCV ; MMR; OPV; DT; DTaP ; DTaP–IPV; HPV

Abbreviations for vaccines:

Non–live vaccines: wP=whole cell pertussis; D=diphtheria; T=tetanus; IPV=inactivated polio vaccine; aP=pertussis vaccine(acellular);

Hib=Haemophilus influenzae type b; PCV=pneumococcal conjugate vaccine; HPV=Human papilloma virus

Live vaccines: MMR=measles–mumps–rubella; OPV=oral polio vaccine

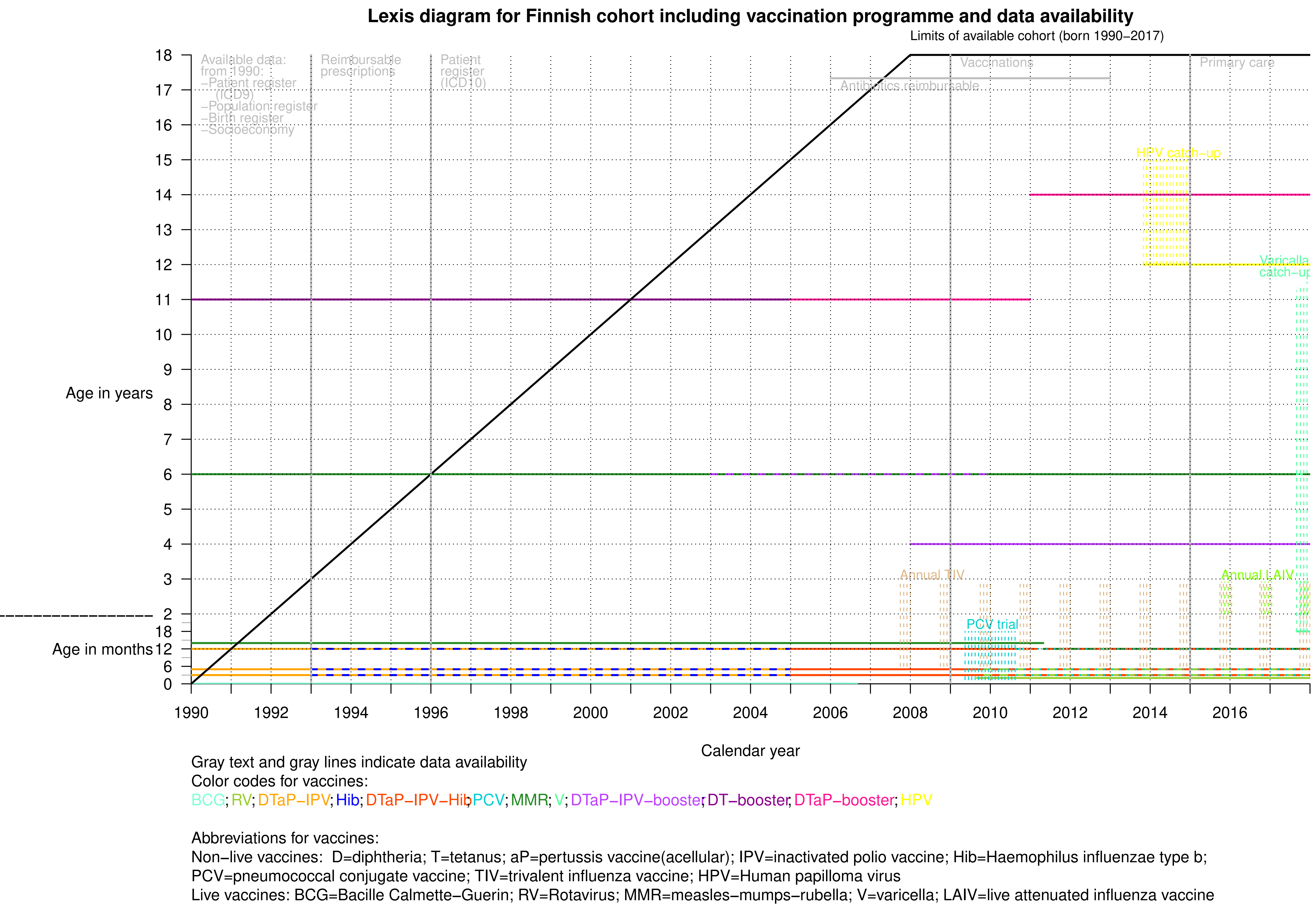
Lexis diagram for Finnish cohort including vaccination programme and data availability
Limits of available cohort (born 1990–2017)
Available data:
from 1990:
–Patient register
(ICD9)
–Population register
–Birth register
–Socioeconomy
Reimbursable
prescriptions
Patient
register
(ICD10)
Vaccinations
Primary care
Antibiotics reimbursable
HPV catch–up
Varicalla
catch–up
Annual TIV
Annual LAIV
PCV trial
Age in years
Age in months
Calendar year
1990
1992
1994
1996
1998
2000
2002
2004
2006
2008
2010
2012
2014
2016
Gray text and gray lines indicate data availability
Color codes for vaccines:
BCG; RV; DTaP–IPV; Hib; DTaP–IPV–Hib; PCV; MMR; V; DTaP–IPV–booster; DT–booster; DTaP–booster; HPV
Abbreviations for vaccines:
Non–live vaccines: D=diphtheria; T=tetanus; aP=pertussis vaccine(acellular); IPV=inactivated polio vaccine; Hib=Haemophilus influenzae type b;
PCV=pneumococcal conjugate vaccine; TIV=trivalent influenza vaccine; HPV=Human papilloma virus
Live vaccines: BCG=Bacille Calmette–Guerin; RV=Rotavirus; MMR=measles–mumps–rubella; V=varicella; LAIV=live attenuated influenza vaccine

Lexis diagram for Norwegian cohort including vaccination programme and data availability

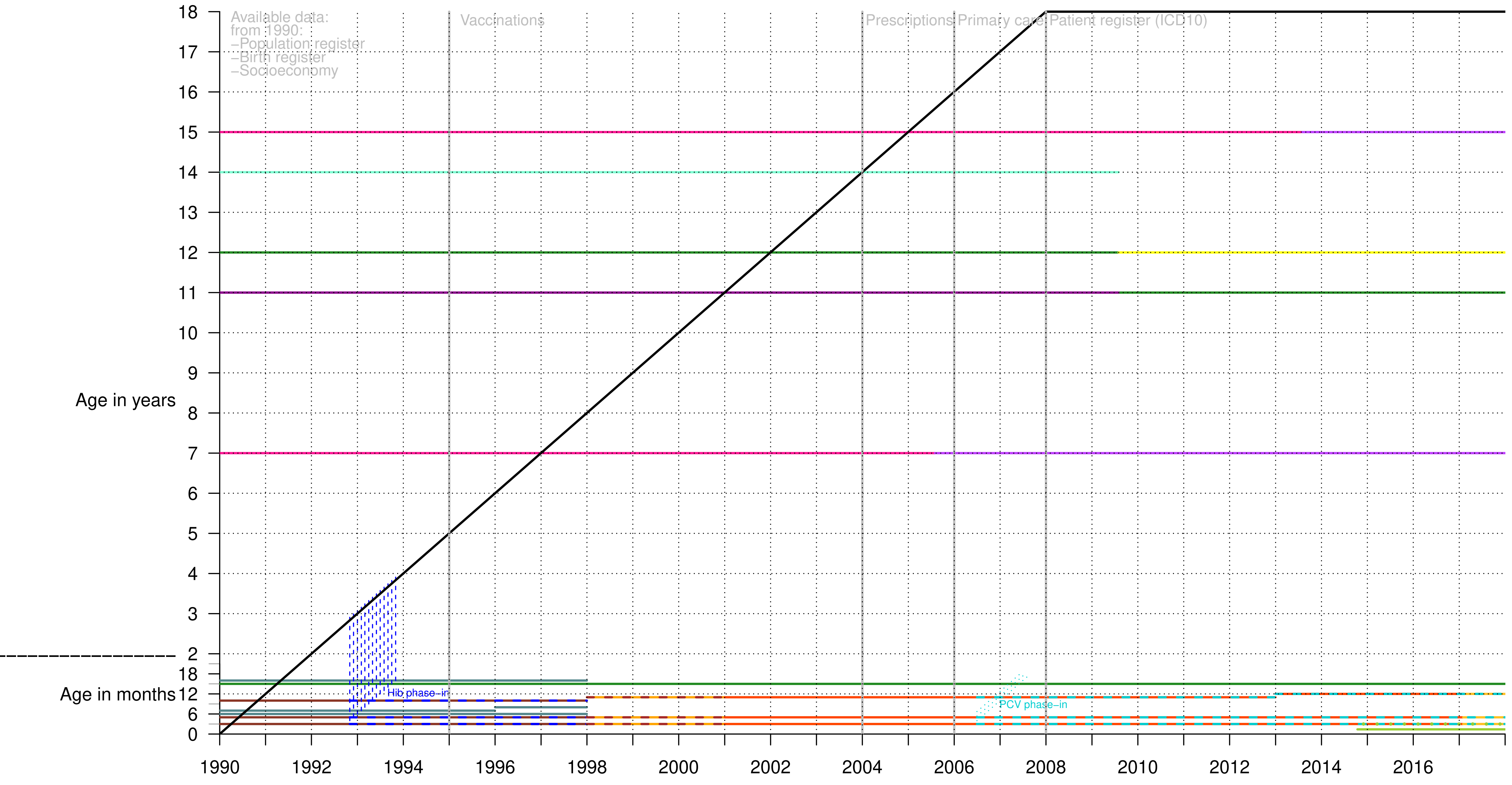

Gray text and gray lines indicate data availability
Color codes for vaccines:
RV; DTwP; Hib; DTaP; IPV–Hib; DTaP–IPV–Hib; PCV; DTaP–IPV–Hib–HepB; IPV; MMR; IPV–booster; DTaP–IPV–booster; DT–booster; HPV; BCG

Abbreviations for vaccines:
Non–live vaccines: D=diphtheria; T=tetanus; wP=whole–cell pertussis vaccine; Hib=Haemophilus influenzae type b;aP=pertussis vaccine(acellular);
IPV=inactivated polio vaccine; PCV=pneumococcal conjugate vaccine; HepB=Hepatitis B; HPV=Human papilloma virus
Live vaccines: RV=Rotavirus; MMR=measles–mumps–rubella; BCG=Bacille Calmette–Guerin

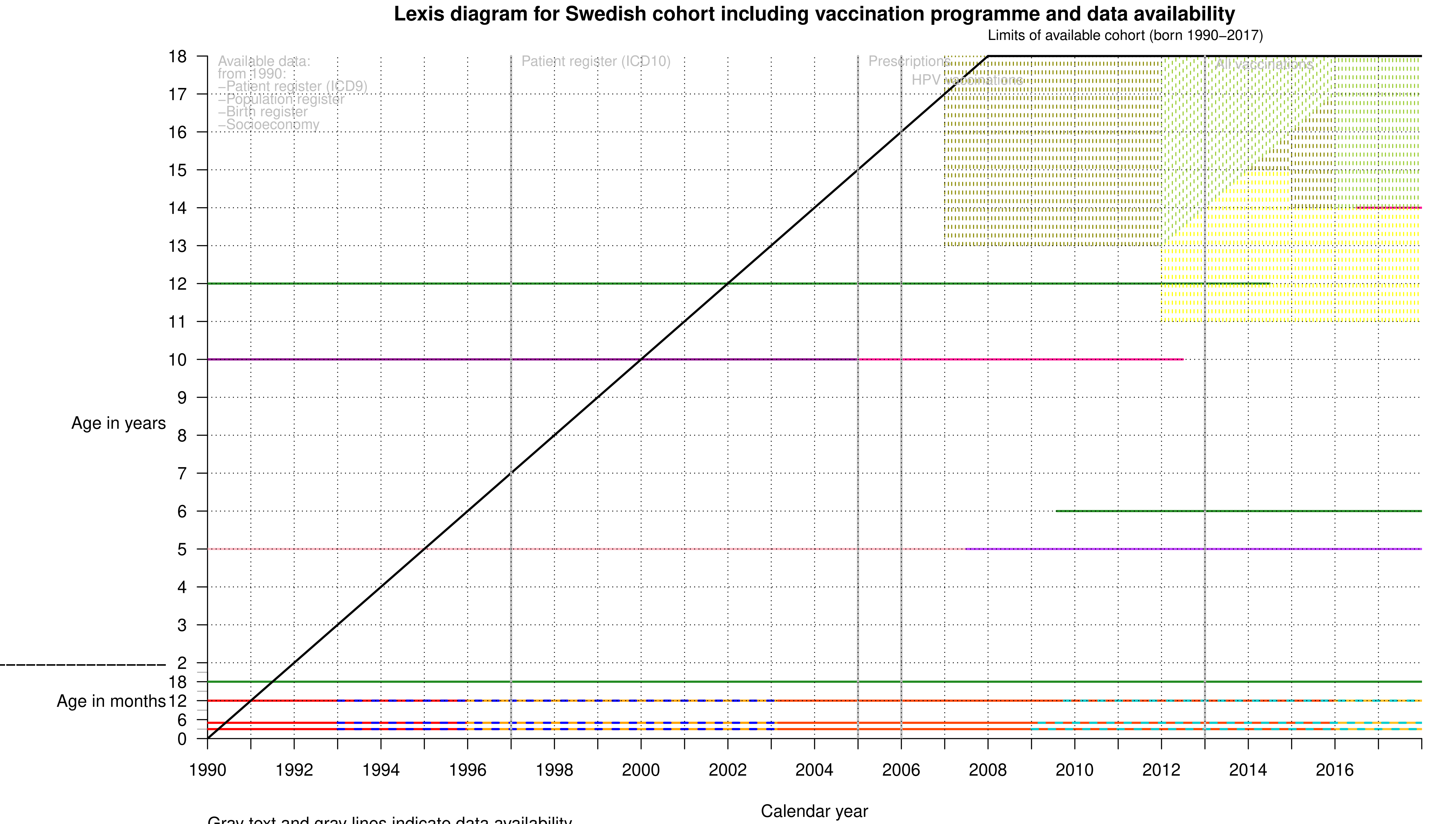

Gray text and gray lines indicate data availability
Color codes for vaccines:
DT−IPV; Hib; DTaP−IPV; DTaP−IPV−Hib; PCV; DTaP−IPV−Hib−HepB; MMR; IPV−booster; DTaP−IPV−booster; DT−booster; DTaP−booster; HPV−recommended age; HPV−own payment with partly subsidy; HPV catch−up
Abbreviations for vaccines:
Non−live vaccines: D=diphtheria; T=tetanus; aP=pertussis vaccine(acellular); IPV=inactivated polio vaccine; Hib=Haemophilus influenzae type b; PCV=pneumococcal conjugate vaccine; HepB=Hepatitis B; HPV=Human papilloma virus
Live vaccines: MMR=measles−mumps−rubella
