# Supplementary material for: Cohort Profile: Childhood morbidity and potential non-specific effects of the childhood vaccination programmes in the Nordic countries (NONSEnse): register-based cohort of children born 1990–2017/2018
Source: BMJ Open. 2023 Feb 10;13(2):e065984. doi: 10.1136/bmjopen-2022-065984 (PMC9923270; doi:10.1136/bmjopen-2022-065984)
Supplement: Supplementary data [file bmjopen-2022-065984supp002.pdf]

Link to *Contents*

NONSense Common Data Model

Nov 28th 2022

Link to *Contents*

Gehrt L, *et al. BMJ Open* 2024; 13:e065984. doi: 10.1136/bmjopen-2022-065984

Link to *Contents*

Contents

Contents 2
Introduction 3
Background/Event tables 4
  Table: prescriptions 4
  Table: hospital_contacts 4
  Table: population1 5
  Table: birth_characteristics 6
  Table: Vaccines 7
  Table: socio_economy 8
Source of data in each country 11
  Table: prescriptions 11
  Table: hospital_contacts 14
  Table: population1 19
  Table: birth_charcteristics 28
  Table: Vaccines 36
  Table: socio_economy 40
Appendix: Vaccine categorization: 50

Link to *Contents*

Link to Contents

Introduction

The Common Data Model (CDM) is a tool for documentation of data preparation and generation of uniform datasets across the Nordic countries (Denmark, Finland, Norway, and Sweden). The aim is to construct a number of uniform background datasets and event tables, which share the same name and entail the same variables, labels and values across countries. Datasets with the exact same format across Countries enables sharing of syntax-files for study analyses.
The CDM is a working document, which will be updated according to country specific data preparation, and expanded as all necessary information will be transformed into background/event tables. In the end, the background/event tables will include all necessary information to conduct all future studies (morbidity/incidence studies and vaccination studies).

The current version presents data content and preparation as per April 2022

The CDM contains 1) “Background/Event tables”, and 2) “Source of data and data preparation in each country”.

Background/Event tables: include information on the name of the dataset to be used by NONSEnse and format and labeling of each variable within the dataset.

Source of data in each country: includes a description of the information on the source register, and source variables, which have been used to generate the variables in the background/event tables. These tables furthermore entail information on important notes (i.e data breaks, limitations such as i.e. restricted information on redeemed prescriptions in Finland) and data preparation (how have the source variables been modified to generate the variables in the background/event tables). The tables on source of data in each country have been filled in by the individual countries following country specific data preparation.

Link to Contents

Link to Contents

Background/Event tables

Table: prescriptions

Description: Table of all redeemed prescriptions (included atc codes in each country is listed in “source of data and datapreparation”) among individuals in the study population.
Structure: 1 observation (line) for every redeemed pharmaceutical.

Variables:

**Variable**	**Label**	**values**
id	Personal id of the child	string
b_date	Birthdate of the child	Date Format (%dD_m_Y)
sex	Sex of the child as recorded in the dataset	1=”male”<br>2=”female”
redeemdate	Date of redeeming the prescription	Date Format (%dD_m_Y)
atc	Full atc code for the redeemed drug	String (7 digits) use capital letters i.e “J01AA01”

Table: hospital_contacts

Description: Table of all diagnoses (both main diagnosis and all other diagnoses) for somatic patients including information on sex and date of birth for all children in the study population. Note that a patient can have multiple diagnoses attached to the same contact.
Structure: 1 observation (line) for each diagnosis received

Variables:

Variable	label	values
id	Personal id of the child	String
b_date	Birthdate of the child	Date Format (%dD_m_Y)
sex	Sex of the child as recorded in the dataset	1=”male”<br>2=”female”
adm_date	Date of admission	Date Format (%dD_m_Y)
discharge_date	Date of discharge	Date Format (%dD_m_Y)
diag	ICD diagnosis code	String (For ICD-10 codes use max 4 digits e.g. A063)
diagtype	Type of diagnosis	1=”Main diagnosis”<br>2=”Other diagnosis”
type_contact	Type of hospital contact	Categorical:<br>1=”inpatient”<br>2=”emergency room patient”<br>3=”outpatient”<br>4=”outpatient or emergency room patient”

Link to Contents

Link to Contents

Table: population1

Description: Background table including information follow-up for each child in the study population. The dataset only includes information on the child's first stay in the country (first in_date and first cens_date is recorded).

Structure: one line for each child

Variable	label	values
id	Personal id of the child	string
b_date	Birthdate of the child	Date Format (%dD_m_Y)
sex	Sex of the child as recorded in the dataset	1="male"<br>2="female"
origin	Born in the country or abroad	1="born in-country"<br>2="born abroad"<br>9= "Unknown"
in_date	Date of entering the cohort	Date Format (%dD_m_Y)
in_reason	Reason for entering the cohort	1="birth"<br>2="immigration"
cens_date	First date of censoring	Date Format (%dD_m_Y)
cens_reason	Reason for being censored	1="death"<br>2="out migration"<br>3="other"
m_id	id of mother	string
f_id	id of father	string
m_age	Mothers age in years at time of delivery	Numeric (discrete)
m_origin	Maternal origin at birth	1="born in-country"<br>2="born abroad"<br>9= "Unknown"
p_origin	Paternal origin at birth	1="born in-country"<br>2="born abroad"<br>9= "Unknown"

Link to Contents

Link to *Contents*

Table: birth_characteristics

Structure: one line for each child in the study population

variable	Label	values	Legal values
id	Personal id of the child	string	
b_weight	Birthweight of child (gram)	Numeric	100-9990
ga	Gestational age (full weeks)	Numeric (discrete)	
sectio	Delivered by caesarean section	0=”not delivered by caesarean section”<br>1=”delivered by caesarean section”<br>9=” unknown”	
smoke	Maternal smoking or snuff at any point during pregnancy	0= ”no”<br>1= “smoking (or snuff) during pregnancy”<br>9= “unknown”	
singleton	singleton	0=”no”<br>1=”yes”<br>9=” unknown”	
child_order	Child order (including the child itself)	Numeric (discrete)	

Link to *Contents*

Link to Contents

Table: Vaccines

variable	Label	values
id	Personal id of the child	string
vacdate	Date of vaccination	Date Format (%dD_m_Y)
vaccine	Type of vaccine administered	Categorical (see coding in appendix ”vaccine categorization”)
credibility	Credibility indication of vaccine information	1=no duplicate<br>2= duplicate same vaccine removed<br>3=duplicate related vaccine removed (keep vaccine that aligns with vaccination schedule)<br>4= duplicate related vaccine removed (none of the vaccines align with vaccination schedule)<br>5= duplicate related vaccine removed (vaccines given outside the vaccination schedule)
TB_endemic	Vaccine recommendations in accordance with connections to TB endemic countries	0=not risk group<br>1=risk group<br>9=not relevant
HepB_endemic	Vaccination recommendations in accordance with connections to HepB endemic countries	0=not risk group<br>1=risk group<br>9=not relevant

Prioritization for duplicate selection:

1. Remove same vaccines (variable name: “vaccine”, see appendix “vaccine categorization”) given 14 days or less after the previous vaccine for the same child (if DTP is registered on day 0, 10 and 20, only remove the vaccine registered at day 10) – *keep the earliest registration*
    i. *Credibility=2*
2. Remove vaccines from the same type of vaccines (variable name: “type” see appendix “vaccine categorization”) given 14 days or less after the previous vaccine of the same type. Register vaccine as given on the earliest date within the duplicate combination **prioritize within combinations:**
    a. Keep vaccine that aligns with vaccination schedule according to ***age*** and ***year of vaccination***
        i. Credibility =3
    b. If no vaccine aligns with vaccination schedule but type and age correspond to timing of childhood vaccinations keep the vaccine that protects against most conditions
        i. Credibility=4
    c. If vaccines are given outside ages for recommended vaccination according to the vaccination program – keep the vaccine that protects against most conditions

        i. Credibility=5

Link to Contents

Link to Contents

Table: socio_economy

Assign information to all children in the study population. If a child has no registrations in the socio economic datasets variables should be coded as 9 or 99="unknown" as described in the table below.

Overall note on timing of information:

Variables ending with "_b" indicate that information is from birth of the child. Depending on the set up of the register information we will use the date or year of birth to obtain the information. If information is not available for the date or year of birth, we will use information from the year after.

Variables ending with "_10y" indicate that information is from the year/date the child turns 10 years. Depending on the set up of the register information we will use the date or year of turning 10 years to obtain the information.

variable	Label	values	Legal values	Notes
id	Personal id of the child	string		
inc_quin_b	Household income quintile at year of birth of the child	1=first (lowest)<br>2=second<br>3= third<br>4= fourth<br>5= fifth (highest)<br>9="unknown"		Quintiles are calculated stratified on year (i.e., calculation of quintiles are done separately for each calendar year.<br>If several income variables are available, selection is based on this priority: 1: equated disposable household/family income; 2: disposable household/family income; 3:household/family income; 4: maternal disposable income; 5: maternal income.
inc_quin_10y	Household income quintile at the year of the child's 10th birthday	1=first (lowest)<br>2=second<br>3= third<br>4= fourth<br>5= fifth (highest)<br>9="unknown"		See notes under inc_quin_b
inc_quin_m_b	Maternal income quintile at year of birth of the child	1=first (lowest)<br>2=second<br>3= third<br>4= fourth		See notes under inc_quin_b

Link to Contents

Link to *Contents*

		5= fifth (highest)<br>9=”unknown”		
inc_quin_m_10y	Maternal income quintile at the year of the child’s 10th birthday	1=first (lowest)<br>2=second<br>3= third<br>4= fourth<br>5= fifth (highest)<br>9=”unknown”		See notes under inc_quin_b
n_children_b	Number of children below 18 years in the household including the child itself at year of birth of the child	Numeric discrete<br><br>99=”unknown”	>=1	
n_children_10y	Number of children below 18 years in the household including the child itself at the year of the child’s 10th birthday	Numeric discrete<br><br>99=”unknown”	>=1	
single_parent_b	Single parenthood at year of birth of the child	0=No<br>1=Yes<br>9=”unknown”		
single_parent_10y	Single parenthood at the year of the child’s 10th birthday	0=No<br>1=Yes<br>9=”unknown”		
m_education_b	Maternal highest attained education at year of birth of the child	1=Low education (ISCED2011 level 0-2)<br>2=Medium education (ISCED2011 level 3-4)<br>3=High education (ISCED2011 level 5-8)<br>9=”unknown”		International Standard Classification of Education (ISCED) 2011 coded into main groups. Read more in reference 1 below the table.
m_education_10y	Maternal highest attained education at	1=Low education (ISCED2011 level 0-2)		See notes under m_education_b.

Link to *Contents*

Link to *Contents*

	the year of the child's 10th birthday	2=Medium education (ISCED2011 level 3-4)<br>3=High education (ISCED2011 level 5-8)<br>9="unknown"		

Reference 1 for ISCED: https://ec.europa.eu/eurostat/statistics-explained/index.php/International_Standard_Classification_of_Education_(ISCED)#Implementation_of_ISCED_2011_.28levels_of_education.29

Link to *Contents*

Link to Contents

Source of data in each country

Table: prescriptions

	Denmark		Finland		Norway		Sweden	
Variable	Source and description	Important notes and data preparation	Source and description	Important notes and data preparation	Source and description	Important notes and data preparation	Source and description	Important notes and data preparation
id	Original name in the Danish data: "pnr". Pseudonomised unique personal identification number for linkage between registers, created by Statistics Denmark. It is linkable (by Statistics Denmark) to the original personal identification number (CPR number) assigned to all Danish residents and used when reporting to all national registers.	Renamed from "pnr"	Obtained from KELA Register: "KELA etuusrekisteri", Table:"Lääkeostot", Variable: "HETU"<br><br>Register: "Kanta Reseptikeskus" Table: "KANTA:RESEPTI.LAAKETOIMITUKSET" Variable: "PATIENT_ID"<br><br>THL pseudonymised the original personal identification code (in these registers HETU and PATIENT_ID) to unique personal identification number for linkage between registers. THL data management can link the id back to original personal identification code.	Statistics Finland pseudonymised HETU and PATIENT_ID	Obtained from Register: "The Norwegian Prescription Database" (NorPD) Pseudonomised unique personal identification number for linkage between registers	Renamed from "pasient_lopenr_pdb2471"	Created by Statistics Sweden Pseudonomised unique personal identification number for linkage between registers	Renamed from lopnr

Link to Contents

Link to *Contents*

b_date	Obtained from the Danish National Health Data Agency. Register: "CPR-Registret" Table: "t_person" Variable: "d_foddato"	Renamed from "d_foddato"	Obtained from KELA Obtained from KELA Register: " KELA etuusrekisteri", Table:"Lääkeostot", Variable: "HETU"<br><br>Register: "Kanta Reseptikeskus" Table: "KANTA:RESEPTI.LAAKETOIMITUKSET" Variable: "PATIENT_ID"	Extracted from "HETU" before pseudonymisation was done.<br><br>Extracted from "PATIENT_ID" before pseudonymisation was done.	Obtained from The National Population Register	We have received information on month and year of birth, but not day. For each individual, we have therefore generated a random integer between 1 and length of their month of birth. Using this random integer as day of birth, everyone is assigned an exact birth date.	Obtained from Statistics Sweden Register: "Register över totalbefolkningen, RTB" Variable: "fodddatum"	Renamed from fodddatum
sex	Obtained from the Danish National Health Data Agency. Register: "CPR-Registret" Table: "t_person" Variable: "C_KON" Sex as recorded by personal identification number.	sex=1 "male" if C_KON is "M" sex=2 "female" if C_KON is "K"	Obtained from KELA Register: " KELA etuusrekisteri", Table:"Lääkeostot", Variable: "HETU"<br><br>Register: "Kanta Reseptikeskus" Table: "KANTA:RESEPTI.LAAKETOIMITUKSET" Variable: "PATIENT_ID"	Extracted from "HETU" and "PATIENT_ID" before pseudonymisation was done. sex=1 "male" sex=2 "female"	Obtained from The National Population Register.	Renamed from "kjonn"	Obtained from Statistics Sweden Register: "RTB" Variable: "kon"	Renamed from "kon"
redeemdate	Obtained from Statistics Denmark. Register: "Lægemiddeldatabasen" Variable: "EKSD" Date of redeeming the prescription	Renamed from "EKSD"	Obtained from KELA Register: " KELA etuusrekisteri", Table:"Lääkeostot", Variable: "OSTOPV"<br><br>Register: "Kanta Reseptikeskus" Table: "KANTA:RESEPTI.LAAKETOIMITUKSET" Variable: "CREATION_DATE" Date of redeeming the prescription	Renamed from "OSTOPV" Renamed from "CREATION_DATE"	Obtained from Register: "NorPD" Variable: "UtleveringsDato" Date of redeeming the prescription	Renamed from "UtleveringsDato"	Obtained from Socialstyrelsen Register: "Läkemedelsregisteret" Variable: "edatum"	Renamed from "edatum". (Date of redeeming the prescription)

Link to *Contents*

Link to Contents

atc	Obtained from statistics Denmark.<br>Register: "Lægemiddeldatabasen"<br>Variable: "ATC"<br>ATC code of purchased drug	All prescriptions with ATC group D, J, R, S and V01, including all sublevels.<br>Renamed from "ATC"	Obtained from KELA Register: " KELA etuusrekisteri"<br>Table:" Lääkeostot"<br>Variable: "ATC"<br><br>Register: "Kanta Reseptikeskus"<br>Table: "KANTA:RESEPTI.LAAKETOIMITUKSET"<br>Variable: "ATC_CODE"<br><br>ATC code of purchased drug	All prescriptions with ATC groups D07, D11AH, J, R01, R03, R06, S01G, S03 and V01, including all sublevels.<br>V01 only from KELA data.<br>In Korvattavat lääkkeet only reimbursable products.<br>Reimbursement of antibiotics:<br>< 2006 no reimbursement if cheap<br>2006-2012: all antibiotics were reimbursed<br>>2012: individual products not reimbursed"<br><br>Duplicates removed: if same purchase (same id, redeemdate and atc) was found from both registers only one of them was included in the data.	Obtained from Register: "NorPD"<br>Variable: "ATCKode"<br>ATC code of purchased drug	All prescriptions with ATC group D, J, R, S and V01, including all sublevels<br>Renamed from "ATCKode"	Obtained from Socialstyrelsen<br>Register: "Läkemedelsregisteret"<br>Variable: "atc"	ATC code of purchased drug. The data from Sweden included all prescriptions within ATC groups D, J, R, S and V01, including all sublevels

Link to Contents

Link to *Contents*

Table: hospital_contacts

	Denmark		Finland		Norway		Sweden	
Variable	Source and Description	Important notes and data preparation	Source and Description	Important notes and data preparation	Source and Description	Important notes and data preparation	Source and Description	Important notes and data preparation
							In the original Swedish register data, each admission date is a separate line with all diagnoses and other information included in one line. The dataset has been reshaped to long format with one line for each diagnosis	
id	Original name in the Danish data: “pnr”. Pseudonomised unique personal identification number for linkage between registers, created by Statistics Denmark. It is linkable (by Statistics Denmark) to the original personal identification number (CPR number) assigned to all Danish residents and used when reporting to all national registers.	Renamed from “pnr”	Obtained from THL Register: “the Finnish National Patient Register THL=Hilmo” Table: “Perustiedot/Asiakas, potilas” Variable: “HT”<br><br>THL pseudonymised the original personal identification code (in this register HT) to unique personal identification number for linkage between registers. THL data management can link the id back to original personal identification code.	Statistics Finland pseudonymised HT with their own id for the remote user system.	Obtained from the Norwegian National Patient Register	Renamed from “ pasientlopenr_pdb2471”	Created by Statistics Sweden Pseudonomised unique personal identification number for linkage between registers	Renamed from “lopnr”

Link to *Contents*

Link to Contents

b_date	Obtained from the Danish National Health Data Agency. Register: “CPR-Registret” Table: “t_person” Variable: “d_foddato”	Renamed from “d_foddato”	Obtained from THL Register: “Hilmo” Table: “” Variable: “SYNTAIKA”	Renamed from “SYNTAIKA”	Obtained from The National Population Register	We have received information on month and year of birth, but not day. For each individual, we have therefore generated a random integer between 1 and length of their month of birth. Using this random integer as day of birth, everyone is assigned an exact birth date.	Obtained from Statistics Sweden Register: “RTB” Variable: “fodddatum”	Renamed from “fodddatum”
sex	Obtained from the Danish National Health Data Agency. Register: “CPR-Registret” Table: “t_person” Variable: “C_KON” Sex as recorded by personal identification number.	sex=1 “male” if C_KON is “M”<br>sex=2 “female” if C_KON is “K”	Obtained from THL Register: “Hilmo” Table: “Perustiedot/Asiakas, potilas” Variable: “SP”	Renamed from “SP”	Obtained from The National Population Register Variable: “kjonn”	Renamed from “kjonn”	Obtained from Statistics Sweden Register: “RTB” Variable: “kon”	Renamed from “kon”<br><br>Note: There were some discrepancies regarding sex in the two registries (RTB and Patientregistret), circa 1100 cases regarding inpatients and circa 2900 regarding outpatients. We used the information from Statistics Sweden.

Link to Contents

Link to Contents

adm_date	Obtained from the Danish National Health Data Agency. Register: Danish national patient registry Table: T_ADM Variable: D_INDDTO	Renamed from "D_INDDTO"<br><br>For outpatient contacts with multiple visits adm_date is recoded according to the date of visit ("D_AMBDTO" from the table "t_bes")	Obtained from THL Register: "Hilmo" Table: "Tulotiedot" Variable: ""TUPVA"	Extracted from "TUPVA" which contain the date and time of arrival	Obtained from Register: Norwegian National Patient Register Variable: "innDato"	Renamed from "innDato"	Obtained from Socialstyrelsen Register: "Patientregistere t" Variable: "INDATUM"	Renamed from "INDATUM".<br><br>Inpatient visits:<br>- Date missing (n=6); left unchanged.<br><br>- Date registered as earlier than birth (n=103);<br>-- dropped observations if both date of admission and discharge came before birth (n=8),<br>-- replaced date of admission with date of birth if less than 15 days apart (n=68),<br>-- replaced month or year, to align with date of discharge (n=29).<br><br>- Date registered as later than discharge but not missing (n=11); adm_date and discharge_date were shifted.<br><br>Outpatient visits:<br>- Date missing (n=1,253); left unchanged
discharge_date	Obtained from the Danish National Health Data Agency. Register: Danish national patient registry Table: T_ADM Variable: D_UDDTO	Renamed from "D_UDDTO"<br><br>For contacts without a discharge date (N=1080) the discharge date is set as the last observed discharge date in the dataset+1day (11May2018)<br><br>For outpatient contacts, discharge date is recoded to be the same date as "adm_date".	Obtained from THL Register: "Hilmo" Table: "Poistumistiedot" Variable: "LPVM"	Extracted from "LPVM" which contain the date and time of discharge	Obtained from Register: "Norwegian National Patient Register" Variable: "utDato"	Renamed from "utDato". The data set only includes admissions that have ended, i.e. utDato before Dec 31, 2018.<br><br>75 contacts had missing utData. These were either outpatient contacts (n=69) or daycare procedures (n=6). utDato was defined innDato in these cases	Obtained from Socialstyrelsen Register: "Patientregistere t" Variable: ""UTDATUM"	For inpatient visits, the variable was renamed from "UTDATUM".<br><br>For outpatient visits, there was no corresponding variable, and the discharge date was therefore created to be equal to the admission date.

Link to Contents

Link to *Contents*

diag	Obtained from the Danish National Health Data Agency. Register: Danish national patient registry Table: T_DIAG Variable: C_DIAG	Renamed from "C_DIAG"<br><br>Diagnosis coded as ICD 8 until December 31 1994, hereafter coded using ICD 10.<br><br>Danish specification letters to the ICD-10 codes removed and the administrative letter "D" in front of all codes removed: Values changed to string4 format (i.e DA011a→A011)<br><br>Diagnoses other than the main or other diagnoses are excluded. Diagnoses with modifications indicating that the diagnosis cannot be validated are excluded (c_diagmod==1 | 2).	Obtained from THL Register: "Hilmo" Table: "Hoitotiedot" Variable: "PDGO, PDGE, SDGO, SDGE"	Renamed from PDGO, PDGE, SDGO, SDGE ICD-codes ICD-codes V01-Y98 not available, codes O00-O99 were not analysed	Obtained from Register: "Norwegian National Patient Register"	Original dataset has one record for each hospital contact with variables hovedtilstand_1, hovedtilstand_2, bitilstand_1, …, bitilstand_19 that contain ICD 10 diagnosis codes. The variables were renamed diag1, diag2, diag3, … where diag1 and diag2 correspond to the 2 primary diagnoses. The dataset was reshaped to long format containing one observation per diagnosis with variables diag, containing the ICD-10 codes and diag_ind = 1, 2, 3, …	Obtained from Socialstyrelsen Register: "Patientregistere t" Variables: "HDIA" and "DIAGNOS1_30"	The variable "DIAGNOS1_30" can contain up to 30 different diagnoses. It was therefore split to create separate variables for each sequential diagnosis. Duplicate codes within each observation and the code _atc were removed.
diagtype	Obtained from the Danish National Health Data Agency. Register: Danish national patient registry Table: T_DIAG Variable: C_DIAGTYPE	Renamed from variable "C_DIAGTYPE" Recoded: C_DIAGTYPE: "A"= "main diagnosis" C_DIAGTYPE: "B"= "other diagnosis" A patient can have multiple other diagnoses for the same contact. Excluding diagnoses other than main or other (i.e temporary diagnoses or additional diagnosis ("tillægsdiagnose").	Obtained from THL Register: "Hilmo" Table: "Hoitotiedot" Variable: "PDGO, PDGE, SDGO, SDGE"	1=main diagnosis: PDGO and PDGE 2=add diagnosis: SDGO and SDGE		diagtype = 1 if diag_ind = 1 or diag_ind = 2 diagtype = 2 if diag_ind > 2	Obtained from socialstyrelsen Register: "Patientregistere t" Variables: "HDIA" and "DIAGNOS1_30"	Diagtype was coded as 1="Main diagnosis" if indicated in variable "HDIA". If no main diagnosis was listed in variable HDIA, the first diagnosis within variable "DIAGNOS1_30" was chosen as the main diagnosis. Other diagnoses listed within DIAGNOS1_30 were coded as 2="Other diagnosis".

Link to *Contents*

Link to Contents

type_contact	Obtained from the Danish National Health Data Agency.<br>Register: Danish national patient registry<br>Table: T_DIAG and t_bes<br>Variables: C_PATTYPE, D_AMBDTO	Renamed variable ”C_PATTYPE”<br><br>Recoded:<br>type_contact=1 “inpatient” if C_PATTYPE is “0” (inpatient) or “1” (Before year 2002 some patients were coded as “1= deldøgnspatienter” (≈part day patient)<br><br>type_contact=2 “emergency room contact” if C_PATTYPE is 3 “emergency room contact”.<br>Outpatient contacts (C_PATTYPE=2) admitted after year 2014 with “C_INDM”= “Acute” are coded as type_contact=2 ”emergency room patient”<br><br>type_contact=3 “outpatient contact” if C_PATTYPE=2 before year 2014 or C_PATTYPE=2 and c_indm is not 1 from and including year 2014<br><br>In Denmark we have some long outpatient contacts with multiple visit dates (D_AMBDTO) during the contact. Each visit date is coded as an independent outpatient contact. All diagnoses within the original outpatient contact is recorded for each visit.	Obtained from THL<br>Register: “Hilmo”<br>Table: “Perustiedot/Hoitojakso tai avohoitokäynti”<br>Variable: “PALA” and ”EA”	All visits with EA = 98 were omitted<br>(EA= special branches of medicine, 98=general practice)<br>- type_contact = 1, if PALA = 1 or PALA = 6 (inpatient)<br>- type_contact = 2, if PALA = 91 (emergency)<br>- type_contact = 3, if PALA is not 1, 6 or 91 (outpatient, not emergency)<br>PALA: 1 = inpatient ward, 6 = rehabilitation ward 91 = emergency room visit	Obtained from Register: “Norwegian National Patient Register”<br>Variable: “”	Based on the variables Behandlingsniva3 and Aktivitetskategori3:<br>For contacts with utDato in 2008-2014:<br><br>IF Behanglingsniva3 = 1 OR Behandlingsniva3 = 2 THEN type_contact = 1<br><br>ELSE IF Behandlingsniva3 = 3 THEN type_contact = 4<br>For contacts with utDato in 2015-2018:<br><br>IF Aktivitetsjkategori3 = 1 OR Aktivitetskategori3 = 2 THEN type_contact = 1<br>ELSE IF Aktivitetskategori3 = 3 THEN type_contact = 4	Obtained from Socialstyrelsen<br>Register: “Patientregisteret”	Variable coded based on which source file the data came from: in- or outpatient data. All data in the outpatient-file was coded = 4, as emergency room visits could not be distinguished.<br>(A variable for emergency room visits [VERKS_AKUT] was only included in the patient registry in 2016 and therefore not part of our data request.).

Link to Contents

Link to Contents

Table: population1

	**Denmark**		**Finland**		**Norway**		**Sweden**	
Variable	Source and Description	Important notes and data preparation	Source and Description	Important notes and data preparation	Source and Description	Important notes and data preparation	Source and Description	Important notes and data preparation
id	Original name in the Danish data: "pnr". Pseudonomised unique personal identification number for linkage between registers, created by Statistics Denmark. It is linkable (by Statistics Denmark) to the original personal identification number (CPR number) assigned to all Danish residents and used when reporting to all national registers.	Renamed from "pnr"	Obtained from: Register: Population register Table: VTJ.HENKILO Variable: hetu<br><br>Table: VTJ.HENKILO_HETU Variable: hetu_voimassa<br><br>THL pseudonymised the original personal identification code (in this register "hetu") to unique personal identification number for linkage between registers. THL data management can link the id back to original personal identification code.	Person included only if hetu_voimassa (=id is valid) is checked.<br><br>Statistics Finland pseudonymised "hetu" with their own id for the remote user system.	Obtained from: SSB (Statistics Norway) Register: "National Population Register"	Renamed from pasientlopenr_pdb2471	Created by Statistics Sweden Register: Registret över totalbefolkningen (RTB) Variable: lopnr	Pseudonomised unique personal identification number for linkage between registers<br><br>Renamed from "lopnr".
b_date	Obtained from the Danish National Health Data Agency. Register: "CPR-Registret" Table: "t_person" Variable: "d_foddato"	Renamed from "d_foddato"	Obtained from: Register: Population register Table:VTJ.HENKILO Variable: syntymapaiva	Renamed from syntymapaiva	Obtained from: SSB (Statistics Norway) Register: "National Population Register"	For all individuals in population1 as well as their parents, we have received information on month and year of birth, but not day. For each individual, we have therefore generated a random integer between 1 and length of their month of birth. Using this random integer as day of birth, everyone is assigned an exact birth date.	Obtained from Statistics Sweden Register: Registret över totalbefolkningen (RTB) Variable: fodddatum	Renamed from "fodddatum"

19

Link to Contents

Link to Contents

sex	Obtained from the Danish National Health Data Agency. Register: "CPR-Registret" Table: "t_person" Variable: "C_KON" Sex as recorded by personal identification number.	sex=1 "male" if C_KON is "M" sex=2 "female" if C_KON is "K"	Obtained from: Register: Population register Table: VTJ.HENKILO Variable: sukupuoli	sex=1 "male" if lapsen sukupuoli is "mies" sex=2 "female" if lapsen sukupuoli is "nainen"	Obtained from: SSB (Statistics Norway) Register: "National Population Register"	Renamed from kjonn	Obtained from Statistics Sweden Register: Registret över totalbefolkningen (RTB) Variable: kon	Renamed from "kon"

Link to Contents

Link to *Contents*

origin	Obtained from the Danish National Health Data Agency. Register: "CPR-Registeret" Table: "T_FODESTED" Variables: "fodested_kode", "fodested_tekst"<br><br>Variables from table: population1; in_date, cens_date are used to define if there is uncertain origin	Children are categorised as:<br>1="born in-country" if fodested_kode=000 or 208 (Denmark),<br>2= "foreign born" if fodested_kode is not 000 or 208<br>9="unknown" if fodested_kode=000 or 208 (Denmark) and if there is date of inmigration not preceded by an outmigration (In this case we cannot be certain that the child is born in Denmark as it appears to have migrated to Denmark after the date of birth)	Obtained from: Register: Population register Table:VTJ.HENKILO Variable: "syntymakunta"	1 = born in country, if the code of syntymakunta (birth municipality is not 200 or NA (not available)<br>2 = born abroad, if syntymakunta is 200<br>3 = uncertain foreign or in-country, if syntymakunta is NA, 198,199 or 000 (children born abroad were excluded as only minority of them had immigration dates available in the THL's population register copy, in which the follow-up begin in 2014, also children with uncertain origin were excluded)	Obtained from: SSB (Statistics Norway) Register: "National Population Register"	Based on the variables in_date (see below) and "fodeland". Origin is coded as 1 if country of birth is Norway (fodeland = 0) and in_date is equal to date of birth. Origin is coded as 2 if country of birth is any other country. Origin is coded as 9 if country of birth is Norway and in_date is later than date of birth.	Obtained from Statistics Sweden Register: Registret över totalbefolkningen (RTB) Variable: UtlSvBakg<br><br>Combined with data from the National Board of Health and Welfare Register: Medical Birth Registry	Recoded from: "UtlSvBakg" where<br>11 = Born abroad<br>12 = Born in the country with two foreign-born parents<br>21 = Born in the country with one native and one foreign born parent<br>22 = Born in the country with two native born parents.<br><br>Individuals were coded 1 = "born in-country", if UtlSvBakg = 12, 21 or 22, and 2 = "born abroad", if UtlSvBakg = 11.<br><br>Individuals were coded 9 = "Unknown" if registered as born in country (UtlSvBakg = 12, 21 or 22) but also had a registered immigration date not preceded by an emigration date. (In this case we cannot be certain that the child was born in the country as it appeared that they have immigrated after the date of birth.)<br><br>If the individual was initially coded as 9 "Unknown", but was registered in the medical birth registry, they were recoded as 1 = "born in-country".

Link to *Contents*

Link to Contents

in_date	Obtained from the Danish National Health Data Agency. Register: “CPR-Registeret” Table: “T_FODESTED” Variables: ”D_FODDATO”<br><br>Table: “ T_ADRESSE_UDLAND_HIST ” Variables: ”C_ANNKOR”, ”D_INDREJSE_DATO” Table: ” T_ARKIV_ADRESSE_UDLAND_HIST” Variables: ”C_ANNKOR”, ”D_INDREJSE_DATO”<br><br>Obtained from NONSense CDM Table: Population1 Variable: ”origin”, ”cens_date”	in_date is defined as date of birth ”D_FODDATO” if ”origin” is 1=”born in-country” . in_date is defined as the first date of in-migration ”D_INDREJSE_DATO” if origin is not 1=”born in-country”.	Obtained from: Register: Population register Table:VTJ.HENKILO Variable: ”syntymapaiva” and	If born in country (origin=1), equal to the date of birth = syntymapaiva	Obtained from: SSB (Statistics Norway) Register: “National Population Register”	Based on the variables “regstatus”, “regstatusdato”, “forstdato” and “fodeland”. Indate is defined as forstdato if invkat = B (immigrants). “forstdato” is the date of first registration in the Population Registry. The variable is only defined for persons with invkat =B (immigrants). Otherwise (invkat = A, C, E, F or G), indate is defined as a person’s earliest regstatusdato with regstatus = 1 (Bosatt). In general, individuals who have been residents in Norway since birth, will be registered with regstatus = 1 and corresponding regstatusdato = date of birth. However, regstatus is only available as of January 1 each year. If a person’s regstatus has changed more than once during a calendar year, we only have information about the most recent change. Therefore, in_date was set to date of birth for individuals with country of birth Norway who died or emigrated in their year of birth even if they do not have a record with regstatus = 1 and regstatusdato = date of birth. Note: cross-checked with the Birth Registry, and > 98% of children with country of birth	Variable created based on information from Statistics Sweden Register: Registret över totalbefolkningen (RTB) Variables: fodddatum and datum [migration], posttyp [migration]	If born in country (origin=1), equal to the date of birth = fodddatum If born outside the country (origin=2), equal to first date of immigration If unknown origin (=9), equal to first date of immigration

Link to Contents

Link to *Contents*

						Norway who died or emigrated in their year of birth have a record in the Birth Registry. Thus, it is a reasonable assumption that these children have been residents of Norway since birth.		
in_reason	Obtained from the Danish National Health Data Agency. Register: "CPR-Registeret"<br><br>Table: "T_FODESTED" Variables: "D_FODDATO"<br><br>Table: " T_ADRESSE_UDLAND_HIST " Variables: "C_ANNKOR", "D_INDREJSE_DATO"<br><br>Table: " T_ARKIV_ADRESSE_UDLAND_HIST" Variables: "C_ANNKOR", "D_INDREJSE_DATO"<br><br>Obtained from NONSense CDM Table: Population1 Variable:"in_date"	in_reason is categorised as:<br>1="birth" if in_date is obtained from "D_FODDATO"<br>2="immigration" if in_date is obtained from D_INDREJSE_DATO	Obtained from: Register: Population register Table:VTJ.HENKILO Variable: "syntymapaiva"	1 = Birth,<br>if born in the country (origin=1)	Obtained from: SSB (Statistics Norway) Register: "National Population RegisterBefolkning	in_reason is coded as 1 if origin = 1. in_reason is coded as 2 if origin = 2 or origin = 9.	Variable created based on information from Statistics Sweden Register: Registret över totalbefolkningen (RTB) Variables: foddatum, datum, posttyp	1 = Birth,<br>if born in the country (origin=1)<br>2 = Immigration,<br>if born abroad (origin=2) or unknown origin (origin=9)

Link to *Contents*

Link to *Contents*

cens_date	Obtained from the Danish National Health Data Agency. Register: "CPR-Registret" Table: "t_person" Variables: "D_STATUS_HEN_START", "C_STATUS"<br><br>Table: " T_ADRESSE_UDLAND_HIST " Variables: "C_ANNKOR", "D_UDREJSE_DATO"<br><br>Table: " T_FORSVIND_HIST" Variables: "C_ANNKOR", "D_FORSVIND_DATO"	Cens_date is defined as the first date of either 1)"D_STATUS_HEN_START" if "C_STATUS" is "90"=death, "20"=CPR number for tax purposes, "70"=disappearing, "80"=out-migration or 2) D_UDREJSE_DATO or 3) D_FORSVIND_DATO.	Obtained from: Register: Population register Table: VTJ.HENKILO Variable: "KUOLINPVM" and Register: Statistic Finland Table: Variable: "kuolinpäivä" Variable: ensimmäinen maastamuuttopäivä<br><br>Table: VTJ.HENKILO Table: KOTIKUNTAHISTORIA: Variable: "kotikunta" and "kunta muuttopaiva"	Equal to date of emigration, if such has occurred, otherwise equal to date of death.<br><br>Emigration from Population register (select min (kunta_muuttopvm) from vtj.henkilo_kotikuntahistoria and kunta='200')<br><br>Ensimmäinen maastamuuttopäivä=first emigration date available only in remote user system Fiona	Obtained from: SSB (Statistics Norway) Register: "National Population RegisterBefolkning	Based on the variables "regstatus", "regstatusdato", and "dodsdato". Date of emigration was defined as a person's earliest regstatusdato with regstatus = 3 (emigration). Date of death was defined as dodsdato. We only have information on month and year of death. Exact date of date was assigned as a random integrer within the month of death. cens_date was set to date of emigration if emigration occurred before date of 18th birthday or January 1, 2019. cens_date was set to date of death if death occurred before date of 18th birthday or January 1, 2019, unless date of death was preceded by date of emigration (N = 40).	Variable created based on information from Statistics Sweden Register: Registret över totalbefolkningen (RTB) Variables: Doddatum, datum [migration], posttyp [migration]	Equal to date of emigration, if such an event had been registered, otherwise equal to date of death.

Link to *Contents*

Link to Contents

cens_reason	Obtained from the Danish National Health Data Agency. Register: "CPR-Registret" Table: "t_person" Variables: "D_STATUS_HEN_START", "C_STATUS"<br><br>Table: " T_ADRESSE_UDLAND_HIST " Variables: "C_ANNKOR", "D_UDREJSE_DATO"<br><br>Table: " T_FORSVIND_HIST" Variables: "C_ANNKOR", "D_FORSVIND_DATO"	Cens_reason is categorized as: 1= "death" if cens_date is obtained from C_STATUS="90" (death) 2="out-migration" if cens_date is obtained from C_STATUS="80" (outmigration) or from D_UDREJSE_DATO 3="other" if cens_date is obtained from C_STATUS="20" (CPR for tax purposes" | "70" (disappearing) or from D_FORSVIND_DATO.<br><br>If more than one cens_reason is registered for the first cens_date preference is given to 1="death"	Obtained from: Register: Population register Table: VTJ.HENKILO Variable:kuolinpvm, muuttopvm	1 = Death<br>If subject died<br>2 = Emigration<br>If subject emigrated	Obtained from: SSB (Statistics Norway) Register: "National Population RegisterBefolkning	cens_reason was coded as 1 if cens_date = date of death. cens_reason is coded as 2 if cens_date = date of emigration.	Variable created based on information from Statistics Sweden Register: Registret över totalbefolkningen (RTB) Variables: Doddatum, datum [migration], posttyp [migration]	1 = Death, if there was a date of death registered in variable Doddatum.<br>2 = Out migration, if there was a registered migration out of the country

Link to Contents

Link to Contents

m_id	Obtained from the Danish National Health Data Agency. Register: "CPR-Registret" Table: "t_person" Variable: "V_MOR_PNR_ENCRYPTED"	Renamed from "V_MOR_PNR_ENCRYPTED"	Obtained from: Birth register Table: Äidin henkilötiedot Variable: aiti_hetunnus<br><br>THL pseudonymised the original personal identification code (in this register "aiti_hetunnus") to unique personal identification number for linkage between registers. THL data management can link the id back to original personal identification code.	Renamed from "aiti_hetunnus" and pseudonymised by Statistics Finland for data lingage.	Obtained from: SSB (Statistics Norway) Register: "National Population Register" Variable: Løpenummer mor	Renamed from lopenr_mor_pdb2471	Obtained from Statistics Sweden Register: Flergenerationsregistret	Renamed from "LopNrMor"
f_id	Obtained from the Danish National Health Data Agency. Register: "CPR-Registret" Table: "t_person" Variable: "V_FAR_PNR_ENCRYPTED"	Renamed from "V_FAR_PNR_ENCRYPTED"	Obtained from: Statistics Finland	Not available for THL. Pseudonymised id for data linkage in Statistics Finland	Obtained from: SSB (Statistics Norway) Register: "National Population Register" Variable: Løpenummer far	Renamed from lopenr_far_pdb2471	Obtained from Statistics Sweden Register: Flergenerationsregistret	Renamed from "LopNrFar"
m_age	Obtained from the Danish National Health Data Agency. Register: "MFR" linked with- Register: "CPR-Registeret" Table: "t_person" Variable: "d_foddato"	Id of the mother is obtained from the dataset "population1" (originally obtained from the CPR register). Using information on maternal birthday (d_foddato) and birthday of the child, Maternal age in years is calculated as age in whole years at time of delivery of the child.	Obtained from Register: Birth register Table: Äidin henkilötiedot Variable: aiti_ika	Renamed from aiti_ika	Obtained from: SSB (Statistics Norway) Register: "National Population RegisterBefolkning	Mother's age in whole years at time of birth of child. Based on the mother's assigned exact date of birth (b_date).	Obtained from Statistics Sweden Register: RTB Variable: datum_fodd	Calculated as mother's date of birth minus the child's date of birth, divided by 365, and rounded down to yield age in years.

Link to Contents

Link to Contents

m_origin	Obtained from the Danish National Health Data Agency. Register MFR Register: "CPR-Registeret" Table: "T_FODESTED" Variables: "fodested_kode", "fodested_tekst"<br><br>Variables from table: population1; in_date, cens_date are used to define if there is uncertain origin	Id of the mother is obtained from the dataset "population1" and linked with information from the CPR register 1="born in-country" if fodested_kode=000 or 208 (Denmark), 2= "born abroad" if fodested_kode is not 000 or 208 9="unknown" if information is missing	Obtained from Register: Statistics Finland Table: Variable: svaltio_aiti	Available only in the Fiona remote user system. svaltio_aiti = 246 -> 1 = "born in-country" svaltio_aiti != 246 (ts joku muu kuin Suomi) -> 2 = "born abroad" svaltio_aiti = NA (ts puuttuu) -> 9 = "Unknown"	Obtained from: SSB (Statistics Norway) Register: "National Population RegisterBefolk ning	Based on the variable "fodeland". m origin = 1 if mother's country of birth is Norway (fodeland = 0), m_origin = 2 if mother's country of birth is any other country, and m_origin = 9 if mother's country of birth is missing (n = 20,559).	Obtained from Statistics Sweden Register: RTB Variable: UtlSvBakg	Recoded from variable "UtlSvBakg" as described above for variable Origin in table Population 1.
p_origin	Obtained from the Danish National Health Data Agency. Register MFR Register: "CPR-Registeret" Table: "T_FODESTED" Variables: "fodested_kode", "fodested_tekst"<br><br>Variables from table: population1; in_date, cens_date are used to define if there is uncertain origin	Id of the father is obtained from the dataset "population1" and linked with information from the CPR register 1="born in-country" if fodested_kode=000 or 208 (Denmark), 2= "born abroad" if fodested_kode is not 000 or 208 9="unknown" if the information is missing	Obtained from Register: Statistics Finland Table: Variable: svaltio_isa	Available only in the Fiona remote user system. svaltio_isa = 246 -> 1 = "born in-country" svaltio_isa != 246 (ts joku muu kuin Suomi) -> 2 = "born abroad" svaltio_isa = NA (ts puuttuu) -> 9 = "Unknown"			Obtained from Statistics Sweden Register: RTB Variable: UtlSvBakg	Recoded from variable "UtlSvBakg" as described above for variable Origin in table Population 1.

Link to Contents

Link to Contents

Table: birth_charcteristics

	Denmark		Finland		Norway		Sweden	
Variable	Source and Description	Important notes and data preparation	Source and Description	Important notes and data preparation	Source and Description	Important notes and data preparation	Source and Description	Important notes and data preparation
id	Original name in the Danish data: “pnr”. Pseudonomised unique personal identification number for linkage between registers, created by Statistics Denmark. It is linkable (by Statistics Denmark) to the original personal identification number (CPR number) assigned to all Danish residents and used when reporting to all national registers.	Renamed from “pnr”	Obtained from Register: Birth Register Table: Variable: lapsi_hetunnus THL pseudonymised the original personal identification code (lapsi_hetunnus) to unique personal identification number for linkage between registers. THL data management can link the id back to original personal identification code.	Statistics Finland pseudonymised lapsi_hetunnus with their own id for the remote user system.		Renamed from “pasientlopenr_pdb2471”	Created by Statistics Sweden Pseudonomised unique personal identification number for linkage between registers	Renamed from lopnr

Link to Contents

Link to Contents

b_weight	Obtained from the Danish National Health Data Agency.<br>Register: “MFR” from 1997 and onwards, “Fødselsregisteret” before 1997<br>Table: “MFR”(from MFR), ”levendefødt” (From fødselsregisteret)<br>Variable: “vaegt_barn” (MFR), V_VAGT (fødselsregisteret)	Renamed from “vaegt_barn” and “ V_VAGT”<br>Registrations of birthweight less than 100g or higher than 9990g are categorized as missing	Obtained from Register: Birth Register<br>Table:<br>Variable: syntymapaino	Registrations of birthweight less than 100g or higher than 9990g are categorized as missing	Obtained from Register: Medical Birth Registry of Norway<br>Variable: vekt	Registrations of birthweight less than 100g or higher than 9990g are defined as missing	Obtained from Socialstyrelsen<br>Register: Medicinska födelseregistret<br>Variable: bvikt	Registrations of birthweight less than 100g or higher than 9990g were categorized as missing.

Link to Contents

Link to *Contents*

ga	Obtained from the Danish National Health Data Agency.<br>Register: "MFR" from 1997 and onwards, "Fødselsregisteret" before 1997<br>Table: "MFR"(from MFR), "levendefødt" (From fødselsregisteret)<br>Variable: "Gestationsalder_dage" (MFR), "V_SVLANGDE" (fødselsregisteret)	Derived from "Gestationsalder_dage" (ga in days) rounded down to whole weeks of gestation: ga=floor(gestationsalder_dage/7)<br><br>Renamed from V_SVLANGDE	Obtained from Register: Birth Register<br>Table:<br>Variable: kestovkpv	kestovkpv, ga will be notified as weeks, the days are not noted.<br><br>Ga <20 or >45 are coded as missing	Obtained from Register: Medical Birth Registry of Norway<br>Variable: svlen	ga is calculated as floor(svlen/7), where svlen is the length of gestation in days based on ultrasound estimation. If ultrasound is not available, the gestational length is calculated from the last menstrual period.	Obtained from Socialstyrelsen<br>Register: Medicinska födelseregistret<br>Variable: grvbs	Socialstyrelsen recommends using this variable (for the best estimated gestational age), over the variable grvfv (which is based on medical records).

Link to *Contents*

Link to *Contents*

sectio	Obtained from the Danish National Health Data Agency.<br>Register: “MFR” from 1997 and onwards, “Fødselsregisteret” before 1997<br>Table: “MFR”(from MFR), ”levendefødt” (From fødselsregisteret)<br><br>Variables: ” Markoer_kejsersnit” (MFR), B_I11, B_SECTIOF, B_SECTIOU (fødselsregisteret)	From MFR:<br>0=”not delivered by caesarean section” if they do not have any diagnosis code indicating caesarean section (”Markoer_kejsersnit”=missing)<br><br>1=”delivered by caesarean section” if they have a diagnosis code indicating caesarean section in the variable ”Markoer_kejsersnit”<br><br>Fødselsregisteret sectio=1 if B_I11=1 | B_SECTIOU=1 | B_SECTIOF=1<br><br>Otherwise sectio=0	Obtained from Register: Birth Register<br>Table:<br>Variable: synnytystapatunnus	Children are categorised as:<br>0=”not delivered by caesarean section” if synnytystapatunnus is 1-4<br><br>1=”delivered by caesarean section” if synnytystapatunnus is 5-8<br><br>9="unknown" if synnytystapatunnus=9 or missing	Obtained from Register: Medical Birth Registry of Norway<br><br>Variable: ksnitt	Information on delivery with c-section is obtained from the variable ksnitt. Possible values of ksnitt are<br>1 = Planned C-section<br>2 = Emergency C-section<br>9 = Unspecified C-section<br>If ksnitt is missing, sectio is coded as 0. Otherwise, sectio is coded as 1.	Obtained from Socialstyrelsen<br>Register: Medicinska födelseregistret<br>Variable: secmark	Variable renamed from secmark; coding unaltered: 0 = no, 1 =yes.

Link to *Contents*

Link to *Contents*

smoke	Obtained from the Danish National Health Data Agency. Register: "CPR-Registeret" Register: "MFR" from 1997 and onwards, "Fødselsregisteret" before 1997 Table: "MFR"(from MFR), "levendefødt" (From fødselsregisteret) Variable: "rygerstatus_moder" (MFR), B_RYGER (fødselsregisteret)<br><br>Obtained from the Danish National Health Data Agency. Register: Danish national patient registry Table: "T_ADM", "T_DIAG" Variables: pnr, recnum, D_INDDTO, D_UDDTO, C_ADIAG, C_TILDIAG,	Information from MFR, variable "RYGERSTATUS_MODER" Smoke=0 if rygerstatus_moder=0 Smoke=1 if rygerstaus moder >0 and <99 (indicating any smoking during pregnancy regardless of magnitude) Smoke=9 if rygerstatus_moder=99(unknown) or missing.<br><br>From fødselsregisteret: smoke=0 if B_RYGER=0 smoke=1 if B_RYGER=1 smoke=9 if B_RYGER=.<br><br>For some pregnancies especially in 1997 and partially in 1998, smoke information is not available in MFR, but we are able to subtract the information from the patient registry using the additional diagnosis "DUT00-DUT99".<br><br>Information about smoke is inserted from the patient registry if: a) the information is not present in MFR/fødselsregisteret; b) if the patient registry indicates smoking while MFR/fødselsregisteret indicates no smoking or unknown.	Obtained from Register: Birth Register Table: Variable: tupakointitunnus	Smoke=0 if tupakointitunnus=1 Smoke=1 if tupakointitunnus =2-4 Smoke=9 if tupakointitunnus=9 (unknown) or missing.	Obtained from Register: Medical Birth Registry of Norway Variable: royk_beg and royk_avsl	Information on smoking at start and end of pregnancy is obtained from royk_beg and royk_avsl, respectively. Both variables are coded as<br>1 = No<br>2 = Sometimes<br>3 = Daily<br>If royk_beg = 1 AND royk_avsl = 1, smoke is coded as 0<br>If royk_beg = 2 OR royk_beg = 3 OR royk_avsl = 2 OR royk_avsl = 3, smoke is coded as 1<br>Otherwise smoke = 9. Mothers can opt out of having information on smoking recorded. Thus, royk_beg and royk_avsl is missing for a high proportion of births. The proportion with smoke = 9 is 43%.	Obtained from Socialstyrelsen Register: Medicinska födelseregistret Variable: rok1	The variable rok1 pertains to smoking habits at registration with maternal health (usually at 8-12 weeks of pregnancy).<br><br>If the woman was smoking >=1 cigarette/day at registration (rok1 coded 2 or 3), the variable smoke was coded = 1.<br>If the woman was not smoking (rok1 coded 1) the variable smoke was coded = 0.<br>If data was missing the variable smoke was coded = 9 (missing).<br><br>(There is another variable, rok2, which pertains to smoking habits at pregnancy week circa 30-32. This was not included due to very poor data quality 1990-1999, and poor completeness thereafter (Source publication: Graviditeter, förlossningar och nyfödda barn (socialstyrelsen.se) , Statistikdatabaser - Förlossningsstatistik - Val (socialstyrelsen.se))

32

Link to *Contents*

Link to *Contents*

singleton	Obtained from the Danish National Health Data Agency.<br>Register: "CPR-Registeret"<br>Register: "MFR" from 1997 and onwards, "Fødselsregisteret" before 1997<br>Table: "MFR"(from MFR), "levendefødt" (From fødselsregisteret)<br>Variable: "Flerfoldsgraviditet" (MFR), C_PLAC (fødselsregisteret)	MFR<br>Children are categorized as:<br>0="no" if there is an indication of multiple child delivery (diagnosis code) or there is registered another child born by the same mother within 1 day from the child's birthday<br><br>1="yes" if there is no indication of multiple child delivery ("Flerfoldsgraviditet"=missing)<br><br>Fødselsregisteret<br>Children are categorized as:<br>0="no" if C_PLAC>0 or there is registered another child born by the same mother within 1 day from the child's birthday<br><br>1="yes" if C_PLAC=0 and no child born by the same mother within 1 day from the child's birthday	Obtained from Register: Birth Register<br>Table:<br>Variable: sikioita	Children are categorized as:<br>0="no" if sikioita=2 or more<br><br>1="yes" if sikioita=1	Obtained from Register: Medical Birth Registry of Norway<br>Variable: flerfodsel	singleton is coded as 0 if flerfodsel = 1 or if another child is born to the same mother in the same month (N = 13). Otherwise, singleton is coded as 1.	Obtained from Socialstyrelsen<br>Register: Medicinska födelseregistret<br>Variable: bordf2	1="Enkelbörd" was left unaltered (=1 "Yes").<br>2="Flerbörd" was recoded to 0 "No".

Link to *Contents*

child_order	Obtained from the Danish National Health Data Agency. Register: “CPR-Registeret” Register: “MFR” from 1997 and onwards, “Fødselsregisteret” before 1997 Table: “MFR”(from MFR), ”levendefødt” (From fødselsregisteret) Variable: ”paritet” (MFR), V_TIDLLEV, V_TIDLDOD (fødselsregisteret)	The variable from MFR contains information on number of fulfilled pragnancies including stillbirths. Before 1997 the variables V_TIDLLEV(previous live births)+V_TIDLDOD (previous still births) has been added plus 1(current delivery), to simulate the information from MFR.<br><br>Second, a counting method is applied using the registered parity indication for the first registered child and counting onwards for following liveborn children. Preparation is done in 3 steps:<br>1) parity of the first registered child is determined:<br>a) missing information on the first registered child by a mother but with information on the second registered child are recoded with parity of the second child minus 1.<br>b) children with missing information on the first registered child are recoded with parity=1 if the second child is registered as parity=1.<br>2) child order of following children is determined using a counting method	Obtained from Register: Birth Register Table: Variable: aiemmatsynnytykset Variable: kuolleenasynt	Number of the child =“Aiemmatsynnytykset” (previous births) minus “kuolleenasynt” (=stillbirths) plus 1 multiple delivered children are identified, and parity is recoded to the lowest value i.e., twins with 1 older sibling will both be coded with child order=2	Obtained from Register: Medical Birth Registry of Norway Variable: paritet	parity is defined as paritet + 1. The variable paritet is defined by MBRN as the highest value of the variables paritet_mor and paritet_mfr, where paritet_mor is number of previous deliveries as stated by mother and paritet_mor is number of previous deliveries registered by MBRN. Stillbirths are included in paritet.<br><br>Pairs of twins should have the same value of parity and will therefore be assigned the same value of parity (lowest within the set).	Obtained from Socialstyrelsen Register: Medicinska födelseregistret Variable: paritet	The child’s order, based on the number of children previously born by the mother, including this birth. Twins were given the same number, the lowest within the set.

Link to *Contents*

Link to *Contents*

		from the parity of the first registered child plus 1 for each following child<br>3) multiple delivered children are identified, and child order is recoded to the lowest value i.e., twins with 1 older sibling will both be coded with parity=2						

Link to *Contents*

Link to *Contents*

Table: Vaccines

	Denmark		Finland		Norway		Sweden	
Variable	Source and Description	Important notes and data preparation	Source and Description	Important notes and data preparation	Source and Description	Important notes and data preparation	Source and Description	Important notes and data preparation
id	Original name in the Danish data: “pnr”. Pseudonomised unique personal identification number for linkage between registers, created by Statistics Denmark. It is linkable (by Statistics Denmark) to the original personal identification number (CPR number) assigned to all Danish residents and used when reporting to all national registers.	string	Obtained from Register: Vaccination Register Table: Variable: hetu<br><br>THL pseudonymised the original personal identification code (hetu) to unique personal identification number for linkage between registers. THL data management can link the id back to original personal identification code.	Statistics Finland pseudonymised “hetu” with their own id for the remote user system.	Obtained from Register: Norwegian Immunisation Registry (SYSVAK)	string	Created by Statistics Sweden Pseudonomised unique personal identification number for linkage between registers	String
vacdate	Obtained from the state serum institue. Register: “vaccinationsregisteret” Variable: “EffectuationDate”	Date Format (%dD_m_Y)	Obtained from Register: Vaccination Register Table: Variable: Recorddate	Date Format (%dD_m_Y)	Obtained from Register: Norwegian Immunisation Registry (SYSVAK) Variable: konsultasjonsdato	Renamed from konsultasjonsdato Date Format (%dD_m_Y)	Obtained from The Public Health Agency of Sweden (PHAS) Register: The National Vaccination Registry (NVR) Variable: vaccination_date	Date Format (%dD_m_Y)

Link to *Contents*

Link to Contents

vaccine	Obtained from the state serum institue. Register: “vaccinationsregisteret” Variable: “ATCCode”	Categorical (see coding in appendix ”vaccine categorization”) Duplicates were handled as follows, so that only one entry was kept: - same ATCCode: duplicate removed. - same group of vaccines (see appendix vaccine categorization) within 14 days: the entry most likely to have been administered according to the national vaccination schedule at the time was kept. -Hib-vaccine given within 14 days of a multivalent Hib-containing vaccine: was removed - IPV given within 14 days of an multivalent IPV-containing vaccines: was removed	Obtained from Register: Vaccination Register Table: Variable: atc_code	Categorical (see coding in appendix ”vaccine categorization”) Duplicates were handled as follows, so that only one entry was kept: - same ATCCode: duplicate removed. - same group of vaccines (see appendix vaccine categorization) within 14 days: the entry most likely to have been administered according to the national vaccination schedule at the time was kept. -Hib-vaccine given within 14 days of a multivalent Hib-containing vaccine: was removed - IPV given within 14 days of an multivalent IPV-containing vaccines: was removed	Obtained from Register: Norwegian Immunisation Registry (SYSVAK) Variable: vaksinekode	Categorical (see coding in appendix ”vaccine categorization”) Duplicates by same ATCCode are removed. Duplicates by same group of vaccines (see appendix vaccine categorization) within 14 days are cleaned based on information on which vaccine is most likely to have been administered according to the national vaccination schedule and historical changes. Hib given within 14 days of Hib containing vaccines are removed IPV given within 14 days of IPV containing vaccines are removed	Obtained from: PHAS Register: NVR Variable: atc, product_name	Categorical (see coding in appendix ”vaccine categorization”) Duplicates were handled as follows, so that only one entry was kept: - same ATCCode: duplicate removed. - same group of vaccines (see appendix vaccine categorization) within 14 days: the entry most likely to have been administered according to the national vaccination schedule at the time was kept. -Hib-vaccine given within 14 days of a multivalent Hib-containing vaccine: was removed - IPV given within 14 days of an multivalent IPV-containing vaccines: was removed

Link to Contents

Link to Contents

credibility	Variable generated based on data preparation	1=no duplicate<br><br>2= duplicate same vaccine removed<br><br>3=duplicate related vaccine removed (keep vaccine that aligns with vaccination schedule)<br><br>4= duplicate related vaccine removed (none of the vaccines align with vaccination schedule)<br><br>5= duplicate related vaccine removed (vaccines given outside usual vaccination ages within the national immunisation programme)	Variable generated based on data preparation	1=no duplicate<br><br>2= duplicate same vaccine removed<br><br>3=duplicate related vaccine removed (keep vaccine that aligns with vaccination schedule)<br><br>4= duplicate related vaccine removed (none of the vaccines align with vaccination schedule)<br><br>5= duplicate related vaccine removed (vaccines given outside usual vaccination ages within the national immunisation programme)	Variable generated based on data preparation	1=no duplicate<br><br>2= duplicate same vaccine removed<br><br>3=duplicate related vaccine removed (keep vaccine that aligns with vaccination schedule)<br><br>4= duplicate related vaccine removed (none of the vaccines align with vaccination schedule)<br><br>5= duplicate related vaccine removed (vaccines given outside usual vaccination ages within the national immunisation programme)	Variable generated based on data preparation	1=no duplicate<br><br>2= duplicate same vaccine removed<br><br>3=duplicate related vaccine removed (keep vaccine that aligns with vaccination schedule)<br><br>4= duplicate related vaccine removed (none of the vaccines align with vaccination schedule)<br><br>5= duplicate related vaccine removed (vaccines given outside usual vaccination ages within the national immunisation programme)
TB_endemic		9= not relevant		9= not relevant		9=not relevant	Obtained from Statistics Sweden Register: RTB Variable: fodelselandnamn	If the child, mother OR father was born in a country with high or very high incidence of tuberculosis ie. >25 cases per 100,000 inhabitants (as listed in WHO:s Global TB report 2018, link), the child was coded 1=risk group, as this corresponds to eligibility for BCG-vaccination.<br><br>All other children were coded = 0.

Link to Contents

Link to Contents

HepB_endemic		9=not relevant		9=not relevant		9=not relevant	Obtained from Statistics Sweden<br>Register:<br>Table:<br>Variable: fodelselandnamn	If the child, mother OR father was born in a country with an intermediary or high prevalence of hepatitis B in the population (> 2 percent HbsAg-positive), the child was coded 1=risk group. *<br><br>If the child and both parents came from low prevalence countries, the child was coded = 0. (This included all native-born children.)<br><br>If the child came from a country with an unknown prevalence, it was coded as missing.
* Source: Schweitzer A, Horn J, Mikolajczyk RT, Krause G, Ott JJ. Estimations of worldwide prevalence of chronic hepatitis B virus infection: a systematic review of data published between 1965 and 2013. The Lancet. 2015;386(10003):1546-55. DOI:https://doi.org/10.1016/S0140-6736(15)61412-X.								

Link to Contents

Link to Contents

Table: socio_economy

	Denmark		Finland		Norway		Sweden	
Variable	Source and Description	Important notes and data preparation	Source and Description	Important notes and data preparation	Source and Description	Important notes and data preparation	Source and Description	Important notes and data preparation
id	Original name in the Danish data: “pnr”. Pseudonomised unique personal identification number for linkage between registers, created by Statistics Denmark. It is linkable (by Statistics Denmark) to the original personal identification number (CPR number) assigned to all Danish residents and used when reporting to all national registers.	Renamed from “pnr”	Obtained from Register: Population register Table: Variable: hetu THL pseudonymised the original personal identification code (lapsi_hetunnus) to unique personal identification number for linkage between registers. THL data management can link the id back to original personal identification code.	Statistics Finland pseudonymised “hetu” with their own id for the remote user system.		Renamed from “pasientlopenr_pdb2471”	Created by Statistics Sweden Pseudonomised unique personal identification number for linkage between registers Variable: lopnr	Renamed from lopnr

Link to Contents

Link to *Contents*

inc_quin_b	Obtained from Statistics Denmark. Table: “FAIK” (tables for each year) Variable: “FAMAEKVIVADISP_13” (Equated disposable family income)<br><br>Link between each child and family is obtained from Statistics Denmark: Table: BEF (tables for each year) Link variable: FAMILIE_ID (combined with calendar year)	Birth year 2016 and higher do not have information on family income at birth. No children have information from the year they are born, because the statistics are made on the first of January each year. Include information from the year after birth. If no info from that year, the child is coded with unknown (9). Note: quintiles made separately for each calendar year for the children born the year before.	Obtained from Register: Statistics Finland Table: Variable: kturaha_ak_lapsi	Only available in Fiona remote user system. Renamed from “kturaha_ak_lapsi” at the year when child was born. Calculation of quintiles are done separately for each calendar year. E.g. calculating income quintiles for 2008 include all children who use income information from 2008 to assess the income quintile at birth kturaha_ak_lapsi = NA, coded as 9 = "Unknown"	Obtained from Statistics Norway Variable: ies_eu	Based on the variable “ies_eu”, defined as total after-tax income for the household per consumption unit calculated according to the EU scale. Total after-tax income is calculated as the sum of the household’s wages and salaries, income from self-employment, property income and transfers received minus total assessed taxes and negative transfers. Each income year includes all persons residing in Norway and resident in a private household as of 31st December of the income year. Household income in year of birth is used to define inc_quin_b. Income quintiles are made separately for each birth cohort. Available for children born 2004–2018.	Obtained from Statistics Sweden Register: Longitudinell integrationsdatabas för Sjukförsäkrings- och Arbetsmarknadsstudier (LISA) Variable: DispInkFam	In Sweden, disposable income is defined as the sum of all household members’ all forms of income (including wages, capital gains, and different forms of financial support/social assistance) minus taxes and other negative transfers (Statistikskolan: Att jämföra inkomster för hushåll (scb.se)). The information *primarily* came from the information registered for the household of the mother in the year of birth of the child. If this was missing, the information was instead taken from the father. Thus, the child was primarily assumed to be part of the mother’s household, and secondly of the father’s. Income quintiles was then calculated based on all children in each birth cohort.

Link to *Contents*

Link to *Contents*

inc_quin_10y	Obtained from Statistics Denmark.<br>Table: "FAIK" (tables for each year)<br>Variable: "FAMAEKVIVADISP_13" (Equated disposable family income)<br><br>Link between each child and family is obtained from Statistics Denmark:<br>Table: BEF(tables for each year)<br>Link variable: FAMILIE_ID (combined with calendar year)	Birth year 2007 and higher do not have info on family income at ten years.<br>If no info from the year the child turn 10 years the variable is coded with unknown (9).<br>Note: quintiles made separately for each calendar year for the children turning 10 years that year.	Obtained from Register: Statistics Finland<br><br>Table:<br>Variable: kturaha_ak_lapsi	Only available in Fiona remote user system. Renamed from "kturaha_ak_lapsi" at the year when child was 10 years old. Calculation of quintiles are done separately for each calendar year. E.g. calculating income quintiles for 2008 include all children who use income information from 2008 to assess the income quintile at birth kturaha_ak_lapsi = NA, coded as 9 = "Unknown"	Obtained from Statistics Norway<br>Variable: ies_eu	Based on the variable "ies_eu", see definition above. Household income in the year of the child's 10th birthday is used to define inc_quin_10y. Income quintiles are made separately for each birth cohort. Available for children born 1994–2008.	As above.	As above, but from the year the child turned 10 years old.
inc_quin_m_b					Obtained from Statistics Norway<br>Variable: wies	Based on the variable "wies", defined as a person's after-tax income. After-tax income is calculated as the sum of wages and salaries, income from self-employment, property income and transfers received minus total assessed taxes and negative transfers. The mother's income in the child's year of birth is used to define inc_quin_m_b. Income quintiles are made separately for each birth cohort. Available for children born 1993–2018.	Obtained from Statistics Sweden<br>Register: Longitudinell integrationsdatabas för Sjukförsäkrings- och Arbetsmarknadsstudier (LISA)<br>Variable: DispInk	Information about disposable income of the mother in the year of birth of the child.<br><br>Income quintiles was then calculated based on all children in each birth cohort.<br><br>See also above.

Link to *Contents*

Link to *Contents*

inc_quin_m_10y					Obtained from Statistics Norway Variable: wies	Based on the variable "wies", see definition above. The mother's income in the year of the child's 10th birthday is used to define inc_quin_m_10y. Income quintiles are made separately for each birth cohort. Available for children born 1990–2008.	As above.	As above, but from the year the child turned 10 years old.
n_children_b	Obtained from Statistics Denmark.<br>Table: "FAM" (tables for each year)<br>Variables: Sumarized from the variables ANTB00-ANTB17 (number of children in the family age 0, 1, 2…,17)<br><br>Link between each child and family is obtained from Statistics Denmark:<br>Table: BEF(tables for each year)<br>Link variable: FAMILIE_ID (combined with calendar year)	Birth year 2018 do not have info on number of children at birth. No children have information from the year they are born, because the statistics made on the first of January each year. Include information from the year after birth. If no info from that year the child is code with unknown (99).<br>Some children end-up with a count of 0 children, as this is not a legal value they are recoded to 99. Based on the values on family_type, it is judged that the children with a count of 0, are children who are registered as the main person in a family and therefore are not counted as a child although they are children.	Obtained from Register: Statistics Finland<br><br>Table:<br>Variable: lkm_lapsi	Only available in Fiona remote user system. Renamed from "lkm_lapsi" at the year when child was born.	Obtained from Statistics Norway Variable: barn_i_regstat_famnr	Based on variable "barn_i_regstat_famnr", number of children in the family. Persons are considered children if they are below 18 years and registered as resident in the family of at least one parent. A family is defined as persons resident in the same dwelling and related to each other as spouse, registered partner, cohabitant, and/or parent and child (regardless of the child's age). At most, a family may consist of two subsequent generations and one couple only. The variable includes residents of Norway as of January 1 each year. We have therefore used number of children in the year after the child's year of birth. Individuals registered with 0 number of children in their family have been recoded to 1. Available for children born 2004–2018.	Obtained from Statistics Sweden Register: LISA Variable: Barn0_3, Barn4_6, Barn7_10, Barn11_15, Barn16_17	Created as the sum of children in variables Barn0_3, Barn4_6, Barn7_10, Barn11_15 and Barn16_17. The sum denotes the number of children living in the household on 31 Dec in the year of birth of the child. The child itself is part of the count.<br><br>The information *primarily* came from the information registered for the mother in the year of birth of the child. If this was missing, the information was instead taken from the father.

Link to Contents

n_children_10y	Obtained from Statistics Denmark.<br>Table: "FAM" (tables for each year)<br>Variables: Sumarized from the variables ANTB00-ANTB17 (number of children in the family age 0, 1, 2…,17 years)<br><br>Link between each child and family is obtained from Statistics Denmark:<br>Table: BEF(tables for each year)<br>Link variable: FAMILIE_ID (combined with calendar year)	Birth year 2009 and higher do not have info on number of children at 10 years.<br>If no info from the year the child turn 10 years the variable is coded with unknown (99).<br>Some children end-up with a count of 0 children, as this is not a legal value they are recoded to 99. Based on the values on family_type, it is judged that the children with a count of 0, are children who are registered as the main person in a family and therefore are not counted as a child although they are children.	Obtained from Register: Statistics Finland<br><br>Table:<br>Variable: lkm_lapsi	Only available in Fiona remote user system. Renamed from "lkm_lapsi" at the year when child was 10 years old.	Obtained from Statistics Norway<br>Variable: barn_i_regstat_famnr	Based on variable "barn_i_regstat_famnr", see above. The variable includes residents of Norway as of January 1 each year. We have therefore used number of children in the year after the year of the child's 10th birthday. Individuals registered with 0 number of children in their family have been recoded to 1. Available for children born 1994–2009.	As above.	As above, but from the year the child turned 10 years old.

Link to Contents

Link to *Contents*

single_parent_b	Obtained from Statistics Denmark. Table: "FAM" (tables for each year) Variable: FAMILIE_TYPE<br><br>Link between each child and family is obtained from Statistics Denmark: Table: BEF(tables for each year) Link variable: FAMILIE_ID (combined with calendar year)	Birth year 2018 do not have info on single parenthood at birth. No children have information from the year they are born, because the statistics made on the first of January each year. Include information from the year after birth. If no info from that year the child is code with unknown (9).<br>I also set children who originally were coded with 0 children on n_children_b as unknown (9) because it is judged that these are children registered as the main person in the family (no adults in the family?).	Obtained from Register: Statistics Finland<br><br>Table:<br>Variable: pety_lapsi	Only available in Fiona remote user system. Calculated from "pety_lapsi" at the year when child was born.<br>If pety_lapsi is 2 (married couple and children) or 5-6 (couple with children) -> single parent =0 (no)<br>If pety_lapsi is 3 or 4 (mother or father with children) -> single parent = 1 (yes).<br>If pety_lapsi is unknown -> single parent = 9	Obtained from Statistics Norway Variable: regstat_famtyp	Based on the variable "regstat_famtyp", a detailed classification of family type, where family is defined as described above. The variable includes residents of Norway as of January 1 each year. We have therefore used the value of family type in the year after a child's year of birth to define single_parent_b. If the registered family type is either "married couple with small children (youngest child aged 0-5 years)" or "cohabitants with small children (youngest child aged 0-5 years)", single_parent_b is coded as 0. If the registered family type is either "mother with small children (youngest child aged 0-5 years)" or "father with small children (youngest child aged 0-5 years)", single_parent_b is coded as 1. Otherwise (family type is any other category or missing), single_parent_b is coded as 9. Available for children born 2004–2018.	Obtained from Statistics Sweden Register: LISA Variable: FamTypF	The information came from the information registered for the mother in the year of birth of the child.<br><br>Codes FamTypF=41, 42 classifies the mother as a single parent, and 50 denotes Other singles. These codes were included when coding single_parent_b=1 (yes). If FamTypF was missing, single_parent_b was coded as 9 (missing). All other FamTypF-codes were recoded as 0 (no).

Link to *Contents*

Link to *Contents*

single_parent_10y	Obtained from Statistics Denmark.<br>Table: "FAM" (tables for each year)<br>Variable: FAMILIE_TYPE<br><br>Link between each child and family is obtained from Statistics Denmark:<br>Table: BEF(tables for each year)<br>Link variable: FAMILIE_ID (combined with calendar year)	Birth year 2018 do not have info on single parenthood at birth.<br>If no info from the year the child turn 10 years the variable is coded with unknown (9).<br>set children who originally were coded with 0 children on n_children_10y as unknown (9) because it is judged that these are children registered as the main person in the family (no adults in the family?).	Obtained from Register: Statistics Finland<br><br>Table:<br>Variable: pety_lapsi	Only available in Fiona remote user system.<br>Calculated from "pety_lapsi" at the year when child was 10 years old.<br>If pety_lapsi is 2 (married couple and children) or 5-6 (couple with children) -> single parent =0 (no)<br>If pety_lapsi is 3 or 4 (mother or father with children) -> single parent = 1 (yes).<br>If pety_lapsi is empty -> single parent = 9 (unknown)	Obtained from Statistics Norway<br>Variable: regstat_famtyp	Based on the variable "regstat_famtyp", see above. The variable includes residents of Norway as of January 1 each year. We have therefore used the value of family type in the year after the year of a child's 10th birthday to define single_parent_10y. If the registered family type is either "married couple with small children (youngest child aged 0-5 years)", "married couple with older children (youngest child aged 6-17 years) "cohabitants with small children (youngest child aged 0-5 years)", or "cohabitants with older children (youngest child aged 6-17 years)", single_parent_10y is coded as 0. If the registered family type is either "mother with small children (youngest child aged 0-5 years)", "mother with older children (youngest child aged 6-17 years)", "father with small children (youngest child aged 0-5 years)", or "father with older children (youngest child aged 6-17 years)", single_parent_10y is coded as 1. Otherwise (family type is any other category or missing), single_parent_10y is coded as 9. Available for children born 1994–2009.	As above.	As above, but from the year the child turned 10 years old.

Link to *Contents*

Link to Contents

m_education_b	Obtained from Statistics Denmark.<br>Table: “UDDF”<br>Variable: ”hfaudd” ”hfaudd” i linked with format from statistics Denmark grouping the Danish education classification into ISCED 2011, based on which maternal education is grouped.<br><br>Link to mother is available from the dataset “population1” (originally obtained from the CPR register).	Use the highest obtained education for the mother on the date of birth of the child. There is no information on this for children born 2017 or later. Statistics Denmark had a format available for transforming national Danish education codes into ISCED.	Obtained from Register: Statistics Finland<br><br>Table: Variable:ututku_aiti and koulutusaste_taso_1 and birthday of child obtained from population1	Education is classified by ISCED-11, although the classes 0-2 are not available for us. In Finland, we have compulsory education during which the ISCED level 2 is achieved and thus we classified education: NA= 1 low education level<br>3-4 = 2 medium education level<br>5-8 = 3 high education level<br>9 = no information of the mother’s Education at child’s birth year.	Obtained from Statistics Norway Variable: bu_niva_YYYY	Based on the variables “bu_niva_YYYY”. The variables contain information on highest level of education as of October 1 of the year YYYY. Mother’s level of education from the child’s year of birth was used to define m_education_b. Education is classified according to The Norwegian Standard Classification of Education (NUS). If the NUS-level is 0 (corresponding to ISCED2011 levels 01, 02), 1 or 2 (corresponding to ISCED2011 level 1 and 2, respectively), m_education is coded as 1. If the NUS level is 3 or 4 (corresponding to ISCED2011 = 3), m_education is coded as 2. If the NUS-level is 6, 7 or 8 (corresponding to ISCED2011 level 6, 7, and 8, respectively), m_education is coded as 3 (https://www.ssb.no/utdanning/artikler-og-publikasjoner/_attachment/240569?_ts=150ebb996e0, page 25). NUS-level = 5 is defined as tertiary vocational educational level not approved as higher education. Tertiary education with duration less than 2 years corresponds to ISCED2011 level 4. In this case, m_educaion_b should be coded as 2. Tertiary education with duration of 2 years corresponds to ISCED2011 level 5, and m_education_b should be coded as 3. However, we do not have information on type or duration of the tertiary	Obtained from Statistics Sweden Register: LISA Variable: Sun2000niva	The variable denotes the highest level of education achieved during the spring semester in the year the child was born. That means, that if the mother achieved a higher level of education mid-year, it will only be visible in the register for the following year.<br>Level of education was recoded from Sun2000 to ISCED by a translational key available from Statistics Sweden: Svensk utbildningsnomenklatur (SUN) (scb.se) (retrieved 2021-08-20).

Link to Contents

Link to Contents

						education. In 2016, 83.6% of women graduating from tertiary vocational education, had finished an education with duration of 2 years, while only 16.4% had finished an education with duration less than 2 years(https://www.ssb.no/en/statbank/table/11635). Therefore, m_education_b was coded as 2 if the NUS-level was 5. Available for children born 1990–2018.		

Link to Contents

Link to Contents

m_education_10y	Obtained from Statistics Denmark.<br>Table: "UDDF"<br>Variable: "hfaudd" "hfaudd" i linked with format from Satistics Denmark grouping the Danish education classification into ISCED 2011, based on which maternal education is grouped.<br><br>Link to mother is available from the dataset "population1" (originally obtained from the CPR register).	Use the highest obtained education for the mother on the date of the child turns 10 years.<br>There is no information on this for children born 2007 or later.	Obtained from Register: Statistics Finland<br><br>Table:<br>Variable: ututku_aiti and koulutusaste_taso_1 and birthday of child obtained from population1	Education is classified by ISCED-11. although the classes 0-2 are not available for us. In Finland, we have compulsory education during which the ISCED level 2 is achieved and thus we classified education: NA= 1 low education level<br>3-4 = 2 medium education level<br>5-8 = 3 high education level<br>9 = no information of the mother's Education when child is 10 years old.<br>If education was lower than m_education_b it was coded to be the same as at birth, also if education was unknown when child was ten, but it was known when child was born, the m_education_b was used as m_education_10y	Obtained from Statistics Norway<br>Variable: bu_niva_YYYY	Mother's highest level of education as of October 1 in the year of child's 10-year birthday was used to define m_education_b. For definitions and coding, see above. Available for children born 1990–2009.	As above.	As above, but from the year the child turned 10 years old.

Link to Contents

Link to Contents

Appendix: Vaccine categorization: presentation of ATC codes for vaccines identified in the vaccination registries in each of the Nordic countries and categorisation hereof into common vaccine categories "vaccine". The vaccines are further grouped by "type" i.e. vaccines against a similar set of diseases. NB in Sweden only vaccines that are included in the national immunisation programme is registered in the vaccination register.

		DENMARK	Finland	Norway	Sweden
Vaccine	Type	ATC Code			
1= "DTaP-IPV-Hib"	1	J07CA06	J07CA06	J07CA06	J07CA06
2= "DTaP-IPV-Hib-HepB"		J07CA09		J07CA09	J07CA09
3= "DTaP-IPV"		J07CA02<br>J07CA02	J07CA02<br>J07CA02	J07CA02<br>J07CA02	J07CA02
4= "DT-Pol"		J07CA01		J07CA01	
5= "DT-HepB"		J07CA07			
6= "DTwP-HepB"		J07CA05			
7= "DTwP-Hib-HepB"		J07CA11			
8= "DTaP-IPV-HepB"		J07CA12			J07CA12
10= "DTaP"		J07AJ52	J07AJ52	J07AJ52	J07AJ52
11= "DTwP"		J07AJ51		J07AJ51	
12= "DT"		J07AM51	J07AM51	J07AM51<br>J07AM52	J07AM51
13= "D"		J07AF01		J07AF01	J07AF01
15= "T"		J07AM01		J07AM01	J07AM01
20= "PCV"	2	J07AL02	J07AL02	J07AL52<br>J07AL02	J07AL52<br>J07AL02
21="PPV"		J07AL01	J07AL01	J07AL01	J07AL01

Link to Contents

Link to Contents

25="HepA"	3	J07BC02	J07BC02	J07BC02	
26= "HepAB"		J07BC20	J07BC20	J07BC20	
27= "HepB"		J07BC01	J07BC01	J07BC01	
28= "HepA-Thyphoid"		J07CA10		J07CA10	
30= "HPV4"	4	J07BM01		J07BM01	J07BM01
31="HPV2"		J07BM02	J07BM02	J07BM02	J07BM02
32="HPV9"		J07BM03	J07BM03	J07BM03	J07BM03
35= "Hib"	5	J07AG01	J07AG01	J07AG01	J07AG01
36= "Hib-MenC"		J07AG53			
37= "Hib-Pol"				J07CA04	
38= "Hib-HepB"		J07CA08			
40= "Influenza (non-live)"	6	J07BB01<br>J07BB02	J07BB02	J07BB01<br>J07BB02	
41= "Influenza (live)"		J07BB03	J07BB03	J07BB03	
45= "wP"	7	J07AJ01		J07AJ01	
46= "aP"		J07AJ02		J07AJ02	
50= "MMR"	8	J07BD52	J07BD52	J07BD52	J07BD52
51= "MMR-Varicella"		J07BD54	J07BD54		
52= "Measles"		J07BD01		J07BD01	
53= "Measles-Mumps"		J07BD51		J07BD51	
54= "Measles-Rubella"		J07BD53		J07BD53	
55= "Rubella"		J07BJ01		J07BJ01	
56= "Mumps"		J07BE01		J07BE01	
60= "OPV"	9	J07BF01		J07BF04<br>J07BF01	

Link to Contents

Link to Contents

		J07BF02		J07BF02	
61= "IPV"		J07BF03	J07BF03	J07BF03	J07BF03
65= "Rota"		J07BH01<br>J07BH02	J07BH02	J07BH01<br>J07BH02	
67= "BCG"		J07AN01	J07AN01	J07AN01	
70="Varicella"		J07BK01<br>J07BK02	J07BK01	J07BK03<br>J07BK02<br>J07BK01	
71= "yellow fever"		J07BL01	J07BL01	J07BL01	
72= "Japanease Encephalitis"		J07BA02	J07BA02	J07BA02	
73= "Tick borne Encephalitis"		J07BA01	J07BA01	J07BA01	
74 = "Cholera"		J07AE51<br>J07AE02<br>J07AE01	J07AE01	J07AE01	
75= "Meningococcal vaccine"		J07AH08<br>J07AH09<br>J07AH03<br>J07AH04<br>J07AH06<br>J07AH05<br>J07AH02<br>J07AH01	J07AH08<br>J07AH09	J07AH07<br>J07AH08<br>J07AH09<br>J07AH03<br>J07AH04<br>J07AH06<br>J07AH01	
76= "Typhus"		J07AP01<br>J07AP10	J07AP01	J07AP01<br>J07AP02<br>J07AP	

Link to Contents

Link to *Contents*

		J07AP03		J07AP03	
77= "Rabies"		J07BG01		J07BG01	
78= "Smallpox"				J07B01	
		J07BX01			
79="Anthrax"				J07AC01	
80="covid-19 vaccine"			J07BX03	J07BX03	
99="other vaccines"		ATC code missing	ATC code missing	ATC code missing	ATC code missing

Link to *Contents*
