# Supplementary material for: Cohort Profile: Childhood morbidity and potential non-specific effects of the childhood vaccination programmes in the Nordic countries (NONSEnse): register-based cohort of children born 1990–2017/2018
Source: BMJ Open. 2023 Feb 10;13(2):e065984. doi: 10.1136/bmjopen-2022-065984 (PMC9923270; doi:10.1136/bmjopen-2022-065984)
Supplement: Supplementary data [file bmjopen-2022-065984supp003.pdf]

Online supplementary files

sTable 1: Vaccination coverage[1] at 2 years of age according to year of birth among children born in the respective countries

sTable2: Human papilloma virus vaccination coverage[1] before 14 years of age of vaccination among girls[2] born in the respective countries

sTable 3: Socio-economic factors at 10 years of age

sTable 4: ATC codes obtained for the study population in each country within NONSEnse

sTable 1: Vaccination coverage[1] at 2 years of age according to year of birth among children born in the respective countries

**Denmark**						
Year of birth	Eligible[2] Children	DTP1 % (95% CI)	DTP2 % (95% CI)	DTP3 % (95% CI)	MMR1 % (95% CI)	
1997	66,406	98.9	96.0	82.2	81.5	
1998	64,936	99.0	97.0	85.0	83.6	
1999	64,996	99.0	97.0	84.9	84.5	
2000	65,811	99.0	97.3	86.2	85.9	
2001	64,207	99.1	97.4	86.4	85.5	
2002	62,948	99.1	97.0	84.1	84.9	
2003	63,462	98.9	96.6	82.5	85.2	
2004	63,339	98.9	96.5	82.7	86.8	
2005	62,912	98.9	96.0	80.1	84.8	
2006	63,769	99.0	95.8	78.7	84.4	
2007	63,006	99.0	96.4	80.9	82.6	
2008	63,892	99.2	97.2	83.9	83.7	
2009	61,676	99.3	97.6	86.6	86.2	
2010	62,200	99.2	97.9	88.8	87.3	
2011	57,892	99.2	98.0	89.7	86.7	
2012	56,842	99.2	98.0	90.0	86.4	
2013	54,881	98.9	97.6	88.5	88.3	
2014	55,753	98.8	97.5	87.6	88.4	
2015	57,100	98.9	98.0	93.2	90.3	
2016	23,103	99.0	98.3	94.7	90.7	
**Finland**						
Year of birth	Eligible[2] Children	DTP1 % (95% CI)	DTP2 % (95% CI)	DTP3 % (95% CI)	MMR1 % (95% CI)	Rota virus vaccine % (95% CI)
2009	59,934	94.0	92.7	89.4	87.8	63.8
2010	60,560	96.7	94.2	90.8	91.6	90.7
2011	59,645	97.0	95.1	90.4	92.3	90.8
2012	59,309	95.7	94.5	90.3	91.9	91.2
2013	58,249	97.5	95.7	91.8	93.3	90.4

2014	57,693	97.6	96.5	89.3	92.6	91.6
2015	55,569	98.0	96.6	88.1	93.4	92.1
**Norway**						
Year of birth	Eligible[2] Children	DTP1 % (95% CI)	DTP2 % (95% CI)	DTP3 % (95% CI)	MMR1 % (95% CI)	Rota virus vaccine % (95% CI)
1995	59964	98.5	97.9	95.8	94.7	
1996	60652	98.2	97.4	95.4	94.3	
1997	59431	98.5	98.0	96.2	94.4	
1998	57999	98.7	98.2	96.4	94.1	
1999	58975	98.8	98.3	96.4	94.1	
2000	58907	98.6	98.0	96.1	89.2	
2001	56405	98.7	98.3	96.1	89.3	
2002	55232	98.8	98.5	96.7	92.7	
2003	56301	99.0	98.6	96.8	94.2	
2004	56734	99.1	98.8	97.5	94.7	
2005	56531	99.2	99.0	97.6	94.4	
2006	58316	99.1	98.8	97.3	94.2	
2007	58199	99.0	98.6	96.7	94.0	
2008	60284	99.0	98.5	96.6	93.8	
2009	61465	98.9	98.6	97.4	94.4	
2010	61080	98.9	98.5	97.3	95.0	
2011	59855	98.8	98.4	97.2	94.8	
2012	59937	98.6	98.2	96.6	95.0	
2013	58745	98.6	98.0	96.5	95.5	
2014	58839	98.7	98.3	96.6	95.9	
2015	58954	98.6	98.1	96.6	95.7	94.1
2016	58975	98.5	97.8	96.3	95.9	94.8
**Sweden**						
Year of birth	Eligible[2] Children	DTP1 N (%)	DTP2 N (%)	DTP3 N (%)	MMR1 (%)	
2013	113,457	97.6	95.1	83.3	89.1	
2014	114,639	98.0	95.9	86.1	90.7	
2015	114,542	98.1	96.3	87.8	91.8	

Abbreviations: DTP1: First dose of Diphtheria, Tetanus, and acellular Pertussis containing vaccine; DTP2: Second dose of Diphtheria, Tetanus, and acellular Pertussis containing vaccine; DTP3: Third dose of Diphtheria, Tetanus, and acellular Pertussis containing vaccine; MMR: Measles-Mumps-Rubella vaccine; Rota: Rota virus vaccine.

[1]The coverage reflects the number of registered vaccines and may thus underestimate the actual vaccination coverage in the countries. [2] Including children born in the country from birth cohorts where vaccines administered between 0-2 years of age are registered in the vaccination registers (data availability period).

sTable 2: Human papilloma virus vaccination coverage[1] before 14 years of age of vaccination among girls[2] born in the respective countries

	Denmark			Finland			Norway			Sweden		
Year of birth	Eligible [3] children	HPV1 vaccinated N (%)	HPV2 vaccinated N (%)	Eligible[3] children	HPV1 vaccinated N (%)	HPV2 vaccinated N (%)	Eligible [3] children	HPV1 vaccinated N (%)	HPV2 vaccinated N (%)	Eligible [3] children	HPV1 vaccinated N (%)	HPV2 vaccinated N (%)
1998	34,392	85.7	81.2				30,914	76.5 (76.0, 77.0)	75.8			
1999	34,484	86.8	82.4				31,391	78.5	77.7			
2000	34,881	86.5	82.3				31,490	80.2	79.5 (79.0, 79.9)			
2001	34,030	81.4	74.5				30,546	82.7	82.0			
2002	33,241	73.6	57.6	4053	71.1	69.3	30,213	84.5	83.7			
2003	33,762	52.3	36.1	27,310	69.8	67.2	30,925	84.8	84.0	53,623	77.9	72.4
2004	13,184	58.4	43.0				31,208	86.8	85.8			

Abbreviations: HPV1: First dose of Human papilloma virus vaccine; HPV2: Second dose of Human papilloma virus vaccine

[1]The coverage reflects the number of registered vaccines and may thus underestimate the actual vaccination coverage. [2] Including girls from birth cohorts where HPV vaccination has been offered from 1 year before age of recommended vaccination until 14 years of age and where vaccinations were registered in the vaccination registers. The years with available data is defined based on introduction of HPV vaccinations into the National immunization programme or introduction of vaccination register whichever comes last until last date with available data from both the population register and vaccination register.

sTable 3: Socio-economic factors at 10 years of age

	Denmark		Finland		Norway		Sweden	
	N	(%)	N	(%)	N	(%)	N	(%)
Children present in country at birth from 2004-2015	793,471		687,721		726,257		1,205,112	
Birth cohorts included	1994-2005		1994-2005		1994-2005		1994-2005	
Income quintile at 10 years of age								
First (lowest)	147,098	18.5%	135,946	19.8%	141,762	19.5%	219,673	18.2%
Second	149,579	18.9%	136,334	19.8%	146,247	20.1%	241,503	20.0%
Third	149,865	18.9%	136,384	19.8%	146,604	20.2%	245,097	20.3%
Fourth	149,310	18.8%	136,259	19.8%	146,471	20.2%	245,138	20.3%
Fifth	146,712	18.5%	135,464	19.7%	144,939	20.0%	241,452	20.0%
Unknown	50,907	6.4%	7334	1.1%	234	0.0%	12,249	1.0%
Number of children in the household the year the child turns 10 years of age								
1	103,585	13.1%	96,861	14.1%	119,295	16.4%	162,968	13.5%
2	405,367	51.1%	293,068	42.6%	336,480	46.3%	587,332	48.7%
3	213,413	26.9%	184,925	26.9%	208,045	28.6%	292,516	24.3%
>3	61,674	7.8%	103,561	15.1%	62,203	8.6%	105,595	8.8%
Unknown	9432	1.2%	9306	1.4%	234	0.0%	12,249	1.0%
Single parenthood in the years the child turns 10 years of age								
Yes	151,471	19.1%	124,986	18.2%	131,761	18.1%	268,484	22.3%
No	632,568	79.7%	553,429	80.5%	587,793	80.9%	924,379	76.7%
Unknown	9432	1.2%	9306	1.4%	6703	0.9%	12,249	1.0%
Highest attained educational level[1] of the mother on the date the child turns 10 years of age								
Low education	135,466	17.1%	75,462	11.0%	138,351	19.0%	193,551	16.1%
Medium education	340,574	42.9%	281,479	40.9%	270,114	37.2%	515,407	42.8%
High education	303,384	38.2%	329,885	48.0%	305,368	42.0%	394,220	32.7%
Unknown	14,047	1.8%	895	0.1%	12,424	1.7%	101,934	8.5%

[1] Highest attained education was categorized based on the International Standard Classification of Education (ISCED) 2011 using the main groups (1).

sTable 4: ATC codes obtained for the study population in each country within NONSEnse

ATC-Group	Denmark	Finland	Norway	Sweden
D	D	D07, D11AH	D	D02AF, D05<br>D07, D11<br>D01, D06, D08
J	J	J	J	J01-J06<br>J07
R	R	R01, R03, R06	R	R01, R03, R06
S	S	S01G, S03	S	S01-S03
V	V01	V01[1]	V01	V01

[1] Data on redeemed prescriptions with ATC=V01 is only available from the Finnish Benefits Registry, which holds information only for reimbursable redeemed prescriptions.

References

1. UNESCO Institute for Statistics. International standard classification of education: ISCED 2011. 2012.
