# Supplementary material for: Cohort Profile: Childhood morbidity and potential non-specific effects of the childhood vaccination programmes in the Nordic countries (NONSEnse): register-based cohort of children born 1990–2017/2018
Source: BMJ Open. 2023 Feb 10;13(2):e065984. doi: 10.1136/bmjopen-2022-065984 (PMC9923270; doi:10.1136/bmjopen-2022-065984)
Supplement: Author's manuscript [file bmjopen-2022-065984.draft_revisions.pdf]

BMJ Open is committed to open peer review. As part of this commitment we make the peer review history of every article we publish publicly available.

When an article is published we post the peer reviewers' comments and the authors' responses online. We also post the versions of the paper that were used during peer review. These are the versions that the peer review comments apply to.

The versions of the paper that follow are the versions that were submitted during the peer review process. They are not the versions of record or the final published versions. They should not be cited or distributed as the published version of this manuscript.

BMJ Open is an open access journal and the full, final, typeset and author-corrected version of record of the manuscript is available on our site with no access controls, subscription charges or pay-per-view fees (http://bmjopen.bmj.com).

If you have any questions on BMJ Open's open peer review process please email info.bmjopen@bmj.com

BMJ Open

Cohort Profile: Childhood morbidity and potential non-specific effects of the childhood vaccination programmes in the Nordic countries (NONSEnse): Register-based cohort of children born 1990-2017/2018.

Journal:	*BMJ Open*
Manuscript ID	bmjopen-2022-065984
Article Type:	Cohort profile
Date Submitted by the Author:	01-Jul-2022

Complete List of Authors:	Gehrt, Lise; University of Southern Denmark, Research Unit OPEN<br>Laake, Ida; Norwegian Institute of Public Health, Division of Infection Control and Environmental Health<br>Englund, Hélène; Public Health Agency of Sweden<br>Nieminen, Heta; Finnish Institute for Health and Welfare, Department of Public Health and Welfare<br>Feiring, Berit; Norwegian Institute of Public Health, Division of Infection Control and Environmental Health<br>Lahdenkari, Mika; Finnish Institute for Health and Welfare, Department of Information Services<br>Palmu, Arto A.; Finnish Institute for Health and Welfare, Department of Public Health and Welfare,<br>Trogstad, Lill; Norwegian Institute of Public Health, Division of Infection Control and Environmental Health<br>Benn, Christine Stabell ; University of Southern Denmark, Bandim Health Project, Research unit Open, Department of Clinical Research, ; University of Southern Denmark, Danish Institute for Advanced Study<br>Sørup, Signe; Aarhus University Hospital, Department of Clinical Epidemiology

Keywords:	Epidemiology < INFECTIOUS DISEASES, Paediatric infectious disease & immunisation < PAEDIATRICS, PUBLIC HEALTH

SCHOLARONE™
Manuscripts

I, the Submitting Author has the right to grant and does grant on behalf of all authors of the Work (as defined in the below author licence), an exclusive licence and/or a non-exclusive licence for contributions from authors who are: i) UK Crown employees; ii) where BMJ has agreed a CC-BY licence shall apply, and/or iii) in accordance with the terms applicable for US Federal Government officers or employees acting as part of their official duties; on a worldwide, perpetual, irrevocable, royalty-free basis to BMJ Publishing Group Ltd ("BMJ") its licensees and where the relevant Journal is co-owned by BMJ to the co-owners of the Journal, to publish the Work in this journal and any other BMJ products and to exploit all rights, as set out in our licence.

The Submitting Author accepts and understands that any supply made under these terms is made by BMJ to the Submitting Author unless you are acting as an employee on behalf of your employer or a postgraduate student of an affiliated institution which is paying any applicable article publishing charge ("APC") for Open Access articles. Where the Submitting Author wishes to make the Work available on an Open Access basis (and intends to pay the relevant APC), the terms of reuse of such Open Access shall be governed by a Creative Commons licence – details of these licences and which Creative Commons licence will apply to this Work are set out in our licence referred to above.

Other than as permitted in any relevant BMJ Author's Self Archiving Policies, I confirm this Work has not been accepted for publication elsewhere, is not being considered for publication elsewhere and does not duplicate material already published. I confirm all authors consent to publication of this Work and authorise the granting of this licence.

For peer review only

Title

Cohort Profile: Childhood morbidity and potential non-specific effects of the childhood vaccination programmes in the Nordic countries (NONSEnse): Register-based cohort of children born 1990-2017/2018.

Author list

Lise Gehrt[1], MSc; Ida Laake, PhD[2]; Hélène Englund, MSc[3]; Heta Nieminen, MD[4]; Berit Feiring, PhD[2]; Mika Lahdenkari[5]; Arto A. Palmu[4]; Lill Trogstad[2]; Christine Stabell Benn[1,6]; Signe Sørup, PhD[7]

[1]Bandim Health Project, Research unit Open, Department of Clinical Research, University of Southern Denmark, Odense C, Denmark
[2]Division of Infection Control, Norwegian Institute of Public Health Oslo, Norway
[3]Public Health Agency of Sweden, Solna, Sweden
[4]Department of Public Health and Welfare, Finnish Institute for Health and Welfare, Tampere, Finland
[5] Department of Information Services, Finnish Institute for Health and Welfare, Helsinki, Finland
[6]Danish Institute for Advanced Study, University of Southern Denmark, Odense C, Denmark
[7]Department of Clinical Epidemiology, Aarhus University Hospital, Aarhus, Denmark

Correspondence

Lise Gehrt, Bandim Health Project, Research unit Open, Department of Clinical Research, University of Southern Denmark, Odense C, Denmark
Telephone: +45 40 54 40 38.

E-mail: lgehrt@health.sdu.dk

For peer review only

ABSTRACT

Purpose: The aim of the NONSEnse project is to investigate the non-specific effects of vaccines and immunisation programmes on the overall health of children by using information from the extensive nationwide registers on health and sociodemographic factors in Denmark, Finland, Norway, and Sweden.

Participants: The cohort covers 9,072,420 children aged 0-17 years, born 1990-2017/2018, and living in Denmark, Finland, Norway, or Sweden. All countries utilise a unique identification number for its permanent residents, which makes it possible to link individual-level information from different registers.

Findings to date: Data collection and harmonisation according to a Common Data Model was completed in March 2022. As a prerequisite for comparing the effects of childhood vaccinations on the overall health of children across the Nordic countries, we have identified indicators measuring similar levels of infectious disease morbidity across these settings. We have also conducted an interrupted-time series analysis of the association between the second dose of measles-mumps-rubella (MMR) vaccination and the rate of infectious disease hospitalisations due to infections not targeted by the MMR vaccine.

Future plans: We are currently performing several studies of the effects on non-targeted infectious disease morbidity across the countries following vaccination against MMR, diphtheria, tetanus, pertussis, human papilloma virus, rotavirus, and influenza. Multiple studies are planned within the next years using different study designs to facilitate triangulation of results and enhance causal inference.

Registration: No clinical trials will be conducted within the NONSEnse project.

For peer review only - http://bmjopen.bmj.com/site/about/guidelines.xhtml

STRENGHTS AND LIMITATIONS

Strengths:

- The cohort covers the entire Nordic child population, which minimizes selection bias.
- Real world data are collected, collated and quality-checked by national register holders.
- Data are harmonized according to a Common Data Model, which enables use of the same statistical coding and uniform data analysis across countries.
- Information on many health and sociodemographic factors facilitates comprehensive adjustment for potential confounding factors.
- The cohort covers four different countries, strengthening the generalisability of concordant results

Limitations:

- Data have been collected for administrative purposes and is therefore limited to the information available in the registers.
- Vaccines given outside the immunisation programmes e.g. travel vaccinations, are not registered in all countries.
- The data harmonisation process may entail loss of details of country-specific data e.g. due to dichotomisation of variables that have more levels in some countries but not all.
- Due to data protection regulations, datasets are stored separately in each country and pooled analyses of individual-level data from all countries have not been possible.
- Analyses of observational data entail an inherent risk of residual confounding.

For peer review only - http://bmjopen.bmj.com/site/about/guidelines.xhtml

INTRODUCTION

An accumulating number of epidemiological and immunological studies have found that vaccines, in addition to the disease-specific protection, may have so called non-specific effects affecting susceptibility towards other diseases than the vaccine-targeted infections (1, 2). Most previous studies on non-specific effects stem from low-income countries with a high infectious disease burden and have had overall childhood mortality as the outcome. The non-specific effects are found to vary depending on the type of vaccine being administered. Live vaccines have been associated with beneficial non-specific effects (1, 2). Non-live vaccines, although protecting against the vaccine-targeted infections, may possibly increase susceptibility to other infections (1, 2). The effects are most pronounced for the most recently administered vaccine (2).

Studies of non-specific effects from high-income countries have primarily focused on infectious disease morbidity (3) and atopic diseases (4-8). Most of these studies are observational, because it would (often) be unethical to randomise children to refrain from or delay recommended childhood vaccinations. Therefore, concerns about different types of bias in different settings and observational designs have been raised (9-12). Triangulation has been proposed as a method to strengthen causal inference in epidemiology by integrating results from several epidemiological designs and between different populations with different bias structures while using the same analysis plan across settings to enhance comparability of results (13, 14).

The “NONSEnse” project is a Nordforsk-funded collaboration between research groups in the four Northern European countries Denmark, Finland, Norway, and Sweden (henceforth referred to as the Nordic countries). The main aim of “NONSEnse” is to do careful evaluation of potential non-specific effects of childhood vaccinations in the Nordic countries. The main hypothesis underlying this evaluation is that having a live vaccine as the most recent vaccine is associated with a lower morbidity in the following time-period, compared with having a non-live vaccine as the most recent vaccine. We will analyse specific research questions using the same methodology in all countries, but also apply different analytical approaches in different studies to facilitate triangulation of the results. The main associations we will examine are associations between childhood vaccinations

For peer review only - http://bmjopen.bmj.com/site/about/guidelines.xhtml

and 1) infectious disease hospitalisations, 2) antibiotic use, and 3) atopic diseases (asthma, atopic dermatitis, allergic rhinoconjunctivitis).

The first step has been to examine and compare infectious disease and atopic morbidity among children in the respective countries over time and by age and sex, to inform choice of design and outcome definitions in the subsequent studies of non-specific effects of vaccines.

COHORT DESCRIPTION

Setting

The Nordic countries have many similarities including the welfare state model with universal tax-funded healthcare and a high level of social security. A detailed description of the Nordic health care systems and basic demographics has been published elsewhere (15).

National immunisation programmes

Childhood vaccinations within the national immunisation programmes (NIP) are voluntary and administered free of charge in all four countries. In Denmark, all childhood vaccines are administered by family practitioners (16). In Finland, Norway, and Sweden vaccines scheduled before school-age are administered at well-baby clinics by nurses; during school age, the vaccines are administered by school nurses (17-19). In 2018, children were offered vaccinations against 10 diseases in Denmark (16), up to 13 diseases in Finland (17), 12 diseases in Norway (19), and 10 diseases in Sweden (18). Children in specific risk groups are offered vaccines against additional diseases according to national guidelines (17-20). An overview of recommended childhood vaccinations in the four countries in 2018 is presented in Table 1 and historical changes are illustrated in Appendix 1.

For peer review only - http://bmjopen.bmj.com/site/about/guidelines.xhtml

Table 1: Vaccines recommended to children in Denmark, Finland, Norway, and Sweden in 2018. The vaccines are included in the childhood immunisation programmes and registered in the vaccination registers, unless otherwise specified.

Disease (Vaccine)	Denmark	Finland	Norway	Sweden
**Tuberculosis (BCG)**	Not within programme	Before 7 years of age, risk groups only[1]	6 weeks of age, risk groups only[1]	After 6 months of age, risk groups only [1,2]
**Hepatitis A**	Not within programme	From 1 year of age, risk groups only[3]	Not within programme	Not within programme
**Hepatitis B**	From birth, risk groups only[4]	From birth, risk groups only[4,5]	3 doses: 3, 5, 12 months of age	Not within programme but recommended to all children.<br>3 doses: 3, 5, 12 months of age[6]
**Rotavirus**	Not within programme	3 doses: 2, 3, 5 months of age	2 doses: 6 weeks, 3 months of age	2 or 3 doses: 6 weeks, 3 and 5 months of age[2, 7]
**Diphtheria, tetanus and pertussis (DTaP)**	4 doses: 3, 5, 12 months, booster at 5 years of age	5 doses: 3, 5, 12 months of age, booster at 4 and 14 years of age	5 doses: 3, 5, 12 months of age, Booster in 2nd and 10th school-year	5 doses: 3, 5, 12 months of age, booster at 5 years of age and in 8th or 9th school-year
**Polio (IPV)**	4 doses: 3, 5, 12 months, booster at 5 years of age	4 doses: 3, 5, 12 months of age, booster at 4 years of age	5 doses: 3, 5, 12 months of age, booster in 2nd and 10th school-year	4 doses: 3, 5, and 12 months of age, booster at 5 years of age
**Haemophilus influenzae type B**	3 doses: 3, 5, 12 months of age	3 doses: 3, 5, 12 months of age	3 doses: 3, 5, 12 months	3 doses: 3, 5, and 12 months of age

For peer review only - http://bmjopen.bmj.com/site/about/guidelines.xhtml

**Pneumococcal disease (PCV)**	13-valent; 3 doses: 3, 5, 12 months of age	10-valent; 3 doses: 3, 5, 12 months of age	13-valent; 3 doses: 3, 5, 12 months of age	10 or 13-valent; 3 doses: 3, 5, and 12 months of age
**Influenza (Live- or non-live influenza vaccine)**	From 6 months of age, risk groups only[8]	Yearly, from 6 months to 6 years of age and for risk groups after 6 years of age[8]	From 6 months of age, risk groups only, through the influenza immunisation programme[8]	Yearly, from 6 months of age, risk groups only[2,8]
**Measles, mumps and rubella**	2 doses: 15 months of age and 4 years of age	2 doses: 12 months of age, 6 years of age	2 doses: 15 months of age, and 6th school-year	2 doses: 18 months of age and 1st or 2nd school-year
**Varicella**	Not recommended	1.5-11 years of age	Not recommended	Not recommended
**Pneumococcal disease (PPV)**	Not within programme	Before 5 years of age, after PCV, risk groups only[9]	Not within programme, but recommended from 2 years of age, to specified risk groups[9]	Not within programme, but recommended from 2 years of age, to specified risk groups[2,9]
**Tick borne encephalitis**	Not within programme	From 3 years of age, risk groups only[10]	Not within programme	Not within programme
**Human papilloma virus**	2 doses: 12 years of age, girls only	2 doses: 6th school-year, girls only	2 doses in 7th school-year	2 doses in 5th or 6th school-year, girls only

Abbreviations: BCG: Bacillus Calmette-Guérin vaccine; DTaP: Diphtheria, tetanus, and acellular pertussis vaccine; IPV: Inactivated Polio Vaccine; PCV: Pneumococcal Conjugated Vaccine; PPV: Pneumococcal Polysaccharide Vaccine.
Information obtained from: The Danish health authority (16), Finnish institute for health and Welfare (17), Norwegian Institute of Public Health (19), The Public Health Agency of Sweden (18).

For peer review only

For peer review only - http://bmjopen.bmj.com/site/about/guidelines.xhtml

[1] Children with a parent from a country with a high incidence of tuberculosis. [2] Not included in the vaccination registry, [3] Children of intravenous drug users. [4] 1) Children of mothers or another member of the household who are Hepatitis B positive, or 2) attend daycare with a child who has Hepatitis B (20, 21). [5] 1) children of parents from countries with high incidence of Hepatitis B, or 2) children of mothers with hepatitis C infection (21). [6] Only offered to children in the risk group before 2016, not included in the vaccination registry before 2016 (22). [7] Rotavirus vaccine was offered by some Swedish regions, as part of regional vaccination schemes.[8] Children with increased risk of severe influenza illness or members of households with high-risk individuals(18, 23-25). [9] Children with increased risk of severe pneumococcal disease e.g. children with chronic diseases (18, 26, 27). [10] Children of families with a permanent home or holiday house in areas within Finland with high tick prevalence (28)

For peer review only

Nordic nationwide register data: a goldmine for epidemiological studies

All individuals residing in the Nordic countries are assigned a unique personal identification code (ID). All four countries have extensive national registers on health, demographic factors, and socio-economic factors collected for administrative purposes and linked to the individual using the personal ID (15, 29). The register information is collected automatically, which minimizes systematic reporting bias e.g. recall bias. The use of national registers limits selection bias as the entire population is included. All information in the registers are dated, which ensures that exposures and outcomes can be temporally linked and facilitates investigation of the cumulative and combined effects of multiple interventions on childhood health. Thus, the structure of the Nordic registers presents a unique opportunity to investigate the real-life effects of childhood vaccinations while incorporating multiple potential confounding factors.

Study population

We used national population registers to identify all children aged 0-17 years, who were born or permanently living in one of the Nordic countries at some point from 1990 until and including 2018 in Denmark and Norway, and 2017 in Finland and Sweden (30-33) (Figure 1). The population data obtained in Finland had incomplete information on migration history before 2014 and thus we were unable to assess the date of entering the country for children born abroad. As a result, we limited the Finnish study population to children born in the country to ensure that they were present in the country from the beginning of follow-up. After exclusions, which were primarily due to uncertain information about residency, a

total of 9,072,420 children were included across the countries (Figure 1). Children were followed from date of birth or date of immigration until the date of first emigration, 18-year birthday, death, or last date with available information, whichever came first.

Source and content of data

Using the personal ID, we linked information from the nationwide registers and obtained individual-level information on gestation and birth, hospital contacts, redeemed prescriptions, and receipt of childhood vaccines. Furthermore, each child was linked to their parents through the population registers in order to extract information on household income, family composition, and highest attained parental education (Figure 2).

Information on administered vaccines including type of vaccine and date of vaccination was obtained from The Danish Vaccination Register in Denmark (34), the Finnish vaccination register in Finland (35), the Norwegian Immunisation Registry (SYSVAK) in Norway (36), and the National vaccination register in Sweden (37). Registration of vaccinations within the NIP is mandatory in all Nordic countries (Table 1).

The Danish Vaccination Register includes information from the Danish National Health Insurance System that collects information on all vaccinations within the NIP (38). Since 2015, it has also been mandatory to report on vaccines given outside the NIP (39). In Denmark, vaccine information is linked to the individual using the personal ID, however before 1997 the information was registered on the ID of the parents only (38). Thus, in Denmark, only information on vaccines administered from 1997 and later was included. In Finland, the register includes all vaccines given in public health care since 2009, and after 2016 also private health care is obligated to register vaccinations (35). In Norway, the immunisation registry holds information since 1995 on all administered vaccines that are part of the NIP (19). Since 2011 notification to the immunisation registry is also mandatory for vaccines given outside the NIP (36). The Swedish national vaccination register has information about vaccinations given since 2013, but only those included in the NIP (37).

Information on hospital contacts was obtained from nationwide patient registers (15, 29), which reached national coverage and recorded individual-level data since 1978 in Denmark, 1994 in Finland, 2008 in Norway, and 1997 in Sweden. Since 1997, diagnoses have been coded according to the International Classification of Diseases version 10 (ICD-10) in all four countries (40).

For peer review only - http://bmjopen.bmj.com/site/about/guidelines.xhtml

The Danish, Norwegian, and Swedish prescription registers hold information on all redeemed prescriptions, classified using the Anatomical Therapeutic Chemical Classification System (ATC) since 1995, 2004, and mid-2005, respectively (41). The Finnish Benefits Registry holds information only for reimbursable redeemed prescriptions (41-43). In addition, the Finnish Prescription Center started gradually in 2010 and collects all redeemed prescriptions irrespective of reimbursement. By 2017, practically all prescriptions were included in the Finnish Prescription Center (44).
Information on socioeconomic factors and birth characteristics was available from the beginning of the study period (1990) in all countries.

The Common Data Model: Harmonised country specific datasets

The country-specific data from the national registers may differ both across countries and within countries over time due to differences in coding practices, administration, and country-specific legislation on health and social aspects (29). We developed a Common Data Model to harmonise all information we obtained into similar datasets using the same variable names and same categories in all four countries (Figure 3). The data harmonisation focused on identifying outliers and country-specific traits that could hinder cross country comparability. Information on source of data and data preparation for each of the variables can be found in Appendix 2 “NONSense Common Data Model”.

Due to national data protection legislation, country-specific data were stored and analysed in the respective countries.

Patient and Public involvement

All studies conducted within NONSEnse will be register based studies only and patients or the public will not be involved in the design or conduct of the planned studies.

Characteristics of the study population

The national study populations range from 1,637,133 children in Finlandto 3,540,560 children in Sweden (Table 2). Mean follow-up time was 11.6 years in Denmark, 12.3 years in Finland, 11.5 years in Norway, and 10.3 years in Sweden. Sweden had the highest proportion of children born abroad; 15.5% compared with 8.4 in Denmark and 11.1% in Norway. The proportion of children who were censored due to migration was lower in Finland, where we only included children born in-country: 0.7% compared with 4.4-6.2% in

For peer review only - http://bmjopen.bmj.com/site/about/guidelines.xhtml

the other countries. The lower emigration rate in Finland represents both underreporting due to incomplete information on migration, and a suspected lower risk of moving out of the country for children born in-country, compared with children born abroad. A higher proportion of children without a link to their mother was seen in Sweden; 5.3% compared to 0.2-1.1% in the other countries. The children without a link to their mother in Sweden were predominantly born abroad (data not presented) and may thus be affected by incomplete registration of migrant families, or children immigrating to Sweden without their mother.

For peer review only

Table 2: Study population – identification and follow-up

	Denmark		Finland[1]		Norway		Sweden	
**Study population (N)**	1,979,670		1,637,133		1,915,057		3,540,560	
**Years of follow-up[2] per child (Mean (sd))**	11.6 (6.3)		12.3 (6.0)		11.5 (6.3)		10.3 (6.9)	
**Year of birth**	1990-2018		1990-2017		1990-2018		1990-2017	
**Sex (N (%))**								
Male	1,014,745	51.3%	836,828	51.1%	985,568	51.5%	1,827,619	51.6%
Female	964,925	48.7%	800,305	48.9%	929,489	48.5%	1,712,941	48.4%
**Reason for entering the cohort (N (%))**								
Birth	1,813,443	91.6%	1,637,133	100.0%	1,703,054	88.9%	2,993,472	84.5%
Immigration	166,227	8.4%	0	0.0%	212,003	11.1%	547,088	15.5%
**Reason for leaving the cohort (N (%))**								
Death	8532	0.4%	761	0.0%	5422	0.3%	5614	0.2%
Emigration	122,916	6.2%	11,789	0.7%	95,406	5.0%	154,878	4.4%
Other[3]	1917	0.1%	0	0.0%	0	0.0%	0	0.0%
18th birthday	704,518	35.6%	608,644	37.2%	703,164	36.7%	1,280,027	36.2%
End of follow-up[4]	1,141,787	57.7%	1,015,939	62.1%	1,111,065	58.0%	2,100,041	59.3%
**Linked with mother in registers (N (%))**	1,961,595	99.1%	1,634,120	99.8%	1,894,916	98.9%	3,352,706	94.7%
**Linked with father in registers (N (%))**	1,920,008	97.0%	1,601,138	97,8%	1,838,444	96.0%	3,248,108	91.7%
**Maternal age at birth of child (median, p25-p75)**	29 (26-33)		29 (26-33)		29 (25-33)		29 (26-33)	
**Missing information on maternal age (N (%))**	18,075	0.9%	10,099	0.6%	20,141	1.1%	187,854	5.3%
**Maternal origin (N (%))**								
Born in country	1,582,885	80.0%	1,520,159	92.9%	1,432,179	74.8%	2,399,234	67.8%
Born abroad	378,710	19.1%	111,611	6.8%	462,319	24.1%	953,467	26.9%
Unknown	18,075	0.9%	5363	0.3%	20,559	1.1%	187,859	5.3%

[1]Finnish data only include children born in country due to incomplete information on migrations. [2]Years of follow-up is calculated as first date of death, emigration, turning 18 years of age or last date with available data from the population registry minus the last date of birth, or immigration divided by 365.25. [3]E.g. disappear from register without specification. [4]Last date with data available from population registry

For peer review only

For peer review only - http://bmjopen.bmj.com/site/about/guidelines.xhtml

Exposure assessment: Vaccinations across the Nordic countries

Figure 4 depicts the coverage of diphtheria, tetanus, and acellular pertussis containing vaccines (DTaP), measles-mumps-rubella vaccine (MMR) and rota virus vaccines (rota) for children born in each country followed from birth until two years of age, date of emigration, or date of death, whichever came first (see Appendix 3, sTable 1 for the coverage at 2 years of age for each of the included birth cohorts in each country).

In Norway, the vaccine uptake rate was highest and closest to the age of recommended vaccination compared with the other countries. In Finland and Sweden, MMR uptake starts at ages earlier than scheduled according to the respective NIPs, which reflects that MMR is recommended to children from 6 and 9 months of age in Finland and Sweden respectively, before travelling abroad. Although MMR is recommended before travelling abroad in all the Nordic countries, early uptake of MMR is much less frequent in Denmark and Norway which may indicate different interpretation and roll-out of the recommendations. The greater variation in the age at MMR vaccination in Finland reflects different vaccination schedules applied to the included birth cohorts: MMR vaccination was recommended at 14-18 months of age before June 2010 and at 12-18 months (preferably 12 months of age) after June 2010. In Finland, Norway, and Sweden, the date of the next vaccination is usually scheduled during earlier well-baby check-ups or provided by post, whereas in Denmark no formal procedures are in place to ensure timely vaccination, which may explain the different variation in age at vaccination across the countries.

Human papilloma virus vaccination (HPV) for girls was introduced in the NIP in 2009 in Denmark, the end of 2013 in Finland, mid-2009 in Norway, and in 2012 in Sweden. (Appendix 1). The vaccine is recommended at age 12 years in Denmark, Finland, and Norway, and at age 11-12 years in Sweden. Figure 5 depicts the registered coverage of HPV vaccinations among girls followed from one year before the recommended age of vaccination until age 14 years, emigration, or death, whichever came first. In Norway, the uptake of the first dose of HPV vaccine follows a steep curve at 12 years of age, representing the age of recommended vaccination (Figure 5). The majority of the included birth cohorts in Norway were only able to receive the HPV vaccination free of charge during the school year it was offered, which may have contributed to the high and steep uptake rate. In Sweden, the uptake starts increasing at 11 years of age with a second

For peer review only - http://bmjopen.bmj.com/site/about/guidelines.xhtml

increase at 12 years of age reflecting that the vaccine may be administered in either the 5th or 6th grade. In Denmark uptake starts increasing at 12 years of age corresponding to recommended age of vaccination, but with more variation in the age of vaccination compared with the other countries. The relative low uptake combined with high age variation may be due to vaccination hesitancy following negative media attention from Danish television portraying alleged serious adverse effects of HPV vaccination (45). Confidence in the safety of the vaccine has since been restored, which is reflected in the slightly increasing vaccination coverage in the last included birth cohort (Appendix 3, sTable2). In Finland, the uptake rates follow a straight curve from 12-13 years of age followed by a small proportion of children with delayed vaccination. The vaccine uptake at 14 years of age within our cohort was highest in Norway (first dose for the birth cohort 2003: 84.8%) followed by Sweden (77.9%), Finland (69.8%) and Denmark (52.3%) (Appendix 3, sTable 2).

Health and sociodemographic characteristics

Data were available for a different set of years across the Nordic countries. For comparing the study populations in this cohort profile, we only present information from years where data are available in all countries.

Prescriptions

Information on redeemed prescriptions was included for the purpose of assessing predefined health outcomes in terms of antibiotic consumption and different atopic outcomes, and to be able to assess potential confounding factors relating to underlying health and healthcare seeking behaviour. The data legislation regulating access to information on drug utilisation differed across countries. Therefore, data were only obtained for a more narrowly defined subset of ATC-codes in Finland and Sweden, compared with Denmark and Norway (Appendix 3). Information on redeemed prescriptions was available from 2005 to 2017 in all countries. We only included information on redeemed prescriptions with ATC-codes available in all countries for the present comparison. The overall proportion of children with redeemed prescriptions ranged from 75.6% in Norway to 86.1% in Finland and varied depending on ATC-group (Table 3). The proportion of children with redeemed prescriptions in ATC-group D “dermatologicals” was 36.3% in Denmark compared with 20.6-24.7% in the other countries. Finland had the

For peer review only - http://bmjopen.bmj.com/site/about/guidelines.xhtml

highest proportion of children with redeemed prescriptions in ATC group J “antiinfectives for systemic use”: 82.3% compared with 62.1-75.0% in the other countries. In ATC group S “eye and ear medications”, the proportion was lower in Finland (7.4%) compared with the other countries (13.0-17.9%). For ATC group R “Respiratory system” and subgroup V01 “Allergens” the proportions were relatively similar across countries.

For peer review only

For peer review only - http://bmjopen.bmj.com/site/about/guidelines.xhtml

Table 3: Health characteristics of children present in the respective countries from year 2005-2017.

	Denmark		Finland		Norway		Sweden	
**Prescriptions**								
Years of follow-up	2005-2017		2005-2017		2005-2017		2005-2017	
Number of children with follow-up[1] (N (%))	1,904,633	100.0%	1,634,031	100.0%	1,817,231	100.0%	3,355,915	100.0%
Children with redeemed prescriptions[2] (N (%))	1,592,361	83.6%	1,407,548	86.1%	1,374,180	75.6%	2,542,676	75.8%
Mean age during follow-up (mean, (sd))	8.3 (5.2)		8.3 (5.2)		8.3 (5.2)		8.2 (5.3)	
Prescriptions pr child (median, p25-p75)	4 (1-9)		5 (2-11)		3 (1-8)		3 (1-7)	
Children with prescriptions with ATC group D (N (%))	691,357	36.3%	360,910	22.1%	449,226	24.7%	692,269	20.6%
Prescriptions per child with ATC group D[2, 3, 4] (median, p25-p75)	1 (1-3)		1 (1-3)		1 (1-3)		1 (1-3)	
Children with prescriptions with ATC group J (N (%))	1,428,652	75.0%	1,345,297	82.3%	1,129,065	62.1%	2,194,753	65.4%
Prescriptions per child with ATC group J[2, 3, 5] (median, p25-p75)	3 (2-6)		4 (2-8)		2 (1-4)		3 (1-5)	
Children with prescriptions with ATC group R (N (%))	806,105	42.3%	748,839	45.8%	841,066	46.3%	1,468,158	43.7%
Prescriptions per child with ATC group R[2, 3, 6] (median, p25-p75)	2 (1-6)		2 (1-7)		3 (1-9)		2 (1-6)	
Children with prescriptions with ATC group S (N (%))	248,522	13.0%	121,721	7.4%	326,077	17.9%	521,658	15.5%
Prescriptions per child with ATC group S[2, 3, 7] (median, p25-p75)	1 (1-2)		1 (1-2)		2 (1-3)		1 (1-2)	

For peer review only

Children with prescriptions with ATC group V01(N (%))	10,384	0.5%	4662	0.3%	11,770	0.6%	5928	0.2%
Prescriptions per child with ATC group V01[2, 3, 8] (median, p25-p75)	5 (3-9)		4 (2-7)		4 (2-8)		4 (2-8)	
**Hospital contacts**								
Years of follow-up	2008-2016		2008-2016		2008-2016		2008-2016	
Number of children with follow-up[9] (N (%))	1,813,600	100.0%	1,581,854	100.0%	1,738,115	100.0%	3,177,371	100.0%
Children with hospital contacts (N (%))	1,069,628	59.0%	861,685	54.5%	982,808	56.5%	1,911,254	60.2%
Years of follow-up (mean, (sd))	5.8 (3.1)		5.9 (3.0)		5.7 (3.0)		5.5 (3.1)	
Mean age during follow-up (mean, (sd))	9.2 (5.8)		9.1 (5.9)		9.1 (5.8)		9.0 (5.9)	
Hospital contacts per child (main diagnosis)(median, p25-p75)	1 (0-2)		1 (0-4)		1 (0-3)		1 (0-4)	
Children with inpatient contacts (N (%))	519,945	28.7%	324,292	20.5%	420,492	24.2%	568,958	17.9%
Inpatient contacts per child (median, p25-p75)	1 (1-2)		1 (1-2)		1 (1-2)		1 (1-2)	
Children with outpatient or emergency room contacts (N (%))	885,243	48.8%	839,569	53.1%	911,877	52.5%	1,826,446	57.5%
Outpatient or emergency room contacts per child (1 per day) (median, p25-p75)	2 (1-3)		3 (1-6)		2 (1-5)		3 (1-6)	

[1] Number of children living in the country at any time in the period 2005-2017. [2] Only including ATC subgroups: D02AF, D05, D07, D11, D01, D06, D08, J01-J07, R01, R03, R06, S01G, S03, V01 - Thus, not reflecting total use of prescription medicines.[3] Per child with redeemed prescriptions of that ATC-group. [4]ATC Group D: Dermatologicals. [5]ATC Group J: Antiinfectives for systemic use. [6]ATC Group R: Respiratory system. [7]ATC Group S: Sensory organs. [8]ATC Subgroup V01: Allergens. [9] Number of children living in the country at any time in the period 2008-2016. Proportions are calculated using number of children with follow-up as the denominator.

For peer review only

Hospital contacts

Information on hospital contacts including inpatient and specialised outpatient care was available in all countries from 2008 to 2016. For comparison across countries, we excluded country-specific codes (e.g. codes for health characteristics of new-borns in Denmark). The proportion of children with hospital contacts was similar across countries (54.5-60.2%, Table 3). The proportion of children with inpatient contacts ranged from 17.9% in Sweden to 28.7% in Denmark. The proportion of children with outpatient contacts in the patient registers was highest in Sweden (57.5%) and lowest in Denmark (48.8%). The higher proportion of inpatient contacts in Denmark is likely explained by contributions of inpatient contacts without overnight stays, as contacts without overnight stays will predominantly be registered as outpatient contacts in the other countries (46). The higher proportion of children with outpatient contacts in Sweden may on the other hand be explained by a broader set of health care facilities (e.g. paediatric outpatient clinics) that report to the patient register in Sweden compared with the other countries (46).

Birth characteristics

Information on birth characteristics was available for birth cohorts from 1990 to 2016 in all countries (Table 4). The completeness of data was high in all countries, ranging from 97.7% to 99.9%. The birth characteristics were also very similar: the median birth weight ranged from 3500 to 3550 grams, the proportion of low-birth-weight children ranged from 3.9% to 5.0%, and the median gestational age was 40 weeks in all countries. For the variables preterm birth, delivered by caesarean section, and singleton births, the proportions only differed by 0.8-2.7 percent points across countries. The greatest difference between countries was seen for registration of maternal smoking during pregnancy, which ranged from 8.3% in Norway to 18.2% in Denmark. The proportion with unknown/missing information on maternal smoking ranged from 2.5% in Finland to 45.8% in Norway, which may be explained by the midwifes having to inform the mothers of the need for obtaining information on smoking before asking this question in Norway, thus additional effort is required, which may hamper completeness.

For peer review only

Table 4: Birth characteristics[1]

	Denmark		Finland		Norway		Sweden	
Children born in the respective country from 1990-2016 (N)	1,728,126		1,586,526		1,591,273		2,877,753	
Children with information available from the birth registry	1,726,318	99.9%	1,576,797	99.4%	1,586,895	99.7%	2,811,119	97.7%
Birth weight in grams (Median, p25-p75)	3500 (3150-3850)		3550 (3210-3880)		3550 (3200-3900)		3540 (3200-3890)	
Low birth weight (<2500g) (N (%))	86,914	5.0%	61,546	3.9%	73,437	4.6%	114,990	4.0%
Birth weight missing (N (%))	23,707	1.4%	12,859	0.8%	5376	0.3%	72,892	2.5%
Gestational age in weeks (Median, p25-p75)	40 (39-41)		40 (39-40)		40 (39-41)		40 (39-40)	
Preterm birth (N (%))	107,656	6.2%	85,069	5.4%	98,923	6.2%	163,168	5.7%
Gestational age missing (N (%))	29,250	1.7%	16,083	1.0%	59,264	3.7%	68,973	2.4%
Delivered by caesarean section (N (%))	305,738	17.7%	258,261	16.3%	238,013	15.0%	435,680	15.1%
Mode of delivery missing (N (%))	1808	0.1%	9729	0.6%	4378	0.3%	66,634	2.3%
Singleton (N (%))	1,660,213	96.1%	1,531,748	96.5%	1,535,556	96.5%	2,731,980	94.9%
**Child order including the child itself (N (%))**								
1 (firstborn)	743,923	43.0%	647,134	40.8%	658,877	41.4%	1,211,084	42.1%
2	635,849	36.8%	532,868	33.6%	568,765	35.7%	1,023,228	35.6%
3	243,162	14.1%	244,137	15.4%	257,294	16.2%	398,331	13.8%
4 or more	86,090	5.0%	149,327	9.4%	101,959	6.4%	178,184	6.2%
Missing	19,102	1.1%	13,060	0.8%	4378	0.3%	66,926	2.3%

For peer review only

Maternal smoking during pregnancy (N (%))	314,174	18.2%	238,337	15.0%	132,734	8.3%	310,691	10.8%
Maternal smoking unknown (N (%))	134,332	7.8%	39,277	2.5%	728,038	45.8%	143,529	5.0%
[1] Information, including percentages are reported according to the number of children born in country from 1990-2016								

Socioeconomic factors

Socioeconomic information is collected yearly in all countries. In the NONSEnse cohort, the information was assessed in the year of birth of each child (Table 5) and in the 10th year of life (Appendix 3, sTable 3). Information from the year of birth was available for the birth cohorts 2004-2015 in all countries. The data presented in Table 5 only include children who were born in-country and living in the country throughout their first year of life, to ensure that they were present in the country at the time of registration.

For peer review only

Table 5: Socio-economic factors at birth

	Denmark		Finland		Norway		Sweden	
	N	(%)	N	(%)	N	(%)	N	(%)
Children present in country at birth from 2004-2015	729,294		699,052		706,443		1,314,701	
Birth cohorts included	2004-2015		2004-2015		2004-2015		2004-2015	
Income quintile at birth								
First (lowest)	134,634	18.5%	138,965	19.9%	137,551	19.5%	247,237	18.8%
Second	137,041	18.8%	138,997	19.9%	141,566	20.1%	265,557	20.2%
Third	137,390	18.8%	139,012	19.9%	141,962	20.1%	267,347	20.3%
Fourth	137,415	18.9%	138,998	19.9%	141,995	20.1%	267,349	20.3%
Fifth	136,935	18.8%	138,900	19.9%	141,533	20.1%	266,528	20.3%
Unknown	45,531	6.2%	4180	0.6%	644	0.1%	605	0.0%
Number of children in the household the year the child is born								
1	310,237	42.6%	287,312	41.1%	298,563	42.3%	574,229	43.7%
2	278,396	38.2%	237,291	33.9%	263,726	37.4%	487,446	37.1%
3	106,184	14.6%	104,278	14.9%	108,822	15.4%	176,338	13.4%
>3	32,106	4.4%	65,527	9.4%	33,496	4.7%	68,060	5.2%
Unknown	2023	0.3%	4644	0.7%	644	0.1%	605	0.0%
Single parenthood in the years the child is born								
Yes	58,646	8.0%	55,089	7.9%	68,018	9.6%	132,243	10.1%
No	668,277	91.7%	639,319	91.5%	635,689	90.1%	1,181,775	89.9%
Unknown	2023	0.3%	4644	0.7%	1544	0.2%	605	0.0%
Highest attained educational level[1] of the mother on the date the child is born								
Low education	114,880	15.8%	98,608	14.1%	126,777	18.0%	149,673	11.4%
Medium education	261,761	35.9%	279,687	40.0%	201,316	28.5%	431,880	32.9%
High education	336,536	46.2%	319,530	45.7%	350,684	49.7%	457,040	34.8%
Unknown	15,769	2.2%	1227	0.2%	26,474	3.8%	276,030	21.0%

[1] Highest attained education was categorized based on the International Standard Classification of Education (ISCED) 2011 using the main groups (47).

In Denmark, 6.2% of the study population had missing information on household income compared with 0-0.6% in the other countries. We have been unable to identify the reason for the higher proportion in Denmark. The proportion of households with 3 or more children was 9.4% in Finland compared with 4.4-5.2% in the remaining Nordic countries. The proportion living with a single parent in the year of birth ranged from 7.9% in Finland to 10.1% in Sweden. Among the remaining socioeconomic variables, the largest cross-

For peer review only

country difference was found for the highest attained education of the mother, where information was missing for 21.0% of the children in Sweden compared with 0.2-3.8% in the other Nordic countries. The proportion of mothers with low education ranged from 11.4% in Sweden to 18.0% in Norway. The high proportion with missing information on maternal education in Sweden is in part caused by a higher proportion of children with an unknown mother in our dataset (Table 2) but may also be caused by education not being registered for mothers born abroad. Since registration of education is often a necessity for employment in more advanced fields, it is reasonable to assume a higher accuracy for registration of high education as compared with low education.

Findings to date

The findings to date are based on data from registers, where we were able to obtain data prior to receiving the final individual-level data with linkage across all registers within each country.

We have conducted investigations of similarities and differences in rates of infectious disease hospitalisations (46), and antibiotic consumption (under review). These studies highlight trends in infectious disease morbidity across the Nordic countries and further guide the use of more consistent infectious disease outcome measures for future studies. We have furthermore investigated if changes in the recommended age at revaccination with MMR in Denmark and Sweden affected the rate of infectious disease hospitalisations using an interrupted time series analysis (48).

Future studies will include population-level investigations of natural experiments in the form of introduction of new vaccines or changes in the immunisation programmes, as well as individual-level studies comparing vaccinated and unvaccinated children with a given vaccine using multiple different study designs.

FURTHER DETAILS

Strengths and limitations

The NONSEnse project represents a unique undertaking for conducting register based epidemiological studies of the overall health effects of routine childhood vaccines.

For peer review only

Data are stored separately in each country, which prevents conducting analyses on the joint data, which is a limitation of the project. However, the Common Data Model enables analysis plans and statistical code to be written in one country and sent to the other countries that can then perform the same analyses and share the results (Figure 3). The use of a Common Data Model thus minimises the risk that different country-specific analytical decisions will hinder comparability of results.

The use of register data presents both strengths and weaknesses. A strength pertains to the multitude of information available for the entire study population and linked to the individual, which minimises selection bias and enables cohort studies with prospective follow-up and control for multiple confounding factors. Limitations include that not all the wished-for information are available in all countries and registration may be incomplete, which limits the possibility to e.g. adjust for hypothesised confounding factors such as day-care attendance and lifestyle factors. Also, previous studies (2) have found the non-specific effect of a vaccine to be strongest when it is the most recent vaccine administered. Therefore, it is relevant to include information on vaccines other than the ones offered through the NIP. In Denmark, Finland, and Norway vaccines outside the NIP may also be registered in the vaccination registers, but registration of these vaccines has only been mandatory in more recent years (35, 36, 39). In Sweden only vaccinations within the NIP are included in the vaccination register. The analyses are thus limited by different possibilities to assess the effect of a given vaccine as long as it is the most recent vaccine, both within and across countries.

In all the Nordic countries information on emigration relies on the individual reporting resettlement to the authorities. This is mandatory when leaving the country for more than 6 months in Denmark (49) and Norway (50), and for more than 12 months in Sweden (51) and Finland (52). Thus, incomplete information on emigrations, due to leaving the country for shorter periods of time or if parents fail to register the resettlement, may result in children being lost to follow-up without us knowing it from the registers. This may in turn result in our studies underestimating events, e.g. infectious disease hospitalisations, as these are only registered for children who are in the country.

Overall, it is clear that expert knowledge is needed before combining and using Nordic register data for research purposes (29). As such, an important strength of NONSEnse pertains to the data harmonisation process through bi-weekly analysis workshops involving

For peer review only

designated research groups from each of the four countries with expert knowledge on country-specific register data, the heath care systems, and immunisation programmes.

Validity of exposure and outcome measures

In all countries, the vaccines offered through the NIP are subject to mandatory registration. However, validity depends on the reporting accuracy by the health care providers that administer the vaccinations. A Danish study validated the coverage of MMR from the registers using medical records from the general practitioner in a subset of the population and found that the coverage in the register was 86% compared with 94% through inspection of the medical records (53). A similar comparison conducted in Sweden also found underreporting of MMR in the register of around 5-7 percentage units (unpublished). It is unlikely that underreporting of vaccines is associated with the outcomes investigated within the NONSEnse project, therefore, the misclassification will most likely be non-differential and would thus bias the results towards no association.

The prescription registers only contain information on drugs dispensed from filled prescriptions, whereas some drugs are also available over the counter, which are not included in the registers. This includes e.g. weak corticosteroids for topical use (ATC: D07AA) or drugs used to treat symptoms in the eye due to e.g. allergy (ATC: S01G). It is thus possible that the observed cross-country differences in the proportion of children with these prescriptions are affected by national policies or guidelines, or the behaviour of the prescriber or purchaser. Atopic outcomes will, in part, be identified using filled prescriptions for products that are also available over the counter, which may hamper cross country comparability. Antibiotics, however, are prescription drugs in all four countries and thus not affected by over-the-counter purchases.

Several differences in health care organisation, administration, and registration may hamper cross country comparability of the health outcomes included in this project. A strength of NONSEnse is the thorough investigation of the intended outcomes in independent studies which has informed and maximised comparability of the outcome measures to be used in the subsequent studies of non-specific effects of vaccines.

Methodological considerations

Evaluating the effect of implemented vaccination programmes is challenging; the high vaccine uptake rate makes comparisons between vaccinated and unvaccinated children difficult due to the individual factors that determine vaccine uptake. Healthy vaccinee bias may arise if the healthiest children are more likely to follow the vaccination recommendations than the less healthy children (54). However, due to different vaccination schedules in different countries, the children who have received MMR at e.g.15 months of age may be classified as vaccinated according to schedule, too early or too late, depending on the country. Furthermore, age is a strong predictor of both vaccination and the risk of infectious diseases (46). A strength therefore pertains to the observed delay in age at vaccination within each country, which facilitates comparison of different vaccination statuses among children of the same age. For vaccines with a steep and high uptake at the recommended age of vaccination, the children who do not receive the vaccines as scheduled are more likely a selected subgroup of the population, thus hampering comparability with the rest of the population. In contrast, larger variation in the age at vaccination increases comparability between children with different vaccination status according to age.

A strength of this study setup is the many differences in the immunisation programmes, and in changes to the immunisation programmes, the country-specific bias structures, and the possibility to integrate results from different study designs, which facilitate triangulation that can strengthen the potential for making causal deductions (13, 14). The project has already led to useful new information regarding differences and similarities in childhood morbidity between the Nordic countries. Most importantly, the project will increase our understanding of vaccines and how they may affect health in more general ways - holding potential for direct translation into more efficient immunisation programmes and improved child health.

Data sharing statement

Due to data protection rules, we are not allowed to share the individual-level data, but other researchers fulfilling the requirements could obtain similar data from the register controllers.

For peer review only

Funding

This work was supported by NordForsk [grant number: 83839], Odense University Hospital Research fund [A-number: 2519], and the faculty scholarship from the University of Southern Denmark [grant number N/A].

Author contributions

All authors made a significant contribution to the work reported, whether that is in the conception, study design, execution, acquisition of data, analysis, and interpretation, or in all these areas; took part in drafting, revising or critically reviewing the article; gave final approval of the version to be published; have agreed on the journal to which the article has been submitted; and agree to be accountable for all aspects of the work.

For peer review only

FIGURE LEGENDS

Figure 1: Flowchart of study population in Denmark, Finland, Norway, and Sweden

Figure 2: Nordic register information linked to the individual using a unique personal identification code (ID)

Figure 3: Transforming country specific datasets into NONSense datasets using a common data model

Figure 4: Vaccination coverage[1] according to age (inverse Kaplan-Meier estimates) among children[2] born in-country in Denmark, Finland, Norway, and Sweden

Abbreviations: DTP1: First dose of Diphtheria, Tetanus, and acellular Pertussis containing vaccine; DTP2: Second dose of Diphtheria, Tetanus, and acellular Pertussis containing vaccine; DTP3: Third dose of Diphtheria, Tetanus, and acellular Pertussis containing vaccine; MMR: Measles-Mumps-Rubella vaccine; Rota: Rota virus vaccine

[1] The coverage reflects the number of registered vaccines and may thus underestimate the actual vaccination coverage in the countries. [2] Including children born in the country from birth cohorts where vaccines administered between 0-2 years of age are registered in the vaccination registers (data availability period). The included birth cohorts are 1997-2016 in Denmark; 2009-2015 in Finland; 1995-2016 for DTP and MMR vaccine and 2015-2016 for Rota in Norway; 2013-2015 in Sweden. Number of children in each birth year is presented in Appendix 3, sTable 1.

Figure 5: Human papilloma virus vaccination coverage[1,2] according to age (inverse Kaplan-Meier estimates) among girls[3] in Denmark, Finland, Norway, and Sweden

Abbreviations: HPV1: First dose of Human papilloma virus vaccine; HPV2: Second dose of Human papilloma virus vaccine

[1]The coverage reflects the number of registered vaccinations and may thus underestimate the actual vaccination coverage. [2]In some countries the recommended vaccination schedule changed from 3 to 2 doses during follow-up. Only the 2 first doses are reported here. [3] Including girls from birth cohorts where HPV vaccination has been offered from 1 year before age of recommended vaccination until 14 years of age and where vaccinations were registered in the vaccination registers. The included birth cohorts are 1998-2004 in Denmark, 2002-2003 in Finland, 1998-2004 in Norway, and 2003 in Sweden. Number of girls included in each birth cohort is presented in Appendix 3, sTable2.

For peer review only

Competing interest

AAP and HN are investigators in vaccine-related studies for which THL has received funding from GSK, Pfizer and Sanofi Pasteur. The remaining authors report no relation that could be construed as a conflict of interest.

References

1. Higgins JPT, Soares-Weiser K, López-López JA, Kakourou A, Chaplin K, Christensen H, et al. Association of BCG, DTP, and measles containing vaccines with childhood mortality: systematic review. BMJ (Clinical research ed). 2016;355.
2. Benn CS, Fisker AB, Rieckmann A, Sørup S, Aaby P. Vaccinology: time to change the paradigm? The Lancet Infectious diseases. 2020;20(10):e274-e83.
3. de Bree LCJ, Koeken V, Joosten LAB, Aaby P, Benn CS, van Crevel R, et al. Non-specific effects of vaccines: Current evidence and potential implications. Seminars in immunology. 2018;39:35-43.
4. Gehrt L, Rieckmann A, Kiraly N, Jensen AKG, Aaby P, Benn CS, et al. Timeliness of DTaP-IPV-Hib Vaccination and Development of Atopic Dermatitis Between 4 Months and 1 Year of Age-Register-Based Cohort Study. The journal of allergy and clinical immunology In practice. 2020.
5. Kiraly N, Koplin JJ, Crawford NW, Bannister S, Flanagan KL, Holt PG, et al. Timing of routine infant vaccinations and risk of food allergy and eczema at one year of age. Allergy. 2016;71(4):541-9.
6. Rieckmann A, Hærskjold A, Benn CS, Aaby P, Lange T, Sørup S. Measles, mumps and rubella vs diphtheria-tetanus-acellular-pertussis-inactivated-polio-Haemophilus influenzae type b as the most recent vaccine and risk of early 'childhood asthma'. Int J Epidemiol. 2019;48(6):2026-38.
7. Steenhuis TJ, van Aalderen WM, Bloksma N, Nijkamp FP, van der Laag J, van Loveren H, et al. Bacille-Calmette-Guerin vaccination and the development of allergic disease in children: a randomized, prospective, single-blind study. Clinical and experimental allergy : journal of the British Society for Allergy and Clinical Immunology. 2008;38(1):79-85.
8. Thostesen LM, Kjaergaard J, Pihl GT, Birk NM, Nissen TN, Aaby P, et al. Neonatal BCG vaccination and atopic dermatitis before 13 months of age: A randomized clinical trial. Allergy. 2018;73(2):498-504.
9. Sørup S. Careful consideration of hypotheses and model assumptions in study of non-specific effects of vaccines. Vaccine. 2020;38(9):2115.
10. Gehrt L, Aaby P, Benn CS, Sørup S. Regarding "Early childhood vaccination and subsequent mortality or morbidity: are observational studies hampered by residual confounding? A Danish register-based cohort study" [Internet]. https://bmjopen.bmj.com/content/9/9/e029794.responses#regarding-%E2%80%9Cearly-childhood-vaccination-and-subsequent-mortality-or-morbidity-are-observational-studies-hampered-by-residual-confounding-a-danish-register-based-cohort-study%E2%80%9D2019. [cited 2021].
11. Tielemans S, de Melker HE, Hahne SJM, Boef AGC, van der Klis FRM, Sanders EAM, et al. Non-specific effects of measles, mumps, and rubella (MMR) vaccination in high income setting: population based cohort study in the Netherlands. BMJ (Clinical research ed). 2017;358:j3862.
12. Sinzinger AX, Von Kries R, Siedler A, Wichmann O, Harder T. Non-specific effects of MMR vaccines on infectious disease related hospitalizations during the second year of life in high-income countries: a systematic review and meta-analysis. Human vaccines & immunotherapeutics. 2019:1-9.

13. Lawlor DA, Tilling K, Davey Smith G. Triangulation in aetiological epidemiology. Int J Epidemiol. 2016;45(6):1866-86.

14. Benn CS, Fisker AB, Rieckmann A, Jensen AKG, Aaby P. How to evaluate potential non-specific effects of vaccines: the quest for randomized trials or time for triangulation? Expert review of vaccines. 2018;17(5):411-20.

15. Laugesen K, Ludvigsson JF, Schmidt M, Gissler M, Valdimarsdottir UA, Lunde A, et al. Nordic Health Registry-Based Research: A Review of Health Care Systems and Key Registries. Clin Epidemiol. 2021;13:533-54.

16. Danish Health Authority. The Danish Childhood Vaccination Programme. https://www.sst.dk/-/media/Udgivelser/2019/Boernevaccinationsprogrammet-pjece/Boernevaccinationsprogrammet-pjece-engelsk.ashx?la=en&hash=A30CD2C279D0C4C08FA22D872FD836292ED9189F; 2018.

17. Finnish Institute for health and welfare. Vaccination Programme for children and adults 2020 [Available from: https://thl.fi/en/web/infectious-diseases-and-vaccinations/information-about-vaccinations/vaccination-programme-for-children-and-adults#children.

18. The Public Health Agency of Sweden. Vaccination Programmes 2021 [Available from: https://www.folkhalsomyndigheten.se/the-public-health-agency-of-sweden/communicable-disease-control/vaccinations/vaccination-programmes/.

19. Norwegian Institute of Public Health. When will your child be offered vaccines? : Norwegian Institute of Public Health; 2020 [Available from: https://www.fhi.no/en/id/vaccines/childhood-immunisation-programme/when-will-your-child-be-offered-vaccines/.

20. Sundhedsstyrelsen. Vejledning om HIV (Human Immundefekt Virus), Hepatitis B og C virus: Forebyggelse af Blodbåren i smitte, Diagnostik og HåNdtering i Sundhedsvæsnet og På Andre Arbejdspladser.

21. Finnish Institute for health and welfare. Hepatitis B vaccine 2022 [updated 2022 Feb 1. Available from: https://thl.fi/en/web/infectious-diseases-and-vaccinations/vaccines-a-to-z/hepatitis-vaccines/hepatitis-b-vaccine.

22. Folkhälsomyndigheten. Rekommendationer om pneumokockvaccination till riskgrupper 2019 [updated 2020 August 10. Available from: https://www.folkhalsomyndigheten.se/publicerat-material/publikationsarkiv/r/rekommendationer-om-pneumokockvaccination-/.

23. Ministry of Health. Bekendtgørelse om gratis influenzavaccination til visse persongrupper. In: Ministry of Health, editor. https://www.retsinformation.dk/eli/lta/2021/20742021.

24. Finnish Institute for health and welfare. Influenza vaccine 2020 [updated 2020 Aug 14. Available from: https://thl.fi/en/web/infectious-diseases-and-vaccinations/vaccines-a-to-z/influenza-vaccine.

25. Norwegian Institute of Public Health. Influenza vaccine for risk groups 2021 [updated 2021.11.11. Available from: https://www.fhi.no/en/id/influensa/influenza-vaccine/influenza-vaccine-for-risk-groups2/.

26. Finnish Institute for health and welfare. Pneumococcal vaccines 2020 [updated 2020 Jun 8. Available from: https://thl.fi/en/web/infectious-diseases-and-vaccinations/vaccines-a-to-z/pneumococcal-vaccines.

27. Norwegian Institute of Public Health. Pneumokokkvaksine - veileder for helsepersonell 2008 [updated 2021.04.21. Available from: https://www.fhi.no/nettpub/vaksinasjonsveilederen-for-helsepersonell/vaksiner-mot-de-enkelte-sykdommene/pneumokokkvaksinasjon---veileder-fo/.

28. Finnish Institute for health and welfare. TBE, or 'tick vaccine' 2021 [Available from: https://thl.fi/en/web/infectious-diseases-and-vaccinations/vaccines-a-to-z/tbe-or-tick-vaccine-.

29. Maret-Ouda J, Tao W, Wahlin K, Lagergren J. Nordic registry-based cohort studies: Possibilities and pitfalls when combining Nordic registry data. Scandinavian journal of public health. 2017;45(17_suppl):14-9.

For peer review only

30. Ludvigsson JF, Almqvist C, Bonamy AK, Ljung R, Michaëlsson K, Neovius M, et al. Registers of the Swedish total population and their use in medical research. European journal of epidemiology. 2016;31(2):125-36.
31. Schmidt M, Pedersen L, Sorensen HT. The Danish Civil Registration System as a tool in epidemiology. European journal of epidemiology. 2014;29(8):541-9.
32. Helsedata. Befolkning 2022 [Available from: https://helsedata.no/no/forvaltere/statistisk-sentralbyra-ssb/befolkning/.
33. Digital and population data services agency. Population Information System [Available from: https://dvv.fi/en/personal-data.
34. Krause TG, Jakobsen S, Haarh M, Mølbak KJE. The Danish vaccination register. 2012;17(17):20155.
35. Baum U, Sundman J, Jaaskelainen S, Nohynek H, Puumalainen T, Jokinen J. Establishing and maintaining the National Vaccination Register in Finland. Euro Surveill. 2017;22(17).
36. Trogstad L, Ung G, Hagerup-Jenssen M, Cappelen I, Haugen I, Feiring BJE. The Norwegian immunisation register–SYSVAK. 2012;17(16):20147.
37. Chrapkowska C, Galanis I, Kark M, Lepp T, Lindstrand A, Roth A, et al. Validation of the new Swedish vaccination register - Accuracy and completeness of register data. Vaccine. 2020;38(25):4104-10.
38. Andersen JS, Olivarius NDF, Krasnik A. The Danish National Health Service Register. Scandinavian journal of public health. 2011;39(7 Suppl):34-7.
39. Hækkerup N. Bekendtgørelse om adgang til og registrering m.v. af lægemiddel- og vaccinationsoplysninger. In: Forebyggelse MfSo, editor. https://www.retsinformation.dk/eli/lta/2014/4602014.
40. Munk-Jørgensen P, Bertelsen A, Dahl AA, Lehtinen K, Lindström E, Tomasson KJNJoP. Implementation of ICD-10 in the Nordic countries. 1999;53(1):5-9.
41. Wettermark B, Zoega H, Furu K, Korhonen M, Hallas J, Norgaard M, et al. The Nordic prescription databases as a resource for pharmacoepidemiological research--a literature review. Pharmacoepidemiol Drug Saf. 2013;22(7):691-9.
42. Klaukka T. The Finnish database on drug utilisation. Norsk Epidemiologi. 2001;11(1).
43. Kanta. e-prescriptions in Kanta-archive: Kanta; [Available from: https://www.kanta.fi/en/system-developers/what-are-kanta-services.
44. Aarnio E, Huupponen R, Martikainen JE, Korhonen MJ. First insight to the Finnish nationwide electronic prescription database as a data source for pharmacoepidemiology research. Research in social & administrative pharmacy : RSAP. 2020;16(4):553-9.
45. Suppli CH, Hansen ND, Rasmussen M, Valentiner-Branth P, Krause TG, Mølbak K. Decline in HPV-vaccination uptake in Denmark – the association between HPV-related media coverage and HPV-vaccination. BMC Public Health. 2018;18(1):1360.
46. Gehrt L, Laake I, Englund H, Nieminen H, Benn CS, Feiring B, et al. Hospital Contacts for Infectious Diseases Among Children in Denmark, Finland, Norway, and Sweden, 2008-2017. Clin Epidemiol. 2022;14:609-21.
47. UNESCO Institute for Statistics. International standard classification of education: ISCED 2011. 2012.
48. Sørup S, Englund H, Laake I, Nieminen H, Gehrt L, Feiring B, et al. Revaccination with measles-mumps-rubella vaccine and hospitalization for infection in Denmark and Sweden - An interrupted time-series analysis. Vaccine. 2021.
49. Careja R, Bevelander P. Using population registers for migration and integration research: examples from Denmark and Sweden. Comparative Migration Studies. 2018;6(1):19.
50. Skatteetaten. Melding om flytting fra Norge [Available from: https://www.skatteetaten.no/person/folkeregister/flytte/fra-norge/.

For peer review only

51. Ludvigsson JF, Almqvist C, Bonamy A-KE, Ljung R, Michaëlsson K, Neovius M, et al. Registers of the Swedish total population and their use in medical research. European journal of epidemiology. 2016;31(2):125-36.

52. Digital and Population Data Services Agency. Moving to Finland or moving abroad from Finland 2022 [Available from: https://dvv.fi/en/moving-abroad-or-from-abroad-to-finland.

53. Holt N, Mygind A, Bro F. Danish MMR vaccination coverage is considerably higher than reported. Danish medical journal. 2017;64(2).

54. Remschmidt C, Wichmann O, Harder T. Frequency and impact of confounding by indication and healthy vaccinee bias in observational studies assessing influenza vaccine effectiveness: a systematic review. BMC Infect Dis. 2015;15:429.

For peer review only

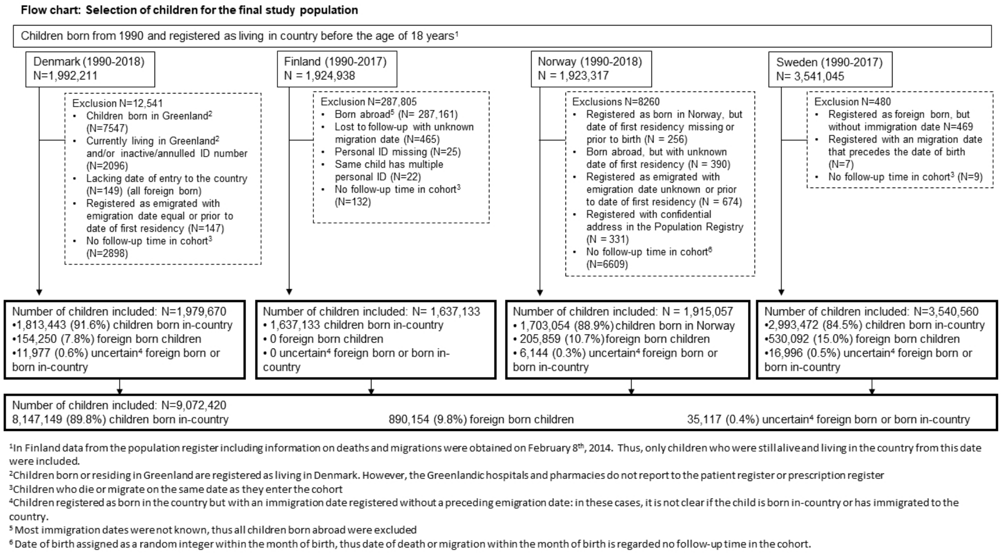

Figure 1: Flowchart of study population in Denmark, Finland, Norway, and Sweden

855x481mm (38 x 38 DPI)

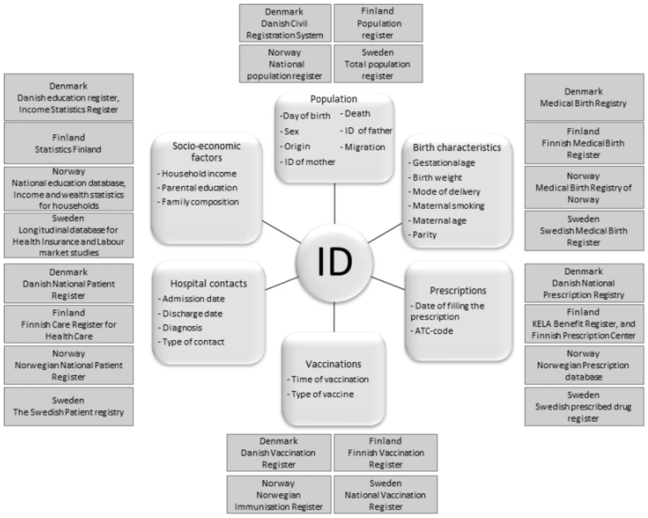

Figure 2: Nordic register information linked to the individual using a unique personal identification code (ID)

855x481mm (38 x 38 DPI)

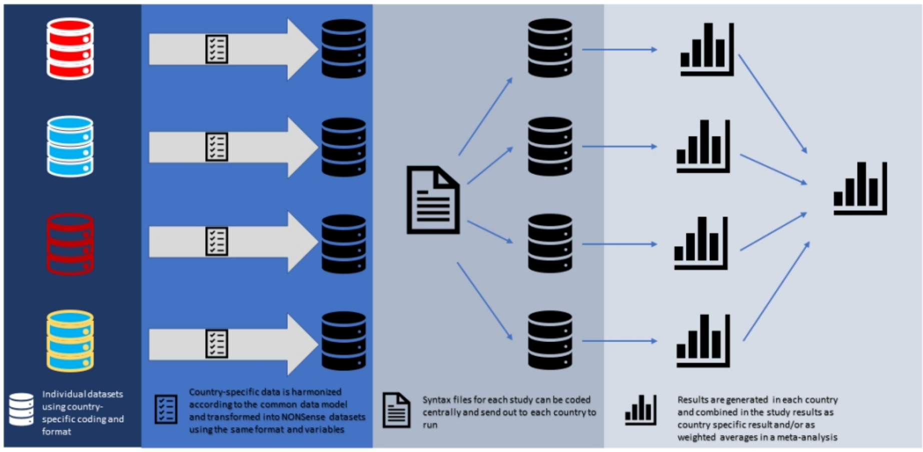

Figure 3: Transforming country specific datasets into NONSense datasets using a common data model

855x481mm (38 x 38 DPI)

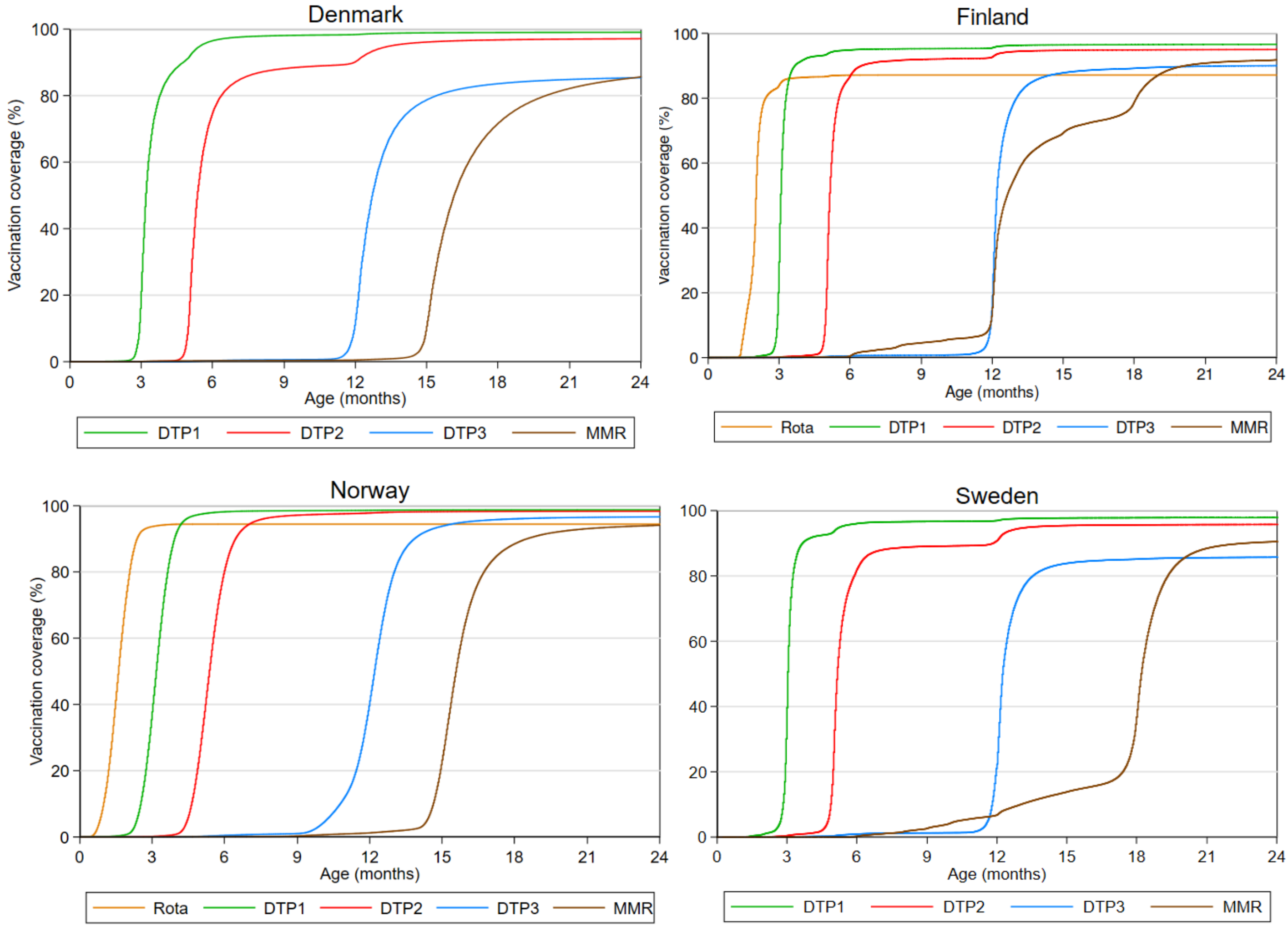
Denmark
Vaccination coverage (%)
Age (months)
DTP1
DTP2
DTP3
MMR
Finland
Vaccination coverage (%)
Age (months)
Rota
DTP1
DTP2
DTP3
MMR
Norway
Vaccination coverage (%)
Age (months)
Rota
DTP1
DTP2
DTP3
MMR
Sweden
Age (months)
DTP1
DTP2
DTP3
MMR

only

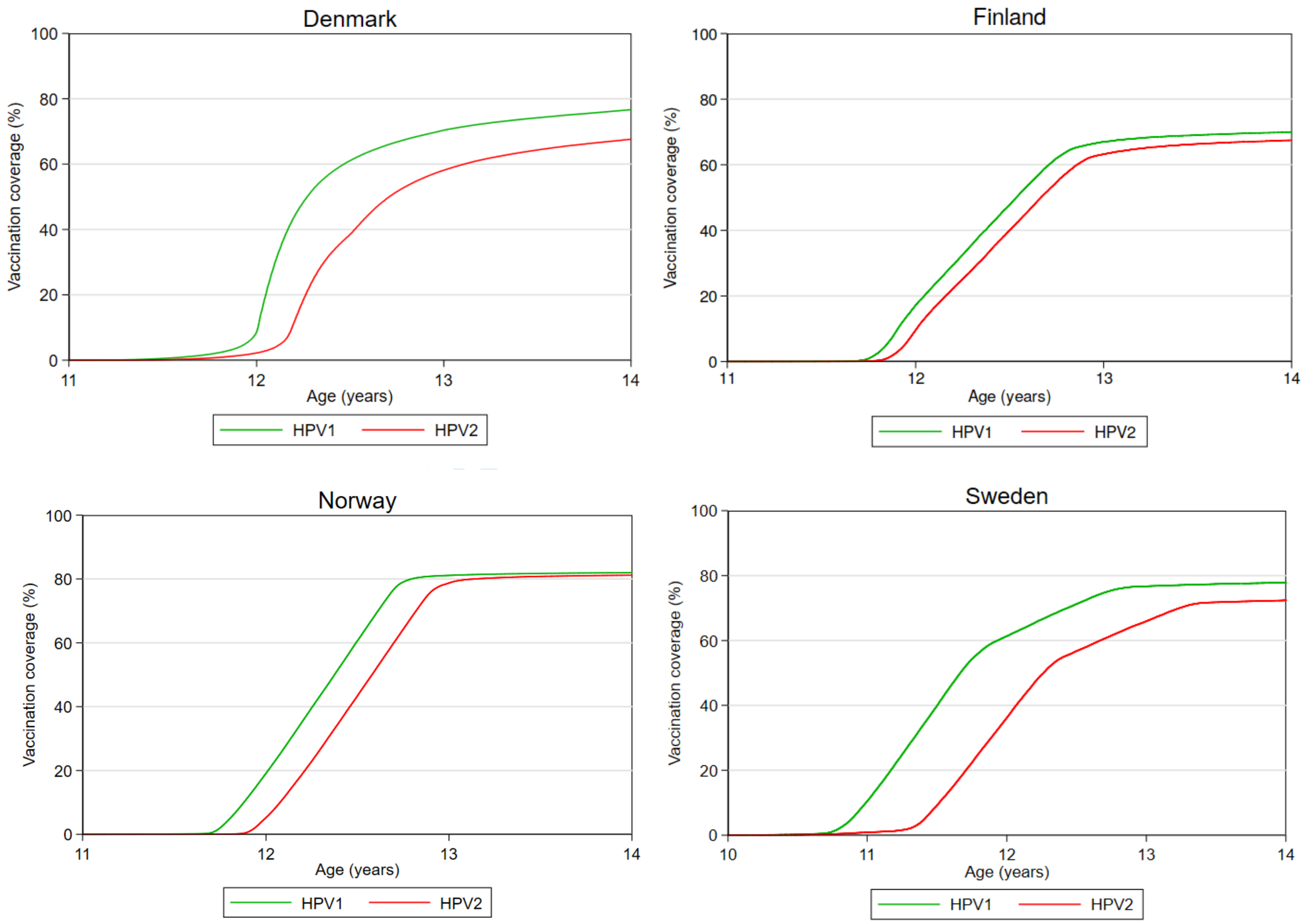
Denmark
Finland
Norway
Sweden
Vaccination coverage (%)
Age (years)
HPV1
HPV2
0
20
40
60
80
100
10
11
12
13
14

Lexis diagram for Danish cohort including vaccination programme and data availability

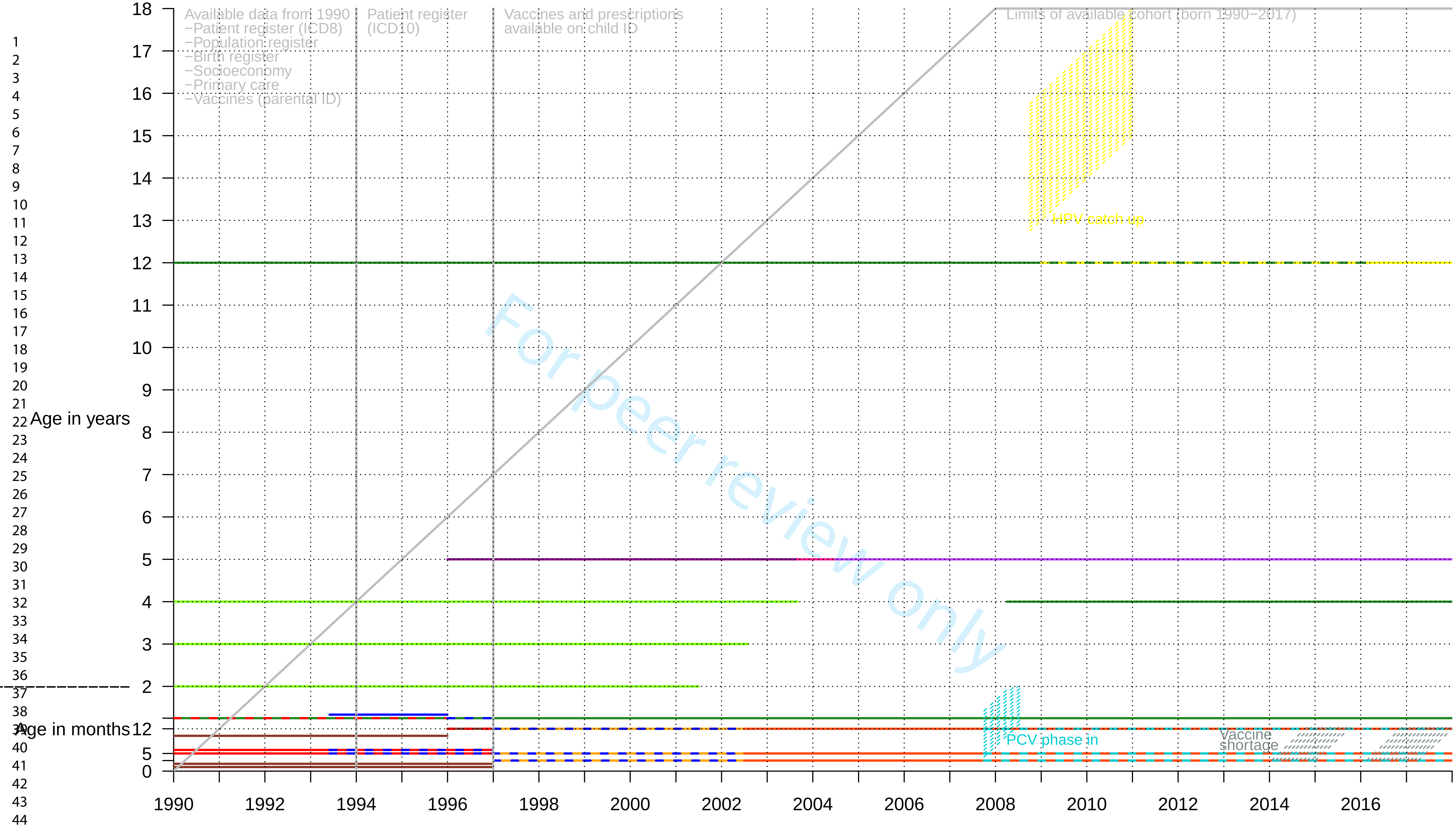

Gray text and gray lines indicate data availability

Color codes for vaccines:

wP; DT−IPV; DTaP−IPV; DTaP−IPV−Hib; Hib; PCV; MMR; OPV; DT; DTaP; DTaP−IPV; HPV

Abbreviations for vaccines:

Non−live vaccines: wP=whole cell pertussis; D=diphtheria; T=tetanus; IPV=inactivated polio vaccine; aP=pertussis vaccine(acellular);
Hib=Haemophilus influenzae type b; PCV=pneumococcal conjugate vaccine; HPV=Human papilloma virus
Live vaccines: MMR=measles−mumps−rubella; OPV=oral polio vaccine

For peer review only

BMJ Open

Lexis diagram for Finnish cohort including vaccination programme and data availability

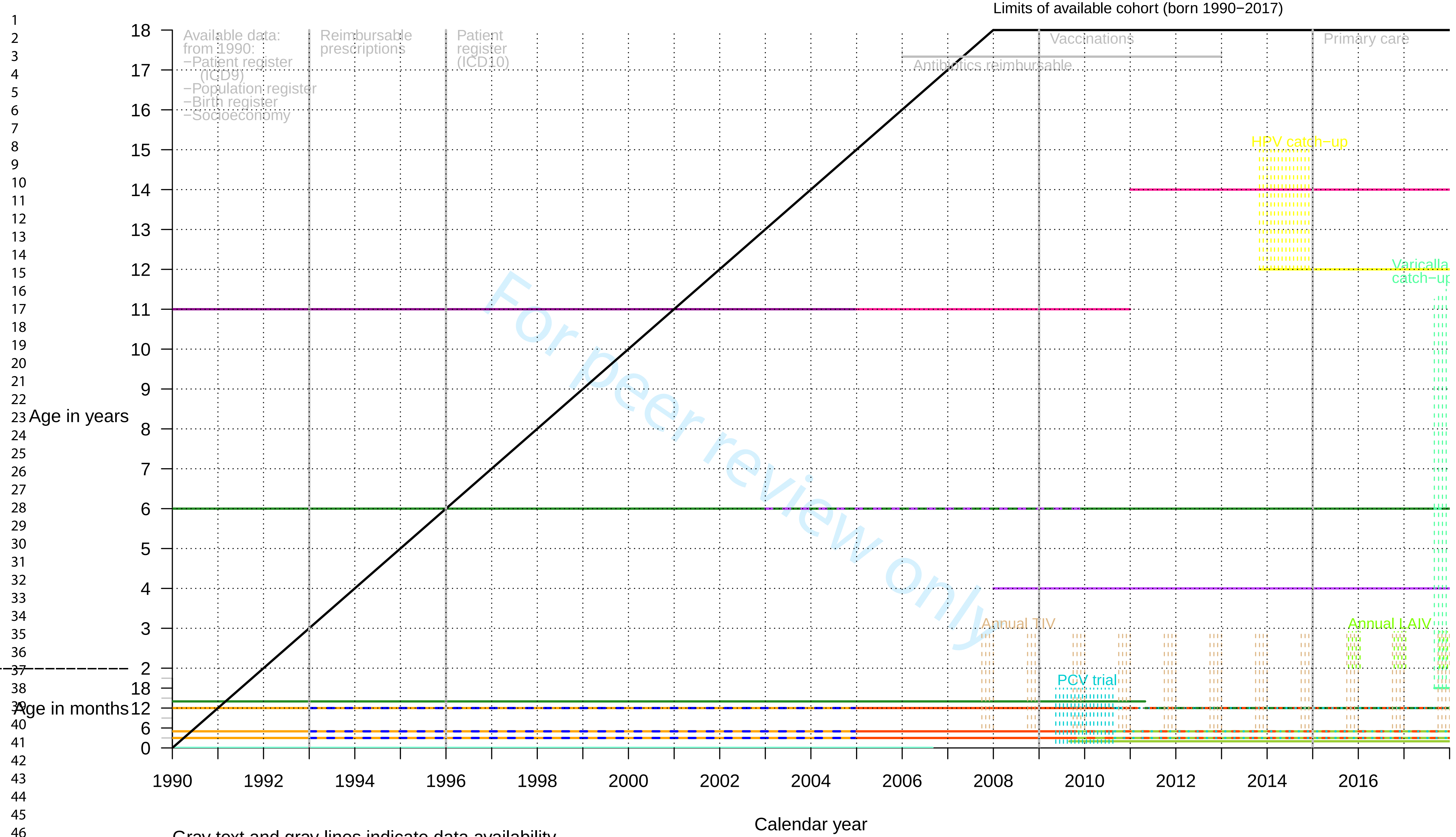

Gray text and gray lines indicate data availability

Color codes for vaccines:

BCG; RV; DTaP−IPV; Hib; DTaP−IPV−Hib; PCV; MMR; V; DTaP−IPV−booster; DT−booster; DTaP−booster; HPV

Abbreviations for vaccines:

Non−live vaccines: D=diphtheria; T=tetanus; aP=pertussis vaccine(acellular); IPV=inactivated polio vaccine; Hib=Haemophilus influenzae type b; PCV=pneumococcal conjugate vaccine; TIV=trivalent influenza vaccine; HPV=Human papilloma virus

Live vaccines: BCG=Bacille Calmette−Guerin; RV=Rotavirus; MMR=measles−mumps−rubella; V=varicella; LAIV=live attenuated influenza vaccine

For peer review only

For peer review only - http://bmjopen.bmj.com/site/about/guidelines.xhtml

Lexis diagram for Norwegian cohort including vaccination programme and data availability

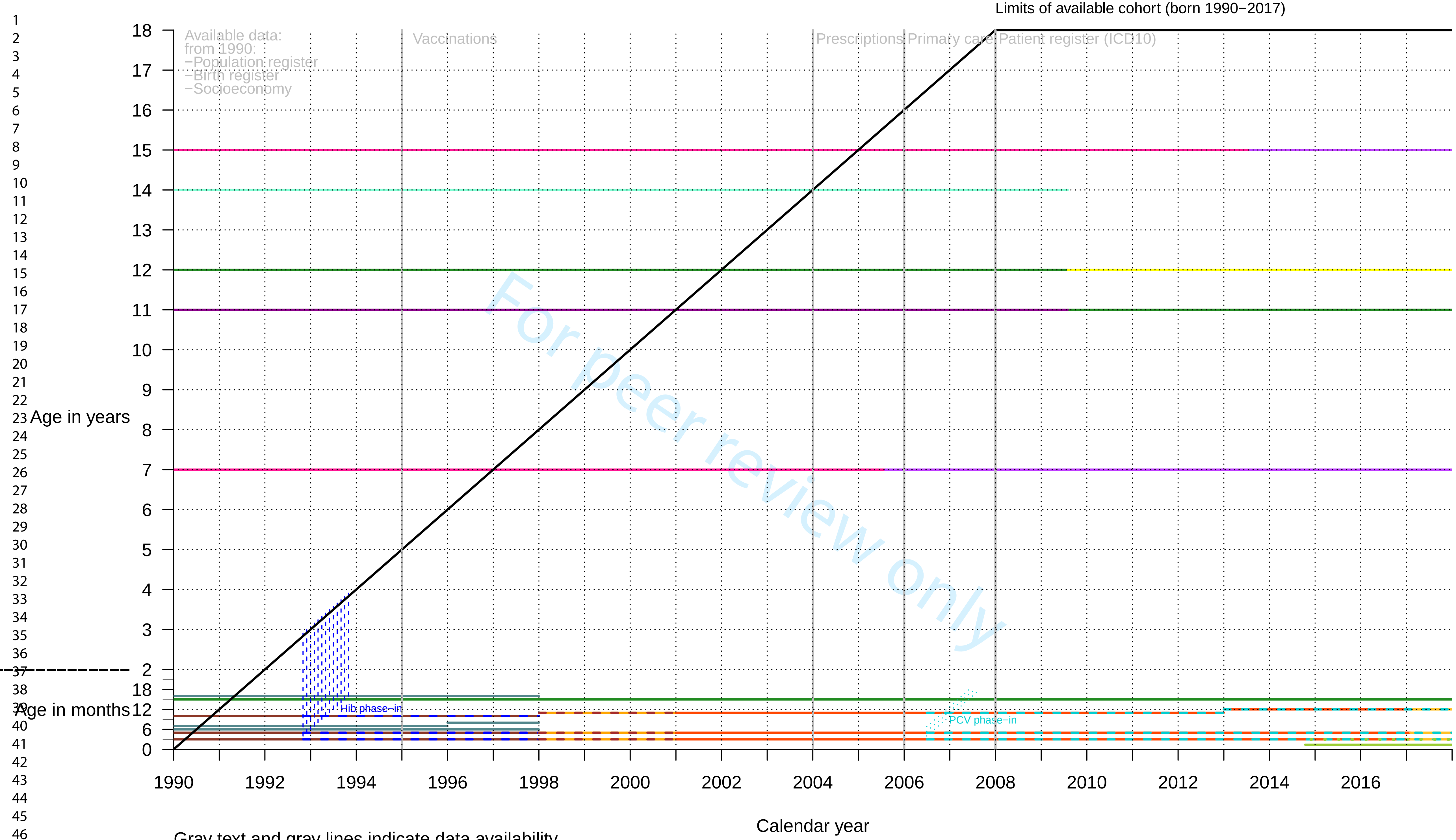

For peer review only

Gray text and gray lines indicate data availability
Color codes for vaccines:
RV; DTwP; Hib; DTaP; IPV−Hib; DTaP−IPV−Hib; PCV; DTaP−IPV−Hib−HepB; IPV; MMR; IPV−booster; DTaP−IPV−booster; DT−booster; HPV; BCG

Abbreviations for vaccines:
Non−live vaccines: D=diphtheria; T=tetanus; wP=whole−cell pertussis vaccine; Hib=Haemophilus influenzae type b;aP=pertussis vaccine(acellular);
IPV=inactivated polio vaccine; PCV=pneumococcal conjugate vaccine; HepB=Hepatitis B; HPV=Human papilloma virus
Live vaccines: RV=Rotavirus; MMR=measles−mumps−rubella; BCG=Bacille Calmette−Guerin

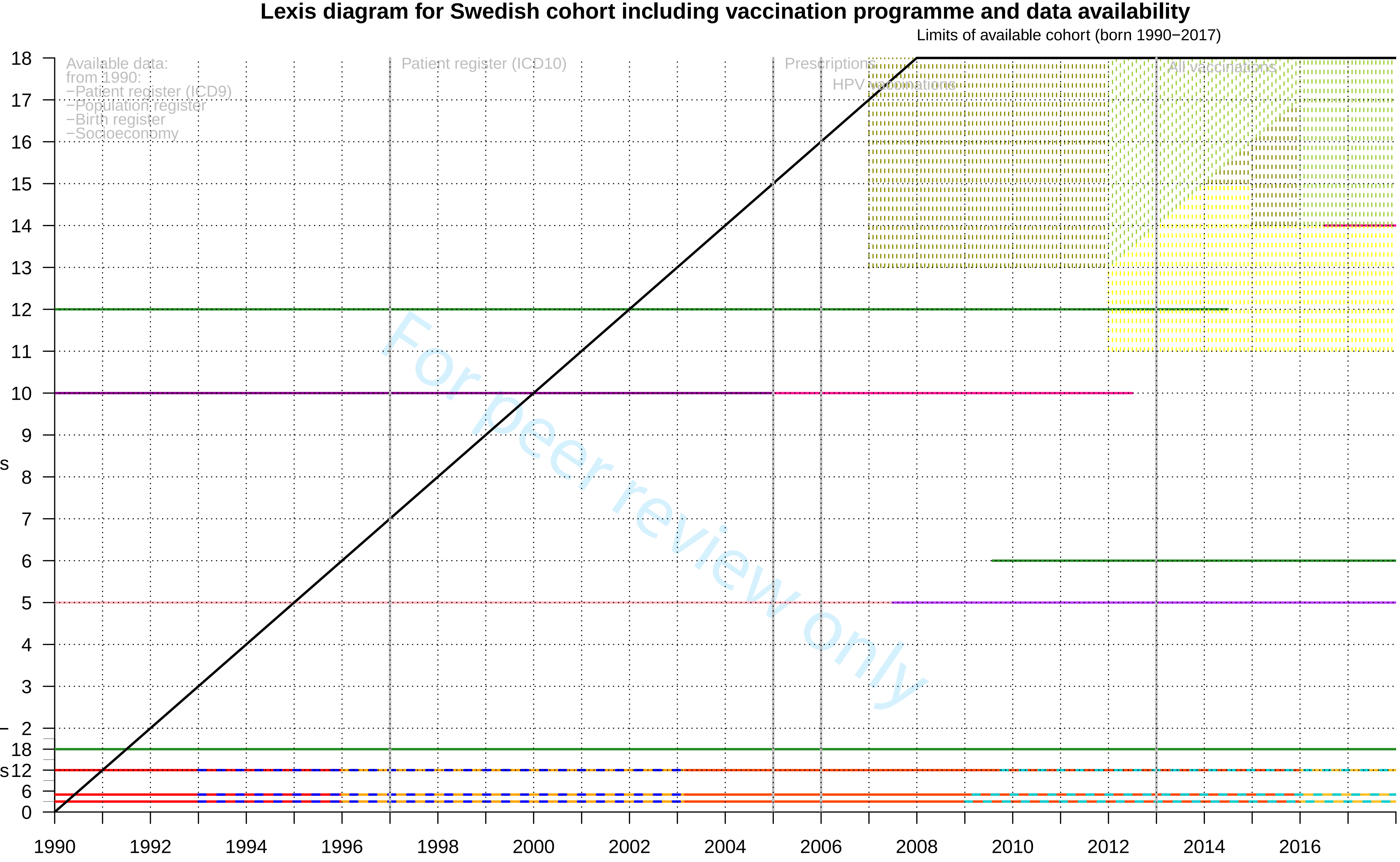

Gray text and gray lines indicate data availability

Color codes for vaccines:

DT−IPV; Hib; DTaP−IPV; DTaP−IPV−Hib; PCV; DTaP−IPV−Hib−HepB; MMR; IPV−booster ; DTaP−IPV−booster ; DT−booster; DTaP−booster; HPV−recommended age ; HPV−own payment with partly subsidy ; HPV catch−up

Abbreviations for vaccines:

Non−live vaccines: D=diphtheria; T=tetanus; aP=pertussis vaccine(acellular); IPV=inactivated polio vaccine; Hib=Haemophilus influenzae type b; PCV=pneumococcal conjugate vaccine; HepB=Hepatitis B; HPV=Human papilloma virus

Live vaccines: MMR=measles−mumps−rubella

For peer review only - http://bmjopen.bmj.com/site/about/guidelines.xhtml

Link to Contents

NONSense Common Data Model

May 18th 2022

For peer review only

Link to Contents

For peer review only - http://bmjopen.bmj.com/site/about/guidelines.xhtml

Link to Contents

Contents

Contents 2
Introduction 4
Background/Event tables 5
Table: prescriptions 5
Table: hospital_contacts 5
Table: population1 6
Table: birth_characteristics 7
Table: Vaccines 8
Table: socio_economy 9
Source of data in each country 12
Table: prescriptions 12
Prescriptions Denmark 12
Prescriptions Finland 12
Prescriptions Norway 14
Prescriptions Sweden 14
Table: hospital_contacts 15
Hospital contacts Denmark 15
Hospital contacts Finland 17
Hospital contacts Norway 18
Hospital contacts Sweden 19
Table: population1 21
Population 1 Denmark 21
Population 1 Finland 24
Population 1 Norway 26
Population 1 Sweden 29
Table: birth_charcteristics 31
birth_characteristics Denmark 31
birth_characteristics Finland 33
birth_characteristics Norway 34
Birth characteristics Sweden 35
Table: Vaccines 37
Vaccines Denmark 37

Link to Contents

For peer review only

For peer review only - http://bmjopen.bmj.com/site/about/guidelines.xhtml

Link to Contents

Vaccines Finland 38
Vaccines Norway 39
Vaccines Sweden 39
Table: socio_economy 42
Socio_economy Denmark 42
Socio_economy Finland 44
Socio_economy Norway 45
Socio_economy Sweden 49
Appendix: Vaccine categorization: 51

For peer review only

Link to Contents

For peer review only - http://bmjopen.bmj.com/site/about/guidelines.xhtml

Link to *Contents*

Introduction

The Common Data Model (CDM) is a tool for documentation of data preparation and generation of uniform datasets across the Nordic countries (Denmark, Finland, Norway, and Sweden). The aim is to construct a number of uniform background datasets and event tables, which share the same name and entail the same variables, labels and values across countries. Datasets with the exact same format across Countries enables sharing of syntax-files for study analyses.
The CDM is a working document, which will be updated according to country specific data preparation, and expanded as all necessary information will be transformed into background/event tables. In the end, the background/event tables will include all necessary information to conduct all future studies (morbidity/incidence studies and vaccination studies).

The current version presents data content and preparation as per April 2022

The CDM contains 1) "Background/Event tables", and 2) "Source of data and data preparation in each country".

Background/Event tables: include information on the name of the dataset to be used by NONSEnse and format and labeling of each variable within the dataset.

Source of data in each country: includes a description of the information on the source register, and source variables, which have been used to generate the variables in the background/event tables. These tables furthermore entail information on important notes (i.e data breaks, limitations such as i.e. restricted information on redeemed prescriptions in Finland) and data preparation (how have the source variables been modified to generate the variables in the background/event tables). The tables on source of data in each country have been filled in by the individual countries following country specific data preparation.

Link to *Contents*

For peer review only - http://bmjopen.bmj.com/site/about/guidelines.xhtml

Link to Contents

Background/Event tables

Table: prescriptions

Description: Table of all redeemed prescriptions (included atc codes in each country is listed in "source of data and datapreparation") among individuals in the study population.
Structure: 1 observation (line) for every redeemed pharmaceutical.

Variables:

Variable	Label	values
id	Personal id of the child	string
b_date	Birthdate of the child	Date Format (%dD_m_Y)
sex	Sex of the child as recorded in the dataset	1="male"<br>2="female"
redeemdate	Date of redeeming the prescription	Date Format (%dD_m_Y)
atc	Full atc code for the redeemed drug	String (7 digits) use capital letters i.e "J01AA01"

Table: hospital_contacts

Description: Table of all diagnoses (both main diagnosis and all other diagnoses) for somatic patients including information on sex and date of birth for all children in the study population. Note that a patient can have multiple diagnoses attached to the same contact.
Structure: 1 observation (line) for each diagnosis received

Variables:

Variable	label	values
id	Personal id of the child	String
b_date	Birthdate of the child	Date Format (%dD_m_Y)
sex	Sex of the child as recorded in the dataset	1="male"<br>2="female"
adm_date	Date of admission	Date Format (%dD_m_Y)
discharge_date	Date of discharge	Date Format (%dD_m_Y)
diag	ICD diagnosis code	String (For ICD-10 codes use max 4 digits e.g. A063)
diagtype	Type of diagnosis	1="Main diagnosis"<br>2="Other diagnosis"
type_contact	Type of hospital contact	Categorical:<br>1="inpatient"<br>2="emergency room patient"<br>3="outpatient"<br>4="outpatient or emergency room patient"

Link to Contents

For peer review only - http://bmjopen.bmj.com/site/about/guidelines.xhtml

Link to Contents

Table: population1

Description: Background table including information follow-up for each child in the study population. The dataset only includes information on the child's first stay in the country (first in_date and first cens_date is recorded).

Structure: one line for each child

Variable	label	values
id	Personal id of the child	string
b_date	Birthdate of the child	Date Format (%dD_m_Y)
sex	Sex of the child as recorded in the dataset	1="male"<br>2="female"
origin	Born in the country or abroad	1="born in-country"<br>2="born abroad"<br>9= "Unknown"
in_date	Date of entering the cohort	Date Format (%dD_m_Y)
in_reason	Reason for entering the cohort	1="birth"<br>2="immigration"
cens_date	First date of censoring	Date Format (%dD_m_Y)
cens_reason	Reason for being censored	1="death"<br>2="out migration"<br>3="other"
m_id	id of mother	string
f_id	id of father	string
m_age	Mothers age in years at time of delivery	Numeric (discrete)
m_origin	Maternal origin at birth	1="born in-country"<br>2="born abroad"<br>9= "Unknown"
p_origin	Paternal origin at birth	1="born in-country"<br>2="born abroad"<br>9= "Unknown"

Link to Contents

For peer review only - http://bmjopen.bmj.com/site/about/guidelines.xhtml

Link to Contents

Table: birth_characteristics

Structure: one line for each child in the study population

variable	Label	values	Legal values
id	Personal id of the child	string	
b_weight	Birthweight of child (gram)	Numeric	100-9990
ga	Gestational age (full weeks)	Numeric (discrete)	
sectio	Delivered by caesarean section	0=”not delivered by caesarean section”<br>1=”delivered by caesarean section”<br>9=” unknown”	
smoke	Maternal smoking or snuff at any point during pregnancy	0= ”no”<br>1= “smoking (or snuff) during pregnancy”<br>9= “unknown”	
singleton	singleton	0=”no”<br>1=”yes”<br>9=” unknown”	
child_order	Child order (including the child itself)	Numeric (discrete)	

For peer review only

Link to Contents

For peer review only - http://bmjopen.bmj.com/site/about/guidelines.xhtml

Link to Contents

Table: Vaccines

variable	Label	values
id	Personal id of the child	string
vacdate	Date of vaccination	Date Format (%dD_m_Y)
vaccine	Type of vaccine administered	Categorical (see coding in appendix ”vaccine categorization”)
credibility	Credibility indication of vaccine information	1=no duplicate<br>2= duplicate same vaccine removed<br>3=duplicate related vaccine removed (keep vaccine that aligns with vaccination schedule)<br>4= duplicate related vaccine removed (none of the vaccines align with vaccination schedule)<br>5= duplicate related vaccine removed (vaccines given outside the vaccination schedule)
TB_endemic	Vaccine recommendations in accordance with connections to TB endemic countries	0=not risk group<br>1=risk group<br>9=not relevant
HepB_endemic	Vaccination recommendations in accordance with connections to HepB endemic countries	0=not risk group<br>1=risk group<br>9=not relevant

Prioritization for duplicate selection:

1. Remove same vaccines (variable name: “vaccine”, see appendix “vaccine categorization”) given 14 days or less after the previous vaccine for the same child (if DTP is registered on day 0, 10 and 20, only remove the vaccine registered at day 10) – *keep the earliest registration*
    i. *Credibility=2*
2. Remove vaccines from the same type of vaccines (variable name: “type” see appendix “vaccine categorization”) given 14 days or less after the previous vaccine of the same type. Register vaccine as given on the earliest date within the duplicate combination **prioritize within combinations:**
    a. Keep vaccine that aligns with vaccination schedule according to ***age*** and ***year of vaccination***
        i. Credibility =3
    b. If no vaccine aligns with vaccination schedule but type and age correspond to timing of childhood vaccinations keep the vaccine that protects against most conditions
        i. Credibility=4
    c. If vaccines are given outside ages for recommended vaccination according to the vaccination program – keep the vaccine that protects against most conditions
        i. Credibility=5

Link to Contents

For peer review only - http://bmjopen.bmj.com/site/about/guidelines.xhtml

Link to Contents

Table: socio_economy

Assign information to all children in the study population. If a child has no registrations in the socio economic datasets variables should be coded as 9 or 99="unknown" as described in the table below.

Overall note on timing of information:

Variables ending with "_b" indicate that information is from birth of the child. Depending on the set up of the register information we will use the date or year of birth to obtain the information. If information is not available for the date or year of birth, we will use information from the year after.

Variables ending with "_10y" indicate that information is from the year/date the child turns 10 years. Depending on the set up of the register information we will use the date or year of turning 10 years to obtain the information.

variable	Label	values	Legal values	Notes
id	Personal id of the child	string		
inc_quin_b	Household income quintile at year of birth of the child	1=first (lowest)<br>2=second<br>3= third<br>4= fourth<br>5= fifth (highest)<br>9="unknown"		Quintiles are calculated stratified on year (i.e., calculation of quintiles are done separately for each calendar year.<br>If several income variables are available, selection is based on this priority: 1: equated disposable household/family income; 2: disposable household/family income; 3:household/family income; 4: maternal disposable income; 5: maternal income.
inc_quin_10y	Household income quintile at the year of the child's 10th birthday	1=first (lowest)<br>2=second<br>3= third<br>4= fourth<br>5= fifth (highest)<br>9="unknown"		See notes under inc_quin_b
inc_quin_m_b	Maternal income quintile at year of birth of the child	1=first (lowest)<br>2=second<br>3= third<br>4= fourth		See notes under inc_quin_b

Link to Contents

For peer review only - http://bmjopen.bmj.com/site/about/guidelines.xhtml

Link to Contents

		5= fifth (highest)<br>9="unknown"		
inc_quin_m_10y	Maternal income quintile at the year of the child's 10th birthday	1=first (lowest)<br>2=second<br>3= third<br>4= fourth<br>5= fifth (highest)<br>9="unknown"		See notes under inc_quin_b
n_children_b	Number of children below 18 years in the household including the child itself at year of birth of the child	Numeric discrete<br><br>99="unknown"	>=1	
n_children_10y	Number of children below 18 years in the household including the child itself at the year of the child's 10th birthday	Numeric discrete<br><br>99="unknown"	>=1	
single_parent_b	Single parenthood at year of birth of the child	0=No<br>1=Yes<br>9="unknown"		
single_parent_10y	Single parenthood at the year of the child's 10th birthday	0=No<br>1=Yes<br>9="unknown"		
m_education_b	Maternal highest attained education at year of birth of the child	1=Low education (ISCED2011 level 0-2)<br>2=Medium education (ISCED2011 level 3-4)<br>3=High education (ISCED2011 level 5-8)<br>9="unknown"		International Standard Classification of Education (ISCED) 2011 coded into main groups. Read more in reference 1 below the table.
m_education_10y	Maternal highest attained education at	1=Low education (ISCED2011 level 0-2)		See notes under m_education_b.

For peer review only

Link to Contents

	the year of the child's 10th birthday	2=Medium education (ISCED2011 level 3-4)<br>3=High education (ISCED2011 level 5-8)<br>9="unknown"		

Reference 1 for ISCED: https://ec.europa.eu/eurostat/statistics-explained/index.php/International_Standard_Classification_of_Education_(ISCED)#Implementation_of_ISCED_2011_.28levels_of_education.29

For peer review only

Link to Contents

For peer review only - http://bmjopen.bmj.com/site/about/guidelines.xhtml

Link to Contents

Source of data in each country

Table: prescriptions

Prescriptions Denmark

Variable	Source and description	Important notes and data preparation
id	Original name in the Danish data: “pnr”. Pseudonomised unique personal identification number for linkage between registers, created by Statistics Denmark. It is linkable (by Statistics Denmark) to the original personal identification number (CPR number) assigned to all Danish residents and used when reporting to all national registers.	Renamed from “pnr”
b_date	Obtained from the Danish National Health Data Agency.<br>Register: “CPR-Registret”<br>Table: “t_person”<br>Variable: “d_foddato”	Renamed from “d_foddato”
sex	Obtained from the Danish National Health Data Agency.<br>Register: “CPR-Registret”<br>Table: “t_person”<br>Variable: “C_KON”<br>Sex as recorded by personal identification number.	sex=1 “male” if C_KON is “M”<br>sex=2 “female” if C_KON is “K”
redeemdate	Obtained from Statistics Denmark.<br>Register: “Lægemiddeldatabasen”<br>Variable: “EKSD”<br>Date of redeeming the prescription	Renamed from “EKSD”
atc	Obtained from statistics Denmark.<br>Register: “Lægemiddeldatabasen”<br>Variable: “ATC”<br>ATC code of purchased drug	All prescriptions with ATC group D, J, R, S and V01, including all sublevels.<br>Renamed from “ATC”

Prescriptions Finland

Variable	Source and description	Important notes and data preparation
id	Obtained from KELA<br>Register: “KELA etuusrekisteri”,<br>Table:”Lääkeostot”,<br>Variable: ”HETU”<br><br>Register: ”Kanta Reseptikeskus”<br>Table:<br>”KANTA:RESEPTI.LAAKETOIMITUKSET”<br>Variable: “PATIENT_ID”	Statistics Finland pseudonymised HETU and PATIENT_ID

For peer review only - http://bmjopen.bmj.com/site/about/guidelines.xhtml

Link to Contents

	THL pseudonymised the original personal identification code (in these registers HETU and PATIENT_ID) to unique personal identification number for linkage between registers. THL data management can link the id back to original personal identification code.	
b_date	Obtained from KELA<br>Obtained from KELA<br>Register: " KELA etuusrekisteri",<br>Table:"Lääkeostot",<br>Variable: "HETU"<br><br>Register: "Kanta Reseptikeskus"<br>Table:<br>"KANTA:RESEPTI.LAAKETOIMITUKSET"<br>Variable: "PATIENT_ID"	Extracted from "HETU" before pseudonymisation was done.<br><br><br><br>Extracted from "PATIENT_ID" before pseudonymisation was done.
sex	Obtained from KELA<br>Register: " KELA etuusrekisteri",<br>Table:"Lääkeostot",<br>Variable: "HETU"<br><br>Register: "Kanta Reseptikeskus"<br>Table:<br>"KANTA:RESEPTI.LAAKETOIMITUKSET"<br>Variable: "PATIENT_ID"	Extracted from "HETU" and "PATIENT_ID" before pseudonymisation was done.<br>sex=1 "male"<br>sex=2 "female"
redeemdate	Obtained from KELA<br>Register: " KELA etuusrekisteri",<br>Table:"Lääkeostot",<br>Variable: "OSTOPV"<br><br>Register: "Kanta Reseptikeskus"<br>Table:<br>"KANTA:RESEPTI.LAAKETOIMITUKSET"<br>Variable: "CREATION_DATE"<br>Date of redeeming the prescription	Renamed from "OSTOPV"<br>Renamed from "CREATION_DATE"
atc	Obtained from KELA<br>Register: " KELA etuusrekisteri"<br>Table:" Lääkeostot"<br>Variable: "ATC"<br><br>Register: "Kanta Reseptikeskus"<br>Table:<br>"KANTA:RESEPTI.LAAKETOIMITUKSET"<br>Variable: "ATC_CODE"<br><br><br>ATC code of purchased drug	All prescriptions with ATC groups D07, D11AH, J, R01, R03, R06, S01G, S03 and V01, including all sublevels. V01 only from KELA data.<br>In Korvattavat lääkkeet only reimbursable products.<br>Reimbursement of antibiotics:<br>< 2006 no reimbursement if cheap<br>2006-2012: all antibiotics were reimbursed<br>>2012: individual products not reimbursed"<br><br>Duplicates removed: if same purchase (same id, redeemdate and atc) was found from both registers only one of them was included in the data.

Link to Contents

For peer review only - http://bmjopen.bmj.com/site/about/guidelines.xhtml

Link to Contents

Prescriptions Norway

Variable	Source and description	Important notes and data preparation
id	Obtained from<br>Register: “The Norwegian Prescription Database” (NorPD)<br>Pseudonomised unique personal identification number for linkage between registers	Renamed from “pasient_lopenr_pdb2471”
b_date	Obtained from The National Population Register	We have received information on month and year of birth, but not day. For each individual, we have therefore generated a random integer between 1 and length of their month of birth. Using this random integer as day of birth, everyone is assigned an exact birth date.
sex	Obtained from The National Population Register.	Renamed from “kjonn”
redeemdate	Obtained from<br>Register: “NorPD”<br>Variable: “UtleveringsDato”<br>Date of redeeming the prescription	Renamed from “UtleveringsDato”
atc	Obtained from<br>Register: “NorPD”<br>Variable: “ATCKode”<br>ATC code of purchased drug	All prescriptions with ATC group D, J, R, S and V01, including all sublevels<br>Renamed from “ATCKode”

Prescriptions Sweden

Variable	Source and description	Important notes and data preparation
id	Created by Statistics Sweden<br>Pseudonomised unique personal identification number for linkage between registers	Renamed from lopnr
b_date	Obtained from Statistics Sweden<br>Register: “Register över totalbefolkningen, RTB”<br>Variable: “fodddatum”	Renamed from fodddatum
sex	Obtained from Statistics Sweden<br>Register: “RTB”<br>Variable: “kon”	Renamed from “kon”
redeemdate	Obtained from Socialstyrelsen<br>Register: “Läkemedelsregisteret”<br>Variable: “edatum”	Renamed from “edatum”.<br>(Date of redeeming the prescription)
atc	Obtained from Socialstyrelsen<br>Register: “Läkemedelsregisteret”<br>Variable: “atc”	ATC code of purchased drug. The data from Sweden included all prescriptions within ATC

Link to Contents

For peer review only - http://bmjopen.bmj.com/site/about/guidelines.xhtml

Link to Contents

		groups D, J, R, S and V01, including all sublevels

Table: hospital_contacts

Hospital contacts Denmark

Variable	Source and Description	Important notes and data preparation
id	Original name in the Danish data: "pnr". Pseudonomised unique personal identification number for linkage between registers, created by Statistics Denmark. It is linkable (by Statistics Denmark) to the original personal identification number (CPR number) assigned to all Danish residents and used when reporting to all national registers.	Renamed from "pnr"
b_date	Obtained from the Danish National Health Data Agency.<br>Register: "CPR-Registret"<br>Table: "t_person"<br>Variable: "d_foddato"	Renamed from "d_foddato"
sex	Obtained from the Danish National Health Data Agency.<br>Register: "CPR-Registret"<br>Table: "t_person"<br>Variable: "C_KON"<br>Sex as recorded by personal identification number.	sex=1 "male" if C_KON is "M"<br>sex=2 "female" if C_KON is "K"
adm_date	Obtained from the Danish National Health Data Agency.<br>Register: Danish national patient registry<br>Table: T_ADM<br>Variable: D_INDDTO	Renamed from "D_INDDTO"<br><br>For outpatient contacts with multiple visits adm_date is recoded according to the date of visit ("D_AMBDTO" from the table "t_bes")
discharge_date	Obtained from the Danish National Health Data Agency.<br>Register: Danish national patient registry<br>Table: T_ADM<br>Variable: D_UDDTO	Renamed from "D_UDDTO"<br><br>For contacts without a discharge date (N=1080) the discharge date is set as the last observed discharge date in the dataset+1day (11May2018)<br><br>For outpatient contacts, discharge date is recoded to be the same date as "adm_date".
diag	Obtained from the Danish National Health Data Agency.	Renamed from "C_DIAG"

Link to Contents

For peer review only - http://bmjopen.bmj.com/site/about/guidelines.xhtml

Link to Contents

	Register: Danish national patient registry<br>Table: T_DIAG<br>Variable: C_DIAG	Diagnosis coded as ICD 8 until December 31 1994, hereafter coded using ICD 10.<br><br>Danish specification letters to the ICD-10 codes removed and the administrative letter "D" in front of all codes removed: Values changed to string4 format (i.e DA011a→A011)<br><br>Diagnoses other than the main or other diagnoses are excluded.<br>Diagnoses with modifications indicating that the diagnosis cannot be validated are excluded (c_diagmod==1 | 2).
diagtype	Obtained from the Danish National Health Data Agency.<br>Register: Danish national patient registry<br>Table: T_DIAG<br>Variable: C_DIAGTYPE	Renamed from variable "C_DIAGTYPE"<br>Recoded: C_DIAGTYPE: "A"= "main diagnosis" C_DIAGTYPE: "B"= "other diagnosis"<br>A patient can have multiple other diagnoses for the same contact.<br>Excluding diagnoses other than main or other (i.e temporary diagnoses or additional diagnosis ("tillægsdiagnose").
type_contact	Obtained from the Danish National Health Data Agency.<br>Register: Danish national patient registry<br>Table: T_DIAG and t_bes<br>Variables: C_PATTYPE, D_AMBDTO	Renamed variable "C_PATTYPE"<br><br>Recoded:<br>type_contact=1 "inpatient" if C_PATTYPE is "0" (inpatient) or "1" (Before year 2002 some patients were coded as "1= deldøgnspatienter" (≈part day patient)<br><br>type_contact=2 "emergency room contact" if C_PATTYPE is 3 "emergency room contact". Outpatient contacts (C_PATTYPE=2) admitted after year 2014 with "C_INDM"= "Acute" are coded as type_contact=2 "emergency room patient"<br><br>type_contact=3 "outpatient contact" if C_PATTYPE=2 before year 2014 or C_PATTYPE=2 and c_indm is not 1 from and including year 2014<br><br>In Denmark we have some long outpatient contacts with multiple visit dates (D_AMBDTO) during the contact. Each visit date is coded as an independent outpatient contact. All diagnoses within the original outpatient contact is recorded for each visit.

Link to Contents

For peer review only - http://bmjopen.bmj.com/site/about/guidelines.xhtml

Link to Contents

Hospital contacts Finland

Variable	Source and Description	Important notes and data preparation
id	Obtained from THL<br>Register: “the Finnish National Patient Register THL=Hilmo”<br>Table: “Perustiedot/Asiakas, potilas”<br>Variable: “HT”<br>THL pseudonymised the original personal identification code (in this register HT) to unique personal identification number for linkage between registers. THL data management can link the id back to original personal identification code.	Statistics Finland pseudonymised HT with their own id for the remote user system.
b_date	Obtained from THL<br>Register: “Hilmo”<br>Table: “”<br>Variable: “SYNTAIKA”	Renamed from “SYNTAIKA”
sex	Obtained from THL<br>Register: “Hilmo”<br>Table: “Perustiedot/Asiakas, potilas”<br>Variable: “SP”	Renamed from “SP”
adm_date	Obtained from THL<br>Register: “Hilmo”<br>Table: “Tulotiedot”<br>Variable: “”TUPVA”	Extracted from “TUPVA” which contain the date and time of arrival
discharge_date	Obtained from THL<br>Register: “Hilmo”<br>Table: “Poistumistiedot”<br>Variable: “LPVM”	Extracted from “LPVM” which contain the date and time of discharge
diag	Obtained from THL<br>Register: “Hilmo”<br>Table: “Hoitotiedot”<br>Variable: “PDGO, PDGE, SDGO, SDGE”	Renamed from PDGO, PDGE, SDGO, SDGE ICD-codes<br>ICD-codes V01-Y98 not available, codes O00-O99 were not analysed
diagtype	Obtained from THL<br>Register: “Hilmo”<br>Table: “Hoitotiedot”<br>Variable: “PDGO, PDGE, SDGO, SDGE”	1=main diagnosis: PDGO and PDGE<br>2=add diagnosis: SDGO and SDGE
type_contact	Obtained from THL<br>Register: “Hilmo”<br>Table: “Perustiedot/Hoitojakso tai avohoitokäynti”<br>Variable: “PALA” and ”EA”	All visits with EA = 98 were omitted (EA= special branches of medicine, 98=general practice)<br>- type_contact = 1, if PALA = 1 or PALA = 6 (inpatient)<br>- type_contact = 2, if PALA = 91 (emergency)<br>- type_contact = 3, if PALA is not 1, 6 or 91 (outpatient, not emergency)

Link to Contents

For peer review only - http://bmjopen.bmj.com/site/about/guidelines.xhtml

Link to Contents

		PALA: 1 = inpatient ward, 6 = rehabilitation ward 91 = emergency room visit

Hospital contacts Norway

Variable	Source and description	Important notes and data preparation
id	Obtained from the Norwegian National Patient Register	Renamed from " pasientlopenr_pdb2471"
b_date	Obtained from The National Population Register	We have received information on month and year of birth, but not day. For each individual, we have therefore generated a random integer between 1 and length of their month of birth. Using this random integer as day of birth, everyone is assigned an exact birth date.
sex	Obtained from The National Population Register<br>Variable: "kjonn"	Renamed from "kjonn"
adm_date	Obtained from<br>Register: Norwegian National Patient Register<br>Variable: "innDato"	Renamed from "innDato"
discharge_date	Obtained from<br>Register: "Norwegian National Patient Register"<br>Variable: "utDato"	Renamed from "utDato". The data set only includes admissions that have ended, i.e. utDato before Dec 31, 2018.<br><br>75 contacts had missing utData. These were either outpatient contacts (n=69) or daycare procedures (n=6). utDato was defined innDato in these cases
diag	Obtained from<br>Register: "Norwegian National Patient Register"	Original dataset has one record for each hospital contact with variables hovedtilstand_1, hovedtilstand_2, bitilstand_1, …, bitilstand_19 that contain ICD 10 diagnosis codes.<br>The variables were renamed diag1, diag2, diag3, … where diag1 and diag2 correspond to the 2 primary diagnoses. The dataset was reshaped to long format containing one observation per diagnosis with variables diag, containing the ICD-10 codes and diag_ind = 1, 2, 3, …
diagtype		diagtype = 1 if diag_ind = 1 or diag_ind = 2<br>diagtype = 2 if diag_ind > 2
type_contact	Obtained from<br>Register: "Norwegian National Patient Register"<br>Variable: ""	Based on the variables Behandlingsniva3 and Aktivitetskategori3:<br>For contacts with utDato in 2008-2014:<br>• IF Behanglingsniva3 = 1 OR Behandlingsniva3 = 2 THEN type_contact = 1<br>• ELSE IF Behandlingsniva3 = 3 THEN type_contact = 4

Link to Contents

For peer review only - http://bmjopen.bmj.com/site/about/guidelines.xhtml

Link to Contents

		For contacts with utDato in 2015-2018:<br>• IF Aktivitetsjkategori3 = 1 OR Aktivitetskategori3 = 2 THEN type_contact = 1<br>• ELSE IF Aktivitetskategori3 = 3 THEN type_contact = 4

Hospital contacts Sweden

In the original Swedish register data, each admission date is a separate line with all diagnoses and other information included in one line. The dataset has been reshaped to long format with one line for each diagnosis		
**Variable**	**Source and Description**	**Restrictions**<br>**Modifications**
id	Created by Statistics Sweden<br>Pseudonomised unique personal identification number for linkage between registers	Renamed from "lopnr"
b_date	Obtained from Statistics Sweden<br>Register: "RTB"<br>Variable: "fodddatum"	Renamed from "fodddatum"
sex	Obtained from Statistics Sweden<br>Register: "RTB"<br>Variable: "kon"	Renamed from "kon"<br><br>Note: There were some discrepancies regarding sex in the two registries (RTB and Patientregistret), circa 1100 cases regarding inpatients and circa 2900 regarding outpatients. We used the information from Statistics Sweden.
adm_date	Obtained from Socialstyrelsen<br>Register: "Patientregisteret"<br>Variable: "INDATUM"	Renamed from "INDATUM".<br><br>Inpatient visits:<br>- Date missing (n=6); left unchanged.<br><br>- Date registered as earlier than birth (n=103);<br>-- dropped observations if both date of admission and discharge came before birth (n=8),<br>-- replaced date of admission with date of birth if less than 15 days apart (n=68),<br>-- replaced month or year, to align with date of discharge (n=29).<br><br>- Date registered as later than discharge but not missing (n=11); adm_date and discharge_date were shifted.<br><br>Outpatient visits:<br>- Date missing (n=1,253); left unchanged

Link to Contents

For peer review only - http://bmjopen.bmj.com/site/about/guidelines.xhtml

Link to Contents

discharge_date	Obtained from Socialstyrelsen Register: "Patientregisteret" Variable: ""UTDATUM"	For inpatient visits, the variable was renamed from "UTDATUM".<br><br>For outpatient visits, there was no corresponding variable, and the discharge date was therefore created to be equal to the admission date.
diag	Obtained from Socialstyrelsen Register: "Patientregisteret" Variables: "HDIA" and "DIAGNOS1_30"	The variable "DIAGNOS1_30" can contain up to 30 different diagnoses. It was therefore split to create separate variables for each sequential diagnosis. Duplicate codes within each observation and the code _atc were removed.
diagtype	Obtained from socialstyrelsen Register: "Patientregisteret" Variables: "HDIA" and "DIAGNOS1_30"	Diagtype was coded as 1="Main diagnosis" if indicated in variable "HDIA". If no main diagnosis was listed in variable HDIA, the first diagnosis within variable "DIAGNOS1_30" was chosen as the main diagnosis. Other diagnoses listed within DIAGNOS1_30 were coded as 2="Other diagnosis".
type_contact	Obtained from Socialstyrelsen Register: "Patientregisteret"	Variable coded based on which source file the data came from: in- or outpatient data. All data in the outpatient-file was coded = 4, as emergency room visits could not be distinguished.<br>(A variable for emergency room visits [VERKS_AKUT] was only included in the patient registry in 2016 and therefore not part of our data request.).

Link to Contents

For peer review only - http://bmjopen.bmj.com/site/about/guidelines.xhtml

Link to Contents

Table: population1

Population 1 Denmark

Variable	Source and Description	Important notes and data preparation
id	Original name in the Danish data: “pnr”. Pseudonomised unique personal identification number for linkage between registers, created by Statistics Denmark. It is linkable (by Statistics Denmark) to the original personal identification number (CPR number) assigned to all Danish residents and used when reporting to all national registers.	Renamed from “pnr”
b_date	Obtained from the Danish National Health Data Agency.<br>Register: “CPR-Registret”<br>Table: “t_person”<br>Variable: “d_foddato”	Renamed from “d_foddato”
sex	Obtained from the Danish National Health Data Agency.<br>Register: “CPR-Registret”<br>Table: “t_person”<br>Variable: “C_KON”<br>Sex as recorded by personal identification number.	sex=1 “male” if C_KON is “M”<br>sex=2 “female” if C_KON is “K”
origin	Obtained from the Danish National Health Data Agency.<br>Register: “CPR-Registeret”<br>Table: “T_FODESTED”<br>Variables: ”fodested_kode”, ”fodested_tekst”<br><br>Variables from table: population1; in_date, cens_date are used to define if there is uncertain origin	Children are categorised as:<br>1=”born in-country” if fodested_kode=000 or 208 (Denmark),<br>2= “foreign born” if fodested_kode is not 000 or 208<br>9=”unknown” if fodested_kode=000 or 208 (Denmark) and if there is date of inmigration not preceded by an outmigration (In this case we cannot be certain that the child is born in Denmark as it appears to have migrated to Denmark after the date of birth)
in_date	Obtained from the Danish National Health Data Agency.<br>Register: “CPR-Registeret”<br>Table: “T_FODESTED”<br>Variables: ”D_FODDATO”<br><br>Table: “ T_ADRESSE_UDLAND_HIST ”<br>Variables: ”C_ANNKOR”, ”D_INDREJSE_DATO”	in_date is defined as date of birth ”D_FODDATO” if ”origin” is 1=”born in-country” . in_date is defined as the first date of in-migration ”D_INDREJSE_DATO” if origin is not 1=”born in-country”.

Link to Contents

For peer review only - http://bmjopen.bmj.com/site/about/guidelines.xhtml

Link to Contents

	Table: " T_ARKIV_ADRESSE_UDLAND_HIST"<br>Variables: "C_ANNKOR", "D_INDREJSE_DATO"<br><br>Obtained from NONSense CDM<br>Table: Population1<br>Variable: "origin", "cens_date"	
in_reason	Obtained from the Danish National Health Data Agency.<br>Register: "CPR-Registeret"<br><br>Table: "T_FODESTED"<br>Variables: "D_FODDATO"<br><br>Table: " T_ADRESSE_UDLAND_HIST "<br>Variables: "C_ANNKOR", "D_INDREJSE_DATO"<br><br>Table: " T_ARKIV_ADRESSE_UDLAND_HIST"<br>Variables: "C_ANNKOR", "D_INDREJSE_DATO"<br><br>Obtained from NONSense CDM<br>Table: Population1<br>Variable:"in_date"	in_reason is categorised as:<br>1="birth" if in_date is obtained from "D_FODDATO"<br>2="immigration" if in_date is obtained from D_INDREJSE_DATO
cens_date	Obtained from the Danish National Health Data Agency.<br>Register: "CPR-Registret"<br>Table: "t_person"<br>Variables: "D_STATUS_HEN_START", "C_STATUS"<br><br>Table: " T_ADRESSE_UDLAND_HIST "<br>Variables: "C_ANNKOR", "D_UDREJSE_DATO"<br><br>Table: " T_FORSVIND_HIST"<br>Variables: "C_ANNKOR", "D_FORSVIND_DATO"	Cens_date is defined as the first date of either 1)"D_STATUS_HEN_START" if "C_STATUS" is "90"=death, "20"=CPR number for tax purposes, "70"=disappearing, "80"=out-migration or 2) D_UDREJSE_DATO or 3) D_FORSVIND_DATO.
cens_reason	Obtained from the Danish National Health Data Agency.<br>Register: "CPR-Registret"	Cens_reason is categorized as:<br>1= "death" if cens_date is obtained from C_STATUS="90" (death)

Link to Contents

For peer review only - http://bmjopen.bmj.com/site/about/guidelines.xhtml

Link to Contents

	Table: “t_person”<br>Variables:<br>“D_STATUS_HEN_START”,<br>“C_STATUS”<br><br>Table: “<br>T_ADRESSE_UDLAND_HIST ”<br>Variables: ”C_ANNKOR”,<br>”D_UDREJSE_DATO”<br><br>Table: ” T_FORSVIND_HIST”<br>Variables: ”C_ANNKOR”,<br>”D_FORSVIND_DATO”	2=”out-migration” if cens_date is obtained from C_STATUS=”80” (outmigration) or from D_UDREJSE_DATO<br>3=”other” if cens_date is obtained from C_STATUS=”20” (CPR for tax purposes” | “70” (disappearing) or from D_FORSVIND_DATO.<br><br>If more than one cens_reason is registered for the first cens_date preference is given to 1=”death”
m_id	Obtained from the Danish National Health Data Agency.<br>Register: “CPR-Registret”<br>Table: “t_person”<br>Variable:<br>“V_MOR_PNR_ENCRYPTED”	Renamed from “V_MOR_PNR_ENCRYPTED”
f_id	Obtained from the Danish National Health Data Agency.<br>Register: “CPR-Registret”<br>Table: “t_person”<br>Variable:<br>“V_FAR_PNR_ENCRYPTED”	Renamed from “V_FAR_PNR_ENCRYPTED”
m_age	Obtained from the Danish National Health Data Agency.<br>Register: “MFR” linked with-<br>Register: “CPR-Registeret”<br>Table: “t_person”<br>Variable: “d_foddato”	Id of the mother is obtained from the dataset “population1” (originally obtained from the CPR register). Using information on maternal birthday (d_foddato) and birthday of the child, Maternal age in years is calculated as age in whole years at time of delivery of the child.
m_origin	Obtained from the Danish National Health Data Agency.<br>Register MFR<br>Register: “CPR-Registeret”<br>Table: “T_FODESTED”<br>Variables: ”fodested_kode”,<br>”fodested_tekst”<br><br>Variables from table: population1; in_date, cens_date are used to define if there is uncertain origin	Id of the mother is obtained from the dataset “population1” and linked with information from the CPR register<br>1=”born in-country” if fodested_kode=000 or 208 (Denmark),<br>2= “born abroad” if fodested_kode is not 000 or 208<br>9=”unknown” if information is missing
p_origin	Obtained from the Danish National Health Data Agency.<br>Register MFR<br>Register: “CPR-Registeret”<br>Table: “T_FODESTED”<br>Variables: ”fodested_kode”,<br>”fodested_tekst”	Id of the father is obtained from the dataset “population1” and linked with information from the CPR register<br>1=”born in-country” if fodested_kode=000 or 208 (Denmark),<br>2= “born abroad” if fodested_kode is not 000 or 208

Link to Contents

For peer review only - http://bmjopen.bmj.com/site/about/guidelines.xhtml

Link to Contents

	Variables from table: population1; in_date, cens_date are used to define if there is uncertain origin	9="unknown" if the information is missing

Population 1 Finland

Variable	Source and Description	Important notes and data preparation
id	Obtained from:<br>Register: Population register<br>Table: VTJ.HENKILO<br>Variable: hetu<br><br>Table: VTJ.HENKILO_HETU<br>Variable: hetu_voimassa<br><br>THL pseudonymised the original personal identification code (in this register "hetu") to unique personal identification number for linkage between registers. THL data management can link the id back to original personal identification code.	Person included only if hetu_voimassa (=id is valid) is checked.<br><br>Statistics Finland pseudonymised "hetu" with their own id for the remote user system.
b_date	Obtained from:<br>Register: Population register<br>Table:VTJ.HENKILO<br>Variable: syntymapaiva	Renamed from syntymapaiva
sex	Obtained from:<br>Register: Population register<br>Table: VTJ.HENKILO<br>Variable: sukupuoli	sex=1 "male" if lapsen sukupuoli is "mies"<br>sex=2 "female" if lapsen sukupuoli is "nainen"
origin	Obtained from:<br>Register: Population register<br>Table:VTJ.HENKILO<br>Variable: "syntymakunta"	1 = born in country,<br>if the code of syntymakunta (birth municipality is not 200 or NA (not available)<br>2 = born abroad,<br>if syntymakunta is 200<br>3 = uncertain foreign or in-country, if syntymakunta is NA, 198,199 or 000 (children born abroad were excluded as only minority of them had immigration dates available in the THL's population register copy, in which the follow-up begin in 2014, also children with uncertain origin were excluded)

Link to Contents

For peer review only - http://bmjopen.bmj.com/site/about/guidelines.xhtml

Link to Contents

in_date	Obtained from:<br>Register: Population register<br>Table:VTJ.HENKILO<br>Variable: ”syntymapaiva” and	If born in country (origin=1), equal to the date of birth = syntymapaiva
in_reason	Obtained from:<br>Register: Population register<br>Table:VTJ.HENKILO<br>Variable: ”syntymapaiva”	1 = Birth,<br>if born in the country (origin=1)
cens_date	Obtained from:<br>Register: Population register<br>Table: VTJ.HENKILO<br>Variable: “KUOLINPVM”<br>and<br>Register: Statistic Finland<br>Table:<br>Variable: “kuolinpäivä”<br>Variable: ensimmäinen maastamuuttopäivä<br><br>Table: VTJ.HENKILO<br>Table: KOTIKUNTAHISTORIA:<br>Variable: ”kotikunta” and ”kunta muuttopaiva”	Equal to date of emigration, if such has occurred, otherwise equal to date of death.<br><br>Emigration from Population register (select min (kunta_muuttopvm) from vtj.henkilo_kotikuntahistoria and kunta='200')<br><br>Ensimmäinen maastamuuttopäivä=first emigration date available only in remote user system Fiona
cens_reason	Obtained from:<br>Register: Population register<br>Table: VTJ.HENKILO<br>Variable:kuolinpvm, muuttopvm	1 = Death<br>If subject died<br>2 = Emigration<br>If subject emigrated
m_id	Obtained from:<br>Birth register<br>Table: Äidin henkilötiedot<br>Variable: aiti_hetunnus<br><br>THL pseudonymised the original personal identification code (in this register “aiti_hetunnus”) to unique personal identification number for linkage between registers. THL data management can link the id back to original personal identification code.	Renamed from “aiti_hetunnus” and pseudonymised by Statistics Finland for data lingage.
f_id	Obtained from: Statistics Finland	Not available for THL. Pseudonymised id for data linkage in Statistics Finland
m_age	Obtained from<br>Register: Birth register<br>Table: Äidin henkilötiedot<br>Variable: aiti_ika	Renamed from aiti_ika
m_origin	Obtained from<br>Register: Statistics Finland<br>Table:<br>Variable: svaltio_aiti	Available only in the Fiona remote user system.<br>svaltio_aiti = 246 -> 1 = "born in-country"<br>svaltio_aiti != 246 (ts joku muu kuin Suomi) -> 2 = "born abroad"

Link to Contents

For peer review only - http://bmjopen.bmj.com/site/about/guidelines.xhtml

Link to Contents

		svaltio_aiti = NA (ts puuttuu) -> 9 = "Unknown"
p_origin	Obtained from<br>Register: Statistics Finland<br>Table:<br>Variable: svaltio_isa	Available only in the Fiona remote user system.<br>svaltio_isa = 246 -> 1 = "born in-country"<br>svaltio_isa != 246 (ts joku muu kuin Suomi) -> 2 = "born abroad"<br>svaltio_isa = NA (ts puuttuu) -> 9 = "Unknown"

Population 1 Norway

Variable	Source and Description	Important notes and data preparation
Variable	Source and Description	Important notes and data preparation
id	Obtained from: SSB (Statistics Norway)<br>Register: “National Population Register”	Renamed from pasientlopenr_pdb2471
b_date	Obtained from: SSB (Statistics Norway)<br>Register: “National Population Register”	For all individuals in population1 as well as their parents, we have received information on month and year of birth, but not day. For each individual, we have therefore generated a random integer between 1 and length of their month of birth. Using this random integer as day of birth, everyone is assigned an exact birth date.
sex	Obtained from: SSB (Statistics Norway)<br>Register: “National Population Register”	Renamed from kjonn
origin	Obtained from: SSB (Statistics Norway)<br>Register: “National Population Register”	Based on the variables in_date (see below) and ”fodeland”. Origin is coded as 1 if country of birth is Norway (fodeland = 0) and in_date is equal to date of birth. Origin is coded as 2 if country of birth is any other country. Origin is coded as 9 if country of birth is Norway and in_date is later than date of birth.
in_date	Obtained from: SSB (Statistics Norway)<br>Register: “National Population Register”	Based on the variables “regstatus”, “regstatusdato”, “forstdato” and “fodeland”. Indate is defined as forstdato if invkat = B (immigrants). “forstdato” is the date of first registration in the Population Registry. The variable is only defined for persons with invkat =B (immigrants).<br>Otherwise (invkat = A, C, E, F or G), indate is defined as a person’s earliest regstatusdato with regstatus = 1 (Bosatt). In general, individuals who have been residents in Norway since birth, will be registered with

Link to Contents

For peer review only - http://bmjopen.bmj.com/site/about/guidelines.xhtml

Link to Contents

		regstatus = 1 and corresponding regstatusdato = date of birth. However, regstatus is only available as of January 1 each year. If a person's regstatus has changed more than once during a calendar year, we only have information about the most recent change. Therefore, in_date was set to date of birth for individuals with country of birth Norway who died or emigrated in their year of birth even if they do not have a record with regstatus = 1 and regstatusdato = date of birth.<br>Note: cross-checked with the Birth Registry, and > 98% of children with country of birth Norway who died or emigrated in their year of birth have a record in the Birth Registry. Thus, it is a reasonable assumption that these children have been residents of Norway since birth.
in_reason	Obtained from: SSB (Statistics Norway)<br>Register: "National Population RegisterBefolkning	in_reason is coded as 1 if origin = 1.<br>in_reason is coded as 2 if origin = 2 or origin = 9.
cens_date	Obtained from: SSB (Statistics Norway)<br>Register: "National Population RegisterBefolkning	Based on the variables "regstatus", "regstatusdato", and "dodsdato". Date of emigration was defined as a person's earliest regstatusdato with regstatus = 3 (emigration). Date of death was defined as dodsdato. We only have information on month and year of death. Exact date of date was assigned as a random integrer within the month of death. cens_date was set to date of emigration if emigration occurred before date of 18th birthday or January 1, 2019. cens_date was set to date of death if death occurred before date of 18th birthday or January 1, 2019, unless date of death was preceded by date of emigration (N = 40).
cens_reason	Obtained from: SSB (Statistics Norway)<br>Register: "National Population RegisterBefolkning	cens_reason was coded as 1 if cens_date = date of death. cens_reason is coded as 2 if cens_date = date of emigration.
m_id	Obtained from: SSB (Statistics Norway)<br>Register: "National Population Register"<br>Variable: Løpenummer mor	Renamed from lopenr_mor_pdb2471
f_id	Obtained from: SSB (Statistics Norway)<br>Register: "National Population Register"	Renamed from lopenr_far_pdb2471

Link to Contents

For peer review only - http://bmjopen.bmj.com/site/about/guidelines.xhtml

Link to Contents

	Variable: Løpenummer far	
m_age	Obtained from: SSB (Statistics Norway)<br>Register: "National Population RegisterBefolkning	Mother's age in whole years at time of birth of child. Based on the mother's assigned exact date of birth (b_date).
m_origin	Obtained from: SSB (Statistics Norway)<br>Register: "National Population RegisterBefolkning	Based on the variable "fodeland". m origin = 1 if mother's country of birth is Norway (fodeland = 0), m_origin = 2 if mother's country of birth is any other country, and m_origin = 9 if mother's country of birth is missing (n = 20,559).

For peer review only

Link to Contents

For peer review only - http://bmjopen.bmj.com/site/about/guidelines.xhtml

Link to Contents

Population 1 Sweden

Variable	Source and Description	Important notes and data preparation
id	Created by Statistics Sweden<br>Register: Registret över totalbefolkningen (RTB)<br>Variable: lopnr	Pseudonomised unique personal identification number for linkage between registers<br><br>Renamed from "lopnr".
b_date	Obtained from Statistics Sweden<br>Register: Registret över totalbefolkningen (RTB)<br>Variable: fodddatum	Renamed from "fodddatum"
sex	Obtained from Statistics Sweden<br>Register: Registret över totalbefolkningen (RTB)<br>Variable: kon	Renamed from "kon"
origin	Obtained from Statistics Sweden<br>Register: Registret över totalbefolkningen (RTB)<br>Variable: UtlSvBakg<br><br>Combined with data from the National Board of Health and Welfare<br>Register: Medical Birth Registry	Recoded from: "UtlSvBakg" where<br>11 = Born abroad<br>12 = Born in the country with two foreign-born parents<br>21 = Born in the country with one native and one foreign born parent<br>22 = Born in the country with two native born parents.<br><br>Individuals were coded<br>1 = "born in-country", if UtlSvBakg = 12, 21 or 22,<br>and 2 = "born abroad", if UtlSvBakg = 11.<br><br>Individuals were coded 9 = "Unknown" if registered as born in country (UtlSvBakg = 12, 21 or 22) but also had a registered immigration date not preceded by an emigration date. (In this case we cannot be certain that the child was born in the country as it appeared that they have immigrated after the date of birth.)<br><br>If the individual was initially coded as 9 "Unknown", but was registered in the medical birth registry, they were recoded as 1 = "born in-country".
in_date	Variable created based on information from Statistics Sweden<br>Register: Registret över totalbefolkningen (RTB)<br>Variables: fodddatum and datum [migration], posttyp [migration]	If born in country (origin=1), equal to the date of birth = fodddatum<br>If born outside the country (origin=2), equal to first date of immigration<br>If unknown origin (=9), equal to first date of immigration
in_reason	Variable created based on information from Statistics Sweden	1 = Birth,<br>if born in the country (origin=1)<br>2 = Immigration,

Link to Contents

For peer review only - http://bmjopen.bmj.com/site/about/guidelines.xhtml

Link to Contents

	Register: Registret över totalbefolkningen (RTB)<br>Variables: foddatum, datum, posttyp	if born abroad (origin=2) or unknown origin (origin=9)
cens_date	Variable created based on information from Statistics Sweden<br>Register: Registret över totalbefolkningen (RTB)<br>Variables: Doddatum, datum [migration], posttyp [migration]	Equal to date of emigration, if such an event had been registered, otherwise equal to date of death.
cens_reason	Variable created based on information from Statistics Sweden<br>Register: Registret över totalbefolkningen (RTB)<br>Variables: Doddatum, datum [migration], posttyp [migration]	1 = Death, if there was a date of death registered in variable Doddatum.<br>2 = Out migration, if there was a registered migration out of the country
m_id	Obtained from Statistics Sweden<br>Register: Flergenerationsregistret	Renamed from “LopNrMor”
f_id	Obtained from Statistics Sweden<br>Register: Flergenerationsregistret	Renamed from “LopNrFar”
m_age	Obtained from Statistics Sweden<br>Register: RTB<br>Variable: datum_fodd	Calculated as mother’s date of birth minus the child’s date of birth, divided by 365, and rounded down to yield age in years.
m_origin	Obtained from Statistics Sweden<br>Register: RTB<br>Variable: UtlSvBakg	Recoded from variable “UtlSvBakg” as described above for variable Origin in table Population 1.
p_origin	Obtained from Statistics Sweden<br>Register: RTB<br>Variable: UtlSvBakg	Recoded from variable “UtlSvBakg” as described above for variable Origin in table Population 1.

Link to Contents

For peer review only - http://bmjopen.bmj.com/site/about/guidelines.xhtml

Link to Contents

Table: birth_charcteristics

birth_characteristics Denmark

Variable	Source and Description	Important notes and data preparation
id	Original name in the Danish data: “pnr”. Pseudonomised unique personal identification number for linkage between registers, created by Statistics Denmark. It is linkable (by Statistics Denmark) to the original personal identification number (CPR number) assigned to all Danish residents and used when reporting to all national registers.	Renamed from “pnr”
b_weight	Obtained from the Danish National Health Data Agency.<br>Register: “MFR” from 1997 and onwards, “Fødselsregisteret” before 1997<br>Table: “MFR”(from MFR), ”levendefødt” (From fødselsregisteret)<br>Variable: “vaegt_barn” (MFR), V_VAGT (fødselsregisteret)	Renamed from “vaegt_barn” and “ V_VAGT”<br>Registrations of birthweight less than 100g or higher than 9990g are categorized as missing
ga	Obtained from the Danish National Health Data Agency.<br>Register: “MFR” from 1997 and onwards, “Fødselsregisteret” before 1997<br>Table: “MFR”(from MFR), ”levendefødt” (From fødselsregisteret)<br>Variable: “Gestationsalder_dage” (MFR), ”V_SVLANGDE” (fødselsregisteret)	Derived from “Gestationsalder_dage” (ga in days) rounded down to whole weeks of gestation: ga=floor(gestationsalder_dage/7)<br><br>Renamed from V_SVLANGDE
sectio	Obtained from the Danish National Health Data Agency.<br>Register: “MFR” from 1997 and onwards, “Fødselsregisteret” before 1997<br>Table: “MFR”(from MFR), ”levendefødt” (From fødselsregisteret)<br><br>Variables: ” Markoer_kejsersnit” (MFR), B_I11, B_SECTIOF, B_SECTIOU (fødselsregisteret)	From MFR:<br>0=”not delivered by caesarean section” if they do not have any diagnosis code indicating caesarean section (”Markoer_kejsersnit”=missing)<br><br>1=”delivered by caesarean section” if they have a diagnosis code indicating caesarean section in the variable ”Markoer_kejsersnit”<br><br>Fødselsregisteret<br>sectio=1 if B_I11=1 | B_SECTIOU=1 | B_SECTIOF=1<br><br>Otherwise sectio=0

Link to Contents

For peer review only - http://bmjopen.bmj.com/site/about/guidelines.xhtml

Link to Contents

smoke	Obtained from the Danish National Health Data Agency.<br>Register: “CPR-Registeret”<br>Register: “MFR” from 1997 and onwards, “Fødselsregisteret” before 1997<br>Table: “MFR”(from MFR), ”levendefødt” (From fødselsregisteret)<br>Variable: ”rygerstatus_moder” (MFR), B_RYGER (fødselsregisteret)<br><br>Obtained from the Danish National Health Data Agency.<br>Register: Danish national patient registry<br>Table: “T_ADM”, “T_DIAG”<br>Variables: pnr, recnum, D_INDDTO, D_UDDTO, C_ADIAG, C_TILDIAG,	Information from MFR, variable “RYGERSTATUS_MODER”<br>Smoke=0 if rygerstatus_moder=0<br>Smoke=1 if rygerstaus moder >0 and <99 (indicating any smoking during pregnancy regardless of magnitude)<br>Smoke=9 if rygerstatus_moder=99(unknown) or missing.<br><br>From fødselsregisteret:<br>smoke=0 if B_RYGER=0<br>smoke=1 if B_RYGER=1<br>smoke=9 if B_RYGER=.<br><br>For some pregnancies especially in 1997 and partially in 1998, smoke information is not available in MFR, but we are able to subtract the information from the patient registry using the additional diagnosis “DUT00-DUT99”.<br><br>Information about smoke is inserted from the patient registry if: a) the information is not present in MFR/fødselsregisteret; b) if the patient registry indicates smoking while MFR/fødselsregisteret indicates no smoking or unknown.
singleton	Obtained from the Danish National Health Data Agency.<br>Register: “CPR-Registeret”<br>Register: “MFR” from 1997 and onwards, “Fødselsregisteret” before 1997<br>Table: “MFR”(from MFR), ”levendefødt” (From fødselsregisteret)<br>Variable: ”Flerfoldsgraviditet” (MFR), C_PLAC (fødselsregisteret)	MFR<br>Children are categorized as:<br>0=”no” if there is an indication of multiple child delivery (diagnosis code) or there is registered another child born by the same mother within 1 day from the child’s birthday<br><br>1=”yes” if there is no indication of multiple child delivery (”Flerfoldsgraviditet”=missing)<br><br>Fødselsregisteret<br>Children are categorized as:<br>0=”no” if C_PLAC>0 or there is registered another child born by the same mother within 1 day from the child’s birthday<br><br>1=”yes” if C_PLAC=0 and no child born by the same mother within 1 day from the child’s birthday
child_order	Obtained from the Danish National Health Data Agency.<br>Register: “CPR-Registeret”	The variable from MFR contains information on number of fulfilled pragnancies including stillbirths. Before 1997 the variables

Link to Contents

For peer review only - http://bmjopen.bmj.com/site/about/guidelines.xhtml

Link to Contents

	Register: “MFR” from 1997 and onwards, “Fødselsregisteret” before 1997<br>Table: “MFR”(from MFR), ”levendefødt” (From fødselsregisteret)<br>Variable: ”paritet” (MFR), V_TIDLLEV, V_TIDLDOD (fødselsregisteret)	V_TIDLLEV(previous live births)+V_TIDLDOD (previous still births) has been added plus 1(current delivery), to simulate the information from MFR.<br><br>Second, a counting method is applied using the registered parity indication for the first registered child and counting onwards for following liveborn children.<br>Preparation is done in 3 steps:<br>1) parity of the first registered child is determined:<br>a) missing information on the first registered child by a mother but with information on the second registered child are recoded with parity of the second child minus 1.<br>b) children with missing information on the first registered child are recoded with parity=1 if the second child is registered as parity=1.<br>2) child order of following children is determined using a counting method from the parity of the first registered child plus 1 for each following child<br>3) multiple delivered children are identified, and child order is recoded to the lowest value i.e., twins with 1 older sibling will both be coded with parity=2

birth_characteristics Finland

Variable	Source and Description	Important notes and data preparation
id	Obtained from<br>Register: Birth Register<br>Table:<br>Variable: lapsi_hetunnus<br>THL pseudonymised the original personal identification code (lapsi_hetunnus) to unique personal identification number for linkage between registers. THL data management can link the id back to original personal identification code.	Statistics Finland pseudonymised lapsi_hetunnus with their own id for the remote user system.
b_weight	Obtained from<br>Register: Birth Register<br>Table:<br>Variable: syntymapaino	Registrations of birthweight less than 100g or higher than 9990g are categorized as missing
ga	Obtained from<br>Register: Birth Register<br>Table:<br>Variable: kestovkpv	kestovkpv, ga will be notified as weeks, the days are not noted.<br><br>Ga <20 or >45 are coded as missing

Link to Contents

For peer review only - http://bmjopen.bmj.com/site/about/guidelines.xhtml

Link to Contents

sectio	Obtained from<br>Register: Birth Register<br>Table:<br>Variable: synnytystapatunnus	Children are categorised as:<br>0=”not delivered by caesarean section” if synnytystapatunnus is 1-4<br><br>1=”delivered by caesarean section” if synnytystapatunnus is 5-8<br><br>9="unknown" if synnytystapatunnus=9 or missing
smoke	Obtained from<br>Register: Birth Register<br>Table:<br>Variable: tupakointitunnus	Smoke=0 if tupakointitunnus=1<br>Smoke=1 if tupakointitunnus =2-4<br>Smoke=9 if tupakointitunnus=9 (unknown) or missing.
singleton	Obtained from<br>Register: Birth Register<br>Table:<br>Variable: sikioita	Children are categorized as:<br>0=”no” if sikioita=2 or more<br><br>1=”yes” if sikioita=1
Child_order	Obtained from<br>Register: Birth Register<br>Table:<br>Variable: aiemmatsynnytykset<br>Variable: kuolleenasynt	Number of the child =“Aiemmatsynnytykset” (previous births) minus “kuolleenasynt” (=stillbirths) plus 1<br>multiple delivered children are identified, and parity is recoded to the lowest value i.e., twins with 1 older sibling will both be coded with child order=2

birth_characteristics Norway

Variable	Source and Description	Important notes and data preparation
id		Renamed from “pasientlopenr_pdb2471”
b_weight	Obtained from<br>Register: Medical Birth Registry of Norway<br>Variable: vekt	Registrations of birthweight less than 100g or higher than 9990g are defined as missing
ga	Obtained from<br>Register: Medical Birth Registry of Norway<br>Variable: svlen	ga is calculated as floor(svlen/7), where svlen is the length of gestation in days based on ultrasound estimation. If ultrasound is not available, the gestational length is calculated from the last menstrual period.
sectio	Obtained from<br>Register: Medical Birth Registry of Norway<br><br>Variable: ksnitt	Information on delivery with c-section is obtained from the variable ksnitt. Possible values of ksnitt are<br>1 = Planned C-section<br>2 = Emergency C-section<br>9 = Unspecified C-section<br>If ksnitt is missing, sectio is coded as 0. Otherwise, sectio is coded as 1.
smoke	Obtained from<br>Register: Medical Birth Registry of Norway<br>Variable: royk_beg and royk_avsl	Information on smoking at start and end of pregnancy is obtained from royk_beg and royk_avsl, respectively. Both variables are coded as

Link to Contents

For peer review only - http://bmjopen.bmj.com/site/about/guidelines.xhtml

Link to Contents

		1 = No<br>2 = Sometimes<br>3 = Daily<br>If royk_beg = 1 AND royk_avsl = 1, smoke is coded as 0<br>If royk_beg = 2 OR royk_beg = 3 OR royk_avsl = 2 OR royk_avsl = 3, smoke is coded as 1<br>Otherwise smoke = 9.<br>Mothers can opt out of having information on smoking recorded. Thus, royk_beg and royk_avsl is missing for a high proportion of births. The proportion with smoke = 9 is 43%.
singleton	Obtained from<br>Register: Medical Birth Registry of Norway<br>Variable: flerfodsel	singleton is coded as 0 if flerfodsel = 1 or if another child is born to the same mother in the same month (N = 13). Otherwise, singleton is coded as 1.
child_order	Obtained from<br>Register: Medical Birth Registry of Norway<br>Variable: paritet	parity is defined as paritet + 1.<br>The variable paritet is defined by MBRN as the highest value of the variables paritet_mor and paritet_mfr, where paritet_mor is number of previous deliveries as stated by mother and paritet_mor is number of previous deliveries registered by MBRN. Stillbirths are included in paritet.<br><br>Pairs of twins should have the same value of parity and will therefore be assigned the same value of parity (lowest within the set).

Birth characteristics Sweden

Variable	Source and Description	Important notes and data preparation
id	Created by Statistics Sweden<br>Pseudonomised unique personal identification number for linkage between registers	Renamed from lopnr
b_weight	Obtained from Socialstyrelsen<br>Register: Medicinska födelseregistret<br>Variable: bvikt	Registrations of birthweight less than 100g or higher than 9990g were categorized as missing.
ga	Obtained from Socialstyrelsen<br>Register: Medicinska födelseregistret<br>Variable: grvbs	Socialstyrelsen recommends using this variable (for the best estimated gestational age), over the variable grvfv (which is based on medical records).
sectio	Obtained from Socialstyrelsen<br>Register: Medicinska födelseregistret<br>Variable: secmark	Variable renamed from secmark; coding unaltered: 0 = no, 1 =yes.

Link to Contents

For peer review only - http://bmjopen.bmj.com/site/about/guidelines.xhtml

Link to Contents

smoke	Obtained from Socialstyrelsen<br>Register: Medicinska födelseregistret<br>Variable: rok1	The variable rok1 pertains to smoking habits at registration with maternal health (usually at 8-12 weeks of pregnancy).<br><br>If the woman was smoking >=1 cigarette/day at registration (rok1 coded 2 or 3), the variable smoke was coded = 1.<br>If the woman was not smoking (rok1 coded 1) the variable smoke was coded = 0.<br>If data was missing the variable smoke was coded = 9 (missing).<br><br>(There is another variable, rok2, which pertains to smoking habits at pregnancy week circa 30-32. This was not included due to very poor data quality 1990-1999, and poor completeness thereafter (Source publication: Graviditeter, förlossningar och nyfödda barn (socialstyrelsen.se) , Statistikdatabaser - Förlossningsstatistik - Val (socialstyrelsen.se))
singleton	Obtained from Socialstyrelsen<br>Register: Medicinska födelseregistret<br>Variable: bordf2	1="Enkelbörd" was left unaltered (=1 "Yes").<br>2="Flerbörd" was recoded to 0 "No".
child_order	Obtained from Socialstyrelsen<br>Register: Medicinska födelseregistret<br>Variable: paritet	The child's order, based on the number of children previously born by the mother, including this birth.<br>Twins were given the same number, the lowest within the set.

For peer review only

Link to Contents

For peer review only - http://bmjopen.bmj.com/site/about/guidelines.xhtml

Link to Contents

Table: Vaccines

Vaccines Denmark

Variable	Source and Description	Important notes and data preparation
id	Original name in the Danish data: "pnr". Pseudonomised unique personal identification number for linkage between registers, created by Statistics Denmark. It is linkable (by Statistics Denmark) to the original personal identification number (CPR number) assigned to all Danish residents and used when reporting to all national registers.	string
vacdate	Obtained from the state serum institue.<br>Register: "vaccinationsregisteret"<br>Variable: "EffectuationDate"	Date Format (%dD_m_Y)
vaccine	Obtained from the state serum institue.<br>Register: "vaccinationsregisteret"<br>Variable: "ATCCode"	Categorical (see coding in appendix "vaccine categorization")<br>Duplicates were handled as follows, so that only one entry was kept:<br>- same ATCCode: duplicate removed.<br>- same group of vaccines (see appendix vaccine categorization) within 14 days: the entry most likely to have been administered according to the national vaccination schedule at the time was kept.<br>-Hib-vaccine given within 14 days of a multivalent Hib-containing vaccine: was removed<br>- IPV given within 14 days of an multivalent IPV-containing vaccines: was removed
credibility	Variable generated based on data preparation	1=no duplicate<br>2= duplicate same vaccine removed<br>3=duplicate related vaccine removed (keep vaccine that aligns with vaccination schedule)<br>4= duplicate related vaccine removed (none of the vaccines align with vaccination schedule)<br>5= duplicate related vaccine removed (vaccines given outside usual vaccination ages within the national immunisation programme)
TB_endemic		9= not relevant
HepB_endemic		9=not relevant

Link to Contents

For peer review only - http://bmjopen.bmj.com/site/about/guidelines.xhtml

Link to Contents

Vaccines Finland

Variable	Source and Description	Important notes and data preparation
id	Obtained from<br>Register: Vaccination Register<br>Table:<br>Variable: hetu<br><br>THL pseudonymised the original personal identification code (hetu) to unique personal identification number for linkage between registers. THL data management can link the id back to original personal identification code.	Statistics Finland pseudonymised “hetu” with their own id for the remote user system.
vacdate	Obtained from<br>Register: Vaccination Register<br>Table:<br>Variable: Recorddate	Date Format (%dD_m_Y)
vaccine	Obtained from<br>Register: Vaccination Register<br>Table:<br>Variable: atc_code	Categorical (see coding in appendix ”vaccine categorization”)<br>Duplicates were handled as follows, so that only one entry was kept:<br>- same ATCCode: duplicate removed.<br>- same group of vaccines (see appendix vaccine categorization) within 14 days: the entry most likely to have been administered according to the national vaccination schedule at the time was kept.<br>-Hib-vaccine given within 14 days of a multivalent Hib-containing vaccine: was removed<br>- IPV given within 14 days of an multivalent IPV-containing vaccines: was removed
credibility	Variable generated based on data preparation	1=no duplicate<br><br>2= duplicate same vaccine removed<br><br>3=duplicate related vaccine removed (keep vaccine that aligns with vaccination schedule)<br><br>4= duplicate related vaccine removed (none of the vaccines align with vaccination schedule)<br><br>5= duplicate related vaccine removed (vaccines given outside usual vaccination ages within the national immunisation programme)
TB_endemic		9= not relevant
HepB_endemic		9=not relevant

For peer review only - http://bmjopen.bmj.com/site/about/guidelines.xhtml

Link to *Contents*

Vaccines Norway

Variable	Source and Description	Important notes and data preparation
id	Obtained from Register: Norwegian Immunisation Registry (SYSVAK)	string
vacdate	Obtained from Register: Norwegian Immunisation Registry (SYSVAK) Variable: konsultasjonsdato	Renamed from konsultasjonsdato Date Format (%dD_m_Y)
vaccine	Obtained from Register: Norwegian Immunisation Registry (SYSVAK) Variable: vaksinekode	Categorical (see coding in appendix ”vaccine categorization”) Duplicates by same ATCCode are removed. Duplicates by same group of vaccines (see appendix vaccine categorization) within 14 days are cleaned based on information on which vaccine is most likely to have been administered according to the national vaccination schedule and historical changes. Hib given within 14 days of Hib containing vaccines are removed IPV given within 14 days of IPV containing vaccines are removed
credibility	Variable generated based on data preparation	1=no duplicate<br>2= duplicate same vaccine removed<br>3=duplicate related vaccine removed (keep vaccine that aligns with vaccination schedule)<br>4= duplicate related vaccine removed (none of the vaccines align with vaccination schedule)<br>5= duplicate related vaccine removed (vaccines given outside usual vaccination ages within the national immunisation programme)
TB_endemic		9=not relevant
HepB_endemic		9=not relevant

Vaccines Sweden

Variable	Source and Description	Important notes and data preparation
id	Created by Statistics Sweden Pseudonomised unique personal identification number for linkage between registers	String

Link to *Contents*

For peer review only - http://bmjopen.bmj.com/site/about/guidelines.xhtml

Link to Contents

vacdate	Obtained from The Public Health Agency of Sweden (PHAS)<br>Register: The National Vaccination Registry (NVR)<br>Variable: vaccination_date	Date Format (%dD_m_Y)
vaccine	Obtained from: PHAS<br>Register: NVR<br>Variable: atc, product_name	Categorical (see coding in appendix ”vaccine categorization”)<br>Duplicates were handled as follows, so that only one entry was kept:<br>- same ATCCode: duplicate removed.<br>- same group of vaccines (see appendix vaccine categorization) within 14 days: the entry most likely to have been administered according to the national vaccination schedule at the time was kept.<br>-Hib-vaccine given within 14 days of a multivalent Hib-containing vaccine: was removed<br>- IPV given within 14 days of an multivalent IPV-containing vaccines: was removed
credibility	Variable generated based on data preparation	1=no duplicate<br>2= duplicate same vaccine removed<br>3=duplicate related vaccine removed (keep vaccine that aligns with vaccination schedule)<br>4= duplicate related vaccine removed (none of the vaccines align with vaccination schedule)<br>5= duplicate related vaccine removed (vaccines given outside usual vaccination ages within the national immunisation programme)
TB_endemic	Obtained from Statistics Sweden<br>Register: RTB<br>Variable: fodelselandnamn	If the child, mother OR father was born in a country with high or very high incidence of tuberculosis ie. >25 cases per 100,000 inhabitants (as listed in WHO:s Global TB report 2018, link), the child was coded 1=risk group, as this corresponds to eligibility for BCG-vaccination.<br>All other children were coded = 0.
HepB_endemic	Obtained from Statistics Sweden<br>Register:<br>Table:<br>Variable: fodelselandnamn	If the child, mother OR father was born in a country with an intermediary or high prevalence of hepatitis B in the population (> 2 percent HbsAg-positive), the child was coded 1=risk group. *

Link to Contents

For peer review only - http://bmjopen.bmj.com/site/about/guidelines.xhtml

Link to Contents

		If the child and both parents came from low prevalence countries, the child was coded = 0. (This included all native-born children.)<br><br>If the child came from a country with an unknown prevalence, it was coded as missing.

* Source: Schweitzer A, Horn J, Mikolajczyk RT, Krause G, Ott JJ. Estimations of worldwide prevalence of chronic hepatitis B virus infection: a systematic review of data published between 1965 and 2013. The Lancet. 2015;386(10003):1546-55. DOI:https://doi.org/10.1016/S0140-6736(15)61412-X.

For peer review only

Link to Contents

For peer review only - http://bmjopen.bmj.com/site/about/guidelines.xhtml

Link to Contents

Table: socio_economy

Socio_economy Denmark

Variable	Source and Description	Important notes and data preparation
id	Original name in the Danish data: "pnr". Pseudonomised unique personal identification number for linkage between registers, created by Statistics Denmark. It is linkable (by Statistics Denmark) to the original personal identification number (CPR number) assigned to all Danish residents and used when reporting to all national registers.	Renamed from "pnr"
inc_quin_b	Obtained from Statistics Denmark. Table: "FAIK" (tables for each year) Variable: "FAMAEKVIVADISP_13" (Equated disposable family income)<br><br>Link between each child and family is obtained from Statistics Denmark: Table: BEF (tables for each year) Link variable: FAMILIE_ID (combined with calendar year)	Birth year 2016 and higher do not have information on family income at birth.<br>No children have information from the year they are born, because the statistics are made on the first of January each year. Include information from the year after birth. If no info from that year, the child is coded with unknown (9).<br>Note: quintiles made separately for each calendar year for the children born the year before.
inc_quin_10y	Obtained from Statistics Denmark. Table: "FAIK" (tables for each year) Variable: "FAMAEKVIVADISP_13" (Equated disposable family income)<br><br>Link between each child and family is obtained from Statistics Denmark: Table: BEF(tables for each year) Link variable: FAMILIE_ID (combined with calendar year)	Birth year 2007 and higher do not have info on family income at ten years.<br>If no info from the year the child turn 10 years the variable is coded with unknown (9).<br>Note: quintiles made separately for each calendar year for the children turning 10 years that year.
n_children_b	Obtained from Statistics Denmark. Table: "FAM" (tables for each year) Variables: Sumarized from the variables ANTB00-ANTB17 (number of children in the family age 0, 1, 2…,17)<br><br>Link between each child and family is obtained from Statistics Denmark: Table: BEF(tables for each year) Link variable: FAMILIE_ID (combined with calendar year)	Birth year 2018 do not have info on number of children at birth.<br>No children have information from the year they are born, because the statistics made on the first of January each year. Include information from the year after birth. If no info from that year the child is code with unknown (99).<br>Some children end-up with a count of 0 children, as this is not a legal value they are recoded to 99. Based on the values on family_type, it is judged that the children with a count of 0, are children who are registered as the main person in a family and therefore are not counted as a child although they are children.
n_children_10y	Obtained from Statistics Denmark. Table: "FAM" (tables for each year)	Birth year 2009 and higher do not have info on number of children at 10 years.

Link to Contents

For peer review only - http://bmjopen.bmj.com/site/about/guidelines.xhtml

Link to Contents

	Variables: Sumarized from the variables ANTB00-ANTB17 (number of children in the family age 0, 1, 2…,17 years)<br><br>Link between each child and family is obtained from Statistics Denmark: Table: BEF(tables for each year) Link variable: FAMILIE_ID (combined with calendar year)	If no info from the year the child turn 10 years the variable is coded with unknown (99).<br>Some children end-up with a count of 0 children, as this is not a legal value they are recoded to 99. Based on the values on family_type, it is judged that the children with a count of 0, are children who are registered as the main person in a family and therefore are not counted as a child although they are children.
Single_parent_b	Obtained from Statistics Denmark. Table: "FAM" (tables for each year) Variable: FAMILIE_TYPE<br><br>Link between each child and family is obtained from Statistics Denmark: Table: BEF(tables for each year) Link variable: FAMILIE_ID (combined with calendar year)	Birth year 2018 do not have info on single parenthood at birth.<br>No children have information from the year they are born, because the statistics made on the first of January each year. Include information from the year after birth. If no info from that year the child is code with unknown (9).<br>I also set children who originally were coded with 0 children on n_children_b as unknown (9) because it is judgded that these are children registered as the main person in the family (no adults in the family?).
Single_parent_10y	Obtained from Statistics Denmark. Table: "FAM" (tables for each year) Variable: FAMILIE_TYPE<br><br>Link between each child and family is obtained from Statistics Denmark: Table: BEF(tables for each year) Link variable: FAMILIE_ID (combined with calendar year)	Birth year 2018 do not have info on single parenthood at birth.<br>If no info from the year the child turn 10 years the variable is coded with unknown (9).<br>set children who originally were coded with 0 children on n_children_10y as unknown (9) because it is judged that these are children registered as the main person in the family (no adults in the family?).
m_education_b	Obtained from Statistics Denmark. Table: "UDDF" Variable: "hfaudd" "hfaudd" i linked with format from statistics Denmark grouping the Danish education classification into ISCED 2011, based on which maternal education is grouped.<br><br>Link to mother is available from the dataset "population1" (originally obtained from the CPR register).	Use the highest obtained education for the mother on the date of birth of the child. There is no information on this for children born 2017 or later. Statistics Denmark had a format available for transforming national Danish education codes into ISCED.
m_education_10y	Obtained from Statistics Denmark. Table: "UDDF" Variable: "hfaudd" "hfaudd" i linked with format from Satistics Denmark grouping the	Use the highest obtained education for the mother on the date of the child turns 10 years.<br>There is no information on this for children born 2007 or later.

Link to Contents

For peer review only - http://bmjopen.bmj.com/site/about/guidelines.xhtml

Link to Contents

	Danish education classification into ISCED 2011, based on which maternal education is grouped.<br><br>Link to mother is available from the dataset “population1” (originally obtained from the CPR register).	

Socio_economy Finland

Variable	Source and Description	Important notes and data preparation
id	Obtained from<br>Register: Population register<br>Table:<br>Variable: hetu<br>THL pseudonymised the original personal identification code (lapsi_hetunnus) to unique personal identification number for linkage between registers. THL data management can link the id back to original personal identification code.	Statistics Finland pseudonymised “hetu” with their own id for the remote user system.
inc_quin_b	Obtained from<br>Register: Statistics Finland<br>Table:<br>Variable: kturaha_ak_lapsi	Only available in Fiona remote user system.<br>Renamed from “kturaha_ak_lapsi” at the year when child was born.<br>Calculation of quintiles are done separately for each calendar year. E.g. calculating income quintiles for 2008 include all children who use income information from 2008 to assess the income quintile at birth<br>kturaha_ak_lapsi = NA, coded as 9 = "Unknown"
inc_quin_10y	Obtained from<br>Register: Statistics Finland<br><br>Table:<br>Variable: kturaha_ak_lapsi	Only available in Fiona remote user system.<br>Renamed from “kturaha_ak_lapsi” at the year when child was 10 years old.<br>Calculation of quintiles are done separately for each calendar year. E.g. calculating income quintiles for 2008 include all children who use income information from 2008 to assess the income quintile at birth<br>kturaha_ak_lapsi = NA, coded as 9 = "Unknown"
n_children_b	Obtained from<br>Register: Statistics Finland<br><br>Table:<br>Variable: lkm_lapsi	Only available in Fiona remote user system.<br>Renamed from “lkm_lapsi” at the year when child was born.
n_children_10y	Obtained from<br>Register: Statistics Finland<br><br>Table:	Only available in Fiona remote user system.<br>Renamed from “lkm_lapsi” at the year when child was 10 years old.

Link to Contents

For peer review only - http://bmjopen.bmj.com/site/about/guidelines.xhtml

Link to Contents

	Variable: lkm_lapsi	
Single_parent_b	Obtained from<br>Register: Statistics Finland<br><br>Table:<br>Variable: pety_lapsi	Only available in Fiona remote user system.<br>Calculated from “pety_lapsi” at the year when child was born.<br>If pety_lapsi is 2 (married couple and children) or 5-6 (couple with children) -> single parent =0 (no)<br>If pety_lapsi is 3 or 4 (mother or father with children) -> single parent = 1 (yes).<br>If pety_lapsi is unknown -> single parent = 9
Single_parent_10y	Obtained from<br>Register: Statistics Finland<br><br>Table:<br>Variable: pety_lapsi	Only available in Fiona remote user system.<br>Calculated from “pety_lapsi” at the year when child was 10 years old.<br>If pety_lapsi is 2 (married couple and children) or 5-6 (couple with children) -> single parent =0 (no)<br>If pety_lapsi is 3 or 4 (mother or father with children) -> single parent = 1 (yes).<br>If pety_lapsi is empty -> single parent = 9 (unknown)
m_education_b	Obtained from<br>Register: Statistics Finland<br><br>Table:<br>Variable:ututku_aiti and koulutusaste_taso_1 and birthday of child obtained from population1	Education is classified by ISCED-11, although the classes 0-2 are not available for us. In Finland, we have compulsory education during which the ISCED level 2 is achieved and thus we classified education:<br>NA= 1 low education level<br>3-4 = 2 medium education level<br>5-8 = 3 high education level<br>9 = no information of the mother’s Education at child’s birth year.
m_education_10y	Obtained from<br>Register: Statistics Finland<br><br>Table:<br>Variable: ututku_aiti and koulutusaste_taso_1 and birthday of child obtained from population1	Education is classified by ISCED-11. although the classes 0-2 are not available for us. In Finland, we have compulsory education during which the ISCED level 2 is achieved and thus we classified education:<br>NA= 1 low education level<br>3-4 = 2 medium education level<br>5-8 = 3 high education level<br>9 = no information of the mother’s Education when child is 10 years old.<br>If education was lower than m_education_b it was coded to be the same as at birth, also if education was unknown when child was ten, but it was known when child was born, the m_education_b was used as m_education_10y

Socio_economy Norway

Variable	Source and Description	Important notes and data preparation
id		Renamed from “pasientlopenr_pdb2471”

Link to Contents

For peer review only - http://bmjopen.bmj.com/site/about/guidelines.xhtml

Link to Contents

inc_quin_b	Obtained from Statistics Norway Variable: ies_eu	Based on the variable "ies_eu", defined as total after-tax income for the household per consumption unit calculated according to the EU scale. Total after-tax income is calculated as the sum of the household's wages and salaries, income from self-employment, property income and transfers received minus total assessed taxes and negative transfers. Each income year includes all persons residing in Norway and resident in a private household as of 31st December of the income year. Household income in year of birth is used to define inc_quin_b. Income quintiles are made separately for each birth cohort. Available for children born 2004–2018.
inc_quin_10y	Obtained from Statistics Norway Variable: ies_eu	Based on the variable "ies_eu", see definition above. Household income in the year of the child's 10th birthday is used to define inc_quin_10y. Income quintiles are made separately for each birth cohort. Available for children born 1994–2008.
inc_quin_m_b	Obtained from Statistics Norway Variable: wies	Based on the variable "wies", defined as a person's after-tax income. After-tax income is calculated as the sum of wages and salaries, income from self-employment, property income and transfers received minus total assessed taxes and negative transfers. The mother's income in the child's year of birth is used to define inc_quin_m_b. Income quintiles are made separately for each birth cohort. Available for children born 1993–2018.
inc_quin_m_10y	Obtained from Statistics Norway Variable: wies	Based on the variable "wies", see definition above. The mother's income in the year of the child's 10th birthday is used to define inc_quin_m_10y. Income quintiles are made separately for each birth cohort. Available for children born 1990–2008.
n_children_b	Obtained from Statistics Norway Variable: barn_i_regstat_famnr	Based on variable "barn_i_regstat_famnr", number of children in the family. Persons are considered children if they are below 18 years and registered as resident in the family of at least one parent. A family is defined as persons resident in the same dwelling and related to each other as spouse, registered partner, cohabitant, and/or parent and child (regardless of the child's age). At most, a family may consist of two subsequent generations and one couple only. The variable includes residents of Norway as of January 1 each year. We

Link to Contents

For peer review only - http://bmjopen.bmj.com/site/about/guidelines.xhtml

Link to Contents

		have therefore used number of children in the year after the child's year of birth. Individuals registered with 0 number of children in their family have been recoded to 1. Available for children born 2004–2018.
n_children_10y	Obtained from Statistics Norway Variable: barn_i_regstat_famnr	Based on variable "barn_i_regstat_famnr", see above. The variable includes residents of Norway as of January 1 each year. We have therefore used number of children in the year after the year of the child's 10th birthday. Individuals registered with 0 number of children in their family have been recoded to 1. Available for children born 1994–2009.
Single_parent_b	Obtained from Statistics Norway Variable: regstat_famtyp	Based on the variable "regstat_famtyp", a detailed classification of family type, where family is defined as described above. The variable includes residents of Norway as of January 1 each year. We have therefore used the value of family type in the year after a child's year of birth to define single_parent_b. If the registered family type is either "married couple with small children (youngest child aged 0-5 years)" or "cohabitants with small children (youngest child aged 0-5 years)", single_parent_b is coded as 0. If the registered family type is either "mother with small children (youngest child aged 0-5 years)" or "father with small children (youngest child aged 0-5 years)", single_parent_b is coded as 1. Otherwise (family type is any other category or missing), single_parent_b is coded as 9. Available for children born 2004–2018.
Single_parent_10y	Obtained from Statistics Norway Variable: regstat_famtyp	Based on the variable "regstat_famtyp", see above. The variable includes residents of Norway as of January 1 each year. We have therefore used the value of family type in the year after the year of a child's 10th birthday to define single_parent_10y. If the registered family type is either "married couple with small children (youngest child aged 0-5 years)", "married couple with older children (youngest child aged 6-17 years) "cohabitants with small children (youngest child aged 0-5 years)", or "cohabitants with older children (youngest child aged 6-17 years)", single_parent_10y is coded as 0. If the registered family type is either "mother with small children (youngest child aged 0-5 years)", "mother with older children (youngest child aged 6-17 years)", "father

Link to Contents

For peer review only - http://bmjopen.bmj.com/site/about/guidelines.xhtml

Link to Contents

		with small children (youngest child aged 0-5 years)", or "father with older children (youngest child aged 6-17 years)", single_parent_10y is coded as 1. Otherwise (family type is any other category or missing), single_parent_10y is coded as 9. Available for children born 1994–2009.
m_education_b	Obtained from Statistics Norway Variable: bu_niva_YYYY	Based on the variables "bu_niva_YYYY". The variables contain information on highest level of education as of October 1 of the year YYYY. Mother's level of education from the child's year of birth was used to define m_education_b. Education is classified according to The Norwegian Standard Classification of Education (NUS). If the NUS-level is 0 (corresponding to ISCED2011 levels 01, 02), 1 or 2 (corresponding to ISCED2011 level 1 and 2, respectively), m_education is coded as 1. If the NUS level is 3 or 4 (corresponding to ISCED2011 = 3), m_education is coded as 2. If the NUS-level is 6, 7 or 8 (corresponding to ISCED2011 level 6, 7, and 8, respectively), m_education is coded as 3 (https://www.ssb.no/utdanning/artikler-og-publikasjoner/_attachment/240569?_ts=150ebb996e0, page 25). NUS-level = 5 is defined as tertiary vocational educational level not approved as higher education. Tertiary education with duration less than 2 years corresponds to ISCED2011 level 4. In this case, m_educaion_b should be coded as 2. Tertiary education with duration of 2 years corresponds to ISCED2011 level 5, and m_education_b should be coded as 3. However, we do not have information on type or duration of the tertiary education. In 2016, 83.6% of women graduating from tertiary vocational education, had finished an education with duration of 2 years, while only 16.4% had finished an education with duration less than 2 years(https://www.ssb.no/en/statbank/table/11635). Therefore, m_education_b was coded as 2 if the NUS-level was 5. Available for children born 1990–2018.
m_education_10y	Obtained from Statistics Norway Variable: bu_niva_YYYY	Mother's highest level of education as of October 1 in the year of child's 10-year birthday was used to define m_education_b. For definitions and coding, see above. Available for children born 1990–2009.

Link to Contents

For peer review only - http://bmjopen.bmj.com/site/about/guidelines.xhtml

Link to Contents

Socio_economy Sweden

Variable	Source and Description	Important notes and data preparation
id	Created by Statistics Sweden<br>Pseudonomised unique personal identification number for linkage between registers<br>Variable: lopnr	Renamed from lopnr
inc_quin_b	Obtained from Statistics Sweden<br>Register: Longitudinell integrationsdatabas<br>för Sjukförsäkrings- och Arbetsmarknadsstudier (LISA)<br>Variable: DispInkFam	In Sweden, disposable income is defined as the sum of all household members' all forms of income (including wages, capital gains, and different forms of financial support/social assistance) minus taxes and other negative transfers (Statistikskolan: Att jämföra inkomster för hushåll (scb.se)).<br>The information *primarily* came from the information registered for the household of the mother in the year of birth of the child. If this was missing, the information was instead taken from the father. Thus, the child was primarily assumed to be part of the mother's household, and secondly of the father's.<br>Income quintiles was then calculated based on all children in each birth cohort.
inc_quin_10y	As above.	As above, but from the year the child turned 10 years old.
inc_quin_m_b	Obtained from Statistics Sweden<br>Register: Longitudinell integrationsdatabas<br>för Sjukförsäkrings- och Arbetsmarknadsstudier (LISA)<br>Variable: DispInk	Information about disposable income of the mother in the year of birth of the child.<br><br>Income quintiles was then calculated based on all children in each birth cohort.<br><br>See also above.
inc_quin_m_10y	As above.	As above, but from the year the child turned 10 years old.
n_children_b	Obtained from Statistics Sweden<br>Register: LISA<br>Variable: Barn0_3, Barn4_6, Barn7_10, Barn11_15, Barn16_17	Created as the sum of children in variables Barn0_3, Barn4_6, Barn7_10, Barn11_15 and Barn16_17. The sum denotes the number of children living in the household on 31 Dec in the year of birth of the child. The child itself is part of the count.<br><br>The information *primarily* came from the information registered for the mother in the year of birth of the child. If this was missing, the information was instead taken from the father.

Link to Contents

For peer review only - http://bmjopen.bmj.com/site/about/guidelines.xhtml

Link to Contents

n_children_10y	As above.	As above, but from the year the child turned 10 years old.
Single_parent_b	Obtained from Statistics Sweden<br>Register: LISA<br>Variable: FamTypF	The information came from the information registered for the mother in the year of birth of the child.<br><br>Codes FamTypF=41, 42 classifies the mother as a single parent, and 50 denotes Other singles. These codes were included when coding single_parent_b=1 (yes).<br>If FamTypF was missing, single_parent_b was coded as 9 (missing).<br>All other FamTypF-codes were recoded as 0 (no).
Single_parent_10y	As above.	As above, but from the year the child turned 10 years old.
m_education_b	Obtained from Statistics Sweden<br>Register: LISA<br>Variable: Sun2000niva	The variable denotes the highest level of education achieved during the spring semester in the year the child was born. That means, that if the mother achieved a higher level of education mid-year, it will only be visible in the register for the following year.<br>Level of education was recoded from Sun2000 to ISCED by a translational key available from Statistics Sweden: Svensk utbildningsnomenklatur (SUN) (scb.se) (retrieved 2021-08-20).
m_education_10y	As above.	As above, but from the year the child turned 10 years old.

Link to Contents

For peer review only - http://bmjopen.bmj.com/site/about/guidelines.xhtml

Link to *Contents*

Appendix: Vaccine categorization: presentation of ATC codes for vaccines identified in the vaccination registries in each of the Nordic countries and categorisation hereof into common vaccine categories "vaccine". The vaccines are further grouped by "type" i.e. vaccines against a similar set of diseases. NB in Sweden only vaccines that are included in the national immunisation programme is registered in the vaccination register.

		**DENMARK**	**Finland**	**Norway**	**Sweden**
Vaccine	Type	ATC Code			
1= "DTaP-IPV-Hib"	1	J07CA06	J07CA06	J07CA06	J07CA06
2= "DTaP-IPV-Hib-HepB"		J07CA09		J07CA09	J07CA09
3= "DTaP-IPV"		J07CA02<br>J07CA02	J07CA02<br>J07CA02	J07CA02<br>J07CA02	J07CA02
4= "DT-Pol"		J07CA01		J07CA01	
5= "DT-HepB"		J07CA07			
6= "DTwP-HepB"		J07CA05			
7= "DTwP-Hib-HepB"		J07CA11			
8= "DTaP-IPV-HepB"		J07CA12			J07CA12
10= "DTaP"		J07AJ52	J07AJ52	J07AJ52	J07AJ52
11= "DTwP"		J07AJ51		J07AJ51	
12= "DT"		J07AM51	J07AM51	J07AM51<br>J07AM52	J07AM51
13= "D"		J07AF01		J07AF01	J07AF01
15= "T"		J07AM01		J07AM01	J07AM01
20= "PCV"	2	J07AL02	J07AL02	J07AL52<br>J07AL02	J07AL52<br>J07AL02
21="PPV"		J07AL01	J07AL01	J07AL01	J07AL01

Link to *Contents*

For peer review only - http://bmjopen.bmj.com/site/about/guidelines.xhtml

Link to *Contents*

25="HepA"	3	J07BC02	J07BC02	J07BC02	
26= "HepAB"		J07BC20	J07BC20	J07BC20	
27= "HepB"		J07BC01	J07BC01	J07BC01	
28= "HepA-Thyphoid"		J07CA10		J07CA10	
30= "HPV4"	4	J07BM01		J07BM01	J07BM01
31="HPV2"		J07BM02	J07BM02	J07BM02	J07BM02
32="HPV9"		J07BM03	J07BM03	J07BM03	J07BM03
35= "Hib"	5	J07AG01	J07AG01	J07AG01	J07AG01
36= "Hib-MenC"		J07AG53			
37= "Hib-Pol"				J07CA04	
38= "Hib-HepB"		J07CA08			
40= "Influenza (non-live)"	6	J07BB01<br>J07BB02	J07BB02	J07BB01<br>J07BB02	
41= "Influenza (live)"		J07BB03	J07BB03	J07BB03	
45= "wP"	7	J07AJ01		J07AJ01	
46= "aP"		J07AJ02		J07AJ02	
50= "MMR"	8	J07BD52	J07BD52	J07BD52	J07BD52
51= "MMR-Varicella"		J07BD54	J07BD54		
52= "Measles"		J07BD01		J07BD01	
53= "Measles-Mumps"		J07BD51		J07BD51	
54= "Measles-Rubella"		J07BD53		J07BD53	
55= "Rubella"		J07BJ01		J07BJ01	
56= "Mumps"		J07BE01		J07BE01	
60= "OPV"	9	J07BF01		J07BF04<br>J07BF01	

Link to *Contents*

Link to Contents

		J07BF02		J07BF02	
61= "IPV"		J07BF03	J07BF03	J07BF03	J07BF03
65= "Rota"		J07BH01<br>J07BH02	J07BH02	J07BH01<br>J07BH02	
67= "BCG"		J07AN01	J07AN01	J07AN01	
70="Varicella"		J07BK01<br>J07BK02	J07BK01	J07BK03<br>J07BK02<br>J07BK01	
71= "yellow fever"		J07BL01	J07BL01	J07BL01	
72= "Japanease Encephalitis"		J07BA02	J07BA02	J07BA02	
73= "Tick borne Encephalitis"		J07BA01	J07BA01	J07BA01	
74 = "Cholera"		J07AE51<br>J07AE02<br>J07AE01	J07AE01	J07AE01	
75= "Meningococcal vaccine"		J07AH08<br>J07AH09<br>J07AH03<br>J07AH04<br>J07AH06<br>J07AH05<br>J07AH02<br>J07AH01	J07AH08<br>J07AH09	J07AH07<br>J07AH08<br>J07AH09<br>J07AH03<br>J07AH04<br>J07AH06<br>J07AH01	
76= "Typhus"		J07AP01<br>J07AP10	J07AP01	J07AP01<br>J07AP02<br>J07AP	

Link to Contents

Link to Contents

		J07AP03		J07AP03	
77= "Rabies"		J07BG01		J07BG01	
78= "Smallpox"				J07B01	
		J07BX01			
79="Anthrax"				J07AC01	
80="covid-19 vaccine"			J07BX03	J07BX03	
99="other vaccines"		ATC code missing	ATC code missing	ATC code missing	ATC code missing

For peer review only - http://bmjopen.bmj.com/site/about/guidelines.xhtml

Online supplementary files

sTable 1: Vaccination coverage[1] at 2 years of age according to year of birth among children born in the respective countries

sTable2: Human papilloma virus vaccination coverage[1] before 14 years of age of vaccination among girls[2] born in the respective countries

sTable 3: Socio-economic factors at 10 years of age

Appendix 3: ATC codes obtained for the study population in each country within NONSEnse

For peer review only

sTable 1: Vaccination coverage[1] at 2 years of age according to year of birth among children born in the respective countries

**Denmark**						
Year of birth	Eligible[2] Children	DTP1 % (95% CI)	DTP2 % (95% CI)	DTP3 % (95% CI)	MMR1 % (95% CI)	
1997	66,406	98.9	96.0	82.2	81.5	
1998	64,936	99.0	97.0	85.0	83.6	
1999	64,996	99.0	97.0	84.9	84.5	
2000	65,811	99.0	97.3	86.2	85.9	
2001	64,207	99.1	97.4	86.4	85.5	
2002	62,948	99.1	97.0	84.1	84.9	
2003	63,462	98.9	96.6	82.5	85.2	
2004	63,339	98.9	96.5	82.7	86.8	
2005	62,912	98.9	96.0	80.1	84.8	
2006	63,769	99.0	95.8	78.7	84.4	
2007	63,006	99.0	96.4	80.9	82.6	
2008	63,892	99.2	97.2	83.9	83.7	
2009	61,676	99.3	97.6	86.6	86.2	
2010	62,200	99.2	97.9	88.8	87.3	
2011	57,892	99.2	98.0	89.7	86.7	
2012	56,842	99.2	98.0	90.0	86.4	
2013	54,881	98.9	97.6	88.5	88.3	
2014	55,753	98.8	97.5	87.6	88.4	
2015	57,100	98.9	98.0	93.2	90.3	
2016	23,103	99.0	98.3	94.7	90.7	
**Finland**						
Year of birth	Eligible[2] Children	DTP1 % (95% CI)	DTP2 % (95% CI)	DTP3 % (95% CI)	MMR1 % (95% CI)	Rota virus vaccine % (95% CI)
2009	59,934	94.0	92.7	89.4	87.8	63.8
2010	60,560	96.7	94.2	90.8	91.6	90.7
2011	59,645	97.0	95.1	90.4	92.3	90.8
2012	59,309	95.7	94.5	90.3	91.9	91.2
2013	58,249	97.5	95.7	91.8	93.3	90.4

For peer review only

2014	57,693	97.6	96.5	89.3	92.6	91.6
2015	55,569	98.0	96.6	88.1	93.4	92.1
**Norway**						
Year of birth	Eligible[2] Children	DTP1 % (95% CI)	DTP2 % (95% CI)	DTP3 % (95% CI)	MMR1 % (95% CI)	Rota virus vaccine % (95% CI)
1995	59964	98.5	97.9	95.8	94.7	
1996	60652	98.2	97.4	95.4	94.3	
1997	59431	98.5	98.0	96.2	94.4	
1998	57999	98.7	98.2	96.4	94.1	
1999	58975	98.8	98.3	96.4	94.1	
2000	58907	98.6	98.0	96.1	89.2	
2001	56405	98.7	98.3	96.1	89.3	
2002	55232	98.8	98.5	96.7	92.7	
2003	56301	99.0	98.6	96.8	94.2	
2004	56734	99.1	98.8	97.5	94.7	
2005	56531	99.2	99.0	97.6	94.4	
2006	58316	99.1	98.8	97.3	94.2	
2007	58199	99.0	98.6	96.7	94.0	
2008	60284	99.0	98.5	96.6	93.8	
2009	61465	98.9	98.6	97.4	94.4	
2010	61080	98.9	98.5	97.3	95.0	
2011	59855	98.8	98.4	97.2	94.8	
2012	59937	98.6	98.2	96.6	95.0	
2013	58745	98.6	98.0	96.5	95.5	
2014	58839	98.7	98.3	96.6	95.9	
2015	58954	98.6	98.1	96.6	95.7	94.1
2016	58975	98.5	97.8	96.3	95.9	94.8
**Sweden**						
Year of birth	Eligible[2] Children	DTP1 N (%)	DTP2 N (%)	DTP3 N (%)	MMR1 (%)	
2013	113,457	97.6	95.1	83.3	89.1	
2014	114,639	98.0	95.9	86.1	90.7	
2015	114,542	98.1	96.3	87.8	91.8	

For peer review only

Abbreviations: DTP1: First dose of Diphtheria, Tetanus, and acellular Pertussis containing vaccine; DTP2: Second dose of Diphtheria, Tetanus, and acellular Pertussis containing vaccine; DTP3: Third dose of Diphtheria, Tetanus, and acellular Pertussis containing vaccine; MMR: Measles-Mumps-Rubella vaccine; Rota: Rota virus vaccine.

[1]The coverage reflects the number of registered vaccines and may thus underestimate the actual vaccination coverage in the countries. [2] Including children born in the country from birth cohorts where vaccines administered between 0-2 years of age are registered in the vaccination registers (data availability period).

For peer review only

sTable 2: Human papilloma virus vaccination coverage[1] before 14 years of age of vaccination among girls[2] born in the respective countries

	Denmark			Finland			Norway			Sweden		
Year of birth	Eligible [3] children	HPV1 vaccinated N (%)	HPV2 vaccinated N (%)	Eligible[3] children	HPV1 vaccinated N (%)	HPV2 vaccinated N (%)	Eligible [3] children	HPV1 vaccinated N (%)	HPV2 vaccinated N (%)	Eligible [3] children	HPV1 vaccinated N (%)	HPV2 vaccinated N (%)
1998	34,392	85.7	81.2				30,914	76.5 (76.0, 77.0)	75.8			
1999	34,484	86.8	82.4				31,391	78.5	77.7			
2000	34,881	86.5	82.3				31,490	80.2	79.5 (79.0, 79.9)			
2001	34,030	81.4	74.5				30,546	82.7	82.0			
2002	33,241	73.6	57.6	4053	71.1	69.3	30,213	84.5	83.7			
2003	33,762	52.3	36.1	27,310	69.8	67.2	30,925	84.8	84.0	53,623	77.9	72.4
2004	13,184	58.4	43.0				31,208	86.8	85.8			

Abbreviations: HPV1: First dose of Human papilloma virus vaccine; HPV2: Second dose of Human papilloma virus vaccine

[1]The coverage reflects the number of registered vaccines and may thus underestimate the actual vaccination coverage. [2] Including girls from birth cohorts where HPV vaccination has been offered from 1 year before age of recommended vaccination until 14 years of age and where vaccinations were registered in the vaccination registers. The years with available data is defined based on introduction of HPV vaccinations into the National immunization programme or introduction of vaccination register whichever comes last until last date with available data from both the population register and vaccination register.

For peer review only

sTable 3: Socio-economic factors at 10 years of age

	Denmark		Finland		Norway		Sweden	
	N	(%)	N	(%)	N	(%)	N	(%)
Children present in country at birth from 2004-2015	793,471		687,721		726,257		1,205,112	
Birth cohorts included	1994-2005		1994-2005		1994-2005		1994-2005	
Income quintile at 10 years of age								
First (lowest)	147,098	18.5%	135,946	19.8%	141,762	19.5%	219,673	18.2%
Second	149,579	18.9%	136,334	19.8%	146,247	20.1%	241,503	20.0%
Third	149,865	18.9%	136,384	19.8%	146,604	20.2%	245,097	20.3%
Fourth	149,310	18.8%	136,259	19.8%	146,471	20.2%	245,138	20.3%
Fifth	146,712	18.5%	135,464	19.7%	144,939	20.0%	241,452	20.0%
Unknown	50,907	6.4%	7334	1.1%	234	0.0%	12,249	1.0%
Number of children in the household the year the child turns 10 years of age								
1	103,585	13.1%	96,861	14.1%	119,295	16.4%	162,968	13.5%
2	405,367	51.1%	293,068	42.6%	336,480	46.3%	587,332	48.7%
3	213,413	26.9%	184,925	26.9%	208,045	28.6%	292,516	24.3%
>3	61,674	7.8%	103,561	15.1%	62,203	8.6%	105,595	8.8%
Unknown	9432	1.2%	9306	1.4%	234	0.0%	12,249	1.0%
Single parenthood in the years the child turns 10 years of age								
Yes	151,471	19.1%	124,986	18.2%	131,761	18.1%	268,484	22.3%
No	632,568	79.7%	553,429	80.5%	587,793	80.9%	924,379	76.7%
Unknown	9432	1.2%	9306	1.4%	6703	0.9%	12,249	1.0%
Highest attained educational level[1] of the mother on the date the child turns 10 years of age								
Low education	135,466	17.1%	75,462	11.0%	138,351	19.0%	193,551	16.1%
Medium education	340,574	42.9%	281,479	40.9%	270,114	37.2%	515,407	42.8%
High education	303,384	38.2%	329,885	48.0%	305,368	42.0%	394,220	32.7%
Unknown	14,047	1.8%	895	0.1%	12,424	1.7%	101,934	8.5%

[1] Highest attained education was categorized based on the International Standard Classification of Education (ISCED) 2011 using the main groups (1).

For peer review only

Appendix 3: ATC codes obtained for the study population in each country within NONSEnse

ATC-Group	Denmark	Finland	Norway	Sweden
D	D	D07, D11AH	D	D02AF, D05<br>D07, D11<br>D01, D06, D08
J	J	J	J	J01-J06<br>J07
R	R	R01, R03, R06	R	R01, R03, R06
S	S	S01G, S03	S	S01-S03
V	V01	V01[1]	V01	V01
[1] Data on redeemed prescriptions with ATC=V01 is only available from the Finnish Benefits Registry, which holds information only for reimbursable redeemed prescriptions.				

References

1. UNESCO Institute for Statistics. International standard classification of education: ISCED 2011. 2012.

For peer review only

BMJ Open

Cohort Profile: Childhood morbidity and potential non-specific effects of the childhood vaccination programmes in the Nordic countries (NONSEnse): Register-based cohort of children born 1990-2017/2018.

Journal:	*BMJ Open*
Manuscript ID	bmjopen-2022-065984.R1
Article Type:	Cohort profile
Date Submitted by the Author:	01-Dec-2022
Complete List of Authors:	Gehrt, Lise; University of Southern Denmark, Research Unit OPEN<br>Laake, Ida; Norwegian Institute of Public Health, Division of Infection Control and Environmental Health<br>Englund, Hélène; Folkhälsomyndigheten<br>Nieminen, Heta; Finnish Institute for Health and Welfare, Department of Public Health and Welfare<br>Feiring, Berit; Norwegian Institute of Public Health, Division of Infection Control and Environmental Health<br>Lahdenkari, Mika; Finnish Institute for Health and Welfare, Department of Information Services<br>Palmu, Arto A.; Finnish Institute for Health and Welfare, Department of Public Health and Welfare,<br>Trogstad, Lill; Norwegian Institute of Public Health, Division of Infection Control and Environmental Health<br>Benn, Christine Stabell ; University of Southern Denmark, Bandim Health Project, Research unit Open, Department of Clinical Research, ; University of Southern Denmark, Danish Institute for Advanced Study<br>Sørup, Signe; Aarhus University Hospital, Department of Clinical Epidemiology
**Primary Subject Heading**:	Epidemiology
Secondary Subject Heading:	Immunology (including allergy), Infectious diseases, Paediatrics
Keywords:	Epidemiology < INFECTIOUS DISEASES, Paediatric infectious disease & immunisation < PAEDIATRICS, PUBLIC HEALTH

SCHOLARONE™
Manuscripts

I, the Submitting Author has the right to grant and does grant on behalf of all authors of the Work (as defined in the below author licence), an exclusive licence and/or a non-exclusive licence for contributions from authors who are: i) UK Crown employees; ii) where BMJ has agreed a CC-BY licence shall apply, and/or iii) in accordance with the terms applicable for US Federal Government officers or employees acting as part of their official duties; on a worldwide, perpetual, irrevocable, royalty-free basis to BMJ Publishing Group Ltd ("BMJ") its licensees and where the relevant Journal is co-owned by BMJ to the co-owners of the Journal, to publish the Work in this journal and any other BMJ products and to exploit all rights, as set out in our licence.

The Submitting Author accepts and understands that any supply made under these terms is made by BMJ to the Submitting Author unless you are acting as an employee on behalf of your employer or a postgraduate student of an affiliated institution which is paying any applicable article publishing charge ("APC") for Open Access articles. Where the Submitting Author wishes to make the Work available on an Open Access basis (and intends to pay the relevant APC), the terms of reuse of such Open Access shall be governed by a Creative Commons licence – details of these licences and which Creative Commons licence will apply to this Work are set out in our licence referred to above.

Other than as permitted in any relevant BMJ Author's Self Archiving Policies, I confirm this Work has not been accepted for publication elsewhere, is not being considered for publication elsewhere and does not duplicate material already published. I confirm all authors consent to publication of this Work and authorise the granting of this licence.

For peer review only

Title

Cohort Profile: Childhood morbidity and potential non-specific effects of the childhood vaccination programmes in the Nordic countries (NONSEnse): Register-based cohort of children born 1990-2017/2018.

Author list

Lise Gehrt[1,2]; Ida Laake[3]; Hélène Englund[4]; Heta Nieminen[5]; Berit Feiring[3]; Mika Lahdenkari[6]; Arto A. Palmu[5]; Lill Trogstad[3]; Christine Stabell Benn[1,2]; Signe Sørup[1,7]

[1]Bandim Health Project, Research unit Open, Department of Clinical Research, University of Southern Denmark, Odense C, Denmark
[2] Danish Institute for Advanced Study, University of Southern Denmark, Odense C, Denmark
[3]Division of Infection Control, Norwegian Institute of Public Health Oslo, Norway
[4]Public Health Agency of Sweden, Solna, Sweden
[5]Department of Public Health and Welfare, Finnish Institute for Health and Welfare, Tampere, Finland
[6] Department of Information Services, Finnish Institute for Health and Welfare, Helsinki, Finland
[7]Department of Clinical Epidemiology, Aarhus University Hospital, Aarhus, Denmark

Correspondence

Hélène Englund, Public Health Agency of Sweden, Solna, Sweden
E-mail: helene.englund@folkhalsomyndigheten.se

For peer review only

ABSTRACT

Purpose: The aim of the NONSEnse project is to investigate the non-specific effects of vaccines and immunisation programmes on the overall health of children by using information from the extensive nationwide registers on health and sociodemographic factors in Denmark, Finland, Norway, and Sweden.

Participants: The cohort covers 9,072,420 children aged 0-17 years, born 1990-2017/2018, and living in Denmark, Finland, Norway, or Sweden. All countries utilise a unique identification number for its permanent residents, which makes it possible to link individual-level information from different registers.

Findings to date: Data collection and harmonisation according to a Common Data Model was completed in March 2022. As a prerequisite for comparing the effects of childhood vaccinations on the overall health of children across the Nordic countries, we have identified indicators measuring similar levels of infectious disease morbidity across these settings. So far studies pertaining to non-specific effects of vaccines are limited to investigations that could be undertaken using aggregated datasets that were available before the NONSEnse cohort with individual level information was completely setup.

Future plans: We are currently performing several studies of the effects on non-targeted infectious disease morbidity across the countries following vaccination against measles, mumps, rubella, diphtheria, tetanus, pertussis, human papilloma virus, rotavirus, and influenza. Multiple studies are planned within the next years using different study designs to facilitate triangulation of results and enhance causal inference.

Registration: No clinical trials will be conducted within the NONSEnse project.

For peer review only - http://bmjopen.bmj.com/site/about/guidelines.xhtml

STRENGHTS AND LIMITATIONS

Strengths:

- Complete population cohort minimizes selection bias.
- Real world data which has been collected, collated and quality checked.
- A Common Data Model enables uniform data analysis across countries.

Limitations:

- lacking information on some potential confounding factors.
- The data harmonisation process may entail loss of details of country-specific data.

For peer review only

For peer review only - http://bmjopen.bmj.com/site/about/guidelines.xhtml

INTRODUCTION

An accumulating number of epidemiological and immunological studies have found that vaccines, in addition to the disease-specific protection, may have so called non-specific effects affecting susceptibility towards other diseases than the vaccine-targeted infections (1, 2). Most previous studies on non-specific effects stem from low-income countries with a high infectious disease burden and have had overall childhood mortality as the outcome. The non-specific effects are found to vary depending on the type of vaccine being administered. Live vaccines have been associated with beneficial non-specific effects (1, 2). Non-live vaccines, although protecting against the vaccine-targeted infections, may possibly increase susceptibility to other infections (1, 2). The effects are most pronounced for the most recently administered vaccine (2).

Studies of non-specific effects from high-income countries have primarily focused on infectious disease morbidity (3) and atopic diseases (4-8). Most of these studies are observational, because it would (often) be unethical to randomise children to refrain from or delay recommended childhood vaccinations. Therefore, concerns about different types of bias in different settings and observational designs have been raised (9-12). Triangulation has been proposed as a method to strengthen causal inference in epidemiology by integrating results from several epidemiological designs and between different populations with different bias structures while using the same analysis plan across settings to enhance comparability of results (13, 14).

The “NONSEnse” project is a Nordforsk-funded collaboration between research groups in the four Northern European countries Denmark, Finland, Norway, and Sweden (henceforth referred to as the Nordic countries. The main aim of NONSEnse is to evaluate if childhood vaccinations influence other health outcomes than those targeted by the vaccine in the Nordic countries. The main hypothesis underlying this evaluation is that having a live vaccine as the most recent vaccine is associated with beneficial non-specific effects and thus a lower morbidity in the following time-period, compared with having a non-live vaccine as the most recent vaccine. The individual studies will be undertaken using the same methodology and statistical coding across countries. Furthermore, we will examine the same research question in multiple studies using different analytical approaches to facilitate triangulation of the results. The main associations we will examine are

For peer review only - http://bmjopen.bmj.com/site/about/guidelines.xhtml

associations between childhood vaccinations and 1) infectious disease hospitalisations, 2) antibiotic use, and 3) atopic diseases (asthma, atopic dermatitis, allergic rhinoconjunctivitis).

The first step has been to examine and compare infectious disease and atopic morbidity among children in the respective countries over time and by age and sex, to inform choice of design and outcome definitions in the subsequent studies of non-specific effects of vaccines.

The aim of the present cohort profile is to describe the content and quality of the data included in the registry-based NONSEnse cohort and present characteristics of the cohort, thereby demonstrating the research potential of the NONSEnse cohort. The insights presented can be used to guide future epidemiological research projects using registry data from the Nordic countries.

COHORT DESCRIPTION

Setting

The Nordic countries have many similarities including the welfare state model with universal tax-funded healthcare and a high level of social security. A detailed description of the Nordic health care systems and basic demographics has been published elsewhere (15).

National immunisation programmes

Childhood vaccinations within the national immunisation programmes (NIP) are voluntary and administered free of charge in all four countries. In Denmark, all childhood vaccines are administered by family practitioners (16). In Finland, Norway, and Sweden vaccines scheduled before school-age are administered at well-baby clinics by nurses; during school age, the vaccines are administered by school nurses (17-19). In 2018, children were offered vaccinations against 10 diseases in Denmark (16), up to 13 diseases in Finland (17), 12 diseases in Norway (19), and 10 diseases in Sweden (18). Children in specific risk groups are offered vaccines against additional diseases according to national guidelines (17-20). An overview of recommended childhood vaccinations in the four

For peer review only - http://bmjopen.bmj.com/site/about/guidelines.xhtml

countries in 2018 is presented in Table 1 and historical changes are illustrated in Appendix 1.

Table 1: Vaccines recommended to children in Denmark, Finland, Norway, and Sweden in 2018. The vaccines are included in the childhood immunisation programmes and registered in the vaccination registers, unless otherwise specified.

Disease (Vaccine)	Denmark	Finland	Norway	Sweden
**Tuberculosis (BCG)**	Not within programme	Before 7 years of age, risk groups only[1]	6 weeks of age, risk groups only[1]	After 6 months of age, risk groups only [1,2]
**Hepatitis A**	Not within programme	From 1 year of age, risk groups only[3]	Not within programme	Not within programme
**Hepatitis B**	From birth, risk groups only[4]	From birth, risk groups only[4,5]	3 doses: 3, 5, 12 months of age	Not within programme but recommended to all children.<br>3 doses: 3, 5, 12 months of age[6]
**Rotavirus**	Not within programme	3 doses: 2, 3, 5 months of age	2 doses: 6 weeks, 3 months of age	2 or 3 doses:<br>6 weeks, 3 and 5 months of age[2, 7]
**Diphtheria, tetanus and pertussis (DTaP)**	4 doses: 3, 5, 12 months, booster at 5 years of age	5 doses: 3, 5, 12 months of age, booster at 4 and 14 years of age	5 doses: 3, 5, 12 months of age, Booster in 2nd and 10th school-year	5 doses: 3, 5, 12 months of age, booster at 5 years of age and in 8th or 9th school-year
**Polio (IPV)**	4 doses: 3, 5, 12 months, booster at 5 years of age	4 doses: 3, 5, 12 months of age, booster at 4 years of age	5 doses: 3, 5, 12 months of age, booster in 2nd and 10th school-year	4 doses: 3, 5, and 12 months of age, booster at 5 years of age

For peer review only - http://bmjopen.bmj.com/site/about/guidelines.xhtml

**Haemophilus influenzae type B**	3 doses: 3, 5, 12 months of age	3 doses: 3, 5, 12 months of age	3 doses: 3, 5, 12 months	3 doses: 3, 5, and 12 months of age
**Pneumococcal disease (PCV)**	13-valent; 3 doses: 3, 5, 12 months of age	10-valent; 3 doses: 3, 5, 12 months of age	13-valent; 3 doses: 3, 5, 12 months of age	10 or 13-valent; 3 doses: 3, 5, and 12 months of age
**Influenza (Live- or non-live influenza vaccine)**	From 6 months of age, risk groups only[8]	Yearly, from 6 months to 6 years of age and for risk groups after 6 years of age[8]	From 6 months of age, risk groups only, through the influenza immunisation programme[8]	Yearly, from 6 months of age, risk groups only[2,8]
**Measles, mumps and rubella**	2 doses: 15 months of age and 4 years of age	2 doses: 12 months of age, 6 years of age	2 doses: 15 months of age, and 6th school-year	2 doses: 18 months of age and 1st or 2nd school-year
**Varicella**	Not recommended	1.5-11 years of age	Not recommended	Not recommended
**Pneumococcal disease (PPV)**	Not within programme	Before 5 years of age, after PCV, risk groups only[9]	Not within programme, but recommended from 2 years of age, to specified risk groups[9]	Not within programme, but recommended from 2 years of age, to specified risk groups[2,9]
**Tick borne encephalitis**	Not within programme	From 3 years of age, risk groups only[10]	Not within programme	Not within programme
**Human papilloma virus**	2 doses: 12 years of age, girls only	2 doses: 6th school-year, girls only	2 doses in 7th school-year	2 doses in 5th or 6th school-year, girls only

Abbreviations: BCG: Bacillus Calmette-Guérin vaccine; DTaP: Diphtheria, tetanus, and acellular pertussis vaccine; IPV: Inactivated Polio Vaccine; PCV: Pneumococcal Conjugated Vaccine; PPV: Pneumococcal Polysaccharide Vaccine.

For peer review only - http://bmjopen.bmj.com/site/about/guidelines.xhtml

Information obtained from: The Danish health authority (16), Finnish institute for health and Welfare (17), Norwegian Institute of Public Health (19), The Public Health Agency of Sweden (18).
[1] Children with a parent from a country with a high incidence of tuberculosis. [2] Not included in the vaccination registry, [3] Children of intravenous drug users. [4] 1) Children of mothers or another member of the household who are Hepatitis B positive, or 2) attend daycare with a child who has Hepatitis B (20, 21). [5] 1) children of parents from countries with high incidence of Hepatitis B, or 2) children of mothers with hepatitis C infection (21). [6] Only offered to children in the risk group before 2016, not included in the vaccination registry before 2016 (22). [7] Rotavirus vaccine was offered by some Swedish regions, as part of regional vaccination schemes.[8] Children with increased risk of severe influenza illness or members of households with high-risk individuals(18, 23-25). [9] Children with increased risk of severe pneumococcal disease e.g. children with chronic diseases (18, 26, 27). [10] Children of families with a permanent home or holiday house in areas within Finland with high tick prevalence (28)

Nordic nationwide register data: a goldmine for epidemiological studies

All individuals residing in the Nordic countries are assigned a unique personal identification code (ID). All four countries have extensive national registers on health, demographic factors, and socio-economic factors collected for administrative purposes and linked to the individual using the personal ID (15, 29). The register information is collected automatically, which minimizes systematic reporting bias e.g. recall bias. The use of national registers limits selection bias as the entire population is included. All information in the registers are dated, which ensures that exposures and outcomes can be temporally linked and facilitates investigation of the cumulative and combined effects of multiple interventions on childhood health. Thus, the structure of the Nordic registers presents a unique opportunity to investigate the real-life effects of childhood vaccinations while incorporating multiple potential confounding factors.

Study population

We used national population registers to identify all children aged 0-17 years, who were born or became permanent residents after migrating to one of the Nordic countries at some point from 1990 until and including 2018 in Denmark and Norway, and 2017 in Finland and Sweden (30-33) (Figure 1). End of follow-up in each country reflects when the data application process was final. The individual registries included in this cohort were established in the respective countries at different time points. We have included the birth cohorts from 1990 in all countries to ensure that we have full information on follow-up from

For peer review only - http://bmjopen.bmj.com/site/about/guidelines.xhtml

birth also for the children who will be included at older ages for e.g., the studies of HPV vaccination given to teenagers. The population data obtained in Finland had incomplete information on migration history before 2014 and thus we were unable to assess the date of entering the country for children born abroad. As a result, we limited the Finnish study population to children born in the country to ensure that they were present in the country from the beginning of follow-up. After exclusions, which were primarily due to uncertain information about residency, a total of 9,072,420 children were included across the countries (Figure 1). Children were followed from date of birth or date of immigration until the date of first emigration, 18-year birthday, death, or last date with available information, whichever came first.

Source and content of data

Using the personal ID, we linked information from the nationwide registers and obtained individual-level information on gestation and birth, hospital contacts, redeemed prescriptions, and receipt of childhood vaccines. Furthermore, each child was linked to their parents through the population registers in order to extract information on household income, family composition, and highest attained parental education (Figure 2). The included data reflects necessary information to identify the vaccination status of the child, relevant outcomes, potential confounding factors, and information to be included as negative control outcomes.

Information on administered vaccines including type of vaccine and date of vaccination was obtained from The Danish Vaccination Register in Denmark (34), the Finnish vaccination register in Finland (35), the Norwegian Immunisation Registry (SYSVAK) in Norway (36), and the National vaccination register in Sweden (37). Registration of vaccinations within the NIP is mandatory in all Nordic countries (Table 1).

The Danish Vaccination Register includes information from the Danish National Health Insurance System that collects information on all vaccinations within the NIP (38). Since 2015, it has also been mandatory to report on vaccines given outside the NIP (39). In Denmark, vaccine information is linked to the individual using the personal ID, however before 1997 the information was registered on the ID of the parents only (38). Thus, in Denmark, only information on vaccines administered from 1997 and later was included. In Finland, the register includes all vaccines given in public health care since 2009, and after 2016 also private health care is obligated to register vaccinations (35). In Norway, the

For peer review only - http://bmjopen.bmj.com/site/about/guidelines.xhtml

immunisation registry holds information since 1995 on all administered vaccines that are part of the NIP (19). Since 2011 notification to the immunisation registry is also mandatory for vaccines given outside the NIP (36). The Swedish national vaccination register has information about vaccinations given since 2013, but only those included in the NIP (37).

Information on hospital contacts was obtained from The Danish National patient register, Finnish Care Register for Health Care, Norwegian National Patient Register, and The Swedish Patient Registry (15, 29). The registries reached national coverage and recorded individual-level data since 1978 in Denmark, 1994 in Finland, 2008 in Norway, and 1997 in Sweden. Since 1997, diagnoses have been coded according to the International Classification of Diseases version 10 (ICD-10) in all four countries (40).

The Danish, Norwegian, and Swedish prescription registers hold information on all redeemed prescriptions, classified using the Anatomical Therapeutic Chemical Classification System (ATC) since 1995, 2004, and mid-2005, respectively (41). The Finnish Benefits Registry holds information only for reimbursable redeemed prescriptions (41-43). In addition, the Finnish Prescription Center started gradually in 2010 and collects all redeemed prescriptions irrespective of reimbursement. By 2017, practically all prescriptions were included in the Finnish Prescription Center (44). We combined the information from the Finnish Prescription Center and the Finnish Benefits Registry to obtain the most complete information on redeemed prescriptions (see Appendix 2 for details on source of data).

Information on socioeconomic factors and birth characteristics was available from the beginning of the study period (1990) in all countries.

The Common Data Model: Harmonised country specific datasets

The country-specific data from the national registers may differ both across countries and within countries over time due to differences in coding practices, administration, and country-specific legislation on health and social aspects (29). We developed a Common Data Model to harmonise all information we obtained into similar datasets using the same variable names and same categories in all four countries (Figure 3). The data harmonisation focused on identifying outliers and country-specific traits that could hinder cross country comparability. Information on source of data and data preparation for each of the variables can be found in Appendix 2 “NONSense Common Data Model”.

For peer review only - http://bmjopen.bmj.com/site/about/guidelines.xhtml

Due to national data protection legislation, country-specific data was stored and analysed in the respective countries using platforms that adhere to country specific regulations to ensure safe storing and handling of data. Country specific data was pseudonymized by the registry holders before being transferred to the research team in each country. The common data model allows for the exchange of aggregated or summary data between countries, thus precluding the need to set up separate platforms to exchange data.

Patient and Public involvement

All studies conducted within NONSEnse will be register based studies only and patients or the public will not be involved in the design or conduct of the planned studies.

Characteristics of the study population

The national study populations range from 1,637,133 children in Finland to 3,540,560 children in Sweden (Table 2). Median follow-up time was 13.1 years in Denmark, 14.2 years in Finland, 12.6 years in Norway, and 10.8 years in Sweden. Sweden had the highest proportion of children born abroad; 15.5% compared with 8.4 in Denmark and 11.1% in Norway. The proportion of children who were censored due to migration was lower in Finland, where we only included children born in-country: 0.7% compared with 4.4-6.2% in the other countries. The lower emigration rate in Finland represents both underreporting due to incomplete information on migration, and a suspected lower risk of moving out of the country for children born in-country, compared with children born abroad. A higher proportion of children without a link to their mother was seen in Sweden; 5.3% compared to 0.2-1.1% in the other countries. The children without a link to their mother in Sweden were predominantly born abroad (data not presented) and may thus be affected by incomplete registration of migrant families, or children immigrating to Sweden without their mother.

Table 2: Study population – identification and follow-up

	Denmark	Finland[1]	Norway	Sweden
**Study population (N)**	1,979,670	1,637,133	1,915,057	3,540,560
**Years of follow-up[2] per child (Median (p25-p75))**	13.1 (5.9-18.0)	14.2 (7.2-18.0)	12.6 (5.7-18.0)	10.8 (4.2-18.0)
**Year of birth**	1990-2018	1990-2017	1990-2018	1990-2017

For peer review only - http://bmjopen.bmj.com/site/about/guidelines.xhtml

**Sex (N (%))**								
Male	1,014,745	51.3%	836,828	51.1%	985,568	51.5%	1,827,619	51.6%
Female	964,925	48.7%	800,305	48.9%	929,489	48.5%	1,712,941	48.4%
**Reason for entering the cohort (N (%))**								
Birth	1,813,443	91.6%	1,637,133	100.0%	1,703,054	88.9%	2,993,472	84.5%
Immigration	166,227	8.4%	0	0.0%	212,003	11.1%	547,088	15.5%
**Reason for leaving the cohort (N (%))**								
Death	8532	0.4%	761	0.0%	5422	0.3%	5614	0.2%
Emigration	122,916	6.2%	11,789	0.7%	95,406	5.0%	154,878	4.4%
Other[3]	1917	0.1%	0	0.0%	0	0.0%	0	0.0%
18th birthday	704,518	35.6%	608,644	37.2%	703,164	36.7%	1,280,027	36.2%
End of follow-up[4]	1,141,787	57.7%	1,015,939	62.1%	1,111,065	58.0%	2,100,041	59.3%
**Linked with mother in registers (N (%))**	1,961,595	99.1%	1,634,120	99.8%	1,894,916	98.9%	3,352,706	94.7%
**Linked with father in registers (N (%))**	1,920,008	97.0%	1,601,138	97,8%	1,838,444	96.0%	3,248,108	91.7%
**Maternal age at birth of child (median, p25-p75)**	29 (26-33)		29 (26-33)		29 (25-33)		29 (26-33)	
**Missing information on maternal age (N (%))**	18,075	0.9%	10,099	0.6%	20,141	1.1%	187,854	5.3%
**Maternal origin (N (%))**								
Born in country	1,582,885	80.0%	1,520,159	92.9%	1,432,179	74.8%	2,399,234	67.8%
Born abroad	378,710	19.1%	111,611	6.8%	462,319	24.1%	953,467	26.9%
Unknown	18,075	0.9%	5363	0.3%	20,559	1.1%	187,859	5.3%

[1]Finnish data only include children born in country due to incomplete information on migrations. [2]Years of follow-up is calculated as first date of death, emigration, turning 18 years of age or last date with available data from the population registry minus the last date of birth, or immigration divided by 365.25. [3]E.g. disappear from register without specification. [4]Last date with data available from population registry

Exposure assessment: Vaccinations across the Nordic countries

Figure 4 depicts the coverage of diphtheria, tetanus, and acellular pertussis containing vaccines (DTaP), measles-mumps-rubella vaccine (MMR) and rota virus vaccines (rota) for children born in each country followed from birth until two years of age, date of

For peer review only

For peer review only - http://bmjopen.bmj.com/site/about/guidelines.xhtml

emigration, or date of death, whichever came first (see Appendix 3, sTable 1 for the coverage at 2 years of age for each of the included birth cohorts in each country).

In Norway, the vaccine uptake rate was highest and closest to the age of recommended vaccination compared with the other countries. In Finland and Sweden, MMR uptake starts at ages earlier than scheduled according to the respective NIPs, which reflects that MMR is recommended to children from 6 and 9 months of age in Finland and Sweden respectively, before travelling abroad. Although MMR is recommended before travelling abroad in all the Nordic countries, early uptake of MMR is much less frequent in Denmark and Norway which may indicate different interpretation and roll-out of the recommendations. The greater variation in the age at MMR vaccination in Finland reflects different vaccination schedules applied to the included birth cohorts: MMR vaccination was recommended at 14-18 months of age before June 2010 and at 12-18 months (preferably 12 months of age) after June 2010. In Finland, Norway, and Sweden, the date of the next vaccination is usually scheduled during earlier well-baby check-ups or provided by post, whereas in Denmark no formal procedures are in place to ensure timely vaccination, which may explain the different variation in age at vaccination across the countries.

Human papilloma virus vaccination (HPV) for girls was introduced in the NIP in 2009 in Denmark, the end of 2013 in Finland, mid-2009 in Norway, and in 2012 in Sweden. (Appendix 1). The vaccine is recommended at age 12 years in Denmark, Finland, and Norway, and at age 11-12 years in Sweden. Figure 5 depicts the registered coverage of HPV vaccinations among girls followed from one year before the recommended age of vaccination until age 14 years, emigration, or death, whichever came first. In Norway, the uptake of the first dose of HPV vaccine follows a steep curve at 12 years of age, representing the age of recommended vaccination (Figure 5). The majority of the included birth cohorts in Norway were only able to receive the HPV vaccination free of charge during the school year it was offered, which may have contributed to the high and steep uptake rate. In Sweden, the uptake starts increasing at 11 years of age with a second increase at 12 years of age reflecting that the vaccine may be administered in either the 5th or 6th grade. In Denmark uptake starts increasing at 12 years of age corresponding to recommended age of vaccination, but with more variation in the age of vaccination compared with the other countries. The relative low uptake combined with high age variation may be due to vaccination hesitancy following negative media attention from

For peer review only

Danish television portraying alleged serious adverse effects of HPV vaccination (45). Confidence in the safety of the vaccine has since been restored, which is reflected in the slightly increasing vaccination coverage in the last included birth cohort (Appendix 3, sTable2). In Finland, the uptake rates follow a straight curve from 12-13 years of age followed by a small proportion of children with delayed vaccination. The vaccine uptake at 14 years of age within our cohort was highest in Norway (first dose for the birth cohort 2003: 84.8%) followed by Sweden (77.9%), Finland (69.8%) and Denmark (52.3%) (Appendix 3, sTable 2).

Health and sociodemographic characteristics

Data were available for a different set of years across the Nordic countries. For comparing the study populations in this cohort profile, we only present information from years where data are available in all countries.

Prescriptions

Information on redeemed prescriptions was included for the purpose of assessing predefined health outcomes in terms of antibiotic consumption and different atopic outcomes, and to be able to assess potential confounding factors relating to underlying health and healthcare seeking behaviour. The data legislation regulating access to information on drug utilisation differed across countries. Therefore, data were only obtained for a more narrowly defined subset of ATC-codes in Finland and Sweden, compared with Denmark and Norway (Appendix 3). Information from the prescription registries was available from 2005 to 2017 in all countries. We only included information on redeemed prescriptions with ATC-codes available in all countries for the present comparison. The overall proportion of children with redeemed prescriptions ranged from 75.6% in Norway to 86.1% in Finland and varied depending on ATC-group (Table 3). The proportion of children with redeemed prescriptions in ATC-group D “dermatologicals” was 36.3% in Denmark compared with 20.6-24.7% in the other countries. Finland had the highest proportion of children with redeemed prescriptions in ATC group J “antiinfectives for systemic use”: 82.3% compared with 62.1-75.0% in the other countries. In ATC group S “eye and ear medications”, the proportion was lower in Finland (7.4%) compared with the other countries (13.0-17.9%). For ATC group R “Respiratory system” and subgroup V01 “Allergens” the proportions were relatively similar across countries.

For peer review only - http://bmjopen.bmj.com/site/about/guidelines.xhtml

Table 3: Health characteristics of children present in the respective countries from year 2005-2017.

	Denmark		Finland		Norway		Sweden	
**Prescriptions**								
Years of follow-up	2005-2017		2005-2017		2005-2017		2005-2017	
Number of children with follow-up[1] (N (%))	1,904,633	100.0%	1,634,031	100.0%	1,817,231	100.0%	3,355,915	100.0%
Children with redeemed prescriptions[2] (N (%))	1,592,361	83.6%	1,407,548	86.1%	1,374,180	75.6%	2,542,676	75.8%
Mean age during follow-up (mean, (sd))	8.3 (5.2)		8.3 (5.2)		8.3 (5.2)		8.2 (5.3)	
Prescriptions pr child (median, p25-p75)	4 (1-9)		5 (2-11)		3 (1-8)		3 (1-7)	
Children with prescriptions with ATC group D (N (%))	691,357	36.3%	360,910	22.1%	449,226	24.7%	692,269	20.6%
Prescriptions per child with ATC group D[2, 3, 4] (median, p25-p75)	1 (1-3)		1 (1-3)		1 (1-3)		1 (1-3)	
Children with prescriptions with ATC group J (N (%))	1,428,652	75.0%	1,345,297	82.3%	1,129,065	62.1%	2,194,753	65.4%
Prescriptions per child with ATC group J[2, 3, 5] (median, p25-p75)	3 (2-6)		4 (2-8)		2 (1-4)		3 (1-5)	
Children with prescriptions with ATC group R (N (%))	806,105	42.3%	748,839	45.8%	841,066	46.3%	1,468,158	43.7%
Prescriptions per child with ATC group R[2, 3, 6] (median, p25-p75)	2 (1-6)		2 (1-7)		3 (1-9)		2 (1-6)	
Children with prescriptions with ATC group S (N (%))	248,522	13.0%	121,721	7.4%	326,077	17.9%	521,658	15.5%

For peer review only - http://bmjopen.bmj.com/site/about/guidelines.xhtml

Prescriptions per child with ATC group S[2, 3, 7] (median, p25-p75)	1 (1-2)		1 (1-2)		2 (1-3)		1 (1-2)	
Children with prescriptions with ATC group V01(N (%))	10,384	0.5%	4662	0.3%	11,770	0.6%	5928	0.2%
Prescriptions per child with ATC group V01[2, 3, 8] (median, p25-p75)	5 (3-9)		4 (2-7)		4 (2-8)		4 (2-8)	
**Hospital contacts**								
Years of follow-up	2008-2016		2008-2016		2008-2016		2008-2016	
Number of children with follow-up[9] (N (%))	1,813,600	100.0%	1,581,854	100.0%	1,738,115	100.0%	3,177,371	100.0%
Children with hospital contacts (N (%))	1,069,628	59.0%	861,685	54.5%	982,808	56.5%	1,911,254	60.2%
Years of follow-up (mean, (sd))	5.8 (3.1)		5.9 (3.0)		5.7 (3.0)		5.5 (3.1)	
Mean age during follow-up (mean, (sd))	9.2 (5.8)		9.1 (5.9)		9.1 (5.8)		9.0 (5.9)	
Hospital contacts per child (main diagnosis)(median, p25-p75)	1 (0-2)		1 (0-4)		1 (0-3)		1 (0-4)	
Children with inpatient contacts (N (%))	519,945	28.7%	324,292	20.5%	420,492	24.2%	568,958	17.9%
Inpatient contacts per child (median, p25-p75)	1 (1-2)		1 (1-2)		1 (1-2)		1 (1-2)	
Children with outpatient or emergency room contacts (N (%))	885,243	48.8%	839,569	53.1%	911,877	52.5%	1,826,446	57.5%
Outpatient or emergency room contacts per child (1 per day) (median, p25-p75)	2 (1-3)		3 (1-6)		2 (1-5)		3 (1-6)	

[1] Number of children living in the country at any time in the period 2005-2017. [2] Only including ATC subgroups: D02AF, D05, D07, D11, D01, D06, D08, J01-J07, R01, R03, R06, S01G, S03, V01 - Thus, not reflecting total use of prescription medicines.[3] Per child with redeemed prescriptions of that ATC-group. [4]ATC Group D: Dermatologicals. [5]ATC Group J: Antiinfectives for systemic use. [6]ATC Group R: Respiratory system. [7]ATC Group S: Sensory organs. [8]ATC Subgroup V01: Allergens. [9] Number of children living in the country at any time in the period 2008-2016. Proportions are calculated using number of children with follow-up as the denominator.

For peer review only

Hospital contacts

Information on hospital contacts including inpatient and specialised outpatient care was available in all countries from 2008 to 2016. For comparison across countries, we excluded country-specific codes (e.g. codes for health characteristics of new-borns in Denmark). The proportion of children with hospital contacts was similar across countries (54.5-60.2%, Table 3). The proportion of children with inpatient contacts ranged from 17.9% in Sweden to 28.7% in Denmark. The proportion of children with outpatient contacts in the patient registers was highest in Sweden (57.5%) and lowest in Denmark (48.8%). The higher proportion of inpatient contacts in Denmark is likely explained by contributions of inpatient contacts without overnight stays, as contacts without overnight stays will predominantly be registered as outpatient contacts in the other countries (46). The higher proportion of children with outpatient contacts in Sweden may on the other hand be explained by a broader set of health care facilities (e.g. paediatric outpatient clinics) that report to the patient register in Sweden compared with the other countries (46).

Birth characteristics

Information on birth characteristics was available for birth cohorts from 1990 to 2016 in all countries (Table 4). The completeness of data was high in all countries, ranging from 97.7% to 99.9%. The birth characteristics were also very similar: the median birth weight ranged from 3500 to 3550 grams, the proportion of low-birthweight (below 2500 g) children ranged from 3.9% to 5.0%, and the median gestational age was 40 weeks in all countries. For the variables preterm birth, delivered by caesarean section, and singleton births, the proportions only differed by 0.8-2.7 percent points across countries. The greatest difference between countries was seen for registration of maternal smoking during pregnancy, which ranged from 8.3% in Norway to 18.2% in Denmark. The proportion with unknown/missing information on maternal smoking ranged from 2.5% in Finland to 45.8% in Norway, which may be explained by the midwifes having to inform the mothers of the need for obtaining information on smoking before asking this question in Norway, thus additional effort is required, which may hamper completeness. However, the greater proportion with missing information on maternal smoking in Norway could partly explain the lower proportion with registered maternal smoking during pregnancy, if missing information is more prevalent among smoking mothers.

For peer review only - http://bmjopen.bmj.com/site/about/guidelines.xhtml

Table 4: Birth characteristics[1]

	Denmark		Finland		Norway		Sweden	
Children born in the respective country from 1990-2016 (N)	1,728,126		1,586,526		1,591,273		2,877,753	
Children with information available from the birth registry	1,726,318	99.9%	1,576,797	99.4%	1,586,895	99.7%	2,811,119	97.7%
Birth weight in grams (Median, p25-p75)	3500 (3150-3850)		3550 (3210-3880)		3550 (3200-3900)		3540 (3200-3890)	
Low birth weight (<2500g) (N (%))	86,914	5.0%	61,546	3.9%	73,437	4.6%	114,990	4.0%
Birth weight missing (N (%))	23,707	1.4%	12,859	0.8%	5376	0.3%	72,892	2.5%
Gestational age in weeks (Median, p25-p75)	40 (39-41)		40 (39-40)		40 (39-41)		40 (39-40)	
Preterm birth (N (%))	107,656	6.2%	85,069	5.4%	98,923	6.2%	163,168	5.7%
Gestational age missing (N (%))	29,250	1.7%	16,083	1.0%	59,264	3.7%	68,973	2.4%
Delivered by caesarean section (N (%))	305,738	17.7%	258,261	16.3%	238,013	15.0%	435,680	15.1%
Mode of delivery missing (N (%))	1808	0.1%	9729	0.6%	4378	0.3%	66,634	2.3%
Singleton (N (%))	1,660,213	96.1%	1,531,748	96.5%	1,535,556	96.5%	2,731,980	94.9%
**Child order including the child itself (N (%))**								
1 (firstborn)	743,923	43.0%	647,134	40.8%	658,877	41.4%	1,211,084	42.1%
2	635,849	36.8%	532,868	33.6%	568,765	35.7%	1,023,228	35.6%
3	243,162	14.1%	244,137	15.4%	257,294	16.2%	398,331	13.8%
4 or more	86,090	5.0%	149,327	9.4%	101,959	6.4%	178,184	6.2%
Missing	19,102	1.1%	13,060	0.8%	4378	0.3%	66,926	2.3%

For peer review only - http://bmjopen.bmj.com/site/about/guidelines.xhtml

Maternal smoking during pregnancy (N (%))	314,174	18.2%	238,337	15.0%	132,734	8.3%	310,691	10.8%
Maternal smoking unknown (N (%))	134,332	7.8%	39,277	2.5%	728,038	45.8%	143,529	5.0%
[1] Information, including percentages are reported according to the number of children born in country from 1990-2016								

Socioeconomic factors

Socioeconomic information is collected yearly in all countries. In the NONSEnse cohort, the information was assessed in the year of birth of each child (Table 5) and in the 10th year of life (Appendix 3, sTable 3). Information from the year of birth was available for the birth cohorts 2004-2015 in all countries. The data presented in Table 5 only include children who were born in-country and living in the country throughout their first year of life, to ensure that they were present in the country at the time of registration.

For peer review only

Table 5: Socio-economic factors at birth

	Denmark		Finland		Norway		Sweden	
	N	(%)	N	(%)	N	(%)	N	(%)
Children present in country at birth from 2004-2015	729,294		699,052		706,443		1,314,701	
Birth cohorts included	2004-2015		2004-2015		2004-2015		2004-2015	
Income quintile at birth								
First (lowest)	134,634	18.5%	138,965	19.9%	137,551	19.5%	247,237	18.8%
Second	137,041	18.8%	138,997	19.9%	141,566	20.1%	265,557	20.2%
Third	137,390	18.8%	139,012	19.9%	141,962	20.1%	267,347	20.3%
Fourth	137,415	18.9%	138,998	19.9%	141,995	20.1%	267,349	20.3%
Fifth	136,935	18.8%	138,900	19.9%	141,533	20.1%	266,528	20.3%
Unknown	45,531	6.2%	4180	0.6%	644	0.1%	605	0.0%
Number of children in the household the year the child is born								
1	310,237	42.6%	287,312	41.1%	298,563	42.3%	574,229	43.7%
2	278,396	38.2%	237,291	33.9%	263,726	37.4%	487,446	37.1%
3	106,184	14.6%	104,278	14.9%	108,822	15.4%	176,338	13.4%
>3	32,106	4.4%	65,527	9.4%	33,496	4.7%	68,060	5.2%
Unknown	2023	0.3%	4644	0.7%	644	0.1%	605	0.0%
Single parenthood in the years the child is born								
Yes	58,646	8.0%	55,089	7.9%	68,018	9.6%	132,243	10.1%
No	668,277	91.7%	639,319	91.5%	635,689	90.1%	1,181,775	89.9%
Unknown	2023	0.3%	4644	0.7%	1544	0.2%	605	0.0%
Highest attained educational level[1] of the mother on the date the child is born								
Low education	114,880	15.8%	98,608	14.1%	126,777	18.0%	149,673	11.4%
Medium education	261,761	35.9%	279,687	40.0%	201,316	28.5%	431,880	32.9%
High education	336,536	46.2%	319,530	45.7%	350,684	49.7%	457,040	34.8%
Unknown	15,769	2.2%	1227	0.2%	26,474	3.8%	276,030	21.0%

[1] Highest attained education was categorized based on the International Standard Classification of Education (ISCED) 2011 using the main groups (47).

In Denmark, 6.2% of the study population had missing information on household income compared with 0-0.6% in the other countries. We have been unable to identify the reason for the higher proportion in Denmark. The proportion of households with 3 or more children was 9.4% in Finland compared with 4.4-5.2% in the remaining Nordic countries. The proportion living with a single parent in the year of birth ranged from 7.9% in Finland to 10.1% in Sweden. Among the remaining socioeconomic variables, the largest cross-

For peer review only - http://bmjopen.bmj.com/site/about/guidelines.xhtml

country difference was found for the highest attained education of the mother, where information was missing for 21.0% of the children in Sweden compared with 0.2-3.8% in the other Nordic countries. The proportion of mothers with low education ranged from 11.4% in Sweden to 18.0% in Norway. The high proportion with missing information on maternal education in Sweden is in part caused by a higher proportion of children with an unknown mother in our dataset (Table 2) but may also be caused by education not being registered for mothers born abroad. Since registration of education is often a necessity for employment in more advanced fields, it is reasonable to assume a higher accuracy for registration of high education as compared with low education.

Findings to date

The data collection process was completed in March 2022. The findings to date pertain to investigations of similarities and differences in rates of infectious disease hospitalisations (46), and antibiotic consumption (48). These studies highlight trends in infectious disease morbidity across the Nordic countries and further guide the use of more consistent infectious disease outcome measures for future studies.

The results regarding the non-specific effects of vaccines are at the moment limited to an interrupted timeseries analysis, which could be undertaken using aggregated data that was ready before all the individual based data was obtained in all countries (49). Future studies will include population-level investigations of natural experiments in the form of introduction of new vaccines or changes in the immunisation programmes, as well as individual-level studies comparing vaccinated and unvaccinated children with a given vaccine using multiple different study designs.

FURTHER DETAILS

Strengths and limitations

The NONSEnse project represents a unique undertaking for conducting register based epidemiological studies of the overall health effects of routine childhood vaccines.

Data are stored separately in each country, which prevents conducting analyses on the joint data, which is a limitation of the project. However, the Common Data Model enables analysis plans and statistical code to be written in one country and sent to the other

For peer review only - http://bmjopen.bmj.com/site/about/guidelines.xhtml

countries that can then perform the same analyses and share the results (Figure 3). The use of a Common Data Model thus minimises the risk that different country-specific analytical decisions will hinder comparability of results.

The use of register data presents both strengths and weaknesses. A strength pertains to the multitude of information available for the entire study population and linked to the individual, which minimises selection bias and enables cohort studies with prospective follow-up and control for multiple confounding factors. The generalisability of the Finnish cohort is limited to children born in-country. However, for most of studies to be undertaken within this project, this will have limited implications since we will often restrict the study population to children born in-country for the studies of childhood vaccinations to ensure complete information on vaccinations given from birth. Limitations include that not all the wished-for information are available in all countries and registration may be incomplete, which limits the possibility to e.g., adjust for hypothesised confounding factors such as day-care attendance and lifestyle factors. Also, previous studies (2) have found the non-specific effect of a vaccine to be strongest when it is the most recent vaccine administered. Therefore, it is relevant to include information on vaccines other than the ones offered through the NIP. In Denmark, Finland, and Norway vaccines outside the NIP may also be registered in the vaccination registers, but registration of these vaccines has only been mandatory in more recent years (35, 36, 39). In Sweden only vaccinations within the NIP are included in the vaccination register. The analyses are thus limited by different possibilities to assess the effect of a given vaccine as long as it is the most recent vaccine, both within and across countries.

In all the Nordic countries information on emigration relies on the individual reporting resettlement to the authorities. This is mandatory when leaving the country for more than 6 months in Denmark (50) and Norway (51), and for more than 12 months in Sweden (52) and Finland (53). Thus, incomplete information on emigrations, due to leaving the country for shorter periods of time or if parents fail to register the resettlement, may result in children being lost to follow-up without us knowing it from the registers. This may in turn result in our studies underestimating events, e.g. infectious disease hospitalisations, as these are only registered for children who are in the country.

Overall, it is clear that expert knowledge is needed before combining and using Nordic register data for research purposes (29). As such, an important strength of NONSEnse

For peer review only - http://bmjopen.bmj.com/site/about/guidelines.xhtml

pertains to the data harmonisation process through bi-weekly analysis workshops involving designated research groups from each of the four countries with expert knowledge on country-specific register data, the heath care systems, and immunisation programmes.

Validity of exposure and outcome measures

In all countries, the vaccines offered through the NIP are subject to mandatory registration. However, validity depends on the reporting accuracy by the health care providers that administer the vaccinations. A Danish study validated the coverage of MMR from the registers using medical records from the general practitioner in a subset of the population and found that the coverage in the register was 86% compared with 94% through inspection of the medical records (54). A similar comparison conducted in Sweden also found underreporting of MMR in the register of around 5-7 percentage units (unpublished). It is unlikely that underreporting of vaccines is associated with the outcomes investigated within the NONSEnse project, therefore, the misclassification will most likely be non-differential and would thus bias the results towards no association.

The prescription registers only contain information on drugs dispensed from filled prescriptions, whereas some drugs are also available over the counter, which are not included in the registers. This includes e.g. weak corticosteroids for topical use (ATC: D07AA) or drugs used to treat symptoms in the eye due to e.g. allergy (ATC: S01G). It is thus possible that the observed cross-country differences in the proportion of children with these prescriptions are affected by national policies or guidelines, or the behaviour of the prescriber or purchaser. Atopic outcomes will, in part, be identified using filled prescriptions for products that are also available over the counter, which may hamper cross country comparability. Antibiotics, however, are prescription drugs in all four countries and thus not affected by over-the-counter purchases.

Several differences in health care organisation, administration, and registration may hamper cross country comparability of the health outcomes included in this project. A strength of NONSEnse is the thorough investigation of the intended outcomes in independent studies which has informed and maximised comparability of the outcome measures to be used in the subsequent studies of non-specific effects of vaccines.

For peer review only - http://bmjopen.bmj.com/site/about/guidelines.xhtml

Methodological considerations

Evaluating the effect of implemented vaccination programmes is challenging; the high vaccine uptake rate makes comparisons between vaccinated and unvaccinated children difficult due to the individual factors that determine vaccine uptake. Healthy vaccinee bias may arise if the healthiest children are more likely to follow the vaccination recommendations than the less healthy children (55). However, due to different vaccination schedules in different countries, the children who have received MMR at e.g.15 months of age may be classified as vaccinated according to schedule, too early or too late, depending on the country. Furthermore, age is a strong predictor of both vaccination and the risk of infectious diseases (46). A strength therefore pertains to the observed delay in age at vaccination within each country, which facilitates comparison of different vaccination statuses among children of the same age. For vaccines with a steep and high uptake at the recommended age of vaccination, the children who do not receive the vaccines as scheduled are more likely a selected subgroup of the population, thus hampering comparability with the rest of the population. In contrast, larger variation in the age at vaccination increases comparability between children with different vaccination status according to age.

A strength of this study setup is the many differences in the immunisation programmes, and in changes to the immunisation programmes, the country-specific bias structures, and the possibility to integrate results from different study designs, which facilitate triangulation that can strengthen the potential for making causal deductions (13, 14). The project has already led to useful new information regarding differences and similarities in childhood morbidity between the Nordic countries. Most importantly, the project will increase our understanding of vaccines and how they may affect health in more general ways - holding potential for direct translation into more efficient immunisation programmes and improved child health.

Data sharing statement

Due to data protection rules and ethical permissions, we are not allowed to share the individual-level data. However, the insights presented in this cohort profile, including the common data model, can serve to guide the construction of similar Nordic databases by other researchers fulfilling the requirements to obtain Nordic registry data. The possibility

For peer review only - http://bmjopen.bmj.com/site/about/guidelines.xhtml

to generate Nordic population-based cohorts could for example be used to study health interventions and outcomes related to the SARS-CoV-2 pandemic across the Nordic countries

Funding

This work was supported by NordForsk [grant number: 83839], Odense University Hospital Research fund [A-number: 2519], and the faculty scholarship from the University of Southern Denmark [grant number N/A], .

Author contributions

LG, IL, HE, HN, BF, ML, AP, LT, CSB, and SS conceptualized the Manuscript. LG, IL, HE, HN, and SS designed the methodology used to present the included data. LG, IL, HE, and ML managed data curation, undertook the country specific coding, and produced the aggregated data presented. LG, IL, HE, HN, BF, ML, AP, LT, CSB, and SS investigated and validated the aggregated data produced. LG and IL did the formal analysis of data. LG, IL, HE, BF, LT, HN, AP, and SS obtained the data to be included in this cohort. LG drafted the first version of the manuscript. IL, HE, HN, BF, ML, AP, LT, CSB, and SS critically revised the draft. LG and IL produced the visualizations. CSB and SS supervised the project. LG, IL, HN, BF, ML, AP, LT, CSB, and SS obtained the funding for the present project.

Ethics

Ethical approval is not required for registry-based studies in Denmark and Finland. However, the study was approved by the Danish Data Protection Agency and by the Institutional Review Board of the Finnish Institute for Health and Welfare. In Norway, study approval was obtained from the Regional Ethics Committee, South-East. In Sweden, study approval was obtained from the Regional Ethical Review Board, Stockholm, Sweden.

Competing interest

AAP, HN and ML are investigators in vaccine-related studies for which THL has received funding from GSK, Pfizer and Sanofi Pasteur. The remaining authors report no relation that could be construed as a conflict of interest.

For peer review only - http://bmjopen.bmj.com/site/about/guidelines.xhtml

FIGURE LEGENDS

Figure 1: Flowchart of study population in Denmark, Finland, Norway, and Sweden

Figure 2: Nordic register information linked to the individual using a unique personal identification code (ID)

Figure 3: Transforming country specific datasets into NONSense datasets using a common data model

Figure 4: Vaccination coverage[1] according to age (inverse Kaplan-Meier estimates) among children[2] born in-country in Denmark, Finland, Norway, and Sweden

Abbreviations: DTP1: First dose of Diphtheria, Tetanus, and acellular Pertussis containing vaccine; DTP2: Second dose of Diphtheria, Tetanus, and acellular Pertussis containing vaccine; DTP3: Third dose of Diphtheria, Tetanus, and acellular Pertussis containing vaccine; MMR: Measles-Mumps-Rubella vaccine; Rota: Rota virus vaccine

[1] The coverage reflects the number of registered vaccines and may thus underestimate the actual vaccination coverage in the countries. [2] Including children born in the country from birth cohorts where vaccines administered between 0-2 years of age are registered in the vaccination registers (data availability period). The included birth cohorts are 1997-2016 in Denmark; 2009-2015 in Finland; 1995-2016 for DTP and MMR vaccine and 2015-2016 for Rota in Norway; 2013-2015 in Sweden. Number of children in each birth year is presented in Appendix 3, sTable 1.

Figure 5: Human papilloma virus vaccination coverage[1,2] according to age (inverse Kaplan-Meier estimates) among girls[3] in Denmark, Finland, Norway, and Sweden

Abbreviations: HPV1: First dose of Human papilloma virus vaccine; HPV2: Second dose of Human papilloma virus vaccine

[1]The coverage reflects the number of registered vaccinations and may thus underestimate the actual vaccination coverage. [2]In some countries the recommended vaccination schedule changed from 3 to 2 doses during follow-up. Only the 2 first doses are reported here. [3] Including girls from birth cohorts where HPV vaccination has been offered from 1 year before age of recommended vaccination until 14 years of age and where vaccinations were registered in the vaccination registers. The included birth cohorts are 1998-2004 in Denmark, 2002-2003 in Finland, 1998-2004 in Norway, and 2003 in Sweden. Number of girls included in each birth cohort is presented in Appendix 3, sTable2.

For peer review only - http://bmjopen.bmj.com/site/about/guidelines.xhtml

References

1. Higgins JPT, Soares-Weiser K, López-López JA, Kakourou A, Chaplin K, Christensen H, et al. Association of BCG, DTP, and measles containing vaccines with childhood mortality: systematic review. BMJ (Clinical research ed). 2016;355.
2. Benn CS, Fisker AB, Rieckmann A, Sørup S, Aaby P. Vaccinology: time to change the paradigm? The Lancet Infectious diseases. 2020;20(10):e274-e83.
3. de Bree LCJ, Koeken V, Joosten LAB, Aaby P, Benn CS, van Crevel R, et al. Non-specific effects of vaccines: Current evidence and potential implications. Seminars in immunology. 2018;39:35-43.
4. Gehrt L, Rieckmann A, Kiraly N, Jensen AKG, Aaby P, Benn CS, et al. Timeliness of DTaP-IPV-Hib Vaccination and Development of Atopic Dermatitis Between 4 Months and 1 Year of Age-Register-Based Cohort Study. The journal of allergy and clinical immunology In practice. 2020.
5. Kiraly N, Koplin JJ, Crawford NW, Bannister S, Flanagan KL, Holt PG, et al. Timing of routine infant vaccinations and risk of food allergy and eczema at one year of age. Allergy. 2016;71(4):541-9.
6. Rieckmann A, Hærskjold A, Benn CS, Aaby P, Lange T, Sørup S. Measles, mumps and rubella vs diphtheria-tetanus-acellular-pertussis-inactivated-polio-Haemophilus influenzae type b as the most recent vaccine and risk of early 'childhood asthma'. Int J Epidemiol. 2019;48(6):2026-38.
7. Steenhuis TJ, van Aalderen WM, Bloksma N, Nijkamp FP, van der Laag J, van Loveren H, et al. Bacille-Calmette-Guerin vaccination and the development of allergic disease in children: a randomized, prospective, single-blind study. Clinical and experimental allergy : journal of the British Society for Allergy and Clinical Immunology. 2008;38(1):79-85.
8. Thostesen LM, Kjaergaard J, Pihl GT, Birk NM, Nissen TN, Aaby P, et al. Neonatal BCG vaccination and atopic dermatitis before 13 months of age: A randomized clinical trial. Allergy. 2018;73(2):498-504.
9. Sørup S. Careful consideration of hypotheses and model assumptions in study of non-specific effects of vaccines. Vaccine. 2020;38(9):2115.
10. Gehrt L, Aaby P, Benn CS, Sørup S. Regarding "Early childhood vaccination and subsequent mortality or morbidity: are observational studies hampered by residual confounding? A Danish register-based cohort study" [Internet]. https://bmjopen.bmj.com/content/9/9/e029794.responses#regarding-%E2%80%9Cearly-childhood-vaccination-and-subsequent-mortality-or-morbidity-are-observational-studies-hampered-by-residual-confounding-a-danish-register-based-cohort-study%E2%80%9D2019. [cited 2021].
11. Tielemans S, de Melker HE, Hahne SJM, Boef AGC, van der Klis FRM, Sanders EAM, et al. Non-specific effects of measles, mumps, and rubella (MMR) vaccination in high income setting: population based cohort study in the Netherlands. BMJ (Clinical research ed). 2017;358:j3862.
12. Sinzinger AX, Von Kries R, Siedler A, Wichmann O, Harder T. Non-specific effects of MMR vaccines on infectious disease related hospitalizations during the second year of life in high-income countries: a systematic review and meta-analysis. Human vaccines & immunotherapeutics. 2019:1-9.
13. Lawlor DA, Tilling K, Davey Smith G. Triangulation in aetiological epidemiology. Int J Epidemiol. 2016;45(6):1866-86.
14. Benn CS, Fisker AB, Rieckmann A, Jensen AKG, Aaby P. How to evaluate potential non-specific effects of vaccines: the quest for randomized trials or time for triangulation? Expert review of vaccines. 2018;17(5):411-20.
15. Laugesen K, Ludvigsson JF, Schmidt M, Gissler M, Valdimarsdottir UA, Lunde A, et al. Nordic Health Registry-Based Research: A Review of Health Care Systems and Key Registries. Clin Epidemiol. 2021;13:533-54.
16. Danish Health Authority. The Danish Childhood Vaccination Programme. https://www.sst.dk/-/media/Udgivelser/2019/Boernevaccinationsprogrammet-

For peer review only - http://bmjopen.bmj.com/site/about/guidelines.xhtml

pjece/Boernevaccinationsprogrammet-pjece-engelsk.ashx?la=en&hash=A30CD2C279D0C4C08FA22D872FD836292ED9189F; 2018.
17. Finnish Institute for health and welfare. Vaccination Programme for children and adults 2020 [Available from: https://thl.fi/en/web/infectious-diseases-and-vaccinations/information-about-vaccinations/vaccination-programme-for-children-and-adults#children.
18. The Public Health Agency of Sweden. Vaccination Programmes 2021 [Available from: https://www.folkhalsomyndigheten.se/the-public-health-agency-of-sweden/communicable-disease-control/vaccinations/vaccination-programmes/.
19. Norwegian Institute of Public Health. When will your child be offered vaccines? : Norwegian Institute of Public Health; 2020 [Available from: https://www.fhi.no/en/id/vaccines/childhood-immunisation-programme/when-will-your-child-be-offered-vaccines/.
20. Sundhedsstyrelsen. Vejledning om HIV (Human Immundefekt Virus), Hepatitis B og C virus: Forebyggelse af Blodbåren i smitte, Diagnostik og HåNdtering i Sundhedsvæsnet og På Andre Arbejdspladser.
21. Finnish Institute for health and welfare. Hepatitis B vaccine 2022 [updated 2022 Feb 1. Available from: https://thl.fi/en/web/infectious-diseases-and-vaccinations/vaccines-a-to-z/hepatitis-vaccines/hepatitis-b-vaccine.
22. Folkhälsomyndigheten. Rekommendationer om pneumokockvaccination till riskgrupper 2019 [updated 2020 August 10. Available from: https://www.folkhalsomyndigheten.se/publicerat-material/publikationsarkiv/r/rekommendationer-om-pneumokockvaccination-/.
23. Ministry of Health. Bekendtgørelse om gratis influenzavaccination til visse persongrupper. In: Ministry of Health, editor. https://www.retsinformation.dk/eli/lta/2021/20742021.
24. Finnish Institute for health and welfare. Influenza vaccine 2020 [updated 2020 Aug 14. Available from: https://thl.fi/en/web/infectious-diseases-and-vaccinations/vaccines-a-to-z/influenza-vaccine.
25. Norwegian Institute of Public Health. Influenza vaccine for risk groups 2021 [updated 2021.11.11. Available from: https://www.fhi.no/en/id/influensa/influenza-vaccine/influenza-vaccine-for-risk-groups2/.
26. Finnish Institute for health and welfare. Pneumococcal vaccines 2020 [updated 2020 Jun 8. Available from: https://thl.fi/en/web/infectious-diseases-and-vaccinations/vaccines-a-to-z/pneumococcal-vaccines.
27. Norwegian Institute of Public Health. Pneumokokkvaksine - veileder for helsepersonell 2008 [updated 2021.04.21. Available from: https://www.fhi.no/nettpub/vaksinasjonsveilederen-for-helsepersonell/vaksiner-mot-de-enkelte-sykdommene/pneumokokkvaksinasjon---veileder-fo/.
28. Finnish Institute for health and welfare. TBE, or ‘tick vaccine’ 2021 [Available from: https://thl.fi/en/web/infectious-diseases-and-vaccinations/vaccines-a-to-z/tbe-or-tick-vaccine-.
29. Maret-Ouda J, Tao W, Wahlin K, Lagergren J. Nordic registry-based cohort studies: Possibilities and pitfalls when combining Nordic registry data. Scandinavian journal of public health. 2017;45(17_suppl):14-9.
30. Ludvigsson JF, Almqvist C, Bonamy AK, Ljung R, Michaëlsson K, Neovius M, et al. Registers of the Swedish total population and their use in medical research. European journal of epidemiology. 2016;31(2):125-36.
31. Schmidt M, Pedersen L, Sorensen HT. The Danish Civil Registration System as a tool in epidemiology. European journal of epidemiology. 2014;29(8):541-9.
32. Helsedata. Befolkning 2022 [Available from: https://helsedata.no/no/forvaltere/statistisk-sentralbyra-ssb/befolkning/.
33. Digital and population data services agency. Population Information System [Available from: https://dvv.fi/en/personal-data.
34. Krause TG, Jakobsen S, Haarh M, Mølbak KJE. The Danish vaccination register. 2012;17(17):20155.

For peer review only - http://bmjopen.bmj.com/site/about/guidelines.xhtml

35. Baum U, Sundman J, Jaaskelainen S, Nohynek H, Puumalainen T, Jokinen J. Establishing and maintaining the National Vaccination Register in Finland. Euro Surveill. 2017;22(17).
36. Trogstad L, Ung G, Hagerup-Jenssen M, Cappelen I, Haugen I, Feiring BJE. The Norwegian immunisation register–SYSVAK. 2012;17(16):20147.
37. Chrapkowska C, Galanis I, Kark M, Lepp T, Lindstrand A, Roth A, et al. Validation of the new Swedish vaccination register - Accuracy and completeness of register data. Vaccine. 2020;38(25):4104-10.
38. Andersen JS, Olivarius NDF, Krasnik A. The Danish National Health Service Register. Scandinavian journal of public health. 2011;39(7 Suppl):34-7.
39. Hækkerup N. Bekendtgørelse om adgang til og registrering m.v. af lægemiddel- og vaccinationsoplysninger. In: Forebyggelse MfSo, editor. https://www.retsinformation.dk/eli/lta/2014/4602014.
40. Munk-Jørgensen P, Bertelsen A, Dahl AA, Lehtinen K, Lindström E, Tomasson KJNJoP. Implementation of ICD-10 in the Nordic countries. 1999;53(1):5-9.
41. Wettermark B, Zoega H, Furu K, Korhonen M, Hallas J, Norgaard M, et al. The Nordic prescription databases as a resource for pharmacoepidemiological research--a literature review. Pharmacoepidemiol Drug Saf. 2013;22(7):691-9.
42. Klaukka T. The Finnish database on drug utilisation. Norsk Epidemiologi. 2001;11(1).
43. Kanta. e-prescriptions in Kanta-archive: Kanta; [Available from: https://www.kanta.fi/en/system-developers/what-are-kanta-services.
44. Aarnio E, Huupponen R, Martikainen JE, Korhonen MJ. First insight to the Finnish nationwide electronic prescription database as a data source for pharmacoepidemiology research. Research in social & administrative pharmacy : RSAP. 2020;16(4):553-9.
45. Suppli CH, Hansen ND, Rasmussen M, Valentiner-Branth P, Krause TG, Mølbak K. Decline in HPV-vaccination uptake in Denmark – the association between HPV-related media coverage and HPV-vaccination. BMC Public Health. 2018;18(1):1360.
46. Gehrt L, Laake I, Englund H, Nieminen H, Benn CS, Feiring B, et al. Hospital Contacts for Infectious Diseases Among Children in Denmark, Finland, Norway, and Sweden, 2008-2017. Clin Epidemiol. 2022;14:609-21.
47. UNESCO Institute for Statistics. International standard classification of education: ISCED 2011. 2012.
48. Skajaa N, Gehrt L, Nieminen H, Laake I, Englund H, Sönksen UW, et al. Trends in Antibiotic Use in Danish, Finnish, Norwegian and Swedish Children. Clin Epidemiol. 2022;14:937-47.
49. Sørup S, Englund H, Laake I, Nieminen H, Gehrt L, Feiring B, et al. Revaccination with measles-mumps-rubella vaccine and hospitalization for infection in Denmark and Sweden - An interrupted time-series analysis. Vaccine. 2021.
50. Careja R, Bevelander P. Using population registers for migration and integration research: examples from Denmark and Sweden. Comparative Migration Studies. 2018;6(1):19.
51. Skatteetaten. Melding om flytting fra Norge [Available from: https://www.skatteetaten.no/person/folkeregister/flytte/fra-norge/.
52. Ludvigsson JF, Almqvist C, Bonamy A-KE, Ljung R, Michaëlsson K, Neovius M, et al. Registers of the Swedish total population and their use in medical research. European journal of epidemiology. 2016;31(2):125-36.
53. Digital and Population Data Services Agency. Moving to Finland or moving abroad from Finland 2022 [Available from: https://dvv.fi/en/moving-abroad-or-from-abroad-to-finland.
54. Holt N, Mygind A, Bro F. Danish MMR vaccination coverage is considerably higher than reported. Danish medical journal. 2017;64(2).
55. Remschmidt C, Wichmann O, Harder T. Frequency and impact of confounding by indication and healthy vaccinee bias in observational studies assessing influenza vaccine effectiveness: a systematic review. BMC Infect Dis. 2015;15:429.

For peer review only - http://bmjopen.bmj.com/site/about/guidelines.xhtml

For peer review only

For peer review only - http://bmjopen.bmj.com/site/about/guidelines.xhtml

Flow chart: Selection of children for the final study population

Children born from 1990 and registered as living in country before the age of 18 years[1]

Denmark (1990-2018)
N=1,992,211

Exclusion N=12,541
- Children born in Greenland[2] (N=7547)
- Currently living in Greenland[2] and/or inactive/annulled ID number (N=2096)
- Lacking date of entry to the country (N=149) (all foreign born)
- Registered as emigrated with emigration date equal or prior to date of first residency (N=147)
- No follow-up time in cohort[3] (N=2898)

Number of children included: N=1,979,670
- 1,813,443 (91.6%) children born in-country
- 154,250 (7.8%) foreign born children
- 11,977 (0.6%) uncertain[4] foreign born or born in-country

Finland (1990-2017)
N = 1,924,938

Exclusion N=287,805
- Born abroad[5] (N= 287,161)
- Lost to follow-up with unknown migration date (N=465)
- Personal ID missing (N=25)
- Same child has multiple personal ID (N=22)
- No follow-up time in cohort[3] (N=132)

Number of children included: N= 1,637,133
- 1,637,133 children born in-country
- 0 foreign born children
- 0 uncertain[4] foreign born or born in-country

Norway (1990-2018)
N = 1,923,317

Exclusions N=8260
- Registered as born in Norway, but date of first residency missing or prior to birth (N = 256)
- Born abroad, but with unknown date of first residency (N = 390)
- Registered as emigrated with emigration date unknown or prior to date of first residency (N = 674)
- Registered with confidential address in the Population Registry (N = 331)
- No follow-up time in cohort[6] (N=6609)

Number of children included: N = 1,915,057
- 1,703,054 (88.9%) children born in Norway
- 205,859 (10.7%) foreign born children
- 6,144 (0.3%) uncertain[4] foreign born or born in-country

Sweden (1990-2017)
N= 3,541,045

Exclusion N=480
- Registered as foreign born, but without immigration date N=469
- Registered with a migration date that precedes the date of birth (N=7)
- No follow-up time in cohort[3] (N=9)

Number of children included: N=3,540,560
- 2,993,472 (84.5%) children born in-country
- 530,092 (15.0%) foreign born children
- 16,996 (0.5%) uncertain[4] foreign born or born in-country

Number of children included: N=9,072,420
- 8,147,149 (89.8%) children born in-country
- 890,154 (9.8%) foreign born children
- 35,117 (0.4%) uncertain[4] foreign born or born in-country

[1] In Finland data from the population register including information on deaths and migrations were obtained on February 8th, 2014. Thus, only children who were still alive and living in the country from this date were included.

[2] Children born or residing in Greenland are registered as living in Denmark. However, the Greenlandic hospitals and pharmacies do not report to the patient register or prescription register

[3] Children who die or migrate on the same date as they enter the cohort

[4] Children registered as born in the country but with an immigration date registered without a preceding emigration date: in these cases, it is not clear if the child is born in-country or has immigrated to the country.

[5] Most immigration dates were not known, thus all children born abroad were excluded

[6] Date of birth assigned as a random integer within the month of birth, thus date of death or migration within the month of birth is regarded no follow-up time in the cohort.

For peer review only - http://bmjopen.bmj.com/site/about/guidelines.xhtml

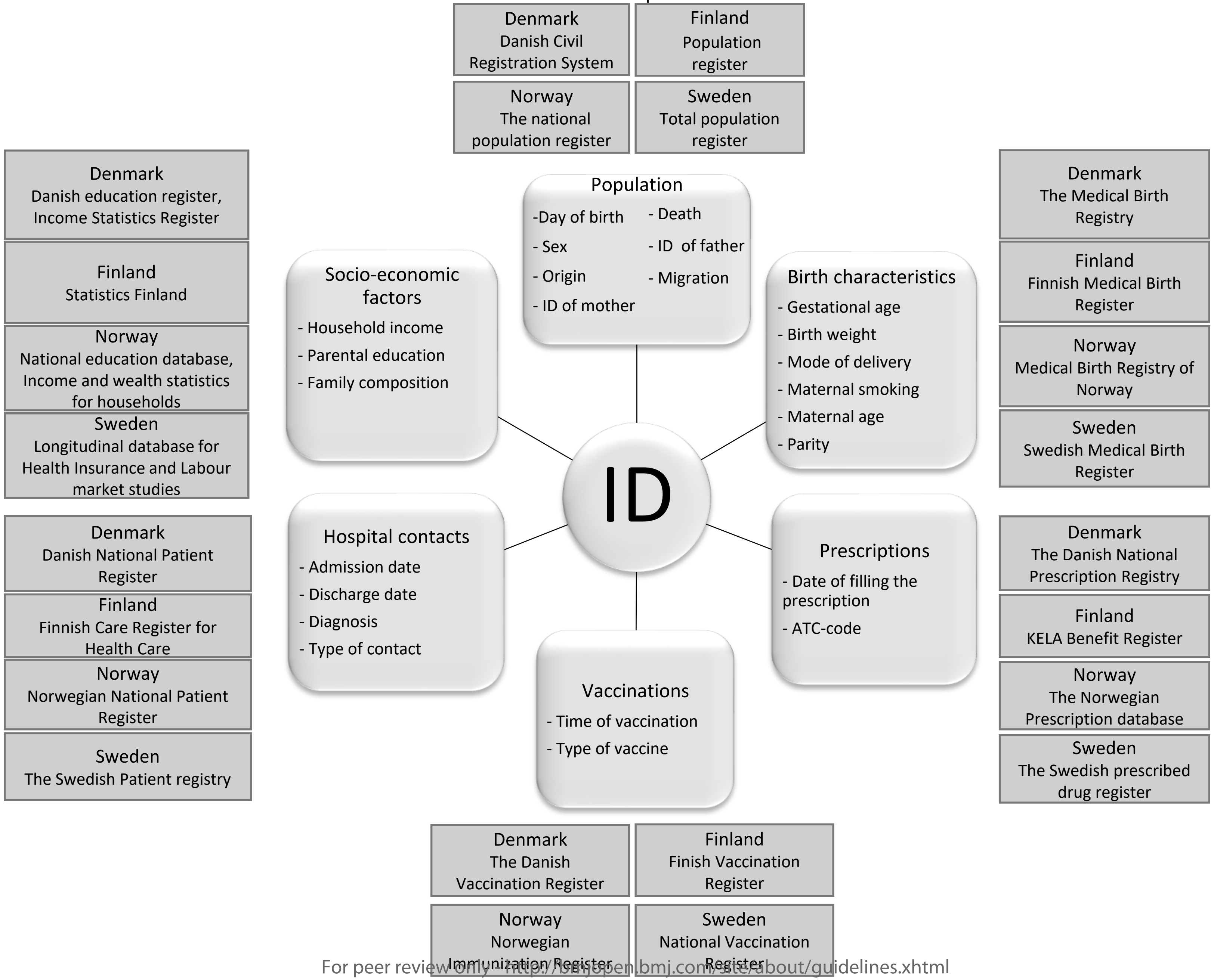
Denmark
Danish Civil Registration System
Finland
Population register
Norway
The national population register
Sweden
Total population register
Population
-Day of birth
- Sex
- Origin
- ID of mother
- Death
- ID of father
- Migration
Denmark
Danish education register, Income Statistics Register
Finland
Statistics Finland
Norway
National education database, Income and wealth statistics for households
Sweden
Longitudinal database for Health Insurance and Labour market studies
Socio-economic factors
- Household income
- Parental education
- Family composition
Birth characteristics
- Gestational age
- Birth weight
- Mode of delivery
- Maternal smoking
- Maternal age
- Parity
Denmark
The Medical Birth Registry
Finland
Finnish Medical Birth Register
Norway
Medical Birth Registry of Norway
Sweden
Swedish Medical Birth Register
ID
Denmark
Danish National Patient Register
Finland
Finnish Care Register for Health Care
Norway
Norwegian National Patient Register
Sweden
The Swedish Patient registry
Hospital contacts
- Admission date
- Discharge date
- Diagnosis
- Type of contact
Prescriptions
- Date of filling the prescription
- ATC-code
Denmark
The Danish National Prescription Registry
Finland
KELA Benefit Register
Norway
The Norwegian Prescription database
Sweden
The Swedish prescribed drug register
Vaccinations
- Time of vaccination
- Type of vaccine
Denmark
The Danish Vaccination Register
Finland
Finish Vaccination Register
Norway
Norwegian Immunization Register
Sweden
National Vaccination Register

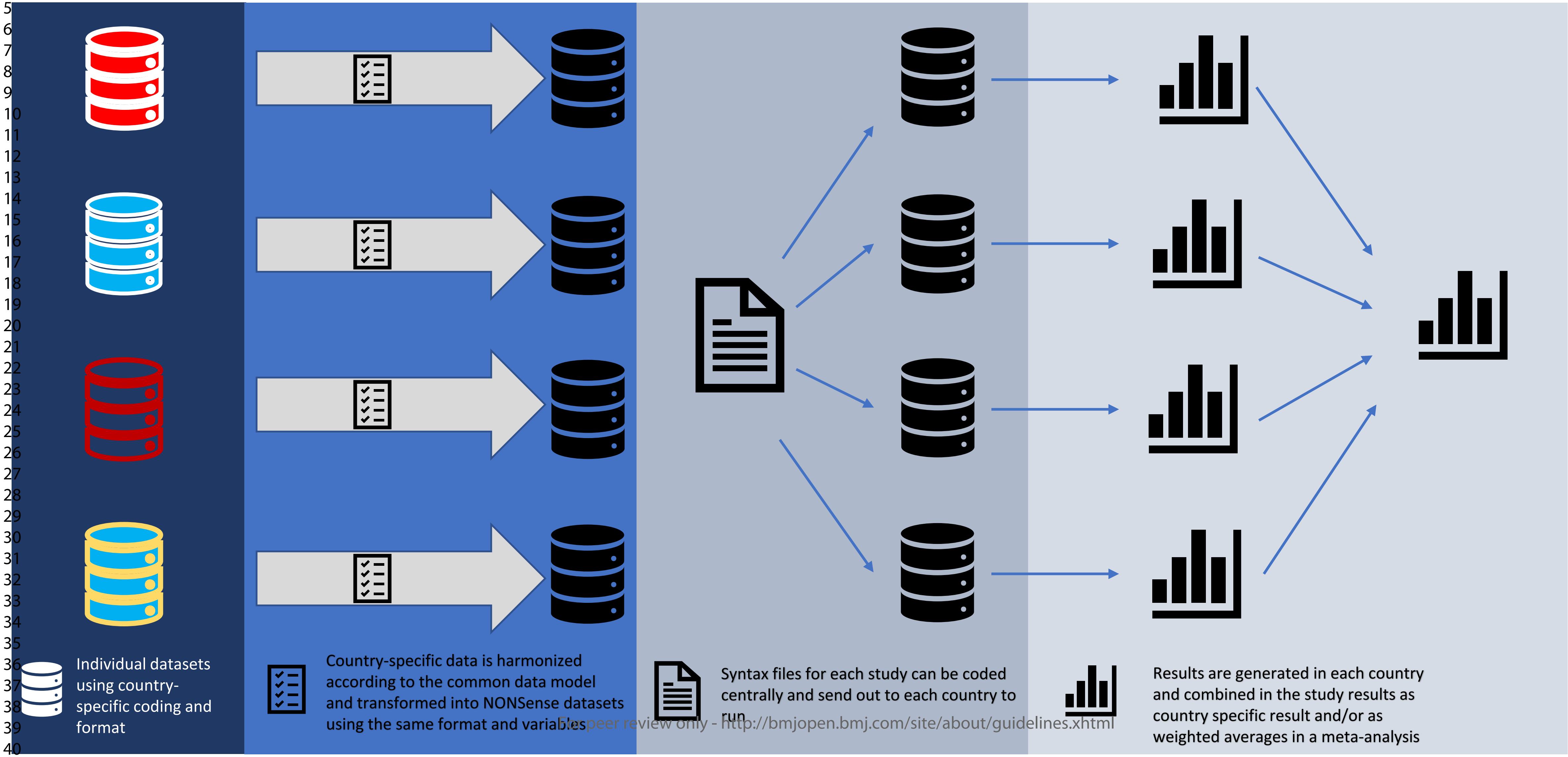

For peer review only - http://bmjopen.bmj.com/site/about/guidelines.xhtml

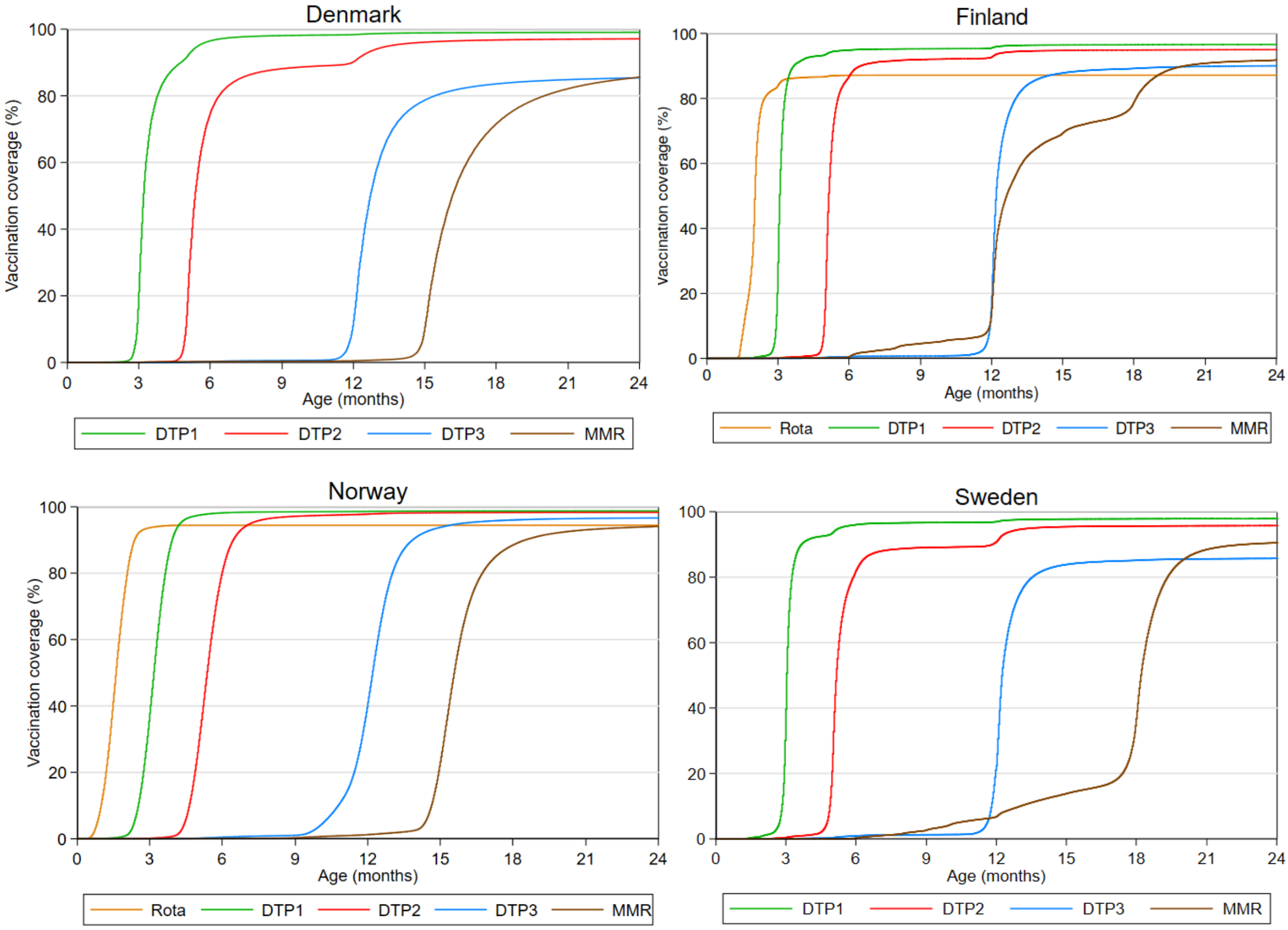
Denmark
Finland
Norway
Sweden
Vaccination coverage (%)
Age (months)
0 20 40 60 80 100
0 3 6 9 12 15 18 21 24
Rota
DTP1
DTP2
DTP3
MMR

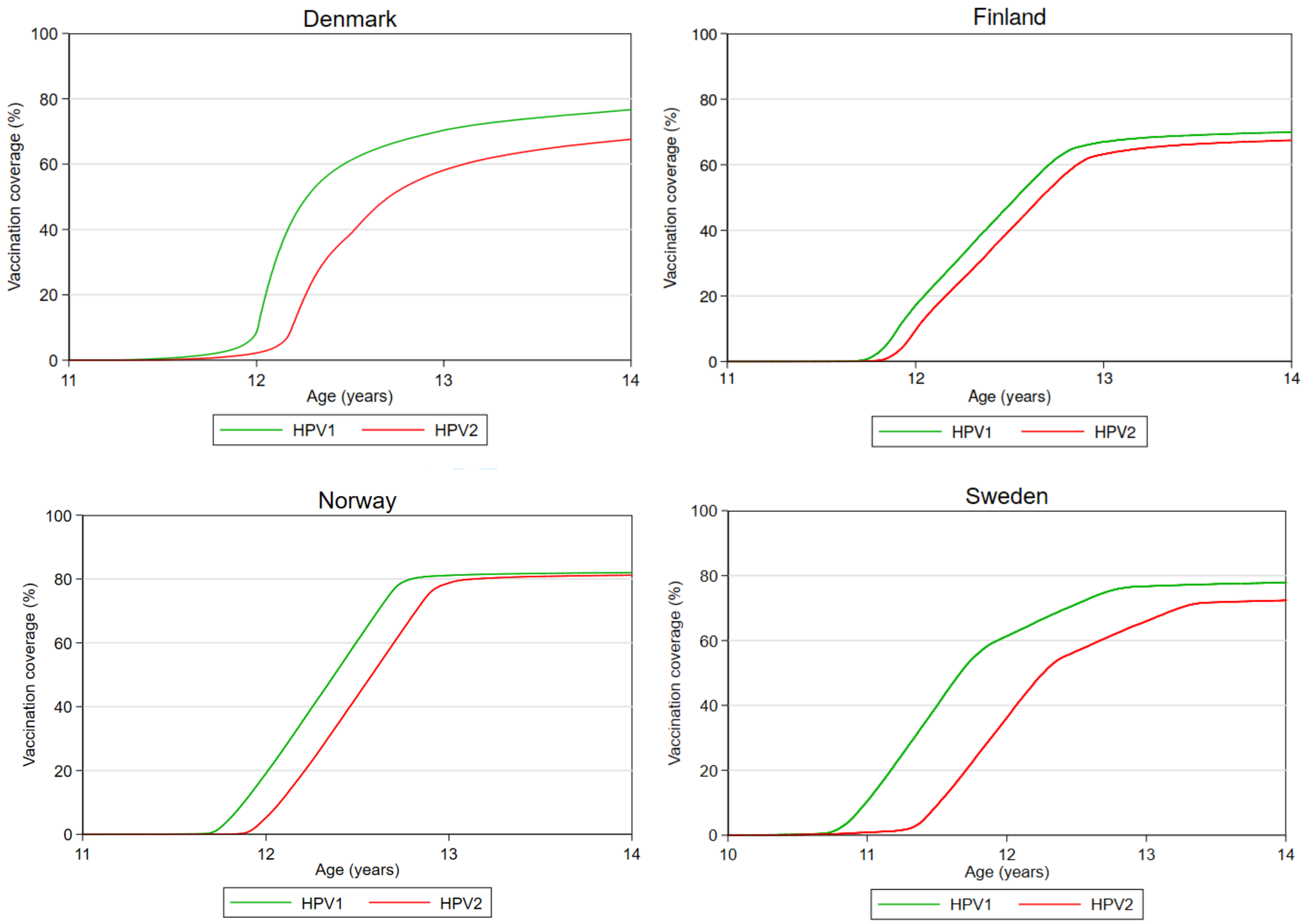
Denmark
Finland
Norway
Sweden
Vaccination coverage (%)
Age (years)
0
20
40
60
80
100
10
11
12
13
14
HPV1
HPV2

Lexis diagram for Danish cohort including vaccination programme and data availability

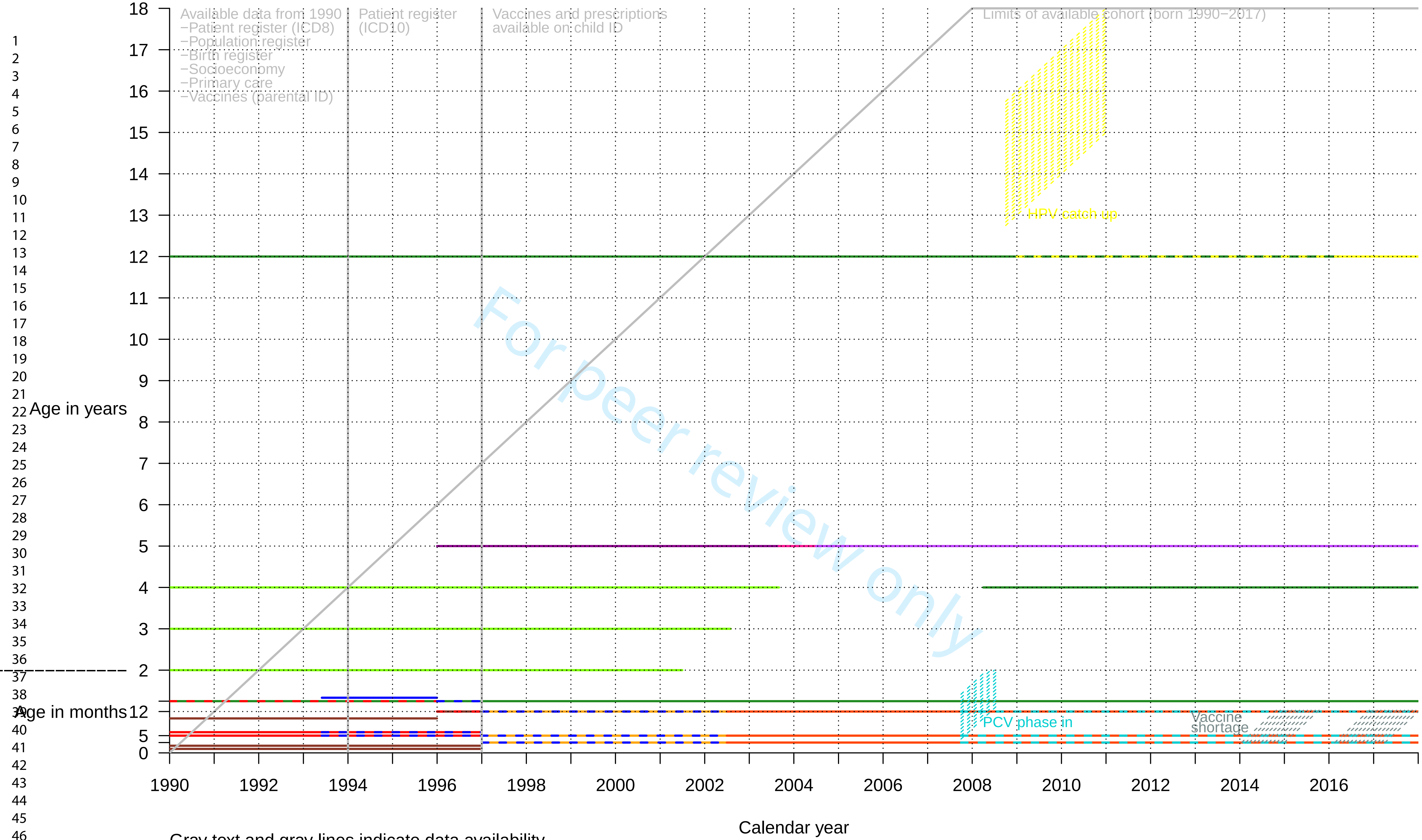

Gray text and gray lines indicate data availability
Color codes for vaccines:
wP; DT−IPV; DTaP−IPV; DTaP−IPV−Hib; Hib; PCV; MMR; OPV; DT; DTaP; DTaP−IPV; HPV

Abbreviations for vaccines:
Non−live vaccines: wP=whole cell pertussis; D=diphtheria; T=tetanus; IPV=inactivated polio vaccine; aP=pertussis vaccine(acellular);
Hib=Haemophilus influenzae type b; PCV=pneumococcal conjugate vaccine; HPV=Human papilloma virus
Live vaccines: MMR=measles−mumps−rubella; OPV=oral polio vaccine

For peer review only

Lexis diagram for Finnish cohort including vaccination programme and data availability

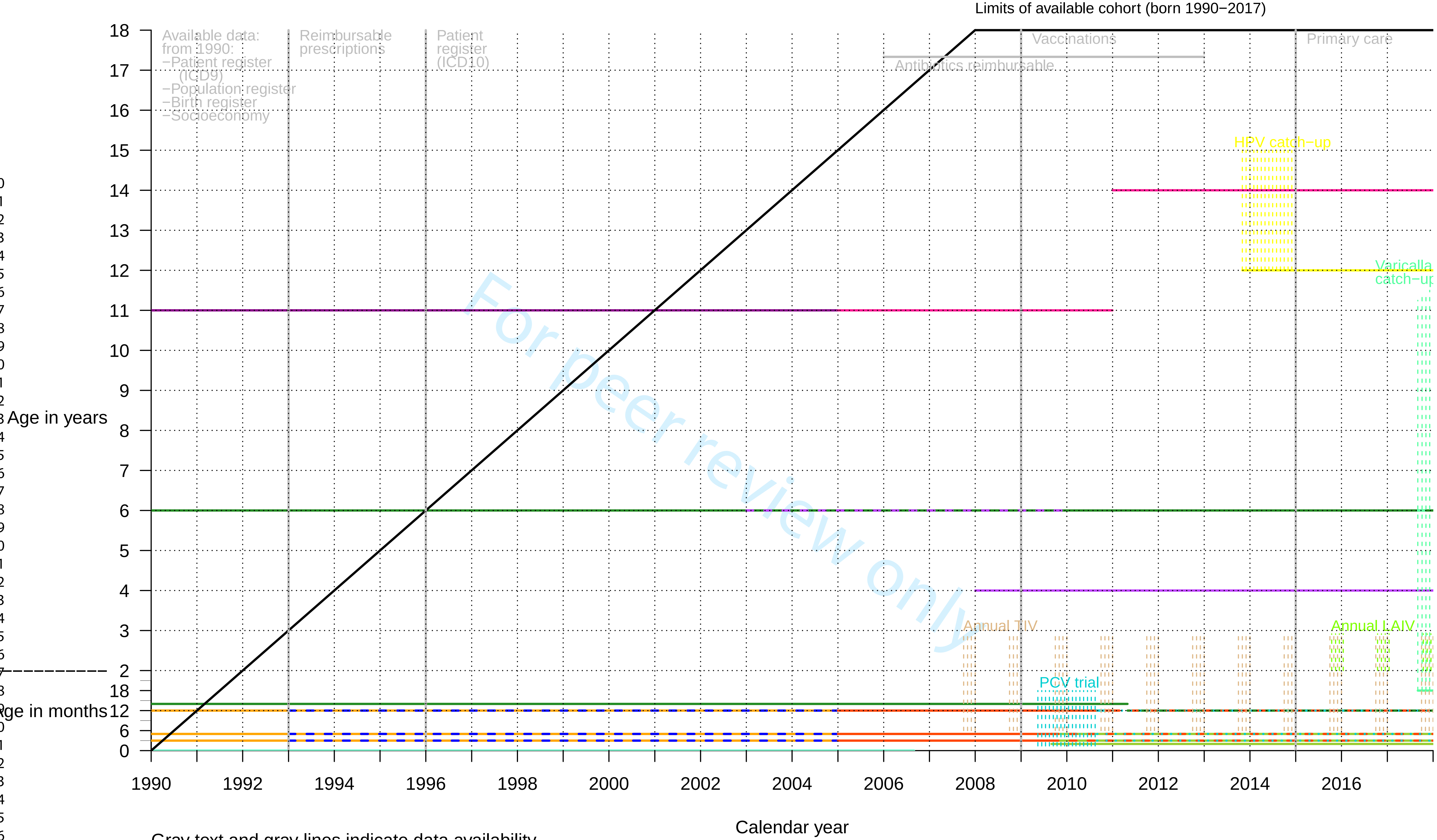

For peer review only

Gray text and gray lines indicate data availability
Color codes for vaccines:
BCG; RV; DTaP−IPV; Hib; DTaP−IPV−Hib; PCV; MMR; V; DTaP−IPV−booster; DT−booster; DTaP−booster; HPV

Abbreviations for vaccines:
Non−live vaccines: D=diphtheria; T=tetanus; aP=pertussis vaccine(acellular); IPV=inactivated polio vaccine; Hib=Haemophilus influenzae type b;
PCV=pneumococcal conjugate vaccine; TIV=trivalent influenza vaccine; HPV=Human papilloma virus
Live vaccines: BCG=Bacille Calmette−Guerin; RV=Rotavirus; MMR=measles−mumps−rubella; V=varicella; LAIV=live attenuated influenza vaccine

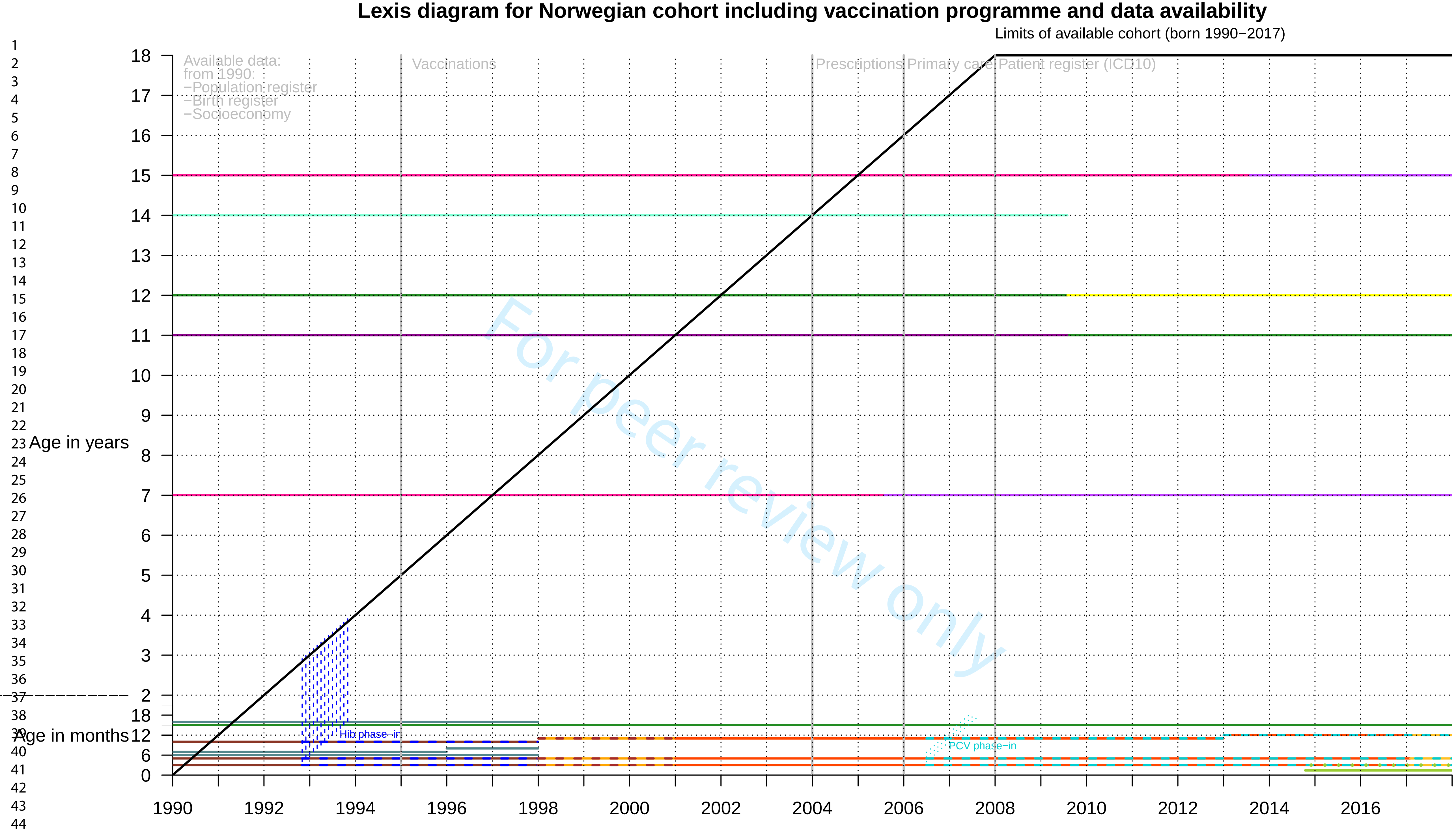

Gray text and gray lines indicate data availability

Color codes for vaccines:

RV; DTwP; Hib; DTaP; IPV−Hib; DTaP−IPV−Hib; PCV; DTaP−IPV−Hib−HepB; IPV; MMR; IPV−booster; DTaP−IPV−booster; DT−booster; HPV; BCG

Abbreviations for vaccines:

Non−live vaccines: D=diphtheria; T=tetanus; wP=whole−cell pertussis vaccine; Hib=Haemophilus influenzae type b;aP=pertussis vaccine(acellular);
IPV=inactivated polio vaccine; PCV=pneumococcal conjugate vaccine; HepB=Hepatitis B; HPV=Human papilloma virus
Live vaccines: RV=Rotavirus; MMR=measles−mumps−rubella; BCG=Bacille Calmette−Guerin

For peer review only - http://bmjopen.bmj.com/site/about/guidelines.xhtml

Lexis diagram for Swedish cohort including vaccination programme and data availability

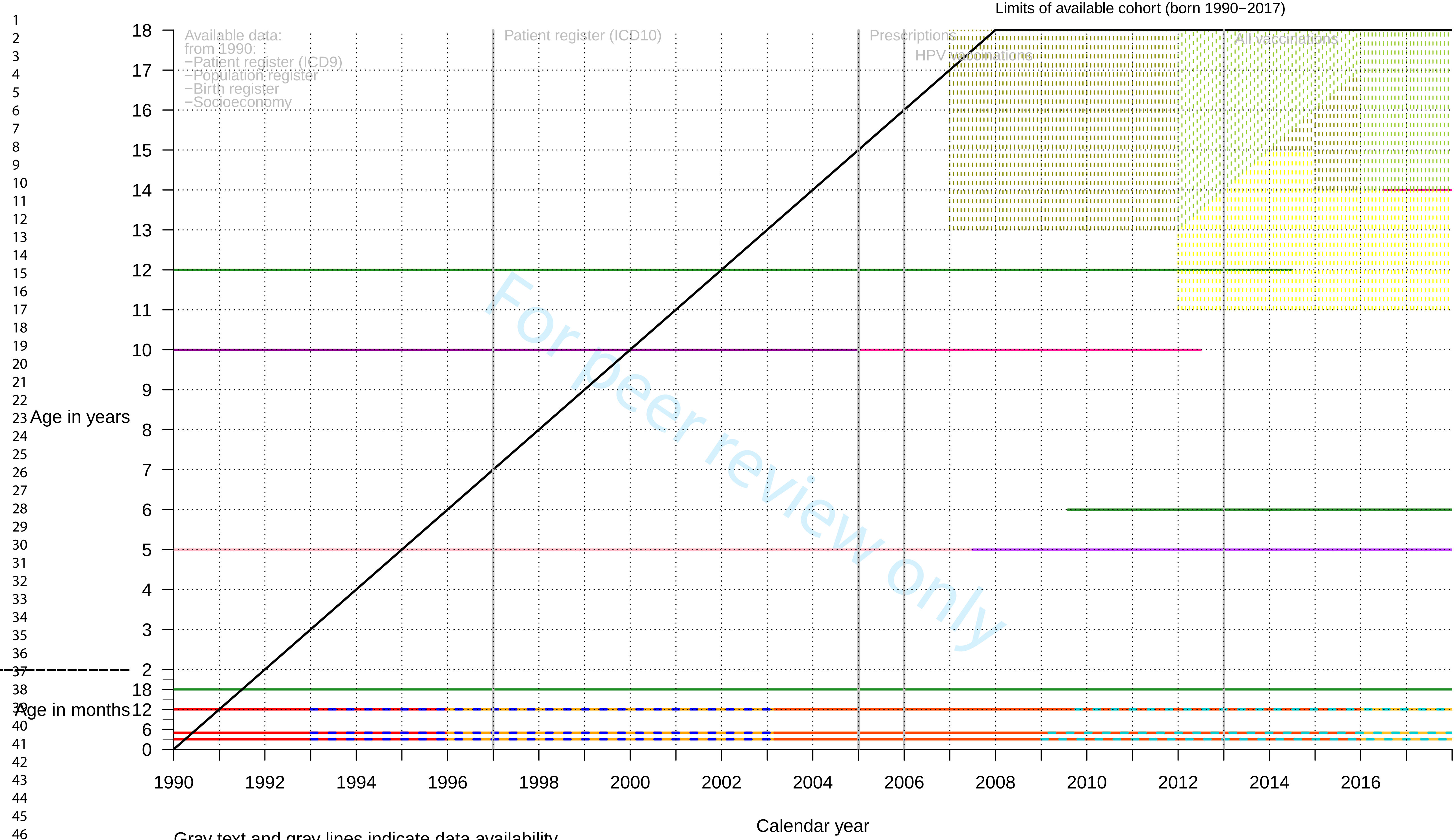

For peer review only

Gray text and gray lines indicate data availability

Color codes for vaccines:

DT−IPV; Hib; DTaP−IPV; DTaP−IPV−Hib; PCV; DTaP−IPV−Hib−HepB; MMR; IPV−booster ; DTaP−IPV−booster ; DT−booster; DTaP−booster; HPV−recommended age ; HPV−own payment with partly subsidy ; HPV catch−up

Abbreviations for vaccines:

Non−live vaccines: D=diphtheria; T=tetanus; aP=pertussis vaccine(acellular); IPV=inactivated polio vaccine; Hib=Haemophilus influenzae type b; PCV=pneumococcal conjugate vaccine; HepB=Hepatitis B; HPV=Human papilloma virus

Live vaccines: MMR=measles−mumps−rubella

Link to Contents

NONSense Common Data Model

Nov 28th 2022

Link to Contents

For peer review only - http://bmjopen.bmj.com/site/about/guidelines.xhtml

Link to Contents

Contents

Contents 2
Introduction 3
Background/Event tables 4
Table: prescriptions 4
Table: hospital_contacts 4
Table: population1 5
Table: birth_characteristics 6
Table: Vaccines 7
Table: socio_economy 8
Source of data in each country 11
Table: prescriptions 11
Table: hospital_contacts 14
Table: population1 19
Table: birth_charcteristics 28
Table: Vaccines 36
Table: socio_economy 40
Appendix: Vaccine categorization: 50

Link to Contents

For peer review only - http://bmjopen.bmj.com/site/about/guidelines.xhtml

Link to Contents

Introduction

The Common Data Model (CDM) is a tool for documentation of data preparation and generation of uniform datasets across the Nordic countries (Denmark, Finland, Norway, and Sweden). The aim is to construct a number of uniform background datasets and event tables, which share the same name and entail the same variables, labels and values across countries. Datasets with the exact same format across Countries enables sharing of syntax-files for study analyses.
The CDM is a working document, which will be updated according to country specific data preparation, and expanded as all necessary information will be transformed into background/event tables. In the end, the background/event tables will include all necessary information to conduct all future studies (morbidity/incidence studies and vaccination studies).

The current version presents data content and preparation as per April 2022

The CDM contains 1) “Background/Event tables”, and 2) “Source of data and data preparation in each country”.

Background/Event tables: include information on the name of the dataset to be used by NONSEnse and format and labeling of each variable within the dataset.

Source of data in each country: includes a description of the information on the source register, and source variables, which have been used to generate the variables in the background/event tables. These tables furthermore entail information on important notes (i.e data breaks, limitations such as i.e. restricted information on redeemed prescriptions in Finland) and data preparation (how have the source variables been modified to generate the variables in the background/event tables). The tables on source of data in each country have been filled in by the individual countries following country specific data preparation.

Link to Contents

For peer review only - http://bmjopen.bmj.com/site/about/guidelines.xhtml

Link to Contents

Background/Event tables

Table: prescriptions

Description: Table of all redeemed prescriptions (included atc codes in each country is listed in “source of data and datapreparation”) among individuals in the study population.
Structure: 1 observation (line) for every redeemed pharmaceutical.

Variables:

**Variable**	**Label**	**values**
id	Personal id of the child	string
b_date	Birthdate of the child	Date Format (%dD_m_Y)
sex	Sex of the child as recorded in the dataset	1=”male”<br>2=”female”
redeemdate	Date of redeeming the prescription	Date Format (%dD_m_Y)
atc	Full atc code for the redeemed drug	String (7 digits) use capital letters i.e “J01AA01”

Table: hospital_contacts

Description: Table of all diagnoses (both main diagnosis and all other diagnoses) for somatic patients including information on sex and date of birth for all children in the study population. Note that a patient can have multiple diagnoses attached to the same contact.
Structure: 1 observation (line) for each diagnosis received

Variables:

Variable	label	values
id	Personal id of the child	String
b_date	Birthdate of the child	Date Format (%dD_m_Y)
sex	Sex of the child as recorded in the dataset	1=”male”<br>2=”female”
adm_date	Date of admission	Date Format (%dD_m_Y)
discharge_date	Date of discharge	Date Format (%dD_m_Y)
diag	ICD diagnosis code	String (For ICD-10 codes use max 4 digits e.g. A063)
diagtype	Type of diagnosis	1=”Main diagnosis”<br>2=”Other diagnosis”
type_contact	Type of hospital contact	Categorical:<br>1=”inpatient”<br>2=”emergency room patient”<br>3=”outpatient”<br>4=”outpatient or emergency room patient”

Link to Contents

For peer review only - http://bmjopen.bmj.com/site/about/guidelines.xhtml

Link to Contents

Table: population1

Description: Background table including information follow-up for each child in the study population. The dataset only includes information on the child's first stay in the country (first in_date and first cens_date is recorded).

Structure: one line for each child

Variable	label	values
id	Personal id of the child	string
b_date	Birthdate of the child	Date Format (%dD_m_Y)
sex	Sex of the child as recorded in the dataset	1="male"<br>2="female"
origin	Born in the country or abroad	1="born in-country"<br>2="born abroad"<br>9= "Unknown"
in_date	Date of entering the cohort	Date Format (%dD_m_Y)
in_reason	Reason for entering the cohort	1="birth"<br>2="immigration"
cens_date	First date of censoring	Date Format (%dD_m_Y)
cens_reason	Reason for being censored	1="death"<br>2="out migration"<br>3="other"
m_id	id of mother	string
f_id	id of father	string
m_age	Mothers age in years at time of delivery	Numeric (discrete)
m_origin	Maternal origin at birth	1="born in-country"<br>2="born abroad"<br>9= "Unknown"
p_origin	Paternal origin at birth	1="born in-country"<br>2="born abroad"<br>9= "Unknown"

Link to Contents

For peer review only - http://bmjopen.bmj.com/site/about/guidelines.xhtml

Link to Contents

Table: birth_characteristics

Structure: one line for each child in the study population

variable	Label	values	Legal values
id	Personal id of the child	string	
b_weight	Birthweight of child (gram)	Numeric	100-9990
ga	Gestational age (full weeks)	Numeric (discrete)	
sectio	Delivered by caesarean section	0=”not delivered by caesarean section”<br>1=”delivered by caesarean section”<br>9=” unknown”	
smoke	Maternal smoking or snuff at any point during pregnancy	0= ”no”<br>1= “smoking (or snuff) during pregnancy”<br>9= “unknown”	
singleton	singleton	0=”no”<br>1=”yes”<br>9=” unknown”	
child_order	Child order (including the child itself)	Numeric (discrete)	

For peer review only

Link to Contents

For peer review only - http://bmjopen.bmj.com/site/about/guidelines.xhtml

Link to Contents

Table: Vaccines

variable	Label	values
id	Personal id of the child	string
vacdate	Date of vaccination	Date Format (%dD_m_Y)
vaccine	Type of vaccine administered	Categorical (see coding in appendix ”vaccine categorization”)
credibility	Credibility indication of vaccine information	1=no duplicate<br>2= duplicate same vaccine removed<br>3=duplicate related vaccine removed (keep vaccine that aligns with vaccination schedule)<br>4= duplicate related vaccine removed (none of the vaccines align with vaccination schedule)<br>5= duplicate related vaccine removed (vaccines given outside the vaccination schedule)
TB_endemic	Vaccine recommendations in accordance with connections to TB endemic countries	0=not risk group<br>1=risk group<br>9=not relevant
HepB_endemic	Vaccination recommendations in accordance with connections to HepB endemic countries	0=not risk group<br>1=risk group<br>9=not relevant

Prioritization for duplicate selection:

1. Remove same vaccines (variable name: “vaccine”, see appendix “vaccine categorization”) given 14 days or less after the previous vaccine for the same child (if DTP is registered on day 0, 10 and 20, only remove the vaccine registered at day 10) – *keep the earliest registration*
    i. *Credibility=2*
2. Remove vaccines from the same type of vaccines (variable name: “type” see appendix “vaccine categorization”) given 14 days or less after the previous vaccine of the same type. Register vaccine as given on the earliest date within the duplicate combination
   prioritize within combinations:
    a. Keep vaccine that aligns with vaccination schedule according to ***age*** and ***year of vaccination***
        i. Credibility =3
    b. If no vaccine aligns with vaccination schedule but type and age correspond to timing of childhood vaccinations keep the vaccine that protects against most conditions
        i. Credibility=4
    c. If vaccines are given outside ages for recommended vaccination according to the vaccination program – keep the vaccine that protects against most conditions

        i. Credibility=5

Link to Contents

For peer review only - http://bmjopen.bmj.com/site/about/guidelines.xhtml

Link to Contents

Table: socio_economy

Assign information to all children in the study population. If a child has no registrations in the socio economic datasets variables should be coded as 9 or 99=”unknown” as described in the table below.

Overall note on timing of information:

Variables ending with “_b” indicate that information is from birth of the child. Depending on the set up of the register information we will use the date or year of birth to obtain the information. If information is not available for the date or year of birth, we will use information from the year after.

Variables ending with “_10y” indicate that information is from the year/date the child turns 10 years. Depending on the set up of the register information we will use the date or year of turning 10 years to obtain the information.

variable	Label	values	Legal values	Notes
id	Personal id of the child	string		
inc_quin_b	Household income quintile at year of birth of the child	1=first (lowest)<br>2=second<br>3= third<br>4= fourth<br>5= fifth (highest)<br>9=”unknown”		Quintiles are calculated stratified on year (i.e., calculation of quintiles are done separately for each calendar year.<br>If several income variables are available, selection is based on this priority: 1: equated disposable household/family income; 2: disposable household/family income; 3:household/family income; 4: maternal disposable income; 5: maternal income.
inc_quin_10y	Household income quintile at the year of the child’s 10th birthday	1=first (lowest)<br>2=second<br>3= third<br>4= fourth<br>5= fifth (highest)<br>9=”unknown”		See notes under inc_quin_b
inc_quin_m_b	Maternal income quintile at year of birth of the child	1=first (lowest)<br>2=second<br>3= third<br>4= fourth		See notes under inc_quin_b

Link to Contents

For peer review only - http://bmjopen.bmj.com/site/about/guidelines.xhtml

Link to Contents

		5= fifth (highest)<br>9="unknown"		
inc_quin_m_10y	Maternal income quintile at the year of the child's 10th birthday	1=first (lowest)<br>2=second<br>3= third<br>4= fourth<br>5= fifth (highest)<br>9="unknown"		See notes under inc_quin_b
n_children_b	Number of children below 18 years in the household including the child itself at year of birth of the child	Numeric discrete<br><br>99="unknown"	>=1	
n_children_10y	Number of children below 18 years in the household including the child itself at the year of the child's 10th birthday	Numeric discrete<br><br>99="unknown"	>=1	
single_parent_b	Single parenthood at year of birth of the child	0=No<br>1=Yes<br>9="unknown"		
single_parent_10y	Single parenthood at the year of the child's 10th birthday	0=No<br>1=Yes<br>9="unknown"		
m_education_b	Maternal highest attained education at year of birth of the child	1=Low education (ISCED2011 level 0-2)<br>2=Medium education (ISCED2011 level 3-4)<br>3=High education (ISCED2011 level 5-8)<br>9="unknown"		International Standard Classification of Education (ISCED) 2011 coded into main groups. Read more in reference 1 below the table.
m_education_10y	Maternal highest attained education at	1=Low education (ISCED2011 level 0-2)		See notes under m_education_b.

Link to Contents

For peer review only - http://bmjopen.bmj.com/site/about/guidelines.xhtml

Link to Contents

	the year of the child's 10th birthday	2=Medium education (ISCED2011 level 3-4) 3=High education (ISCED2011 level 5-8) 9="unknown"		

Reference 1 for ISCED: https://ec.europa.eu/eurostat/statistics-explained/index.php/International_Standard_Classification_of_Education_(ISCED)#Implementation_of_ISCED_2011_.28levels_of_education.29

For peer review only

Link to Contents

For peer review only - http://bmjopen.bmj.com/site/about/guidelines.xhtml

Link to Contents

Source of data in each country

Table: prescriptions

	Denmark		Finland		Norway		Sweden	
Variable	Source and description	Important notes and data preparation	Source and description	Important notes and data preparation	Source and description	Important notes and data preparation	Source and description	Important notes and data preparation
id	Original name in the Danish data: “pnr”. Pseudonomised unique personal identification number for linkage between registers, created by Statistics Denmark. It is linkable (by Statistics Denmark) to the original personal identification number (CPR number) assigned to all Danish residents and used when reporting to all national registers.	Renamed from “pnr”	Obtained from KELA Register: “KELA etuusrekisteri”, Table:”Lääkeostot”, Variable: ”HETU”<br><br>Register: ”Kanta Reseptikeskus” Table: ”KANTA:RESEPTI.LAAKETOIMITUKSET” Variable: “PATIENT_ID”<br><br>THL pseudonymised the original personal identification code (in these registers HETU and PATIENT_ID) to unique personal identification number for linkage between registers. THL data management can link the id back to original personal identification code.	Statistics Finland pseudonymised HETU and PATIENT_ID	Obtained from Register: “The Norwegian Prescription Database” (NorPD) Pseudonomised unique personal identification number for linkage between registers	Renamed from “pasient_lopenr_pdb2471”	Created by Statistics Sweden Pseudonomised unique personal identification number for linkage between registers	Renamed from lopnr

Link to Contents

For peer review only - http://bmjopen.bmj.com/site/about/guidelines.xhtml

Link to Contents

b_date	Obtained from the Danish National Health Data Agency.<br>Register: "CPR-Registret"<br>Table: "t_person"<br>Variable: "d_foddato"	Renamed from "d_foddato"	Obtained from KELA<br>Obtained from KELA<br>Register: " KELA etuusrekisteri",<br>Table:"Lääkeostot",<br>Variable: "HETU"<br><br>Register: "Kanta Reseptikeskus"<br>Table: "KANTA:RESEPTI.LAAKETOIMITUKSET"<br>Variable: "PATIENT_ID"	Extracted from "HETU" before pseudonymisation was done.<br><br>Extracted from "PATIENT_ID" before pseudonymisation was done.	Obtained from The National Population Register	We have received information on month and year of birth, but not day. For each individual, we have therefore generated a random integer between 1 and length of their month of birth. Using this random integer as day of birth, everyone is assigned an exact birth date.	Obtained from Statistics Sweden<br>Register: "Register över totalbefolkningen, RTB"<br>Variable: "fodddatum"	Renamed from fodddatum
sex	Obtained from the Danish National Health Data Agency.<br>Register: "CPR-Registret"<br>Table: "t_person"<br>Variable: "C_KON"<br>Sex as recorded by personal identification number.	sex=1 "male" if C_KON is "M"<br>sex=2 "female" if C_KON is "K"	Obtained from KELA<br>Register: " KELA etuusrekisteri",<br>Table:"Lääkeostot",<br>Variable: "HETU"<br><br>Register: "Kanta Reseptikeskus"<br>Table: "KANTA:RESEPTI.LAAKETOIMITUKSET"<br>Variable: "PATIENT_ID"	Extracted from "HETU" and "PATIENT_ID" before pseudonymisation was done.<br>sex=1 "male"<br>sex=2 "female"	Obtained from The National Population Register.	Renamed from "kjonn"	Obtained from Statistics Sweden<br>Register: "RTB"<br>Variable: "kon"	Renamed from "kon"
redeemdate	Obtained from Statistics Denmark.<br>Register: "Lægemiddeldatabasen"<br>Variable: "EKSD"<br>Date of redeeming the prescription	Renamed from "EKSD"	Obtained from KELA<br>Register: " KELA etuusrekisteri",<br>Table:"Lääkeostot",<br>Variable: "OSTOPV"<br><br>Register: "Kanta Reseptikeskus"<br>Table: "KANTA:RESEPTI.LAAKETOIMITUKSET"<br>Variable: "CREATION_DATE"<br>Date of redeeming the prescription	Renamed from "OSTOPV"<br>Renamed from "CREATION_DATE"	Obtained from Register: "NorPD"<br>Variable: "UtleveringsDato"<br>Date of redeeming the prescription	Renamed from "UtleveringsDato"	Obtained from Socialstyrelsen<br>Register: "Läkemedelsregisteret"<br>Variable: "edatum"	Renamed from "edatum". (Date of redeeming the prescription)

For peer review only

Link to Contents

atc	Obtained from statistics Denmark.<br>Register: “Lægemiddeldatabasen”<br>Variable: “ATC”<br>ATC code of purchased drug	All prescriptions with ATC group D, J, R, S and V01, including all sublevels.<br>Renamed from “ATC”	Obtained from KELA<br>Register: “ KELA etuusrekisteri”<br>Table:” Lääkeostot”<br>Variable: “ATC”<br><br>Register: ”Kanta Reseptikeskus”<br>Table: ”KANTA:RESEPTI.LAAKETOIMITUKSET”<br>Variable: “ATC_CODE”<br><br>ATC code of purchased drug	All prescriptions with ATC groups D07, D11AH, J, R01, R03, R06, S01G, S03 and V01, including all sublevels.<br>V01 only from KELA data.<br>In Korvattavat lääkkeet only reimbursable products.<br>Reimbursement of antibiotics:<br>< 2006 no reimbursement if cheap<br>2006-2012: all antibiotics were reimbursed<br>>2012: individual products not reimbursed”<br><br>Duplicates removed: if same purchase (same id, redeemdate and atc) was found from both registers only one of them was included in the data.	Obtained from Register: “NorPD”<br>Variable: “ATCKode”<br>ATC code of purchased drug	All prescriptions with ATC group D, J, R, S and V01, including all sublevels<br>Renamed from “ATCKode”	Obtained from Socialstyrelsen<br>Register: “Läkemedelsregisteret”<br>Variable: “atc”	ATC code of purchased drug. The data from Sweden included all prescriptions within ATC groups D, J, R, S and V01, including all sublevels

Link to Contents

For peer review only - http://bmjopen.bmj.com/site/about/guidelines.xhtml

Link to Contents

Table: hospital_contacts

	Denmark		Finland		Norway		Sweden	
Variable	Source and Description	Important notes and data preparation	Source and Description	Important notes and data preparation	Source and Description	Important notes and data preparation	Source and Description	Important notes and data preparation
							In the original Swedish register data, each admission date is a separate line with all diagnoses and other information included in one line. The dataset has been reshaped to long format with one line for each diagnosis	
id	Original name in the Danish data: "pnr". Pseudonomised unique personal identification number for linkage between registers, created by Statistics Denmark. It is linkable (by Statistics Denmark) to the original personal identification number (CPR number) assigned to all Danish residents and used when reporting to all national registers.	Renamed from "pnr"	Obtained from THL Register: "the Finnish National Patient Register THL=Hilmo" Table: "Perustiedot/Asiakas, potilas" Variable: "HT"<br><br>THL pseudonymised the original personal identification code (in this register HT) to unique personal identification number for linkage between registers. THL data management can link the id back to original personal identification code.	Statistics Finland pseudonymised HT with their own id for the remote user system.	Obtained from the Norwegian National Patient Register	Renamed from " pasientlopenr_pdb2471"	Created by Statistics Sweden Pseudonomised unique personal identification number for linkage between registers	Renamed from "lopnr"

For peer review only

Link to Contents

For peer review only - http://bmjopen.bmj.com/site/about/guidelines.xhtml

Link to *Contents*

b_date	Obtained from the Danish National Health Data Agency.<br>Register: “CPR-Registret”<br>Table: “t_person”<br>Variable: “d_foddato”	Renamed from “d_foddato”	Obtained from THL<br>Register: “Hilmo”<br>Table: “”<br>Variable: “SYNTAIKA”	Renamed from “SYNTAIKA”	Obtained from The National Population Register	We have received information on month and year of birth, but not day. For each individual, we have therefore generated a random integer between 1 and length of their month of birth. Using this random integer as day of birth, everyone is assigned an exact birth date.	Obtained from Statistics Sweden<br>Register: “RTB”<br>Variable: “fodddatum”	Renamed from “fodddatum”
sex	Obtained from the Danish National Health Data Agency.<br>Register: “CPR-Registret”<br>Table: “t_person”<br>Variable: “C_KON”<br>Sex as recorded by personal identification number.	sex=1 “male” if C_KON is “M”<br>sex=2 “female” if C_KON is “K”	Obtained from THL<br>Register: “Hilmo”<br>Table: “Perustiedot/Asiakas, potilas”<br>Variable: “SP”	Renamed from “SP”	Obtained from The National Population Register<br>Variable: “kjonn”	Renamed from “kjonn”	Obtained from Statistics Sweden<br>Register: “RTB”<br>Variable: “kon”	Renamed from “kon”<br><br>Note: There were some discrepancies regarding sex in the two registries (RTB and Patientregistret), circa 1100 cases regarding inpatients and circa 2900 regarding outpatients. We used the information from Statistics Sweden.

For peer review only

Link to *Contents*

Link to Contents

adm_date	Obtained from the Danish National Health Data Agency.<br>Register: Danish national patient registry<br>Table: T_ADM<br>Variable: D_INDDTO	Renamed from "D_INDDTO"<br><br>For outpatient contacts with multiple visits adm_date is recoded according to the date of visit ("D_AMBDTO" from the table "t_bes")	Obtained from THL<br>Register: "Hilmo"<br>Table: "Tulotiedot"<br>Variable: ""TUPVA"	Extracted from "TUPVA" which contain the date and time of arrival	Obtained from Register: Norwegian National Patient Register<br>Variable: "innDato"	Renamed from "innDato"	Obtained from Socialstyrelsen<br>Register: "Patientregistere t"<br>Variable: "INDATUM"	Renamed from "INDATUM".<br><br>Inpatient visits:<br>- Date missing (n=6); left unchanged.<br><br>- Date registered as earlier than birth (n=103);<br>-- dropped observations if both date of admission and discharge came before birth (n=8),<br>-- replaced date of admission with date of birth if less than 15 days apart (n=68),<br>-- replaced month or year, to align with date of discharge (n=29).<br><br>- Date registered as later than discharge but not missing (n=11); adm_date and discharge_date were shifted.<br><br>Outpatient visits:<br>- Date missing (n=1,253); left unchanged
discharge_date	Obtained from the Danish National Health Data Agency.<br>Register: Danish national patient registry<br>Table: T_ADM<br>Variable: D_UDDTO	Renamed from "D_UDDTO"<br><br>For contacts without a discharge date (N=1080) the discharge date is set as the last observed discharge date in the dataset+1day (11May2018)<br><br>For outpatient contacts, discharge date is recoded to be the same date as "adm_date".	Obtained from THL<br>Register: "Hilmo"<br>Table: "Poistumistiedot"<br>Variable: "LPVM"	Extracted from "LPVM" which contain the date and time of discharge	Obtained from Register: "Norwegian National Patient Register"<br>Variable: "utDato"	Renamed from "utDato". The data set only includes admissions that have ended, i.e. utDato before Dec 31, 2018.<br><br>75 contacts had missing utData. These were either outpatient contacts (n=69) or daycare procedures (n=6). utDato was defined innDato in these cases	Obtained from Socialstyrelsen<br>Register: "Patientregistere t"<br>Variable: ""UTDATUM"	For inpatient visits, the variable was renamed from "UTDATUM".<br><br>For outpatient visits, there was no corresponding variable, and the discharge date was therefore created to be equal to the admission date.

Link to Contents

For peer review only - http://bmjopen.bmj.com/site/about/guidelines.xhtml

Link to Contents

diag	Obtained from the Danish National Health Data Agency.<br>Register: Danish national patient registry<br>Table: T_DIAG<br>Variable: C_DIAG	Renamed from "C_DIAG"<br><br>Diagnosis coded as ICD 8 until December 31 1994, hereafter coded using ICD 10.<br><br>Danish specification letters to the ICD-10 codes removed and the administrative letter "D" in front of all codes removed: Values changed to string4 format (i.e DA011a→A011)<br><br>Diagnoses other than the main or other diagnoses are excluded. Diagnoses with modifications indicating that the diagnosis cannot be validated are excluded (c_diagmod==1 | 2).	Obtained from THL<br>Register: "Hilmo"<br>Table: "Hoitotiedot"<br>Variable: "PDGO, PDGE, SDGO, SDGE"	Renamed from PDGO, PDGE, SDGO, SDGE ICD-codes<br>ICD-codes V01-Y98 not available, codes O00-O99 were not analysed	Obtained from Register: "Norwegian National Patient Register"	Original dataset has one record for each hospital contact with variables hovedtilstand_1, hovedtilstand_2, bitilstand_1, …, bitilstand_19 that contain ICD 10 diagnosis codes.<br>The variables were renamed diag1, diag2, diag3, … where diag1 and diag2 correspond to the 2 primary diagnoses. The dataset was reshaped to long format containing one observation per diagnosis with variables diag, containing the ICD-10 codes and diag_ind = 1, 2, 3, …	Obtained from Socialstyrelsen<br>Register: "Patientregistere t"<br>Variables: "HDIA" and "DIAGNOS1_30"	The variable "DIAGNOS1_30" can contain up to 30 different diagnoses. It was therefore split to create separate variables for each sequential diagnosis. Duplicate codes within each observation and the code _atc were removed.
diagtype	Obtained from the Danish National Health Data Agency.<br>Register: Danish national patient registry<br>Table: T_DIAG<br>Variable: C_DIAGTYPE	Renamed from variable "C_DIAGTYPE"<br>Recoded:<br>C_DIAGTYPE: "A"= "main diagnosis"<br>C_DIAGTYPE: "B"= "other diagnosis"<br>A patient can have multiple other diagnoses for the same contact.<br>Excluding diagnoses other than main or other (i.e temporary diagnoses or additional diagnosis ("tillægsdiagnose").	Obtained from THL<br>Register: "Hilmo"<br>Table: "Hoitotiedot"<br>Variable: "PDGO, PDGE, SDGO, SDGE"	1=main diagnosis: PDGO and PDGE<br>2=add diagnosis: SDGO and SDGE		diagtype = 1 if diag_ind = 1 or diag_ind = 2<br>diagtype = 2 if diag_ind > 2	Obtained from socialstyrelsen<br>Register: "Patientregistere t"<br>Variables: "HDIA" and "DIAGNOS1_30"	Diagtype was coded as 1="Main diagnosis" if indicated in variable "HDIA". If no main diagnosis was listed in variable HDIA, the first diagnosis within variable "DIAGNOS1_30" was chosen as the main diagnosis. Other diagnoses listed within DIAGNOS1_30 were coded as 2="Other diagnosis".

Link to Contents

For peer review only - http://bmjopen.bmj.com/site/about/guidelines.xhtml

Link to Contents

type_contact	Obtained from the Danish National Health Data Agency.<br>Register: Danish national patient registry<br>Table: T_DIAG and t_bes<br>Variables: C_PATTYPE, D_AMBDTO	Renamed variable ”C_PATTYPE”<br><br>Recoded:<br>type_contact=1 “inpatient” if C_PATTYPE is “0” (inpatient) or “1” (Before year 2002 some patients were coded as “1= deldøgnspatienter” (≈part day patient)<br><br>type_contact=2 “emergency room contact” if C_PATTYPE is 3 “emergency room contact”.<br>Outpatient contacts (C_PATTYPE=2) admitted after year 2014 with “C_INDM”= “Acute” are coded as type_contact=2 ”emergency room patient”<br><br>type_contact=3 “outpatient contact” if C_PATTYPE=2 before year 2014 or C_PATTYPE=2 and c_indm is not 1 from and including year 2014<br><br>In Denmark we have some long outpatient contacts with multiple visit dates (D_AMBDTO) during the contact. Each visit date is coded as an independent outpatient contact. All diagnoses within the original outpatient contact is recorded for each visit.	Obtained from THL<br>Register: “Hilmo”<br>Table: “Perustiedot/Hoitojakso tai avohoitokäynti”<br>Variable: “PALA” and ”EA”	All visits with EA = 98 were omitted<br>(EA= special branches of medicine, 98=general practice)<br>- type_contact = 1, if PALA = 1 or PALA = 6 (inpatient)<br>- type_contact = 2, if PALA = 91 (emergency)<br>- type_contact = 3, if PALA is not 1, 6 or 91 (outpatient, not emergency)<br>PALA: 1 = inpatient ward, 6 = rehabilitation ward 91 = emergency room visit	Obtained from Register: “Norwegian National Patient Register”<br>Variable: “”	Based on the variables Behandlingsniva3 and Aktivitetskategori3:<br>For contacts with utDato in 2008-2014:<br><br>IF Behanglingsniva3 = 1 OR Behandlingsniva3 = 2 THEN type_contact = 1<br><br>ELSE IF Behandlingsniva3 = 3 THEN type_contact = 4<br>For contacts with utDato in 2015-2018:<br><br>IF Aktivitetsjkategori3 = 1 OR Aktivitetskategori3 = 2 THEN type_contact = 1<br>ELSE IF Aktivitetskategori3 = 3 THEN type_contact = 4	Obtained from Socialstyrelsen<br>Register: “Patientregistere t”	Variable coded based on which source file the data came from: in- or outpatient data. All data in the outpatient-file was coded = 4, as emergency room visits could not be distinguished.<br>(A variable for emergency room visits [VERKS_AKUT] was only included in the patient registry in 2016 and therefore not part of our data request.).

Link to Contents

For peer review only - http://bmjopen.bmj.com/site/about/guidelines.xhtml

Link to Contents

Table: population1

	Denmark		Finland		Norway		Sweden	
Variable	Source and Description	Important notes and data preparation	Source and Description	Important notes and data preparation	Source and Description	Important notes and data preparation	Source and Description	Important notes and data preparation
id	Original name in the Danish data: "pnr". Pseudonomised unique personal identification number for linkage between registers, created by Statistics Denmark. It is linkable (by Statistics Denmark) to the original personal identification number (CPR number) assigned to all Danish residents and used when reporting to all national registers.	Renamed from "pnr"	Obtained from: Register: Population register Table: VTJ.HENKILO Variable: hetu<br><br>Table: VTJ.HENKILO_HETU Variable: hetu_voimassa<br><br>THL pseudonymised the original personal identification code (in this register "hetu") to unique personal identification number for linkage between registers. THL data management can link the id back to original personal identification code.	Person included only if hetu_voimassa (=id is valid) is checked.<br><br>Statistics Finland pseudonymised "hetu" with their own id for the remote user system.	Obtained from: SSB (Statistics Norway) Register: "National Population Register"	Renamed from pasientlopenr_pdb2471	Created by Statistics Sweden Register: Registret över totalbefolkningen (RTB) Variable: lopnr	Pseudonomised unique personal identification number for linkage between registers<br><br>Renamed from "lopnr".
b_date	Obtained from the Danish National Health Data Agency. Register: "CPR-Registret" Table: "t_person" Variable: "d_foddato"	Renamed from "d_foddato"	Obtained from: Register: Population register Table:VTJ.HENKILO Variable: syntymapaiva	Renamed from syntymapaiva	Obtained from: SSB (Statistics Norway) Register: "National Population Register"	For all individuals in population1 as well as their parents, we have received information on month and year of birth, but not day. For each individual, we have therefore generated a random integer between 1 and length of their month of birth. Using this random integer as day of birth, everyone is assigned an exact birth date.	Obtained from Statistics Sweden Register: Registret över totalbefolkningen (RTB) Variable: fodddatum	Renamed from "fodddatum"

Link to Contents

For peer review only - http://bmjopen.bmj.com/site/about/guidelines.xhtml

Link to Contents

sex	Obtained from the Danish National Health Data Agency. Register: "CPR-Registret" Table: "t_person" Variable: "C_KON" Sex as recorded by personal identification number.	sex=1 "male" if C_KON is "M" sex=2 "female" if C_KON is "K"	Obtained from: Register: Population register Table: VTJ.HENKILO Variable: sukupuoli	sex=1 "male" if lapsen sukupuoli is "mies" sex=2 "female" if lapsen sukupuoli is "nainen"	Obtained from: SSB (Statistics Norway) Register: "National Population Register"	Renamed from kjonn	Obtained from Statistics Sweden Register: Registret över totalbefolkningen (RTB) Variable: kon	Renamed from "kon"

Link to Contents

For peer review only - http://bmjopen.bmj.com/site/about/guidelines.xhtml

Link to Contents

origin	Obtained from the Danish National Health Data Agency. Register: "CPR-Registeret" Table: "T_FODESTED" Variables: "fodested_kode", "fodested_tekst"<br><br>Variables from table: population1; in_date, cens_date are used to define if there is uncertain origin	Children are categorised as:<br>1="born in-country" if fodested_kode=000 or 208 (Denmark),<br>2= "foreign born" if fodested_kode is not 000 or 208<br>9="unknown" if fodested_kode=000 or 208 (Denmark) and if there is date of inmigration not preceded by an outmigration (In this case we cannot be certain that the child is born in Denmark as it appears to have migrated to Denmark after the date of birth)	Obtained from:<br>Register: Population register<br>Table:VTJ.HENKILO<br>Variable: "syntymakunta"	1 = born in country, if the code of syntymakunta (birth municipality is not 200 or NA (not available)<br>2 = born abroad, if syntymakunta is 200<br>3 = uncertain foreign or in-country, if syntymakunta is NA, 198,199 or 000 (children born abroad were excluded as only minority of them had immigration dates available in the THL's population register copy, in which the follow-up begin in 2014, also children with uncertain origin were excluded)	Obtained from: SSB (Statistics Norway)<br>Register: "National Population Register"	Based on the variables in_date (see below) and "fodeland". Origin is coded as 1 if country of birth is Norway (fodeland = 0) and in_date is equal to date of birth. Origin is coded as 2 if country of birth is any other country. Origin is coded as 9 if country of birth is Norway and in_date is later than date of birth.	Obtained from Statistics Sweden<br>Register: Registret över totalbefolkningen (RTB)<br>Variable: UtlSvBakg<br><br>Combined with data from the National Board of Health and Welfare<br>Register: Medical Birth Registry	Recoded from: "UtlSvBakg" where<br>11 = Born abroad<br>12 = Born in the country with two foreign-born parents<br>21 = Born in the country with one native and one foreign born parent<br>22 = Born in the country with two native born parents.<br><br>Individuals were coded 1 = "born in-country", if UtlSvBakg = 12, 21 or 22, and 2 = "born abroad", if UtlSvBakg = 11.<br><br>Individuals were coded 9 = "Unknown" if registered as born in country (UtlSvBakg = 12, 21 or 22) but also had a registered immigration date not preceded by an emigration date. (In this case we cannot be certain that the child was born in the country as it appeared that they have immigrated after the date of birth.)<br><br>If the individual was initially coded as 9 "Unknown", but was registered in the medical birth registry, they were recoded as 1 = "born in-country".

Link to Contents

For peer review only - http://bmjopen.bmj.com/site/about/guidelines.xhtml

Link to Contents

in_date	Obtained from the Danish National Health Data Agency. Register: "CPR-Registeret" Table: "T_FODESTED" Variables: "D_FODDATO"<br><br>Table: " T_ADRESSE_UDLAND_HIST " Variables: "C_ANNKOR", "D_INDREJSE_DATO" Table: " T_ARKIV_ADRESSE_UDLAND_HIST" Variables: "C_ANNKOR", "D_INDREJSE_DATO"<br><br>Obtained from NONSense CDM Table: Population1 Variable: "origin", "cens_date"	in_date is defined as date of birth "D_FODDATO" if "origin" is 1="born in-country" . in_date is defined as the first date of in-migration "D_INDREJSE_DATO" if origin is not 1="born in-country".	Obtained from: Register: Population register Table:VTJ.HENKILO Variable: "syntymapaiva" and	If born in country (origin=1), equal to the date of birth = syntymapaiva	Obtained from: SSB (Statistics Norway) Register: "National Population Register"	Based on the variables "regstatus", "regstatusdato", "forstdato" and "fodeland". Indate is defined as forstdato if invkat = B (immigrants). "forstdato" is the date of first registration in the Population Registry. The variable is only defined for persons with invkat =B (immigrants). Otherwise (invkat = A, C, E, F or G), indate is defined as a person's earliest regstatusdato with regstatus = 1 (Bosatt). In general, individuals who have been residents in Norway since birth, will be registered with regstatus = 1 and corresponding regstatusdato = date of birth. However, regstatus is only available as of January 1 each year. If a person's regstatus has changed more than once during a calendar year, we only have information about the most recent change. Therefore, in_date was set to date of birth for individuals with country of birth Norway who died or emigrated in their year of birth even if they do not have a record with regstatus = 1 and regstatusdato = date of birth. Note: cross-checked with the Birth Registry, and > 98% of children with country of birth	Variable created based on information from Statistics Sweden Register: Registret över totalbefolkningen (RTB) Variables: fodddatum and datum [migration], posttyp [migration]	If born in country (origin=1), equal to the date of birth = fodddatum If born outside the country (origin=2), equal to first date of immigration If unknown origin (=9), equal to first date of immigration

Link to Contents

For peer review only - http://bmjopen.bmj.com/site/about/guidelines.xhtml

Link to Contents

						Norway who died or emigrated in their year of birth have a record in the Birth Registry. Thus, it is a reasonable assumption that these children have been residents of Norway since birth.		
in_reason	Obtained from the Danish National Health Data Agency. Register: "CPR-Registeret"<br><br>Table: "T_FODESTED" Variables: "D_FODDATO"<br><br>Table: " T_ADRESSE_UDLAND_HIST " Variables: "C_ANNKOR", "D_INDREJSE_DATO"<br><br>Table: " T_ARKIV_ADRESSE_UDLAND_HIST" Variables: "C_ANNKOR", "D_INDREJSE_DATO"<br><br>Obtained from NONSense CDM Table: Population1 Variable:"in_date"	in_reason is categorised as:<br>1="birth" if in_date is obtained from "D_FODDATO"<br>2="immigration" if in_date is obtained from D_INDREJSE_DATO	Obtained from: Register: Population register Table:VTJ.HENKILO Variable: "syntymapaiva"	1 = Birth, if born in the country (origin=1)	Obtained from: SSB (Statistics Norway) Register: "National Population RegisterBefolkning	in_reason is coded as 1 if origin = 1. in_reason is coded as 2 if origin = 2 or origin = 9.	Variable created based on information from Statistics Sweden Register: Registret över totalbefolkningen (RTB) Variables: foddatum, datum, posttyp	1 = Birth, if born in the country (origin=1)<br>2 = Immigration, if born abroad (origin=2) or unknown origin (origin=9)

Link to Contents

For peer review only - http://bmjopen.bmj.com/site/about/guidelines.xhtml

Link to Contents

cens_date	Obtained from the Danish National Health Data Agency.<br>Register: "CPR-Registret"<br>Table: "t_person"<br>Variables: "D_STATUS_HEN_START", "C_STATUS"<br><br>Table: " T_ADRESSE_UDLAND_HIST "<br>Variables: "C_ANNKOR", "D_UDREJSE_DATO"<br><br>Table: " T_FORSVIND_HIST"<br>Variables: "C_ANNKOR", "D_FORSVIND_DATO"	Cens_date is defined as the first date of either 1)"D_STATUS_HEN_START" if "C_STATUS" is "90"=death, "20"=CPR number for tax purposes, "70"=disappearing, "80"=out-migration or 2) D_UDREJSE_DATO or 3) D_FORSVIND_DATO.	Obtained from:<br>Register: Population register<br>Table: VTJ.HENKILO<br>Variable: "KUOLINPVM" and<br>Register: Statistic Finland<br>Table:<br>Variable: "kuolinpäivä"<br>Variable: ensimmäinen maastamuuttopäivä<br><br>Table: VTJ.HENKILO<br>Table: KOTIKUNTAHISTORIA: Variable: "kotikunta" and "kunta muuttopaiva"	Equal to date of emigration, if such has occurred, otherwise equal to date of death.<br><br>Emigration from Population register (select min (kunta_muuttopvm) from vtj.henkilo_kotikuntahistoria and kunta='200')<br><br>Ensimmäinen maastamuuttopäivä=first emigration date available only in remote user system Fiona	Obtained from: SSB (Statistics Norway)<br>Register: "National Population RegisterBefolkning	Based on the variables "regstatus", "regstatusdato", and "dodsdato". Date of emigration was defined as a person's earliest regstatusdato with regstatus = 3 (emigration). Date of death was defined as dodsdato. We only have information on month and year of death. Exact date of date was assigned as a random integrer within the month of death.<br>cens_date was set to date of emigration if emigration occurred before date of 18th birthday or January 1, 2019. cens_date was set to date of death if death occurred before date of 18th birthday or January 1, 2019, unless date of death was preceded by date of emigration (N = 40).	Variable created based on information from Statistics Sweden<br>Register: Registret över totalbefolkningen (RTB)<br>Variables: Doddatum, datum [migration], posttyp [migration]	Equal to date of emigration, if such an event had been registered, otherwise equal to date of death.

For peer review only

For peer review only - http://bmjopen.bmj.com/site/about/guidelines.xhtml

Link to Contents

cens_reason	Obtained from the Danish National Health Data Agency. Register: "CPR-Registret" Table: "t_person" Variables: "D_STATUS_HEN_START", "C_STATUS"<br><br>Table: " T_ADRESSE_UDLAND_HIST " Variables: "C_ANNKOR", "D_UDREJSE_DATO"<br><br>Table: " T_FORSVIND_HIST" Variables: "C_ANNKOR", "D_FORSVIND_DATO"	Cens_reason is categorized as: 1= "death" if cens_date is obtained from C_STATUS="90" (death) 2="out-migration" if cens_date is obtained from C_STATUS="80" (outmigration) or from D_UDREJSE_DATO 3="other" if cens_date is obtained from C_STATUS="20" (CPR for tax purposes" | "70" (disappearing) or from D_FORSVIND_DATO.<br><br>If more than one cens_reason is registered for the first cens_date preference is given to 1="death"	Obtained from: Register: Population register Table: VTJ.HENKILO Variable:kuolinpvm, muuttopvm	1 = Death If subject died 2 = Emigration If subject emigrated	Obtained from: SSB (Statistics Norway) Register: "National Population RegisterBefolkning	cens_reason was coded as 1 if cens_date = date of death. cens_reason is coded as 2 if cens_date = date of emigration.	Variable created based on information from Statistics Sweden Register: Registret över totalbefolkningen (RTB) Variables: Doddatum, datum [migration], posttyp [migration]	1 = Death, if there was a date of death registered in variable Doddatum. 2 = Out migration, if there was a registered migration out of the country

For peer review only

Link to Contents

For peer review only - http://bmjopen.bmj.com/site/about/guidelines.xhtml

Link to Contents

m_id	Obtained from the Danish National Health Data Agency.<br>Register: “CPR-Registret”<br>Table: “t_person”<br>Variable: “V_MOR_PNR_ENCRYPTED”	Renamed from “V_MOR_PNR_ENCRYPTED”	Obtained from: Birth register<br>Table: Äidin henkilötiedot<br>Variable: aiti_hetunnus<br><br>THL pseudonymised the original personal identification code (in this register “aiti_hetunnus”) to unique personal identification number for linkage between registers. THL data management can link the id back to original personal identification code.	Renamed from “aiti_hetunnus” and pseudonymised by Statistics Finland for data lingage.	Obtained from: SSB (Statistics Norway)<br>Register: “National Population Register”<br>Variable: Løpenummer mor	Renamed from lopenr_mor_pdb2471	Obtained from Statistics Sweden<br>Register: Flergenerationsregistret	Renamed from “LopNrMor”
f_id	Obtained from the Danish National Health Data Agency.<br>Register: “CPR-Registret”<br>Table: “t_person”<br>Variable: “V_FAR_PNR_ENCRYPTED”	Renamed from “V_FAR_PNR_ENCRYPTED”	Obtained from: Statistics Finland	Not available for THL. Pseudonymised id for data linkage in Statistics Finland	Obtained from: SSB (Statistics Norway)<br>Register: “National Population Register”<br>Variable: Løpenummer far	Renamed from lopenr_far_pdb2471	Obtained from Statistics Sweden<br>Register: Flergenerationsregistret	Renamed from “LopNrFar”
m_age	Obtained from the Danish National Health Data Agency.<br>Register: “MFR” linked with-<br>Register: “CPR-Registeret”<br>Table: “t_person”<br>Variable: “d_foddato”	Id of the mother is obtained from the dataset “population1” (originally obtained from the CPR register). Using information on maternal birthday (d_foddato) and birthday of the child, Maternal age in years is calculated as age in whole years at time of delivery of the child.	Obtained from Register: Birth register<br>Table: Äidin henkilötiedot<br>Variable: aiti_ika	Renamed from aiti_ika	Obtained from: SSB (Statistics Norway)<br>Register: “National Population RegisterBefolkning	Mother’s age in whole years at time of birth of child. Based on the mother’s assigned exact date of birth (b_date).	Obtained from Statistics Sweden<br>Register: RTB<br>Variable: datum_fodd	Calculated as mother’s date of birth minus the child’s date of birth, divided by 365, and rounded down to yield age in years.

For peer review only

Link to Contents

m_origin	Obtained from the Danish National Health Data Agency. Register MFR Register: "CPR-Registeret" Table: "T_FODESTED" Variables: "fodested_kode", "fodested_tekst"<br><br>Variables from table: population1; in_date, cens_date are used to define if there is uncertain origin	Id of the mother is obtained from the dataset "population1" and linked with information from the CPR register 1="born in-country" if fodested_kode=000 or 208 (Denmark), 2= "born abroad" if fodested_kode is not 000 or 208 9="unknown" if information is missing	Obtained from Register: Statistics Finland Table: Variable: svaltio_aiti	Available only in the Fiona remote user system. svaltio_aiti = 246 -> 1 = "born in-country" svaltio_aiti != 246 (ts joku muu kuin Suomi) -> 2 = "born abroad" svaltio_aiti = NA (ts puuttuu) -> 9 = "Unknown"	Obtained from: SSB (Statistics Norway) Register: "National Population RegisterBefolk ning	Based on the variable "fodeland". m origin = 1 if mother's country of birth is Norway (fodeland = 0), m_origin = 2 if mother's country of birth is any other country, and m_origin = 9 if mother's country of birth is missing (n = 20,559).	Obtained from Statistics Sweden Register: RTB Variable: UtlSvBakg	Recoded from variable "UtlSvBakg" as described above for variable Origin in table Population 1.
p_origin	Obtained from the Danish National Health Data Agency. Register MFR Register: "CPR-Registeret" Table: "T_FODESTED" Variables: "fodested_kode", "fodested_tekst"<br><br>Variables from table: population1; in_date, cens_date are used to define if there is uncertain origin	Id of the father is obtained from the dataset "population1" and linked with information from the CPR register 1="born in-country" if fodested_kode=000 or 208 (Denmark), 2= "born abroad" if fodested_kode is not 000 or 208 9="unknown" if the information is missing	Obtained from Register: Statistics Finland Table: Variable: svaltio_isa	Available only in the Fiona remote user system. svaltio_isa = 246 -> 1 = "born in-country" svaltio_isa != 246 (ts joku muu kuin Suomi) -> 2 = "born abroad" svaltio_isa = NA (ts puuttuu) -> 9 = "Unknown"			Obtained from Statistics Sweden Register: RTB Variable: UtlSvBakg	Recoded from variable "UtlSvBakg" as described above for variable Origin in table Population 1.

For peer review only

Link to Contents

For peer review only - http://bmjopen.bmj.com/site/about/guidelines.xhtml

Link to Contents

Table: birth_charcteristics

	Denmark		Finland		Norway		Sweden	
Variable	Source and Description	Important notes and data preparation	Source and Description	Important notes and data preparation	Source and Description	Important notes and data preparation	Source and Description	Important notes and data preparation
id	Original name in the Danish data: “pnr”. Pseudonomised unique personal identification number for linkage between registers, created by Statistics Denmark. It is linkable (by Statistics Denmark) to the original personal identification number (CPR number) assigned to all Danish residents and used when reporting to all national registers.	Renamed from “pnr”	Obtained from Register: Birth Register Table: Variable: lapsi_hetunnus THL pseudonymised the original personal identification code (lapsi_hetunnus) to unique personal identification number for linkage between registers. THL data management can link the id back to original personal identification code.	Statistics Finland pseudonymised lapsi_hetunnus with their own id for the remote user system.		Renamed from “pasientlopenr_pdb2471”	Created by Statistics Sweden Pseudonomised unique personal identification number for linkage between registers	Renamed from lopnr

Link to Contents

For peer review only - http://bmjopen.bmj.com/site/about/guidelines.xhtml

Link to Contents

b_weight	Obtained from the Danish National Health Data Agency. Register: "MFR" from 1997 and onwards, "Fødselsregisteret" before 1997 Table: "MFR"(from MFR), "levendefødt" (From fødselsregisteret) Variable: "vaegt_barn" (MFR), V_VAGT (fødselsregisteret)	Renamed from "vaegt_barn" and " V_VAGT" Registrations of birthweight less than 100g or higher than 9990g are categorized as missing	Obtained from Register: Birth Register Table: Variable: syntymapaino	Registrations of birthweight less than 100g or higher than 9990g are categorized as missing	Obtained from Register: Medical Birth Registry of Norway Variable: vekt	Registrations of birthweight less than 100g or higher than 9990g are defined as missing	Obtained from Socialstyrelsen Register: Medicinska födelseregistret Variable: bvikt	Registrations of birthweight less than 100g or higher than 9990g were categorized as missing.

Link to Contents

For peer review only - http://bmjopen.bmj.com/site/about/guidelines.xhtml

Link to Contents

ga	Obtained from the Danish National Health Data Agency. Register: “MFR” from 1997 and onwards, “Fødselsregisteret” before 1997 Table: “MFR”(from MFR), ”levendefødt” (From fødselsregisteret) Variable: “Gestationsalder_dage” (MFR), ”V_SVLANGDE” (fødselsregisteret)	Derived from “Gestationsalder_dage” (ga in days) rounded down to whole weeks of gestation: ga=floor(gestationsalder_dage/7)<br><br>Renamed from V_SVLANGDE	Obtained from Register: Birth Register Table: Variable: kestovkpv	kestovkpv, ga will be notified as weeks, the days are not noted.<br><br>Ga <20 or >45 are coded as missing	Obtained from Register: Medical Birth Registry of Norway Variable: svlen	ga is calculated as floor(svlen/7), where svlen is the length of gestation in days based on ultrasound estimation. If ultrasound is not available, the gestational length is calculated from the last menstrual period.	Obtained from Socialstyrelsen Register: Medicinska födelseregistret Variable: grvbs	Socialstyrelsen recommends using this variable (for the best estimated gestational age), over the variable grvfv (which is based on medical records).

Link to Contents

For peer review only - http://bmjopen.bmj.com/site/about/guidelines.xhtml

Link to Contents

sectio	Obtained from the Danish National Health Data Agency.<br>Register: "MFR" from 1997 and onwards, "Fødselsregisteret" before 1997<br>Table: "MFR"(from MFR), "levendefødt" (From fødselsregisteret)<br><br>Variables: " Markoer_kejsersnit" (MFR), B_I11, B_SECTIOF, B_SECTIOU (fødselsregisteret)	From MFR:<br>0="not delivered by caesarean section" if they do not have any diagnosis code indicating caesarean section ("Markoer_kejsersnit"=missing)<br><br>1="delivered by caesarean section" if they have a diagnosis code indicating caesarean section in the variable "Markoer_kejsersnit"<br><br>Fødselsregisteret<br>sectio=1 if B_I11=1 | B_SECTIOU=1 | B_SECTIOF=1<br><br>Otherwise sectio=0	Obtained from Register: Birth Register<br>Table:<br>Variable: synnytystapatunnus	Children are categorised as:<br>0="not delivered by caesarean section" if synnytystapatunnus is 1-4<br><br>1="delivered by caesarean section" if synnytystapatunnus is 5-8<br><br>9="unknown" if synnytystapatunnus=9 or missing	Obtained from Register: Medical Birth Registry of Norway<br><br>Variable: ksnitt	Information on delivery with c-section is obtained from the variable ksnitt. Possible values of ksnitt are<br>1 = Planned C-section<br>2 = Emergency C-section<br>9 = Unspecified C-section<br>If ksnitt is missing, sectio is coded as 0. Otherwise, sectio is coded as 1.	Obtained from Socialstyrelsen<br>Register: Medicinska födelseregistret<br>Variable: secmark	Variable renamed from secmark; coding unaltered: 0 = no, 1 =yes.

Link to Contents

For peer review only - http://bmjopen.bmj.com/site/about/guidelines.xhtml

Link to Contents

smoke	Obtained from the Danish National Health Data Agency.<br>Register: “CPR-Registeret”<br>Register: “MFR” from 1997 and onwards, “Fødselsregisteret” before 1997<br>Table: “MFR”(from MFR), ”levendefødt” (From fødselsregisteret)<br>Variable: ”rygerstatus_moder” (MFR), B_RYGER (fødselsregisteret)<br><br>Obtained from the Danish National Health Data Agency.<br>Register: Danish national patient registry<br>Table: “T_ADM”, “T_DIAG”<br>Variables: pnr, recnum, D_INDDTO, D_UDDTO, C_ADIAG, C_TILDIAG,	Information from MFR, variable “RYGERSTATUS_MODER”<br>Smoke=0 if rygerstatus_moder=0<br>Smoke=1 if rygerstaus moder >0 and <99 (indicating any smoking during pregnancy regardless of magnitude)<br>Smoke=9 if rygerstatus_moder=99(unknown) or missing.<br><br>From fødselsregisteret:<br>smoke=0 if B_RYGER=0<br>smoke=1 if B_RYGER=1<br>smoke=9 if B_RYGER=.<br><br>For some pregnancies especially in 1997 and partially in 1998, smoke information is not available in MFR, but we are able to subtract the information from the patient registry using the additional diagnosis “DUT00-DUT99”.<br><br>Information about smoke is inserted from the patient registry if: a) the information is not present in MFR/fødselsregisteret; b) if the patient registry indicates smoking while MFR/fødselsregisteret indicates no smoking or unknown.	Obtained from Register: Birth Register<br>Table:<br>Variable: tupakointitunnus	Smoke=0 if tupakointitunnus=1<br>Smoke=1 if tupakointitunnus =2-4<br>Smoke=9 if tupakointitunnus=9 (unknown) or missing.	Obtained from Register: Medical Birth Registry of Norway<br>Variable: royk_beg and royk_avsl	Information on smoking at start and end of pregnancy is obtained from royk_beg and royk_avsl, respectively. Both variables are coded as<br>1 = No<br>2 = Sometimes<br>3 = Daily<br>If royk_beg = 1 AND royk_avsl = 1, smoke is coded as 0<br>If royk_beg = 2 OR royk_beg = 3 OR royk_avsl = 2 OR royk_avsl = 3, smoke is coded as 1<br>Otherwise smoke = 9.<br>Mothers can opt out of having information on smoking recorded. Thus, royk_beg and royk_avsl is missing for a high proportion of births. The proportion with smoke = 9 is 43%.	Obtained from Socialstyrelsen<br>Register: Medicinska födelseregistret<br>Variable: rok1	The variable rok1 pertains to smoking habits at registration with maternal health (usually at 8-12 weeks of pregnancy).<br><br>If the woman was smoking >=1 cigarette/day at registration (rok1 coded 2 or 3), the variable smoke was coded = 1.<br>If the woman was not smoking (rok1 coded 1) the variable smoke was coded = 0.<br>If data was missing the variable smoke was coded = 9 (missing).<br><br>(There is another variable, rok2, which pertains to smoking habits at pregnancy week circa 30-32. This was not included due to very poor data quality 1990-1999, and poor completeness thereafter (Source publication: Graviditeter, förlossningar och nyfödda barn (socialstyrelsen.se) , Statistikdatabaser - Förlossningsstatistik - Val (socialstyrelsen.se))

Link to Contents

For peer review only - http://bmjopen.bmj.com/site/about/guidelines.xhtml

Link to Contents

singleton	Obtained from the Danish National Health Data Agency.<br>Register: "CPR-Registeret"<br>Register: "MFR" from 1997 and onwards, "Fødselsregisteret" before 1997<br>Table: "MFR"(from MFR), "levendefødt" (From fødselsregisteret)<br>Variable: "Flerfoldsgraviditet" (MFR), C_PLAC (fødselsregisteret)	MFR<br>Children are categorized as:<br>0="no" if there is an indication of multiple child delivery (diagnosis code) or there is registered another child born by the same mother within 1 day from the child's birthday<br><br>1="yes" if there is no indication of multiple child delivery ("Flerfoldsgraviditet"=missing)<br><br>Fødselsregisteret<br>Children are categorized as:<br>0="no" if C_PLAC>0 or there is registered another child born by the same mother within 1 day from the child's birthday<br><br>1="yes" if C_PLAC=0 and no child born by the same mother within 1 day from the child's birthday	Obtained from Register: Birth Register<br>Table:<br>Variable: sikioita	Children are categorized as:<br>0="no" if sikioita=2 or more<br><br>1="yes" if sikioita=1	Obtained from Register: Medical Birth Registry of Norway<br>Variable: flerfodsel	singleton is coded as 0 if flerfodsel = 1 or if another child is born to the same mother in the same month (N = 13). Otherwise, singleton is coded as 1.	Obtained from Socialstyrelsen<br>Register: Medicinska födelseregistret<br>Variable: bordf2	1="Enkelbörd" was left unaltered (=1 "Yes").<br>2="Flerbörd" was recoded to 0 "No".

For peer review only

Link to Contents

For peer review only - http://bmjopen.bmj.com/site/about/guidelines.xhtml

Link to Contents

child_order	Obtained from the Danish National Health Data Agency. Register: “CPR-Registeret” Register: “MFR” from 1997 and onwards, “Fødselsregisteret” before 1997 Table: “MFR”(from MFR), ”levendefødt” (From fødselsregisteret) Variable: ”paritet” (MFR), V_TIDLLEV, V_TIDLDOD (fødselsregisteret)	The variable from MFR contains information on number of fulfilled pragnancies including stillbirths. Before 1997 the variables V_TIDLLEV(previous live births)+V_TIDLDOD (previous still births) has been added plus 1(current delivery), to simulate the information from MFR.<br><br>Second, a counting method is applied using the registered parity indication for the first registered child and counting onwards for following liveborn children. Preparation is done in 3 steps:<br>1) parity of the first registered child is determined:<br>a) missing information on the first registered child by a mother but with information on the second registered child are recoded with parity of the second child minus 1.<br>b) children with missing information on the first registered child are recoded with parity=1 if the second child is registered as parity=1.<br>2) child order of following children is determined using a counting method	Obtained from Register: Birth Register Table: Variable: aiemmatsynnytykset Variable: kuolleenasynt	Number of the child =“Aiemmatsynnytykset” (previous births) minus “kuolleenasynt” (=stillbirths) plus 1 multiple delivered children are identified, and parity is recoded to the lowest value i.e., twins with 1 older sibling will both be coded with child order=2	Obtained from Register: Medical Birth Registry of Norway Variable: paritet	parity is defined as paritet + 1. The variable paritet is defined by MBRN as the highest value of the variables paritet_mor and paritet_mfr, where paritet_mor is number of previous deliveries as stated by mother and paritet_mor is number of previous deliveries registered by MBRN. Stillbirths are included in paritet.<br><br>Pairs of twins should have the same value of parity and will therefore be assigned the same value of parity (lowest within the set).	Obtained from Socialstyrelsen Register: Medicinska födelseregistret Variable: paritet	The child’s order, based on the number of children previously born by the mother, including this birth. Twins were given the same number, the lowest within the set.

Link to Contents

For peer review only - http://bmjopen.bmj.com/site/about/guidelines.xhtml

Link to Contents

		from the parity of the first registered child plus 1 for each following child<br>3) multiple delivered children are identified, and child order is recoded to the lowest value i.e., twins with 1 older sibling will both be coded with parity=2						

For peer review only

Link to Contents

For peer review only - http://bmjopen.bmj.com/site/about/guidelines.xhtml

Link to Contents

Table: Vaccines

	Denmark		Finland		Norway		Sweden	
Variable	Source and Description	Important notes and data preparation	Source and Description	Important notes and data preparation	Source and Description	Important notes and data preparation	Source and Description	Important notes and data preparation
id	Original name in the Danish data: "pnr". Pseudonomised unique personal identification number for linkage between registers, created by Statistics Denmark. It is linkable (by Statistics Denmark) to the original personal identification number (CPR number) assigned to all Danish residents and used when reporting to all national registers.	string	Obtained from Register: Vaccination Register Table: Variable: hetu<br><br>THL pseudonymised the original personal identification code (hetu) to unique personal identification number for linkage between registers. THL data management can link the id back to original personal identification code.	Statistics Finland pseudonymised "hetu" with their own id for the remote user system.	Obtained from Register: Norwegian Immunisation Registry (SYSVAK)	string	Created by Statistics Sweden Pseudonomised unique personal identification number for linkage between registers	String
vacdate	Obtained from the state serum institue. Register: "vaccinationsregisteret" Variable: "EffectuationDate"	Date Format (%dD_m_Y)	Obtained from Register: Vaccination Register Table: Variable: Recorddate	Date Format (%dD_m_Y)	Obtained from Register: Norwegian Immunisation Registry (SYSVAK) Variable: konsultasjonsdato	Renamed from konsultasjonsdato Date Format (%dD_m_Y)	Obtained from The Public Health Agency of Sweden (PHAS) Register: The National Vaccination Registry (NVR) Variable: vaccination_date	Date Format (%dD_m_Y)

Link to Contents

For peer review only - http://bmjopen.bmj.com/site/about/guidelines.xhtml

Link to *Contents*

vaccine	Obtained from the state serum institue.<br>Register: "vaccinationsregisteret"<br>Variable: "ATCCode"	Categorical (see coding in appendix "vaccine categorization")<br>Duplicates were handled as follows, so that only one entry was kept:<br>- same ATCCode: duplicate removed.<br>- same group of vaccines (see appendix vaccine categorization) within 14 days: the entry most likely to have been administered according to the national vaccination schedule at the time was kept.<br>-Hib-vaccine given within 14 days of a multivalent Hib-containing vaccine: was removed<br>- IPV given within 14 days of an multivalent IPV-containing vaccines: was removed	Obtained from Register: Vaccination Register<br>Table:<br>Variable: atc_code	Categorical (see coding in appendix "vaccine categorization")<br>Duplicates were handled as follows, so that only one entry was kept:<br>- same ATCCode: duplicate removed.<br>- same group of vaccines (see appendix vaccine categorization) within 14 days: the entry most likely to have been administered according to the national vaccination schedule at the time was kept.<br>-Hib-vaccine given within 14 days of a multivalent Hib-containing vaccine: was removed<br>- IPV given within 14 days of an multivalent IPV-containing vaccines: was removed	Obtained from Register: Norwegian Immunisation Registry (SYSVAK)<br>Variable: vaksinekode	Categorical (see coding in appendix "vaccine categorization")<br>Duplicates by same ATCCode are removed.<br>Duplicates by same group of vaccines (see appendix vaccine categorization) within 14 days are cleaned based on information on which vaccine is most likely to have been administered according to the national vaccination schedule and historical changes.<br>Hib given within 14 days of Hib containing vaccines are removed<br>IPV given within 14 days of IPV containing vaccines are removed	Obtained from: PHAS<br>Register: NVR<br>Variable: atc, product_name	Categorical (see coding in appendix "vaccine categorization")<br>Duplicates were handled as follows, so that only one entry was kept:<br>- same ATCCode: duplicate removed.<br>- same group of vaccines (see appendix vaccine categorization) within 14 days: the entry most likely to have been administered according to the national vaccination schedule at the time was kept.<br>-Hib-vaccine given within 14 days of a multivalent Hib-containing vaccine: was removed<br>- IPV given within 14 days of an multivalent IPV-containing vaccines: was removed

For peer review only

Link to *Contents*

For peer review only - http://bmjopen.bmj.com/site/about/guidelines.xhtml

Link to *Contents*

credibility	Variable generated based on data preparation	1=no duplicate<br><br>2= duplicate same vaccine removed<br><br>3=duplicate related vaccine removed (keep vaccine that aligns with vaccination schedule)<br><br>4= duplicate related vaccine removed (none of the vaccines align with vaccination schedule)<br><br>5= duplicate related vaccine removed (vaccines given outside usual vaccination ages within the national immunisation programme)	Variable generated based on data preparation	1=no duplicate<br><br>2= duplicate same vaccine removed<br><br>3=duplicate related vaccine removed (keep vaccine that aligns with vaccination schedule)<br><br>4= duplicate related vaccine removed (none of the vaccines align with vaccination schedule)<br><br>5= duplicate related vaccine removed (vaccines given outside usual vaccination ages within the national immunisation programme)	Variable generated based on data preparation	1=no duplicate<br><br>2= duplicate same vaccine removed<br><br>3=duplicate related vaccine removed (keep vaccine that aligns with vaccination schedule)<br><br>4= duplicate related vaccine removed (none of the vaccines align with vaccination schedule)<br><br>5= duplicate related vaccine removed (vaccines given outside usual vaccination ages within the national immunisation programme)	Variable generated based on data preparation	1=no duplicate<br><br>2= duplicate same vaccine removed<br><br>3=duplicate related vaccine removed (keep vaccine that aligns with vaccination schedule)<br><br>4= duplicate related vaccine removed (none of the vaccines align with vaccination schedule)<br><br>5= duplicate related vaccine removed (vaccines given outside usual vaccination ages within the national immunisation programme)
TB_endemic		9= not relevant		9= not relevant		9=not relevant	Obtained from Statistics Sweden Register: RTB Variable: fodelselandnamn	If the child, mother OR father was born in a country with high or very high incidence of tuberculosis ie. >25 cases per 100,000 inhabitants (as listed in WHO:s Global TB report 2018, link), the child was coded 1=risk group, as this corresponds to eligibility for BCG-vaccination.<br><br>All other children were coded = 0.

Link to *Contents*

For peer review only - http://bmjopen.bmj.com/site/about/guidelines.xhtml

Link to Contents

HepB_endemic		9=not relevant		9=not relevant		9=not relevant	Obtained from Statistics Sweden Register: Table: Variable: fodelselandnamn	If the child, mother OR father was born in a country with an intermediary or high prevalence of hepatitis B in the population (> 2 percent HbsAg-positive), the child was coded 1=risk group. * If the child and both parents came from low prevalence countries, the child was coded = 0. (This included all native-born children.) If the child came from a country with an unknown prevalence, it was coded as missing.

* Source: Schweitzer A, Horn J, Mikolajczyk RT, Krause G, Ott JJ. Estimations of worldwide prevalence of chronic hepatitis B virus infection: a systematic review of data published between 1965 and 2013. The Lancet. 2015;386(10003):1546-55. DOI:https://doi.org/10.1016/S0140-6736(15)61412-X.

For peer review only

Link to Contents

For peer review only - http://bmjopen.bmj.com/site/about/guidelines.xhtml

Link to Contents

Table: socio_economy

	Denmark		Finland		Norway		Sweden	
Variable	Source and Description	Important notes and data preparation	Source and Description	Important notes and data preparation	Source and Description	Important notes and data preparation	Source and Description	Important notes and data preparation
id	Original name in the Danish data: “pnr”. Pseudonomised unique personal identification number for linkage between registers, created by Statistics Denmark. It is linkable (by Statistics Denmark) to the original personal identification number (CPR number) assigned to all Danish residents and used when reporting to all national registers.	Renamed from “pnr”	Obtained from Register: Population register Table: Variable: hetu THL pseudonymised the original personal identification code (lapsi_hetunnus) to unique personal identification number for linkage between registers. THL data management can link the id back to original personal identification code.	Statistics Finland pseudonymised “hetu” with their own id for the remote user system.		Renamed from “pasientlopenr_pdb2471”	Created by Statistics Sweden Pseudonomised unique personal identification number for linkage between registers Variable: lopnr	Renamed from lopnr

For peer review only

Link to Contents

For peer review only - http://bmjopen.bmj.com/site/about/guidelines.xhtml

Link to Contents

inc_quin_b	Obtained from Statistics Denmark.<br>Table: "FAIK" (tables for each year)<br>Variable: "FAMAEKVIVADISP_13" (Equated disposable family income)<br><br>Link between each child and family is obtained from Statistics Denmark:<br>Table: BEF (tables for each year)<br>Link variable: FAMILIE_ID (combined with calendar year)	Birth year 2016 and higher do not have information on family income at birth.<br>No children have information from the year they are born, because the statistics are made on the first of January each year. Include information from the year after birth. If no info from that year, the child is coded with unknown (9).<br>Note: quintiles made separately for each calendar year for the children born the year before.	Obtained from Register: Statistics Finland<br>Table:<br>Variable: kturaha_ak_lapsi	Only available in Fiona remote user system.<br>Renamed from "kturaha_ak_lapsi" at the year when child was born.<br>Calculation of quintiles are done separately for each calendar year.<br>E.g. calculating income quintiles for 2008 include all children who use income information from 2008 to assess the income quintile at birth<br>kturaha_ak_lapsi = NA, coded as 9 = "Unknown"	Obtained from Statistics Norway<br>Variable: ies_eu	Based on the variable "ies_eu", defined as total after-tax income for the household per consumption unit calculated according to the EU scale. Total after-tax income is calculated as the sum of the household's wages and salaries, income from self-employment, property income and transfers received minus total assessed taxes and negative transfers. Each income year includes all persons residing in Norway and resident in a private household as of 31st December of the income year. Household income in year of birth is used to define inc_quin_b. Income quintiles are made separately for each birth cohort. Available for children born 2004–2018.	Obtained from Statistics Sweden<br>Register: Longitudinell integrationsdatabas för Sjukförsäkrings- och Arbetsmarknadsstudier (LISA)<br>Variable: DispInkFam	In Sweden, disposable income is defined as the sum of all household members' all forms of income (including wages, capital gains, and different forms of financial support/social assistance) minus taxes and other negative transfers (Statistikskolan: Att jämföra inkomster för hushåll (scb.se)).<br>The information *primarily* came from the information registered for the household of the mother in the year of birth of the child. If this was missing, the information was instead taken from the father. Thus, the child was primarily assumed to be part of the mother's household, and secondly of the father's.<br>Income quintiles was then calculated based on all children in each birth cohort.

For peer review only

Link to Contents

Link to Contents

inc_quin_10y	Obtained from Statistics Denmark.<br>Table: "FAIK" (tables for each year)<br>Variable: "FAMAEKVIVADISP_13" (Equated disposable family income)<br><br>Link between each child and family is obtained from Statistics Denmark:<br>Table: BEF(tables for each year)<br>Link variable: FAMILIE_ID (combined with calendar year)	Birth year 2007 and higher do not have info on family income at ten years.<br>If no info from the year the child turn 10 years the variable is coded with unknown (9).<br>Note: quintiles made separately for each calendar year for the children turning 10 years that year.	Obtained from Register: Statistics Finland<br><br>Table:<br>Variable: kturaha_ak_lapsi	Only available in Fiona remote user system. Renamed from "kturaha_ak_lapsi" at the year when child was 10 years old. Calculation of quintiles are done separately for each calendar year. E.g. calculating income quintiles for 2008 include all children who use income information from 2008 to assess the income quintile at birth kturaha_ak_lapsi = NA, coded as 9 = "Unknown"	Obtained from Statistics Norway<br>Variable: ies_eu	Based on the variable "ies_eu", see definition above. Household income in the year of the child's 10th birthday is used to define inc_quin_10y. Income quintiles are made separately for each birth cohort. Available for children born 1994–2008.	As above.	As above, but from the year the child turned 10 years old.
inc_quin_m_b					Obtained from Statistics Norway<br>Variable: wies	Based on the variable "wies", defined as a person's after-tax income. After-tax income is calculated as the sum of wages and salaries, income from self-employment, property income and transfers received minus total assessed taxes and negative transfers. The mother's income in the child's year of birth is used to define inc_quin_m_b. Income quintiles are made separately for each birth cohort. Available for children born 1993–2018.	Obtained from Statistics Sweden<br>Register: Longitudinell integrationsdatabas för Sjukförsäkrings- och Arbetsmarknadsstudier (LISA)<br>Variable: DispInk	Information about disposable income of the mother in the year of birth of the child.<br><br>Income quintiles was then calculated based on all children in each birth cohort.<br><br>See also above.

Link to Contents

For peer review only - http://bmjopen.bmj.com/site/about/guidelines.xhtml

Link to Contents

inc_quin_m_10y					Obtained from Statistics Norway Variable: wies	Based on the variable "wies", see definition above. The mother's income in the year of the child's 10th birthday is used to define inc_quin_m_10y. Income quintiles are made separately for each birth cohort. Available for children born 1990–2008.	As above.	As above, but from the year the child turned 10 years old.
n_children_b	Obtained from Statistics Denmark. Table: "FAM" (tables for each year) Variables: Sumarized from the variables ANTB00-ANTB17 (number of children in the family age 0, 1, 2…,17)<br><br>Link between each child and family is obtained from Statistics Denmark: Table: BEF(tables for each year) Link variable: FAMILIE_ID (combined with calendar year)	Birth year 2018 do not have info on number of children at birth. No children have information from the year they are born, because the statistics made on the first of January each year. Include information from the year after birth. If no info from that year the child is code with unknown (99). Some children end-up with a count of 0 children, as this is not a legal value they are recoded to 99. Based on the values on family_type, it is judged that the children with a count of 0, are children who are registered as the main person in a family and therefore are not counted as a child although they are children.	Obtained from Register: Statistics Finland<br><br>Table: Variable: lkm_lapsi	Only available in Fiona remote user system. Renamed from "lkm_lapsi" at the year when child was born.	Obtained from Statistics Norway Variable: barn_i_regstat_famnr	Based on variable "barn_i_regstat_famnr", number of children in the family. Persons are considered children if they are below 18 years and registered as resident in the family of at least one parent. A family is defined as persons resident in the same dwelling and related to each other as spouse, registered partner, cohabitant, and/or parent and child (regardless of the child's age). At most, a family may consist of two subsequent generations and one couple only. The variable includes residents of Norway as of January 1 each year. We have therefore used number of children in the year after the child's year of birth. Individuals registered with 0 number of children in their family have been recoded to 1. Available for children born 2004–2018.	Obtained from Statistics Sweden Register: LISA Variable: Barn0_3, Barn4_6, Barn7_10, Barn11_15, Barn16_17	Created as the sum of children in variables Barn0_3, Barn4_6, Barn7_10, Barn11_15 and Barn16_17. The sum denotes the number of children living in the household on 31 Dec in the year of birth of the child. The child itself is part of the count.<br><br>The information *primarily* came from the information registered for the mother in the year of birth of the child. If this was missing, the information was instead taken from the father.

Link to Contents

For peer review only - http://bmjopen.bmj.com/site/about/guidelines.xhtml

Link to Contents

n_children_10y	Obtained from Statistics Denmark.<br>Table: "FAM" (tables for each year)<br>Variables: Sumarized from the variables ANTB00-ANTB17 (number of children in the family age 0, 1, 2…,17 years)<br><br>Link between each child and family is obtained from Statistics Denmark:<br>Table: BEF(tables for each year)<br>Link variable: FAMILIE_ID (combined with calendar year)	Birth year 2009 and higher do not have info on number of children at 10 years. If no info from the year the child turn 10 years the variable is coded with unknown (99). Some children end-up with a count of 0 children, as this is not a legal value they are recoded to 99. Based on the values on family_type, it is judged that the children with a count of 0, are children who are registered as the main person in a family and therefore are not counted as a child although they are children.	Obtained from Register: Statistics Finland<br><br>Table:<br>Variable: lkm_lapsi	Only available in Fiona remote user system. Renamed from "lkm_lapsi" at the year when child was 10 years old.	Obtained from Statistics Norway<br>Variable: barn_i_regstat_famnr	Based on variable "barn_i_regstat_famnr", see above. The variable includes residents of Norway as of January 1 each year. We have therefore used number of children in the year after the year of the child's 10th birthday. Individuals registered with 0 number of children in their family have been recoded to 1. Available for children born 1994–2009.	As above.	As above, but from the year the child turned 10 years old.

Link to Contents

For peer review only - http://bmjopen.bmj.com/site/about/guidelines.xhtml

Link to Contents

single_parent_b	Obtained from Statistics Denmark.<br>Table: "FAM" (tables for each year)<br>Variable: FAMILIE_TYPE<br><br>Link between each child and family is obtained from Statistics Denmark:<br>Table: BEF(tables for each year)<br>Link variable: FAMILIE_ID (combined with calendar year)	Birth year 2018 do not have info on single parenthood at birth. No children have information from the year they are born, because the statistics made on the first of January each year. Include information from the year after birth. If no info from that year the child is code with unknown (9).<br>I also set children who originally were coded with 0 children on n_children_b as unknown (9) because it is judgded that these are children registered as the main person in the family (no adults in the family?).	Obtained from Register: Statistics Finland<br><br>Table:<br>Variable: pety_lapsi	Only available in Fiona remote user system. Calculated from "pety_lapsi" at the year when child was born.<br>If pety_lapsi is 2 (married couple and children) or 5-6 (couple with children) -> single parent =0 (no)<br>If pety_lapsi is 3 or 4 (mother or father with children) -> single parent = 1 (yes).<br>If pety_lapsi is unknown -> single parent = 9	Obtained from Statistics Norway<br>Variable: regstat_famtyp	Based on the variable "regstat_famtyp", a detailed classification of family type, where family is defined as described above. The variable includes residents of Norway as of January 1 each year. We have therefore used the value of family type in the year after a child's year of birth to define single_parent_b. If the registered family type is either "married couple with small children (youngest child aged 0-5 years)" or "cohabitants with small children (youngest child aged 0-5 years)", single_parent_b is coded as 0. If the registered family type is either "mother with small children (youngest child aged 0-5 years)" or "father with small children (youngest child aged 0-5 years)", single_parent_b is coded as 1. Otherwise (family type is any other category or missing), single_parent_b is coded as 9. Available for children born 2004–2018.	Obtained from Statistics Sweden<br>Register: LISA<br>Variable: FamTypF	The information came from the information registered for the mother in the year of birth of the child.<br><br>Codes FamTypF=41, 42 classifies the mother as a single parent, and 50 denotes Other singles. These codes were included when coding single_parent_b=1 (yes). If FamTypF was missing, single_parent_b was coded as 9 (missing). All other FamTypF-codes were recoded as 0 (no).

For peer review only

Link to Contents

For peer review only - http://bmjopen.bmj.com/site/about/guidelines.xhtml

Link to Contents

single_parent_10y	Obtained from Statistics Denmark.<br>Table: "FAM" (tables for each year)<br>Variable: FAMILIE_TYPE<br><br>Link between each child and family is obtained from Statistics Denmark:<br>Table: BEF(tables for each year)<br>Link variable: FAMILIE_ID (combined with calendar year)	Birth year 2018 do not have info on single parenthood at birth.<br>If no info from the year the child turn 10 years the variable is coded with unknown (9).<br>set children who originally were coded with 0 children on n_children_10y as unknown (9) because it is judged that these are children registered as the main person in the family (no adults in the family?).	Obtained from Register: Statistics Finland<br><br>Table:<br>Variable: pety_lapsi	Only available in Fiona remote user system.<br>Calculated from "pety_lapsi" at the year when child was 10 years old.<br>If pety_lapsi is 2 (married couple and children) or 5-6 (couple with children) -> single parent =0 (no)<br>If pety_lapsi is 3 or 4 (mother or father with children) -> single parent = 1 (yes).<br>If pety_lapsi is empty -> single parent = 9 (unknown)	Obtained from Statistics Norway<br>Variable: regstat_famtyp	Based on the variable "regstat_famtyp", see above. The variable includes residents of Norway as of January 1 each year. We have therefore used the value of family type in the year after the year of a child's 10th birthday to define single_parent_10y. If the registered family type is either "married couple with small children (youngest child aged 0-5 years)", "married couple with older children (youngest child aged 6-17 years) "cohabitants with small children (youngest child aged 0-5 years)", or "cohabitants with older children (youngest child aged 6-17 years)", single_parent_10y is coded as 0. If the registered family type is either "mother with small children (youngest child aged 0-5 years)", "mother with older children (youngest child aged 6-17 years)", "father with small children (youngest child aged 0-5 years)", or "father with older children (youngest child aged 6-17 years)", single_parent_10y is coded as 1. Otherwise (family type is any other category or missing), single_parent_10y is coded as 9. Available for children born 1994–2009.	As above.	As above, but from the year the child turned 10 years old.

Link to Contents

For peer review only - http://bmjopen.bmj.com/site/about/guidelines.xhtml

Link to Contents

m_education_b	Obtained from Statistics Denmark.<br>Table: "UDDF"<br>Variable: "hfaudd" "hfaudd" i linked with format from statistics Denmark grouping the Danish education classification into ISCED 2011, based on which maternal education is grouped.<br><br>Link to mother is available from the dataset "population1" (originally obtained from the CPR register).	Use the highest obtained education for the mother on the date of birth of the child. There is no information on this for children born 2017 or later. Statistics Denmark had a format available for transforming national Danish education codes into ISCED.	Obtained from Register: Statistics Finland<br><br>Table: Variable:ututku_aiti and koulutusaste_taso_1 and birthday of child obtained from population1	Education is classified by ISCED-11, although the classes 0-2 are not available for us. In Finland, we have compulsory education during which the ISCED level 2 is achieved and thus we classified education: NA= 1 low education level<br>3-4 = 2 medium education level<br>5-8 = 3 high education level<br>9 = no information of the mother's Education at child's birth year.	Obtained from Statistics Norway Variable: bu_niva_YYYY	Based on the variables "bu_niva_YYYY". The variables contain information on highest level of education as of October 1 of the year YYYY. Mother's level of education from the child's year of birth was used to define m_education_b. Education is classified according to The Norwegian Standard Classification of Education (NUS). If the NUS-level is 0 (corresponding to ISCED2011 levels 01, 02), 1 or 2 (corresponding to ISCED2011 level 1 and 2, respectively), m_education is coded as 1. If the NUS level is 3 or 4 (corresponding to ISCED2011 = 3), m_education is coded as 2. If the NUS-level is 6, 7 or 8 (corresponding to ISCED2011 level 6, 7, and 8, respectively), m_education is coded as 3 (https://www.ssb.no/utdanning/artikler-og-publikasjoner/_attachment/240569?_ts=150ebb996e0, page 25). NUS-level = 5 is defined as tertiary vocational educational level not approved as higher education. Tertiary education with duration less than 2 years corresponds to ISCED2011 level 4. In this case, m_educaion_b should be coded as 2. Tertiary education with duration of 2 years corresponds to ISCED2011 level 5, and m_education_b should be coded as 3. However, we do not have information on type or duration of the tertiary	Obtained from Statistics Sweden Register: LISA Variable: Sun2000niva	The variable denotes the highest level of education achieved during the spring semester in the year the child was born. That means, that if the mother achieved a higher level of education mid-year, it will only be visible in the register for the following year.<br>Level of education was recoded from Sun2000 to ISCED by a translational key available from Statistics Sweden: Svensk utbildningsnomenklatur (SUN) (scb.se) (retrieved 2021-08-20).

Link to Contents

For peer review only - http://bmjopen.bmj.com/site/about/guidelines.xhtml

Link to Contents

						education. In 2016, 83.6% of women graduating from tertiary vocational education, had finished an education with duration of 2 years, while only 16.4% had finished an education with duration less than 2 years(https://www.ssb.no/en/statbank/table/11635). Therefore, m_education_b was coded as 2 if the NUS-level was 5. Available for children born 1990–2018.		

Link to Contents

For peer review only - http://bmjopen.bmj.com/site/about/guidelines.xhtml

Link to Contents

m_education_10y	Obtained from Statistics Denmark.<br>Table: “UDDF”<br>Variable: ”hfaudd”<br>”hfaudd” i linked with format from Satistics Denmark grouping the Danish education classification into ISCED 2011, based on which maternal education is grouped.<br><br>Link to mother is available from the dataset “population1” (originally obtained from the CPR register).	Use the highest obtained education for the mother on the date of the child turns 10 years.<br>There is no information on this for children born 2007 or later.	Obtained from Register: Statistics Finland<br><br>Table:<br>Variable: ututku_aiti and koulutusaste_taso_1 and birthday of child obtained from population1	Education is classified by ISCED-11. although the classes 0-2 are not available for us. In Finland, we have compulsory education during which the ISCED level 2 is achieved and thus we classified education: NA=<br>1 low education level<br>3-4 = 2 medium education level<br>5-8 = 3 high education level<br>9 = no information of the mother’s Education when child is 10 years old.<br>If education was lower than m_education_b it was coded to be the same as at birth, also if education was unknown when child was ten, but it was known when child was born, the m_education_b was used as m_education_10y	Obtained from Statistics Norway<br>Variable: bu_niva_YYYY	Mother’s highest level of education as of October 1 in the year of child’s 10-year birthday was used to define m_education_b. For definitions and coding, see above. Available for children born 1990–2009.	As above.	As above, but from the year the child turned 10 years old.

Link to Contents

For peer review only - http://bmjopen.bmj.com/site/about/guidelines.xhtml

Link to Contents

Appendix: Vaccine categorization: presentation of ATC codes for vaccines identified in the vaccination registries in each of the Nordic countries and categorisation hereof into common vaccine categories “vaccine”. The vaccines are further grouped by “type” i.e. vaccines against a similar set of diseases. NB in Sweden only vaccines that are included in the national immunisation programme is registered in the vaccination register.

		**DENMARK**	**Finland**	**Norway**	**Sweden**
Vaccine	Type	ATC Code			
1= "DTaP-IPV-Hib"	1	J07CA06	J07CA06	J07CA06	J07CA06
2= "DTaP-IPV-Hib-HepB"		J07CA09		J07CA09	J07CA09
3= "DTaP-IPV"		J07CA02<br>J07CA02	J07CA02<br>J07CA02	J07CA02<br>J07CA02	J07CA02
4= "DT-Pol"		J07CA01		J07CA01	
5= "DT-HepB"		J07CA07			
6= "DTwP-HepB"		J07CA05			
7= "DTwP-Hib-HepB"		J07CA11			
8= "DTaP-IPV-HepB"		J07CA12			J07CA12
10= "DTaP"		J07AJ52	J07AJ52	J07AJ52	J07AJ52
11= "DTwP"		J07AJ51		J07AJ51	
12= "DT"		J07AM51	J07AM51	J07AM51<br>J07AM52	J07AM51
13= "D"		J07AF01		J07AF01	J07AF01
15= "T"		J07AM01		J07AM01	J07AM01
20= "PCV"	2	J07AL02	J07AL02	J07AL52<br>J07AL02	J07AL52<br>J07AL02
21="PPV"		J07AL01	J07AL01	J07AL01	J07AL01

Link to Contents

For peer review only - http://bmjopen.bmj.com/site/about/guidelines.xhtml

Link to *Contents*

25="HepA"	3	J07BC02	J07BC02	J07BC02	
26= "HepAB"		J07BC20	J07BC20	J07BC20	
27= "HepB"		J07BC01	J07BC01	J07BC01	
28= "HepA-Thyphoid"		J07CA10		J07CA10	
30= "HPV4"	4	J07BM01		J07BM01	J07BM01
31="HPV2"		J07BM02	J07BM02	J07BM02	J07BM02
32="HPV9"		J07BM03	J07BM03	J07BM03	J07BM03
35= "Hib"	5	J07AG01	J07AG01	J07AG01	J07AG01
36= "Hib-MenC"		J07AG53			
37= "Hib-Pol"				J07CA04	
38= "Hib-HepB"		J07CA08			
40= "Influenza (non-live)"	6	J07BB01<br>J07BB02	J07BB02	J07BB01<br>J07BB02	
41= "Influenza (live)"		J07BB03	J07BB03	J07BB03	
45= "wP"	7	J07AJ01		J07AJ01	
46= "aP"		J07AJ02		J07AJ02	
50= "MMR"	8	J07BD52	J07BD52	J07BD52	J07BD52
51= "MMR-Varicella"		J07BD54	J07BD54		
52= "Measles"		J07BD01		J07BD01	
53= "Measles-Mumps"		J07BD51		J07BD51	
54= "Measles-Rubella"		J07BD53		J07BD53	
55= "Rubella"		J07BJ01		J07BJ01	
56= "Mumps"		J07BE01		J07BE01	
60= "OPV"	9	J07BF01		J07BF04<br>J07BF01	

Link to *Contents*

Link to *Contents*

		J07BF02		J07BF02	
61= "IPV"		J07BF03	J07BF03	J07BF03	J07BF03
65= "Rota"		J07BH01<br>J07BH02	J07BH02	J07BH01<br>J07BH02	
67= "BCG"		J07AN01	J07AN01	J07AN01	
70="Varicella"		J07BK01<br>J07BK02	J07BK01	J07BK03<br>J07BK02<br>J07BK01	
71= "yellow fever"		J07BL01	J07BL01	J07BL01	
72= "Japanease Encephalitis"		J07BA02	J07BA02	J07BA02	
73= "Tick borne Encephalitis"		J07BA01	J07BA01	J07BA01	
74 = "Cholera"		J07AE51<br>J07AE02<br>J07AE01	J07AE01	J07AE01	
75= "Meningococcal vaccine"		J07AH08<br>J07AH09<br>J07AH03<br>J07AH04<br>J07AH06<br>J07AH05<br>J07AH02<br>J07AH01	J07AH08<br>J07AH09	J07AH07<br>J07AH08<br>J07AH09<br>J07AH03<br>J07AH04<br>J07AH06<br>J07AH01	
76= "Typhus"		J07AP01<br>J07AP10	J07AP01	J07AP01<br>J07AP02<br>J07AP	

Link to *Contents*

For peer review only - http://bmjopen.bmj.com/site/about/guidelines.xhtml

Link to Contents

		J07AP03		J07AP03	
77= "Rabies"		J07BG01		J07BG01	
78= "Smallpox"				J07B01	
		J07BX01			
79="Anthrax"				J07AC01	
80="covid-19 vaccine"			J07BX03	J07BX03	
99="other vaccines"		ATC code missing	ATC code missing	ATC code missing	ATC code missing

Link to Contents

For peer review only - http://bmjopen.bmj.com/site/about/guidelines.xhtml

Online supplementary files

sTable 1: Vaccination coverage[1] at 2 years of age according to year of birth among children born in the respective countries

sTable2: Human papilloma virus vaccination coverage[1] before 14 years of age of vaccination among girls[2] born in the respective countries

sTable 3: Socio-economic factors at 10 years of age

Appendix 3: ATC codes obtained for the study population in each country within NONSEnse

For peer review only

sTable 1: Vaccination coverage[1] at 2 years of age according to year of birth among children born in the respective countries

**Denmark**						
Year of birth	Eligible[2] Children	DTP1 % (95% CI)	DTP2 % (95% CI)	DTP3 % (95% CI)	MMR1 % (95% CI)	
1997	66,406	98.9	96.0	82.2	81.5	
1998	64,936	99.0	97.0	85.0	83.6	
1999	64,996	99.0	97.0	84.9	84.5	
2000	65,811	99.0	97.3	86.2	85.9	
2001	64,207	99.1	97.4	86.4	85.5	
2002	62,948	99.1	97.0	84.1	84.9	
2003	63,462	98.9	96.6	82.5	85.2	
2004	63,339	98.9	96.5	82.7	86.8	
2005	62,912	98.9	96.0	80.1	84.8	
2006	63,769	99.0	95.8	78.7	84.4	
2007	63,006	99.0	96.4	80.9	82.6	
2008	63,892	99.2	97.2	83.9	83.7	
2009	61,676	99.3	97.6	86.6	86.2	
2010	62,200	99.2	97.9	88.8	87.3	
2011	57,892	99.2	98.0	89.7	86.7	
2012	56,842	99.2	98.0	90.0	86.4	
2013	54,881	98.9	97.6	88.5	88.3	
2014	55,753	98.8	97.5	87.6	88.4	
2015	57,100	98.9	98.0	93.2	90.3	
2016	23,103	99.0	98.3	94.7	90.7	
**Finland**						
Year of birth	Eligible[2] Children	DTP1 % (95% CI)	DTP2 % (95% CI)	DTP3 % (95% CI)	MMR1 % (95% CI)	Rota virus vaccine % (95% CI)
2009	59,934	94.0	92.7	89.4	87.8	63.8
2010	60,560	96.7	94.2	90.8	91.6	90.7
2011	59,645	97.0	95.1	90.4	92.3	90.8
2012	59,309	95.7	94.5	90.3	91.9	91.2
2013	58,249	97.5	95.7	91.8	93.3	90.4

For peer review only

2014	57,693	97.6	96.5	89.3	92.6	91.6
2015	55,569	98.0	96.6	88.1	93.4	92.1
**Norway**						
Year of birth	Eligible[2] Children	DTP1 % (95% CI)	DTP2 % (95% CI)	DTP3 % (95% CI)	MMR1 % (95% CI)	Rota virus vaccine % (95% CI)
1995	59964	98.5	97.9	95.8	94.7	
1996	60652	98.2	97.4	95.4	94.3	
1997	59431	98.5	98.0	96.2	94.4	
1998	57999	98.7	98.2	96.4	94.1	
1999	58975	98.8	98.3	96.4	94.1	
2000	58907	98.6	98.0	96.1	89.2	
2001	56405	98.7	98.3	96.1	89.3	
2002	55232	98.8	98.5	96.7	92.7	
2003	56301	99.0	98.6	96.8	94.2	
2004	56734	99.1	98.8	97.5	94.7	
2005	56531	99.2	99.0	97.6	94.4	
2006	58316	99.1	98.8	97.3	94.2	
2007	58199	99.0	98.6	96.7	94.0	
2008	60284	99.0	98.5	96.6	93.8	
2009	61465	98.9	98.6	97.4	94.4	
2010	61080	98.9	98.5	97.3	95.0	
2011	59855	98.8	98.4	97.2	94.8	
2012	59937	98.6	98.2	96.6	95.0	
2013	58745	98.6	98.0	96.5	95.5	
2014	58839	98.7	98.3	96.6	95.9	
2015	58954	98.6	98.1	96.6	95.7	94.1
2016	58975	98.5	97.8	96.3	95.9	94.8
**Sweden**						
Year of birth	Eligible[2] Children	DTP1 N (%)	DTP2 N (%)	DTP3 N (%)	MMR1 (%)	
2013	113,457	97.6	95.1	83.3	89.1	
2014	114,639	98.0	95.9	86.1	90.7	
2015	114,542	98.1	96.3	87.8	91.8	

For peer review only

Abbreviations: DTP1: First dose of Diphtheria, Tetanus, and acellular Pertussis containing vaccine; DTP2: Second dose of Diphtheria, Tetanus, and acellular Pertussis containing vaccine; DTP3: Third dose of Diphtheria, Tetanus, and acellular Pertussis containing vaccine; MMR: Measles-Mumps-Rubella vaccine; Rota: Rota virus vaccine.

[1]The coverage reflects the number of registered vaccines and may thus underestimate the actual vaccination coverage in the countries. [2] Including children born in the country from birth cohorts where vaccines administered between 0-2 years of age are registered in the vaccination registers (data availability period).

For peer review only

sTable 2: Human papilloma virus vaccination coverage[1] before 14 years of age of vaccination among girls[2] born in the respective countries

	Denmark			Finland			Norway			Sweden		
Year of birth	Eligible[3] children	HPV1 vaccinated N (%)	HPV2 vaccinated N (%)	Eligible[3] children	HPV1 vaccinated N (%)	HPV2 vaccinated N (%)	Eligible[3] children	HPV1 vaccinated N (%)	HPV2 vaccinated N (%)	Eligible[3] children	HPV1 vaccinated N (%)	HPV2 vaccinated N (%)
1998	34,392	85.7	81.2				30,914	76.5 (76.0, 77.0)	75.8			
1999	34,484	86.8	82.4				31,391	78.5	77.7			
2000	34,881	86.5	82.3				31,490	80.2	79.5 (79.0, 79.9)			
2001	34,030	81.4	74.5				30,546	82.7	82.0			
2002	33,241	73.6	57.6	4053	71.1	69.3	30,213	84.5	83.7			
2003	33,762	52.3	36.1	27,310	69.8	67.2	30,925	84.8	84.0	53,623	77.9	72.4
2004	13,184	58.4	43.0				31,208	86.8	85.8			

Abbreviations: HPV1: First dose of Human papilloma virus vaccine; HPV2: Second dose of Human papilloma virus vaccine

[1]The coverage reflects the number of registered vaccines and may thus underestimate the actual vaccination coverage. [2] Including girls from birth cohorts where HPV vaccination has been offered from 1 year before age of recommended vaccination until 14 years of age and where vaccinations were registered in the vaccination registers. The years with available data is defined based on introduction of HPV vaccinations into the National immunization programme or introduction of vaccination register whichever comes last until last date with available data from both the population register and vaccination register.

For peer review only

sTable 3: Socio-economic factors at 10 years of age

	Denmark		Finland		Norway		Sweden	
	N	(%)	N	(%)	N	(%)	N	(%)
Children present in country at birth from 2004-2015	793,471		687,721		726,257		1,205,112	
Birth cohorts included	1994-2005		1994-2005		1994-2005		1994-2005	
Income quintile at 10 years of age								
First (lowest)	147,098	18.5%	135,946	19.8%	141,762	19.5%	219,673	18.2%
Second	149,579	18.9%	136,334	19.8%	146,247	20.1%	241,503	20.0%
Third	149,865	18.9%	136,384	19.8%	146,604	20.2%	245,097	20.3%
Fourth	149,310	18.8%	136,259	19.8%	146,471	20.2%	245,138	20.3%
Fifth	146,712	18.5%	135,464	19.7%	144,939	20.0%	241,452	20.0%
Unknown	50,907	6.4%	7334	1.1%	234	0.0%	12,249	1.0%
Number of children in the household the year the child turns 10 years of age								
1	103,585	13.1%	96,861	14.1%	119,295	16.4%	162,968	13.5%
2	405,367	51.1%	293,068	42.6%	336,480	46.3%	587,332	48.7%
3	213,413	26.9%	184,925	26.9%	208,045	28.6%	292,516	24.3%
>3	61,674	7.8%	103,561	15.1%	62,203	8.6%	105,595	8.8%
Unknown	9432	1.2%	9306	1.4%	234	0.0%	12,249	1.0%
Single parenthood in the years the child turns 10 years of age								
Yes	151,471	19.1%	124,986	18.2%	131,761	18.1%	268,484	22.3%
No	632,568	79.7%	553,429	80.5%	587,793	80.9%	924,379	76.7%
Unknown	9432	1.2%	9306	1.4%	6703	0.9%	12,249	1.0%
Highest attained educational level[1] of the mother on the date the child turns 10 years of age								
Low education	135,466	17.1%	75,462	11.0%	138,351	19.0%	193,551	16.1%
Medium education	340,574	42.9%	281,479	40.9%	270,114	37.2%	515,407	42.8%
High education	303,384	38.2%	329,885	48.0%	305,368	42.0%	394,220	32.7%
Unknown	14,047	1.8%	895	0.1%	12,424	1.7%	101,934	8.5%

[1] Highest attained education was categorized based on the International Standard Classification of Education (ISCED) 2011 using the main groups (1).

For peer review only

Appendix 3: ATC codes obtained for the study population in each country within NONSEnse

ATC-Group	Denmark	Finland	Norway	Sweden
D	D	D07, D11AH	D	D02AF, D05<br>D07, D11<br>D01, D06, D08
J	J	J	J	J01-J06<br>J07
R	R	R01, R03, R06	R	R01, R03, R06
S	S	S01G, S03	S	S01-S03
V	V01	V01[1]	V01	V01

[1] Data on redeemed prescriptions with ATC=V01 is only available from the Finnish Benefits Registry, which holds information only for reimbursable redeemed prescriptions.

For peer review only

References

1. UNESCO Institute for Statistics. International standard classification of education: ISCED 2011. 2012.
